# Geographical variation in metabolite profiles and bioactivity of *Thesium chinense* Turcz. revealed by UPLC-Q-TOF-MS-based metabolomics

**DOI:** 10.3389/fpls.2024.1471729

**Published:** 2025-01-10

**Authors:** Fang Zhang, Guanglei Zhang, Cong Wang, Haonan Xu, Ke Che, Tingting Sun, Qisheng Yao, Youyi Xiong, Niannian Zhou, Mengyuan Chen, Hao Yu, Hao Chen

**Affiliations:** ^1^ College of Life and Health Sciences, Anhui Science and Technology University, Fengyang, China; ^2^ School of Food Engineering, Anhui Science and Technology University, Fengyang, China; ^3^ Center of Molecular Metabolism, Nanjing University of Science and Technology, Nanjing, China; ^4^ College of Animal Science, Anhui Science and Technology University, Fengyang, China; ^5^ Planting Department, Jiuhua Huayuan Pharmaceutical Co., Ltd., Chuzhou, China; ^6^ School of Pharmacy, Anhui University of Chinese Medicine, Hefei, China; ^7^ Bozhou University, Bozhou, China

**Keywords:** *Thesium chinense* Turcz., UPLC-Q-TOF-MS/MS, geographical variations, non-targeted metabolomics, total flavonoid content, antioxidant, anti-inflammatory

## Abstract

**Introduction:**

This study aims to investigate the impact of geographical origin on the metabolite composition and bioactivity of Thesium chinense Turcz. (TCT), a member of the Apiaceae family renowned for its wide range of pharmacological properties, including antioxidant, antimicrobial, and anti-inflammatory effects. In this study, we investigated the whole plants of TCT from different regions in China, aiming to explore the geographical variation of TCT.

**Methods:**

A non-targeted metabolomics approach was employed using ultra-high-performance liquid chromatography combined with quadrupole time-of-flight mass spectrometry (UPLC-Q-TOF-MS). Principal component analysis (PCA) and partial least squares discriminant analysis (PLS-DA) were utilized to identify and differentiate the metabolite profiles. We investigated the bioactivity, antioxidant activity, total flavonoid content (TFC), and the content of characteristic compounds from TCT sourced from different regions. This aims to further explore the metabolic differences and quality characteristics of TCT from various origins.

**Results:**

PCA and PLS-DA analyses indicated that samples from different origins could be clearly distinguished. The analysis revealed 54 differential metabolites, predominantly flavonoids and alkaloids. KEGG pathway analysis indicated significant variations in the biosynthesis pathways of flavonoids and flavanols among the samples. TCT from Anhui province exhibited the highest TFC and strongest antioxidant and anti-inflammatory activities, while samples from Jilin province showed the lowest.

**Discussion:**

A strong correlation was observed between metabolite content and geographical origins, suggesting that the bioactivity of TCT is significantly influenced by its provenance. Additionally, the antioxidant and anti-inflammatory activities of TCT were validated, showing a strong predictive relationship with TFC. This research highlights the potential of metabolomics in discerning the subtleties of plant metabolomes, contributing to the advancement of traditional Chinese medicine and its integration into modern healthcare practices.

## 1 Introduction

*Thesium chinens*e Turcz. (TCT), known in Chinese as “Bai Rui Cao,” is the dried whole herb of an Apiaceae plant recognized for its diverse pharmacological properties. With a long history in traditional Chinese medicine, TCT has been used to treat various human ailments. Chinese ethnomedical texts such as ‘Ben Cao Tu Jing’ and ‘Guo Yao Ti Yao’ have extensively documented its medicinal applications (Li et al., 2021). In traditional Chinese medicine, TCT, often referred to as “Botanical Antibiotics,” is primarily used to treat conditions including mastitis, pulmonitis, tonsillitis, laryngopharyngitis, and upper respiratory tract infections (Parveen et al., 2007; Liu et al., 2023). Previous research has identified various bioactive compounds in TCT, including polysaccharides, flavonoids, D-mannitol, terpenoids, alkaloids, aromatic compounds, and aliphatic acids (Lombard et al., 2020; Lombard et al., 2022). Flavonoids are the principal components of this plant, significantly contributing to the biological efficacy of TCT. Nineteen distinct flavonoids have been isolated from TCT, predominantly as flavones and flavonols, including kaempferol, astragalin, afzelin, and rutin, among others. Several modern pharmaceutical formulations incorporating TCT, such as Bairui Pills and Bairui Granules, have been employed clinically for the treatment of acute and chronic pharyngitis, fever, bronchitis, pulmonitis, and rhinitis (Li et al., 2021). Notably, TCT exhibits a wide array of pharmacological functions characteristic of traditional Chinese medicine, including antioxidant properties, anti-inflammatory activity, and the mitigation of oxidative stress (Li et al., 2024). Liu M.J. et al. found that the ethyl acetate fraction of TCT significantly mitigated the progression of chronic obstructive pulmonary disease by inhibiting ferroptosis, primarily through the activation of the Nrf2/SLC7A11/GPX4 signaling pathway. The main active components responsible for these effects are flavonoids (Liu et al., 2024). However, there is currently limited research comparing TCT from different geographical origins. This study aims to evaluate TCT from various regions through metabolomics and biological activity testing.

The plant metabolome, which consists of primary and secondary metabolites, is the ultimate recipient of biological information and affects protein stability, metabolic changes, and gene expression (Hill et al., 2015; Li-Beisson et al., 2024). These metabolites fulfill various physiological roles, such as maintaining cellular structural integrity, serving as mobile signals, participating in defense mechanisms, and supporting basic cellular functions (Hong et al., 2016). Metabolomics, involving the qualitative and quantitative analysis of low molecular weight molecules (below 1000 Da), offers valuable insights into the cellular state and biochemical processes of plants. This high-throughput, high-sensitivity technology is applicable in fields such as food science, disease diagnosis, botany, and toxicology (Zhang et al., 2012; Tenenboim and Brotman, 2016). It reveals the impact of external and internal disturbances on plant metabolism, aligning with the holistic perspective of traditional Chinese medicine (Chen et al., 2020). Untargeted metabolomics, using UPLC-Q-TOF-MS/MS and multivariate statistical analysis, has been employed to trace the geographic origins of various food products (Zhao et al., 2020; Liu et al., 2022). Thus, metabolomic comparative assessments are feasible for evaluating intraspecific and interspecific differences in plants from different geographical locations.

Despite the extensive research on the chemical composition and pharmacological effects of TCT (Lombard et al., 2022; Peng et al., 2024), there is a notable gap in the literature regarding the use of metabolomics for quality assessment of this herb. Previous studies have not fully explored the geographical variations in the metabolite profiles of TCT and their impact on its bioactivity. This study aims to fill this gap by employing a non-targeted metabolomics approach using UPLC-Q-TOF-MS/MS to analyze TCT samples from seven Chinese provinces.

The present study aimed to achieve the following objectives (i) harness the power of UPLC-Q-TOF-MS/MS to provide a detailed overview of the metabolite profiles. This cutting-edge method affords us the capability to identify a vast array of metabolites, thus unveiling the rich biochemical tapestry of TCT and offering deeper insights into its inherent diversity. (ii) A critical aspect of our research was to underscore the profound impact of geographical provenance on the metabolite composition and bioactivity of TCT. By employing PCA and PLS-DA, we sought to distinguish the metabolite profiles based on their geographical origins. These sophisticated analytical tools provide a robust framework for the quality assessment of TCT, enabling the differentiation of samples with precision. (iii) Furthermore, we endeavored to establish a correlation between the metabolite composition and the antioxidant and anti-inflammatory activities intrinsic to TCT. By quantifying the TFC and aligning these measurements with our metabolomic findings, we aimed to construct a predictive model that links the biochemical makeup of TCT to its biological effects.

## 2 Materials and methods

### 2.1 Material

Fifteen batches of fresh TCT samples were purchased from seven regions, which were listed in **Table 1**. Three samples were collected from each city, with the selection criteria being two-year-old, dried whole plants measuring 25-30 cm in length. These acquisitions were made between September and October 2022. All TCT samples were identified by Professor Hanzhen Liu and stored in the laboratory of Anhui Science and Technology University. In subsequent *in vitro* anti-inflammatory and antioxidant experiments, Shangluo was selected to represent Henan province, Xian was selected to represent Shaanxi province, and Lvliang was selected to represent Shanxi province for the sake of convenience in comparison. Reference substances, including Afzelin (C21H20O10, FY144B01125), Kaempferol (C15H10O6, FY67BD803), Rutin (C27H30O16, FY22B712) and Astragalin (C21H20O11, FY01B1122) were purchased from Nantong Feiyu Biological Technology Co., Ltd., with a purity of above 98%. HPLC grade Acetonitrile was purchased from Thermo Fisher Scientific Co. Phosphoric acid was purchased from Sinopharm Chemical Reagent Co., Ltd.

**Table 1:** Sampling locations and corresponding coordinates of the samples.

NO.	Sampling Locations	Latitude and Longitude
1	Chuzhou, Anhui	118.34068, 32.31163
2	Chuzhou, Anhui	118.34261, 32.31085
3	Luanchuan, Henan	111.62243, 33.79182
4	Lvliang, Shanxi	111.15045, 37.52450
5	Daning, shanxi	110.75938, 36.47131
6	Hongdong, Shanxi	111.68159, 36.25947
7	Lushi, Henan	111.05456, 34.05992
8	Shangluo, Henan	109.92441, 33.87863
9	Fuxian, Shaanxi	109.00426, 35.96442
10	Baoji, Shaanxi	107.24457, 34.36892
11	Xian, Shaanxi	108.83852, 34.52957
12	Qingyang, Gansu	107.64938, 35.71522
13	Jilin	126.55563, 43.84357
14	Wuhan, Hubei	113.83259, 31.62248
15	Chuzhou, Anhui	118.27538, 32.29353

### 2.2 Sample treatment

For the fifteen batches of TCT samples, processing was conducted under a liquid nitrogen environment. Initially, the whole plant hay of TCT was ground into a fine powder using a mortar. Subsequently, 15 mg of the TCT powder was transferred into a EP tube. 1 mL mixture of chloroform, water, and methanol (20/20/60, v/v/v) was added along with tungsten carbide beads. The samples were then dried under a stream of nitrogen gas, followed by the addition of 500 μL of 50% methanol for reconstitution. The samples underwent ultrasonication for 30 seconds and were centrifuged at 12,000 rpm at 4 °C for 15 minutes to remove debris particles. Finally, the supernatant was transferred to UPLC vials for liquid chromatography-mass spectrometry analysis.

### 2.3 UPLC-Q-TOF-MS/MS analysis

Sample analysis was conducted using the SCIEX Exion LC UPLC system, fitted with a Phenomenex Kinetex^®^ C18 column (100 × 2.1 mm, 2.6 μm) and a Security Guard UPLC C18 guard column. The column temperature was consistently maintained at 40 °C throughout the analysis. The mobile phase system included 0.1% formic acid in water (solvent A) and acetonitrile (solvent B), following the gradient elution program detailed in **Table 2**. The flow rate was set to 0.4 mL/min, with injection volumes of 1 μL for positive ion mode and 2 μL for negative ion mode. The temperature of the automatic sampler was kept at 4 °C for the duration of the analysis.

**Table 2:** Gradient elution flow conditions.

Time (min)	Linear Gradient (B%)
0	1
1	1
10	99
13	99
14	1
17	1

A hybrid quadrupole time-of-flight mass spectrometer equipped with a Duospray ion source (Triple TOF™ 5600^+^; AB Sciex, USA) was employed for data acquisition in both positive and negative ionization modes. The conditions for the ESI source were established as follows: nitrogen gas was used for nebulization and auxiliary gases, with nitrogen gas as nebulizer gas 1 at 55 psi, auxiliary gas at 55 psi, curtain gas at 35 psi, ion source temperature at 550°C, and spray voltage at 5500 V (+)/-4500 V (–).

In TOF MS-IDA-MS/MS acquisition, the TOF MS spectra were scanned within a mass range of 50 to 1000 m/z, with an accumulation time of 0.10 s per spectrum. The product ion spectra were acquired in a mass range of 40 to 1000 m/z, with an accumulation time of 0.05 s per spectrum. Information-dependent acquisition (IDA) was employed in high-sensitivity mode for product ion scanning. Parameters for the IDA mode were configured as follows: collision energy of 35 ± 15 V (+)/-35 ± 15 V (–), collision voltage offset of 80 V (+)/-80 V (–), ion intensity threshold not lower than 100 cps, exclusion of isotopes within 4 Da, ion tolerance of 50 mDa, and monitoring of 10 candidate ions per cycle. Dynamic background subtraction (DBS) was enabled to enhance sensitivity for low-abundance or trace-level analytes. External calibration using the external calibration system was performed every five samples for automatic calibration of TOF MS and TOF MS/MS spectra.

### 2.4 Data processing, statistical analysis and metabolite identification

Data acquisition was performed using SCIEX Analyst 1.8.1 Software, followed by converting the raw data files in wiff format to MZML format. Subsequently, peak discovery, filtering, and alignment were conducted using the open-source R-Package. The processed data were then subjected to standardized analysis using MetaboAnalyst 5.0. The standardization process involved three distinct steps: sample standardization, data transformation, and data scaling. Sample standardization allowed for overall adjustment of differences between samples, while data transformation and scaling often rendered individual features more comparable. Therefore, sample normalization was conducted using the median of samples, data were transformed by logarithmization, and automatic scaling was applied.

Unsupervised PCA was performed using MetaboAnalyst 5.0 to gain a general understanding of relationships among the data matrices. Subsequently, PLS-DA was employed to investigate metabolic differences among TCT samples from seven regions. In brief, data were imported into SIMCA 14.1, models and classes were selected, and the “auto-adjust” option was chosen. This option provided all relevant features of the model, including 1 predictive component and 2 orthogonal components. Variable Importance in Projection (VIP) was crucial in explaining the data obtained from PLS-DA. Metabolites considered important in distinguishing TCT samples from seven regions were those with VIP values exceeding 1 (VIP > 1.0), with a p-value < 0.001 (independent samples t-test).

Metabolites meeting these criteria were considered differential metabolites. Identification was performed using the Metlin database and relevant published literature, based on mass spectrometry data. Confirmation of identification was carried out by matching retention times and fragmentation patterns.

### 2.5 KEGG metabolic pathway enrichment

Metabolites were annotated using the Kyoto Encyclopedia of Genes and Genomes (KEGG) compound database. These annotated metabolites were subsequently matched to the KEGG pathway database. Significantly regulated metabolites were then imported into MetaboAnalyst 5.0 for pathway enrichment analysis. The enrichment results, calculated for significance (p-values) using hypergeometric tests, were presented as bubble plots.

### 2.6 Determination of antioxidant activity

#### 2.6.1 DPPH radical scavenging activity assay

50 mg of TCT powder from different batches of seven origins was weighed. Following the manufacturer’s instructions for the reagent kit (Yuanye, China), nitrogen radical extraction solution and DPPH solution were added to each sample. The absorbance at a wavelength of 517 nm was measured. A control sample, which did not contain DPPH solution, was used for comparison. The DPPH scavenging rate was calculated by comparing the absorbance of the samples to that of the control.

#### 2.6.2 Hydroxyl radical scavenging assay

The hydroxyl radical scavenging activity was measured by the Fenton reaction (Sanna and Fadda, 2022). A total of 100 mg of TCT powder was weighed and extracted ultrasonically in 4 mL of methanol. The supernatant was collected and diluted. Subsequently, following the manufacturer’s instructions for the reagent kit (Jiancheng, China), double-distilled water and substrate application solution were added to the diluted supernatant, and the mixture was incubated at 37 °C for 1 minute. The reaction was immediately terminated by adding a chromogenic agent. The hydroxyl radical scavenging ability of the samples was determined by comparing their absorbance with that of a control group (without sample) and a blank group, following standard procedures.

### 2.7 Determination of anti-inflammatory activity

#### 2.7.1 Cell viability assays

HUVECs were provided by the Junsong Wang research group at Nanjing University of Science and Technology and cultured in H-DMEM (Gibco, USA) supplemented with 10% fetal bovine serum (FBS) (Gibco, USA). The 7th-10th generation cells were used in the experiments. HUVECs were incubated with different concentrations of TCT (0, 50 ng/mL, 500 ng/mL, 5 μg/mL, 50 μg/mL and 500 μg/mL) for 24 h, and cell viability was determined by Cell Counting Kit-8 (CCK-8) assay. CCK-8 (Beyotime, China) was used according to the manufacturer’s instructions. The cells were incubated with 10 μL CCK-8 in culture medium at 37 for 2 h. The resulting absorbance was measured at 450 nm.

#### 2.7.2 LPS-induced inflammation model and drug treatment

Based on previous cell viability assays, it was observed that HUVECs viability begins to decline at a concentration of 500 μg/mL (**Supplementary File 1**). Therefore, for subsequent experiments, 5 μg/mL was set as the low-dose group and 50 μg/mL was set as the high-dose group. After two washes with phosphate-buffered saline (PBS, pH 7.4), the cells were exposed to 10 μg/mL LPS (Sigma, USA) and different doses of TCT from various origins, diluted in culture medium, for 24 hours at 37°C, thereby establishing a model of inflammatory and injured endothelium. After removing the culture medium, 500 µL of TRIzol (Beyotime, China) was added to the cells, and the samples were subsequently frozen at -80°C.

#### 2.7.3 Quantitative real-time PCR

Total RNA was extracted from cell samples of each experimental group, and 5 µg of total RNA was reverse transcribed into cDNA using a reverse transcription kit (Vazyme) following the manufacturer’s instructions. RT-qPCR was carried out in a 25 µL reaction system, which included SYBR Green/Fluorescein qPCR Master Mix, forward and reverse primers, and the synthesized cDNA. PCR amplification was performed using the ABI7500 real-time PCR system (Applied Biosystems). The relative mRNA expression levels were calculated using the 2^(-ΔΔCt) method, with GAPDH serving as the endogenous control for normalization. The specific PCR primers used in this study are listed in **Supplementary File 2**.

### 2.8 Determination of total flavonoid content

TFC was determined using the aluminum chloride colorimetric method. Pipette 0.3 mL of the TCT methanol extract prepared under section 2.6.2, and add 3.4 mL of 30% methanol, 0.15 mL of 0.5 M NaNO_2_, and 0.15 mL of 0.3 M AlCl_3_·6H_2_O. After 5 min, 1 mL of 1 M NaOH was added to the mixture, and absorbance was measured at 510 nm. The TFC was calculated from a calibration curve of Rutin within the concentration range of 0.0016-1 mg/mL (calibration curve: y = 0.7276 x + 0.0522, R2 = 0.9963). The results are expressed as milligrams of Rutin equivalents (RE) per 75 mg dry weight of the sample (mg RE/75 mg DW).

### 2.9 Quantification of characteristic chemical components in TCT

#### 2.9.1 Sample preparation

Accurately weigh 2.00 g of powdered sample and add it to 40 mL of methanol for extraction. The liquid-to-solid ratio is 1:20. Perform ultrasonic extraction at 40 kHz for 1 hour. Filter the extraction solution and then transfer it into a 50 mL volumetric flask. Make up the volume with methanol and mix thoroughly. Repeat this procedure twice for each group and combine the extraction solutions, containing 0.02 g of crude drug per mL. The final concentrated solution was subjected to HPLC analysis after filtration through a 0.45 μm membrane filter.

#### 2.9.2 Apparatus and chromatographic conditions

The extracting solution was analyzed using a Shimadzu LC 20AT HPLC system (Shimadzu, Kyoto, Japan) equipped with a UV detector, a binary pump, and an automatic sampler. Chromatographic separation was performed on an Agilent SB-C18 column (4.6 × 250 mm, 5.0 µm) using Solvent-B (acetonitrile) and Solvent-A (0.5% phosphoric acid aqueous solution). The column temperature was maintained at 30 °C, with an injection volume of 15 µL and a flow rate of 1.0 mL/min. Binary gradient elution was applied as follows: a linear gradient starting from 5% B at 0.01 min and increasing to 5% B at 5 min, 15% B at 10 min, 55% B at 40 min, and 5% B at 42 min, maintained at 5% B for 8 minutes (from 42 to 50 min). Detection was carried out at a wavelength of 254 nm. Solvent-A was degassed using ultrasonic cleaners (Skymen, Shenzhen, China) for 20 min before use.

### 2.10 Network pharmacology analysis of characteristic chemical components

#### 2.10.1 Acquisition of active ingredients

The SMILES names of these ingredients (Rutin, Kaempferol, Afzelin and Astragalin) were searched in the PubChem database (https://pubchem.ncbi.nlm.nih.gov/). The SMILES names were further input into the SwissTargetPrediction database (http://www.swisstargetprediction.ch/) to obtain their target proteins. The obtained active ingredients targets were intersected to obtain the common targets. The Venn diagram of the intersection between these ingredients targets was generated using the Venny platform (https://bioinfogp.cnb.csic.es/tools/venny/).

#### 2.10.2 Disease enrichment analysis

The intersected targets were input into the DAVID database (https://david.ncifcrf.gov/). All the 26 enriched terms were plotted using bioinformatics platforms.

### 2.11 Statistical analysis

Data were represented as mean ± SEM. The statistical graphs and chromatograms were performed using GraphPad Prism 9 and OriginPro 9.0, respectively. Student’s t-test was used for comparisons between two groups. For multiple group comparisons, one-way analysis of variance (ANOVA) was performed followed by Tukey’s *post hoc* test to calculate p values and determine differences among groups.

## 3 Results

### 3.1 Metabolite profiling overview

**Figure 1:**
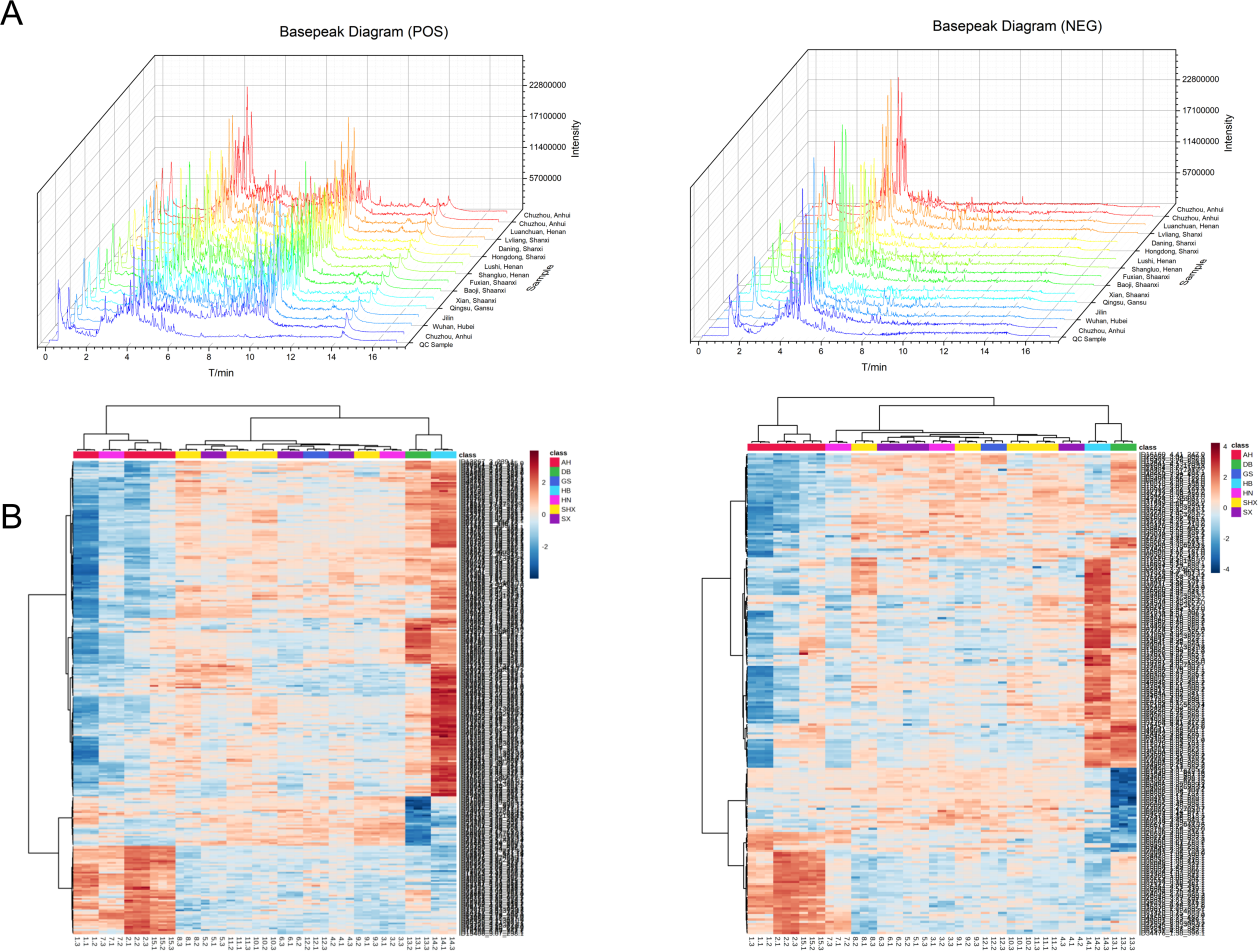
Metabolite profiling of TCT samples from different geographical origins. **(A)** Basepeak diagrams of 15 batches of TCT samples and QC samples. **(B)** Heatmap of mass spectral data from TCT samples across seven regions. The heatmap, with distinct colors representing samples from different geographical origins.

It is well known that UPLC-Q-TOF-MS/MS is an effective tool for analyzing various metabolites because of its high selectivity and sensitivity (Bhatia et al., 2019). In this work, a high-throughput untargeted analytical strategy based on UPLC-Q-TOF-MS/MS was established to comprehensively acquire secondary metabolites from TCT samples of various origins. The basepeak diagrams of TCT samples from the fifteen different regions and QC sample in a stacked format were shown (**Figure 1A**). Under the current experimental conditions, a substantial amount of secondary mass spectral information was detected, indicating that the established UPLC-Q-TOF-MS/MS method could yield rich chemical information from TCT samples. And all batch sample Total Ion Chromatogram (TIC) graphs can be found in **Supplementary File 3**. Notably, there were significant differences in the peak heights of the secondary metabolite fingerprint profiles among TCT samples from different regions. However, the metabolite peak retention times of TCT from different origins largely overlapped, indicating that the differences lie not in the presence or absence of certain metabolites but in the levels of metabolites. This suggests that the effective components within TCT vary with geographical distribution, ultimately leading to differences in metabolic fingerprints.

Heatmap of mass spectral data from TCT samples across seven regions is presented in **Figure 1B**, where different colors represent samples from various geographical regions. Under the positive ion mode, a total of 289 compounds were co-identified, whereas under the negative ion mode, 217 compounds were co-identified. Distinct colors in the heatmap correspond to TCT samples from various geographic regions, and the heatmap incorporates clustering information. Preliminary examination of the PCA scores plot for both positive and negative ions reveals that there are discernible chemical compositional differences among TCT samples from the seven regions. Notably, these differences exhibit a certain degree of geographical correlation.

### 3.2 Metabolic phenotype differences

**Figure 2:**
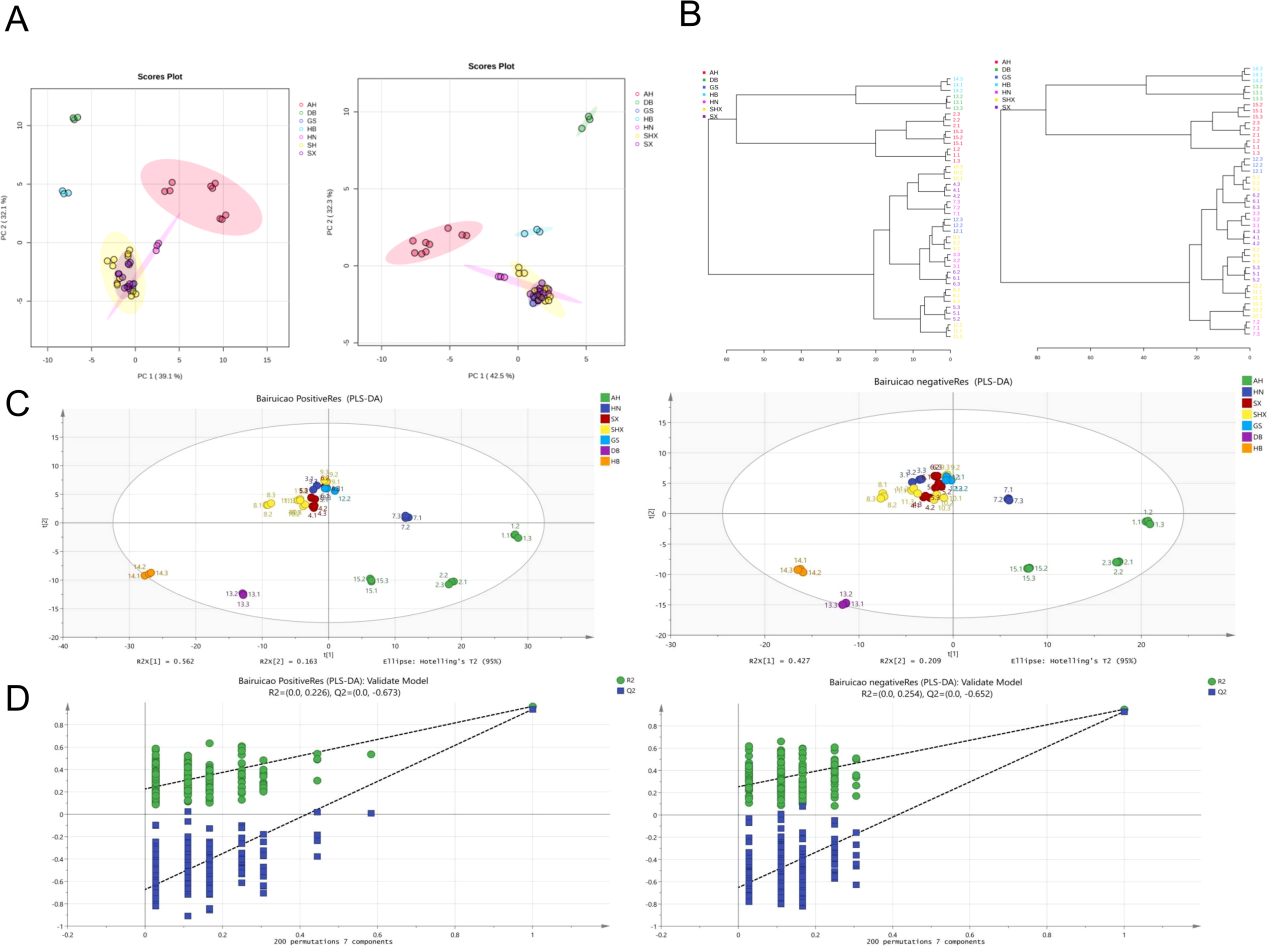
Multivariate analysis of TCT samples from various geographical regions. **(A)** PCA score plot of TCT samples in POS and NEG ion modes. **(B)** Hierarchical clustering dendrogram of TCT samples in POS and NEG ion modes, based on Euclidean distance and Ward’s clustering algorithm. **(C)** PLS-DA score plot of TCT samples, demonstrating clear differentiation among samples from seven regions. **(D)** Permutation test results (n=200) for the PLS-DA model.

PCA was employed to analyze the data, and **Figure 2A** illustrates the PCA plot for TCT samples from different geographic origins. In the positive ion mode (POS), PC1 contributed to 39.1% of the variance, while PC2 contributed to 32.1%. In the negative ion mode (NEG), PC1 accounted for 42.5% of the variance, and PC2 contributed to 32.3%. Each data point represents an individual sample. From the preliminary PCA model of TCT samples, it is evident that there are significant differences among samples from Anhui, Jilin, and Hubei regions. Conversely, samples from Gansu, Shanxi, Shaanxi, and Henan regions cluster closely together, indicating similar compositional components and contents. This clustering may be attributed to the geographical proximity of the sampling locations within a radius of 200 kilometers in these four regions. However, these four regions exhibit distinct differences compared to Anhui, Jilin, and Hubei regions.

To further validate these findings, hierarchical clustering was performed using MetaboAnalyst 5.0 with the following parameters: Distance measure–Euclidean; Clustering algorithm–Ward. The resulting dendrogram, shown in **Figure 2B**, supports the conclusions drawn from PCA. Samples from Gansu, Shanxi, Shaanxi, and Henan regions exhibit higher similarity, with consistent dendrogram patterns in both positive and negative ion modes.

PCA is an unsupervised model that takes into account all variables, while PLS-DA is a supervised discriminant analysis statistical method that filters systematic noise and extracts variable information. Consequently, PLS-DA models exhibit superior classification efficiency compared to PCA models. We applied PLS-DA to establish relationship models between metabolite expression levels and sample categories, enabling the prediction of sample categories.

PLS-DA models were validated using SIMCA 14.1 software. The results indicated that in the POS ion mode, the parameters R2Xcum, R2Ycum, and Q2cum were 0.891, 0.837, and 0.6, respectively. In the NEG ion mode, the parameters R2Xcum, R2Ycum, and Q2cum were 0.853, 0.902, and 0.717, respectively. These results demonstrated that the PLS-DA models possessed strong classification capabilities (**Figure 2C**).

Analysis using the PLS-DA model revealed clear differentiation among TCT samples from seven regions. This outcome suggests the presence of metabolic phenotypic differences among the seven groups. To further validate the model, permutation tests (n=200) were conducted, yielding R2 values ranging from 0.0 to 0.254 and Q2 values ranging from 0.0 to -0.652. These results indicate the stability and reproducibility of the PLS-DA model, with no evidence of overfitting, as shown in **Figure 2D**.

In this study, PCA and PLS-DA were combined, with PCA being used for an initial exploration of the overall view and PLS-DA being employed for more detailed classification and feature selection. This combined approach enhances our understanding of the data and improves the accuracy of the analysis.

### 3.3 Screening of differential metabolites

Multivariate analysis was performed on the VIP values obtained from the aforementioned PLS-DA model, with VIP values ranked in descending order. A higher VIP value indicates a greater contribution to the model. Components with VIP values ≥ 1 and P-values < 0.05 were selected. A total of 54 differential metabolites were identified, with flavonoids comprising 18 of them, accounting for 33.33% of the total number of constituents. Including typical flavonoid compounds such as Kaempferol, Apigenin, Rutin, Kaempferol-3-O-glucorhamnoside, and Astragalin, Afzelin, and 6’’-O-L-Arabinopyranosylastragalin (Kaempferol 3-O-vicianoside), among others. Flavonoids are characterized by a tricyclic structure (C6-C3-C6), represented as A, B, and C in their basic framework (Shen et al., 2022). Flavonoids are groups of polyphenolic hydroxylated compounds characterized by a benzo-π-pyrone backbone. These compounds are ubiquitous in plants and are produced via the phenylpropanoid pathway (Liga et al., 2023). The metabolism, biological activity, and bioavailability of flavonoids are closely linked to their structure. Flavonoids suitable for human consumption are commonly found in vegetables and fruits (Sumanta and Syed, 2020). These compounds can be utilized in the treatment of COVID-19, pregnancy-induced disorders, and neurodegenerative disorders. They have demonstrated significant antihypertensive, anticancer, antioxidant, and anticholinesterase properties (Achika et al., 2023). A total of 11 alkaloid compounds, constituting 20.37% of the composition, were identified, including Oxysophocarpine, Vincanidine, Myristoylcarnitine, Vinpocetine, Pyridoxal, among others. Alkaloids play a crucial role in both human medicine and the natural defense mechanisms of organisms. Caffeine and cocaine are used clinically as stimulants and sedatives, morphine is also commonly used as a powerful analgesic for pain relief, and nicotine, an important alkaloid, is also used to treat ulcerative colitis (Hilal et al., 2024). In plants, alkaloids serve to protect them from predators and regulate their growth (Chik et al., 2013). In the realm of therapeutics, alkaloids are widely recognized for their use as anesthetics, cardiac protectants, and anti-inflammatory agents. Well-known alkaloids used in clinical practice include vinblastine, vinorelbine, vincristine, and vindesine (Mondal et al., 2019). There are a total of 7 organic acid compounds, accounting for 12.96% of the total. These include 16-Hydroxyhexadecanoic acid, Hexadecanedioic acid, Heneicosanoic acid, cis-Aconitic acid, 2-(4-Hydroxyphenyl) propanoic acid, and others. Organic acids are characterized by the presence of carboxyl groups (-COOH). Many medicinal herbs with a sour taste contain organic acid components, with common examples being citric acid, malic acid, succinic acid, tartaric acid, and oxalic acid. China boasts abundant natural resources of organic acids, and a variety of organic acid compounds have been discovered in traditional Chinese medicine (Shang et al., 2011). These compounds exhibit pharmacological effects including anti-inflammatory, antioxidant, anti-platelet aggregation, and anti-thrombotic properties (Zou et al., 2024). Additionally, there are 2 terpenoid compounds, constituting 3.70% of the total, such as Phytol and Valtrate, terpenoid compounds are natural plant-derived secondary metabolites. They constitute a chemically diverse group of secondary metabolites which are biosynthesized in the flowering shoots, roots or rhizomes. Such plant terpenoid compounds most commonly occur in a cyclic form (Zeng et al., 2019). Due to this structural variation, they also demonstrate a range of pharmacological and biological activities, including antitumor (paclitaxel) (Kingston, 1994), positive inotropic (forskolin) (Lindner et al., 1978), vasodilatory, and hypotensive (manool) properties (Monteiro et al., 2020). There are 16 compounds belonging to other categories, accounting for 29.63% of the total. These include 1,2-Dihydroxyheptadec-16-yn-4-yl acetate, Loliolide, and others. All the 54 differential metabolites were listed in **Table 3**, and the structures of these compounds and their secondary mass spectrometry information are listed in **Supplementary File 4**.

**Table 3:** Detailed information of the 54 differential metabolites.

No.	Compound Name	Formula	RT	MW	Adducts	Mass Error (ppm)	m/z	p-value	VIP	ion modes
1	Oxysophocarpine	C_15_H_22_N_2_O_2_	2.955	262.35	[M+H]+	-1.09	263.1751	0.000626	1.3605	POS
2	2,2-Difluoro-1,3-benzodioxole-4-carboxylic acid	C8H4F2O4	3.103	202.11	[M+H]+	0.74	203.0152	4.23E-12	1.0768	POS
3	Neodiosmin	C_28_H_32_O_15_	3.137	608.5	[M+H]+	0.03	609.1814	0.000797	1.2258	POS
4	Baohuoside I	C_27_H_30_O_10_	3.441	514.5	[M+Na]+	0.34	537.1727	8.02E-11	1.0293	POS
5	kaempferol	C_15_H_10_O_6_	3.492	286.24	[M+H]+	-0.29	287.0549	0.000508	1.2151	POS
6	Sanggenone H	C_20_H_18_O_6_	3.507	354.4	[M+CH3OH+H]+	1.48	387.1444	4.23E-08	1.6431	POS
7	3,4,5-Trihydroxy-1-cyclohexene-1-carboxylic acid	C_7_H_10_O_5_	3.73	174.15	[M+H]+	2.74	175.0610	2.74E-17	1.0545	POS
8	Myristicin	C_11_H_12_O_3_	3.737	192.21	[M+H]+	-3.94	193.0852	3.59E-06	1.7053	POS
9	13-alpha-(21)-Epoxyeurycomanone	C_20_H_24_O_10_	3.834	424.4	[M+H]+	2.36	425.1452	2.15E-08	1.3866	POS
10	Sophoraflavonoloside	C_27_H_30_O_16_	3.859	610.5	[M+H]+	2.57	611.1622	3.24E-05	1.3312	POS
11	Loliolide	C_11_H_16_O_3_	4.221	196.24	[M+H]+	0.86	197.1174	0.002305	1.1425	POS
12	Ferulic acid	C_10_H_10_O_4_	4.26	194.18	[M+H]+	2.43	195.0657	7.34E-11	1.7858	POS
13	Kaempferol 3-O-vicianoside	C_26_H_28_O_15_	4.392	580.5	[M+H]+	0.49	581.1504	9.79E-11	1.8086	POS
14	Piceid	C_20_H_22_O_8_	4.402	390.4	[M+H]+	1.59	391.1394	0.000964	1.0822	POS
15	Lespedin	C_27_H_30_O_14_	4.594	578.5	[M+H]+	2.39	579.1722	6.94E-14	1.2153	POS
16	Vincanidine	C_19_H_20_N_2_O_2_	4.612	308.4	[M+H]+	-2.60	309.1589	5.01E-06	1.0835	POS
17	3,4,5-Trimethoxybenzoic acid	C_10_H_12_O_5_	4.65	212.2	[M+H]+	0.84	213.0759	0.010923	1.2104	POS
18	16-Hydroxyhexadecanoic acid	C_16_H_32_O_3_	4.685	272.42	[M+H]+	1.77	273.2429	4.93E-08	1.0838	POS
19	Hexadecanedioic acid	C_16_H_30_O_4_	4.695	286.41	[M+H]+	2.33	287.2224	1.58E-06	1.0465	POS
20	4-(Tridecanoylamino)benzoic acid	C_20_H_31_NO_3_	5.004	333.5	[M+H]+	2.01	334.2383	3.56E-09	1.0672	POS
21	Heneicosanoic acid	C_21_H_42_O_2_	5.189	326.6	[M+H]+	-4.58	327.3243	1.23E-08	1.0256	POS
22	2-Amino-1-phenylethanol	C_8_H_11_NO	5.202	137.18	[M+NH4]+	-0.18	155.1173	5.15E-10	1.3795	POS
23	Valtrate	C_22_H_30_O_8_	5.25	422.5	[M+2H]2+	1.94	212.1050	3.42E-05	1.1788	POS
24	7-Hydroxy-3-(2-hydroxy-propyl)-5-methyl-isochromen-1-one	C_13_H_14_O_4_	5.328	234.25	[M+H]+	2.04	235.0970	1.30E-08	1.0485	POS
25	Phytol	C_20_H_40_O	5.596	296.5	[M+H-H2O]+	0.67	279.3048	7.07E-05	1.1856	POS
26	15-Kete	C_20_H_30_O_3_	5.678	318.4	[M+H]+	1.71	319.2273	1.50E-06	1.0095	POS
27	Okaramine D	C_33_H_34_N_4_O_6_	5.72	582.6	[M+H]+	0.5328	583.2554	6.79E-19	1.1622	POS
28	Afzelin	C_21_H_20_O_10_	5.852	432.4	[M+H]+	0.36	433.1131	1.82E-13	1.7514	POS
29	Anileridine	C_22_H_28_N_2_O_2_	6.006	352.5	[M+Na]+	-1.19	375.2033	2.26E-06	1.0451	POS
30	Tricin 5-glucoside	C_23_H_24_O_12_	6.093	492.4	[M+H]+	1.20	493.1346	3.93E-11	1.1085	POS
31	1,2-Dihydroxyheptadec-16-yn-4-yl acetate	C_19_H_34_O_4_	6.177	326.5	[M+H]+	1.54	327.2535	2.71E-06	1.1025	POS
32	Glechomafuran	C_15_H_20_O_3_	6.252	248.32	[M+H]+	2.08	249.1490	2.18E-09	1.0662	POS
33	4-Methoxy-6-pentyl-2-prop-1-en-2-yl-2,3-dihydro-1-benzofuran-7-carboxylic acid	C_18_H_24_O_4_	6.656	304.4	[M+H]+	2.23	305.1754	1.33E-18	1.3762	POS
34	Vinpocetine	C_22_H_26_N_2_O_2_	6.685	350.5	[M+H]+	4.89	351.2084	0.000142	1.1893	POS
35	Myristoylcarnitine	C_21_H_41_NO_4_	6.688	371.6	[M+H]+	0.54	372.3110	0.000671	1.0901	POS
36	Sodium Houttuyfonate	C12H23NaO5S	7.216	302.36	[M+NH4]+	0.19	320.1497	0.003393695	1.1872	POS
37	Tryptamine	C_10_H_12_N_2_	4.412	160.22	[M-H]-	0.25	159.0917	4.66E-13	1.0182	NEG
38	6-Pentyl-2H-pyran-2-one	C_10_H_14_O_2_	3.588	166.22	[M-H2O-H]-	0.47	147.0805	8.51E-15	1.0893	NEG
39	Cyclomethyltryptophan	C_12_H_12_N_2_O_2_	3.425	216.24	[M-H]-	1.21	215.0818	4.82E-22	1.4431	NEG
40	Apigenin	C_15_H_10_O_5_	4.832	270.24	[M-H]-	2.94	269.0452	2.23E-10	1.0738	NEG
41	4,6-Dihydroxy-4-(hydroxymethyl)-3,4a,8,8-tetramethyl-5,6,7,8a-tetrahydronaphthalen-1-one	C_15_H_24_O_4_	5.044	268.35	[M-H]-	0.55	267.1592	1.04E-15	1.1182	NEG
42	(10E,15E)-12,13-dihydroxy-9-oxooctadeca-10,15-dienoic acid	C_18_H_30_O_5_	5.815	326.4	[M-H]-	0.44	325.2011	2.12E-11	1.1102	NEG
43	Astragalin	C_21_H_20_O_11_	4.139	448.4	[M-H]-	2.09	447.0931	4.57E-15	1.1085	NEG
44	2-(3,4-dihydroxyphenyl)-3,5-dihydroxy-8-methoxy-7-[(2S,3R,4S,5S,6R)-3,4,5-trihydroxy-6-(hydroxymethyl)oxan-2-yl]oxychromen-4-one	C_22_H_22_O_13_	5.829	494.4	[M-H]-	0.09	493.0977	1.40E-16	1.0677	NEG
45	Methyl 11-methoxy-19-methyl-16,17-didehydro-18-oxayohimban-16-carboxylate	C_22_H_26_N_2_O_4_	8.357	382.5	[M-H]-	1.96	381.1816	3.25E-11	1.1923	NEG
46	Luteolin 7-(6’’-malonylglucoside)	C_24_H_22_O_14_	4.643	534.4	[M-H]-	0.38	533.0928	1.35E-02	1.1197	NEG
47	Isoschaftoside	C_26_H_28_O_14_	3.804	564.5	[M-H]-	3.18	563.1413	0.000370	1.0224	NEG
48	Kaempferol-3-O-glucorhamnoside	C_27_H_30_O_15_	4.358	594.5	[M-H]-	0.37	593.1503	2.62E-04	1.1127	NEG
49	Kaempferol 7-neohesperidoside	C_27_H_30_O_15_	4.142	594.5	[M+Cl]-	2.62	629.1290	2.95E-27	1.1101	NEG
50	Rutin	C_27_H_30_O_16_	5.673	610.5	[M-H]-	2.76	609.1467	0.003493	1.1411	NEG
51	Rhamnetin 3-sophoroside	C_28_H_32_O_17_	4.659	640.5	[M-H]-	0.19	639.1557	1.41E-08	1.2257	NEG
52	cis-Aconitic acid	C_6_H_6_O_6_	2.565	174.11	[M-H]-	-3.01	173.0075	1.14E-27	1.0474	NEG
53	Pyridoxal	C_8_H_9_NO_3_	3.296	167.16	[M-H]-	2.18	166.0502	0.000034	1.1788	NEG
54	2-(4-Hydroxyphenyl)propanoic acid	C_9_H_10_O_3_	3.76	166.17	[M-H]-	3.00	165.0551	8.33E-13	1.0797	NEG

**Figure 3:**
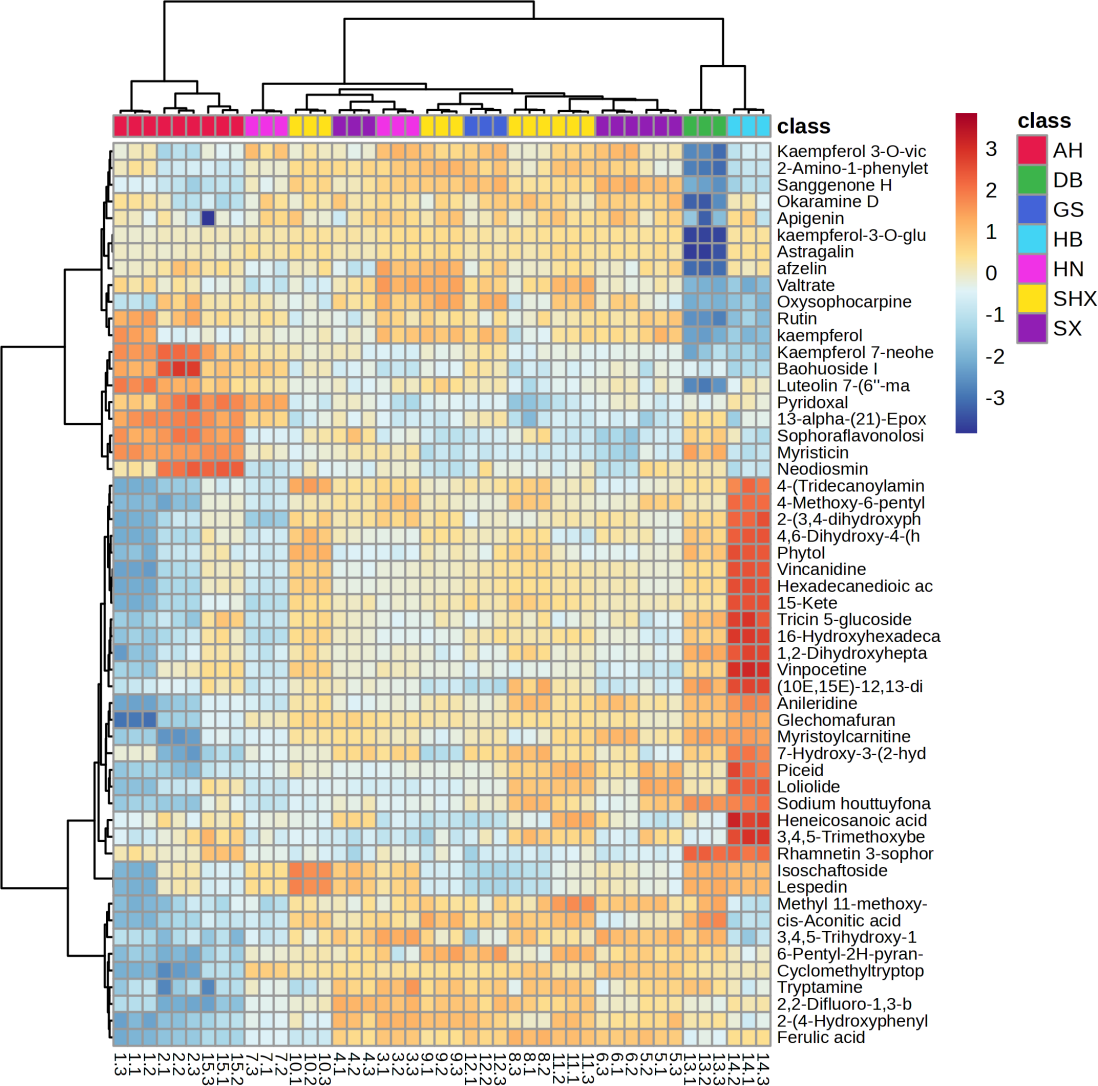
Heatmap of the 54 differential metabolites from fifteen batches of TCT samples.

Heatmap of the 54 differential metabolites from seven different regions’ TCT samples were shown in **Figure 3**. The heatmap revealed a consistent trend in the variation of differential metabolites in the Shaanxi, Shanxi, Gansu, and Henan regions. In contrast, the differential metabolites in the Jilin, Hubei, and Anhui regions deviated previous from the aforementioned four regions. Moreover, the Jilin and Hubei regions exhibited similar trends in differential metabolite changes, with the most significant differences observed in comparison to the Anhui region. These trends showed a clear correlation with geographical locations.

### 3.4 Differential metabolic pathway analysis(KEGG)

**Figure 4:**
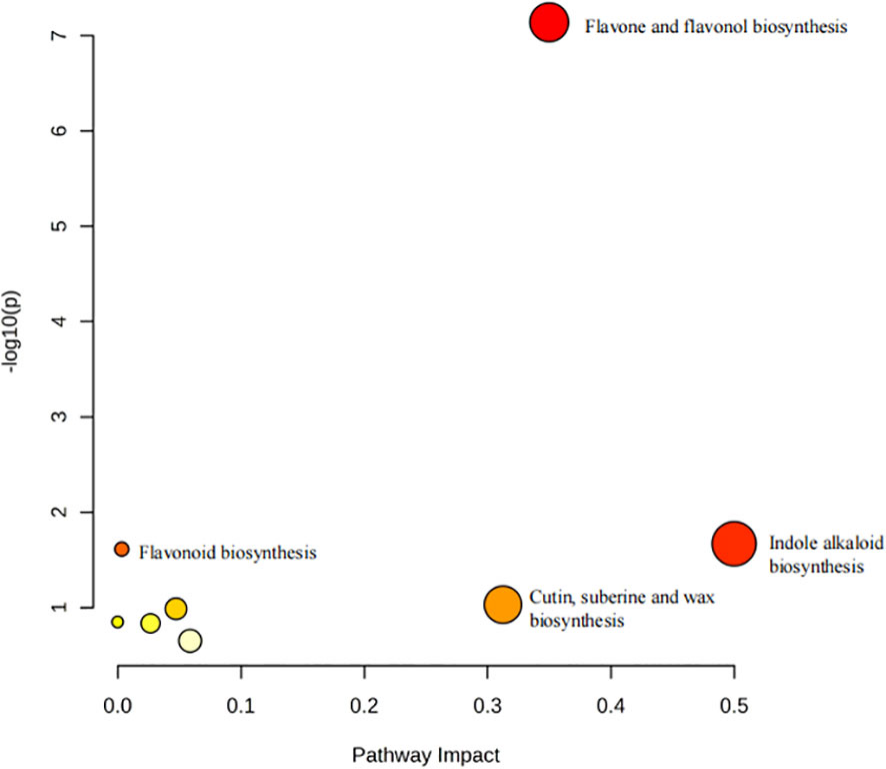
Enriched metabolic pathways of different TCT samples from various origins.

The annotation of metabolites was performed, and data were imported into MetaboAnalyst 5.0 for KEGG metabolic pathway enrichment analysis. Enriched metabolic pathways were visualized using a bubble plot, as shown in **Figure 4**. The metabolic pathways include flavone and flavonol biosynthesis, indole alkaloid biosynthesis, flavonoid biosynthesis, and more. From the graph, it can be observed that the biosynthesis pathways of flavonoids and flavonols in TCT samples from seven different regions exhibited the greatest differences, the highest enrichment levels, and the most significant variations. Consequently, we speculate that the differences in metabolite contents among these TCT samples are likely the result of regulatory mechanisms in the biosynthetic pathways of flavones and flavonols, thereby potentially influencing the bioactivity of TCT from different geographical origins.

### 3.5 Regional variation in antioxidant and anti-inflammatory activities of TCT correlates with TFC

**Figure 5:**
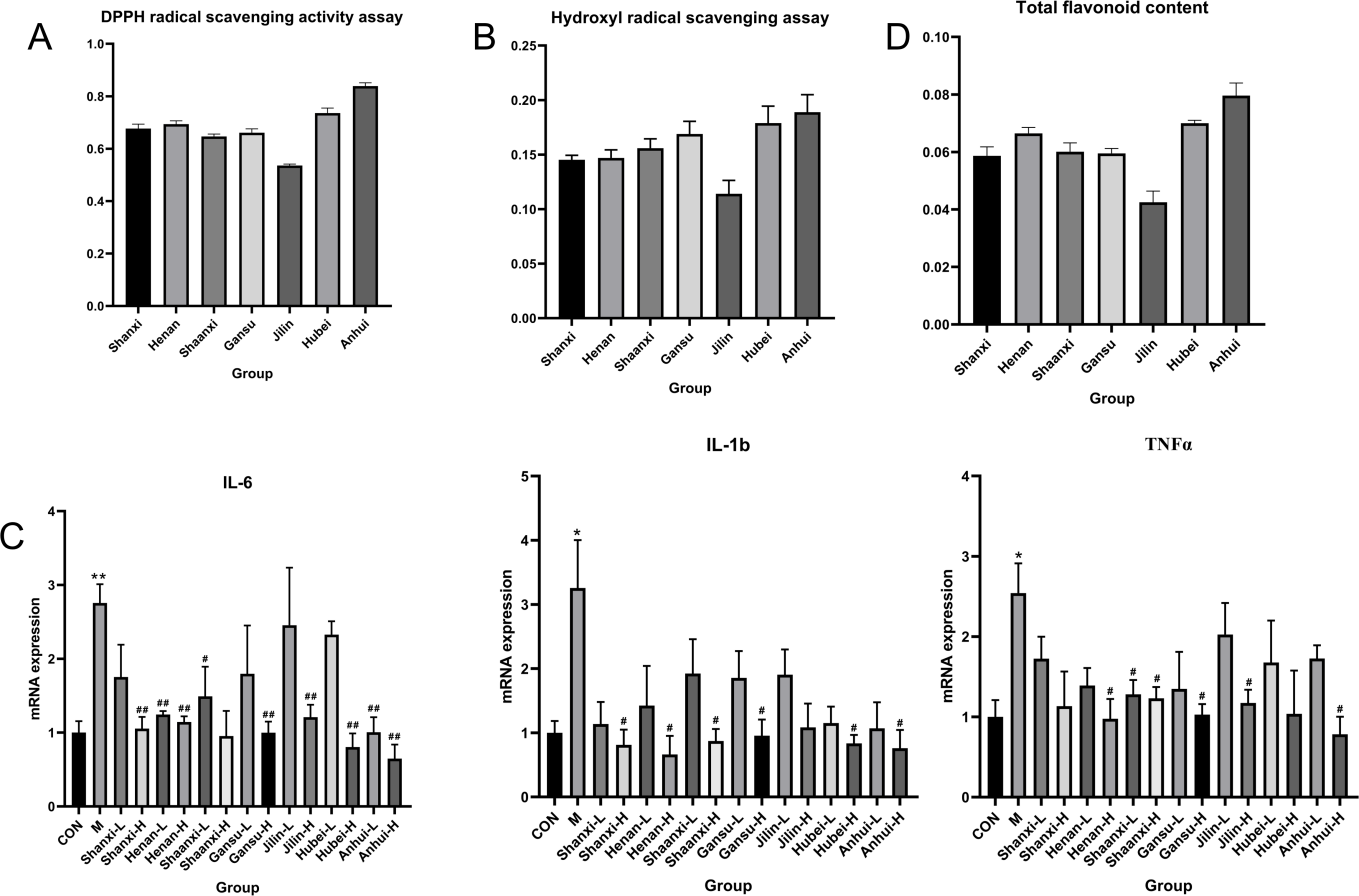
Regional variation in antioxidant and anti-inflammatory activities of TCT correlates with TFC. **(A)** DPPH radical scavenging activity of TCT extracts from different regions. **(B)** Hydroxyl radical scavenging activity of TCT extracts from different regions. **(C)** The expression levels of mRNA for inflammatory markers (IL-6, IL-1β, and TNF-α) in LPS-induced inflammatory response assays. *p < 0.05, **p < 0.01 M *vs*. CON; #p < 0.05, ##p < 0.01 different treatment group *vs*. M; **(D)** TFC of TCT extracts from different regions.

The antioxidant activity of TCT from different regions was evaluated using DPPH and Hydroxyl radical scavenging assays. The results, as shown in **Figures 5A, B**, indicate that TCT from Shanxi, Henan, Shaanxi, and Gansu provinces exhibited similar free radical scavenging rates. In contrast, TCT from Jilin province demonstrated the lowest free radical scavenging rate, while TCT from Anhui province showed the highest rate. These findings suggest that the antioxidant properties of TCT differ considerably based on its geographical origin.

In the LPS-induced inflammatory response assay, the anti-inflammatory activity of TCT was assessed by measuring the levels of three inflammatory markers: IL-6, IL-1β, and TNF-α (**Figure 5C**). Consistent with the antioxidant activity results, TCT from Anhui province exhibited the highest anti-inflammatory activity, while TCT from Jilin province showed the lowest. The anti-inflammatory activities of TCT from Shanxi, Henan, Shaanxi, and Gansu provinces were similar.

To further elucidate the observed differences in antioxidant and anti-inflammatory activities, the TFC of TCT from seven regions was measured. The results revealed that TCT from Anhui province had the highest TFC, whereas TCT from Jilin province had the lowest. The TFC of TCT from Shanxi, Henan, Shaanxi, and Gansu provinces were similar (**Figure 5D**). These findings are consistent with the metabolomic clustering analysis, indicating that the primary contributors to the antioxidant and anti-inflammatory activities of TCT are the total flavonoid components.

### 3.6 Characterization and quantification of key flavonoids in TCT from different regions

**Figure 6:**
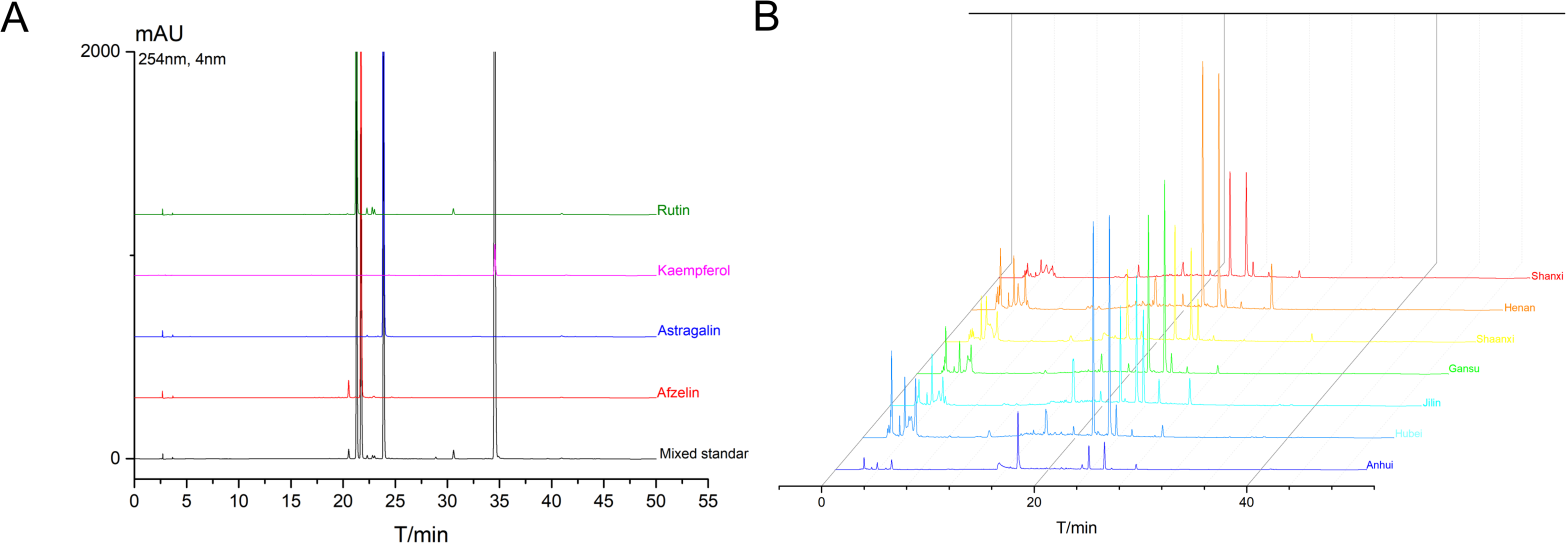
HPLC analysis of characteristic chemical components in TCT. **(A)** HPLC chromatograms of the four major components (Afzelin, Kaempferol, Astragalin, and Rutin) in TCT. **(B)** Chromatographic profiles of TCT samples from different regions.

Afzelin, Kaempferol, and Astragalin are characteristic chemical components in TCT and serve as differential metabolites among the various regions. The levels of Afzelin, Kaempferol, Astragalin, and Rutin in TCT from several regions were further quantified. The analysis of these four major components is presented in **Figure 6A**, which demonstrates the absence of interfering peaks, thereby confirming the high specificity of the High-performance liquid chromatography (HPLC) method employed in this study. The calibration curve for Afzelin, Kaempferol, Astragalin, and Rutin are shown in **Table 4**, indicating good linearity within the range of 1.6-1000 μg/mL.

**Table 4:** Linear equations, linear ranges, and correlation coefficients of four components.

Ingredient	Regression equation	Linearity range (μg/mL)	R^2^
Rutin	y=18902301.1x-15820	1.6-1000	0.9993
Afzelin	y=14042648.9x-27152	1.6-1000	0.9995
Astragalin	y=30000000x-162226	1.6-1000	0.9995
Kaempferol	y=1862151x-84068.2	1.6-1000	0.9998

The chromatographic profiles of TCT from different regions are depicted in **Figure 6B**. Based on the HPLC peak areas, the contents of these four chemical components in TCT from various regions were calculated and are presented in **Table 5**. Among these, Afzelin was found to be the most abundant, followed by Astragalin, with Kaempferol being the least abundant. Specifically, TCT from Anhui province exhibited the highest levels of Astragalin and Rutin, at 3.876 mg/g and 0.362 mg/g, respectively, but the lowest level of Kaempferol, at 0.0561 mg/g. Conversely, TCT from Jilin province had the lowest levels of all components, consistent with the total flavonoid content measurements. These findings underscore the regional variations in the chemical composition of TCT, which are in line with the observed differences in total flavonoid content and the associated antioxidant and anti-inflammatory activities.

**Table 5:** Contents of four components in TCT from seven origins.

Regions	Rutin(mg/g)	Afzelin(mg/g)	Astragalin(mg/g)	Kaempferol(mg/g)
Anhui	0.362	8.892	3.876	0.0561
Jilin	0.127	0.152	0.992	0.1380
Hubei	0.335	15.053	1.648	0.2566
Shaanxi	0.353	9.104	1.564	0.2475
Shanxi	0.356	15.493	0.761	0.0838
Henan	0.266	6.821	1.083	0.0633
Gansu	0.168	10.114	0.922	0.1612

### 3.7 Target prediction and enrichment analysis of characteristic chemical components in TCT

**Figure 7:**
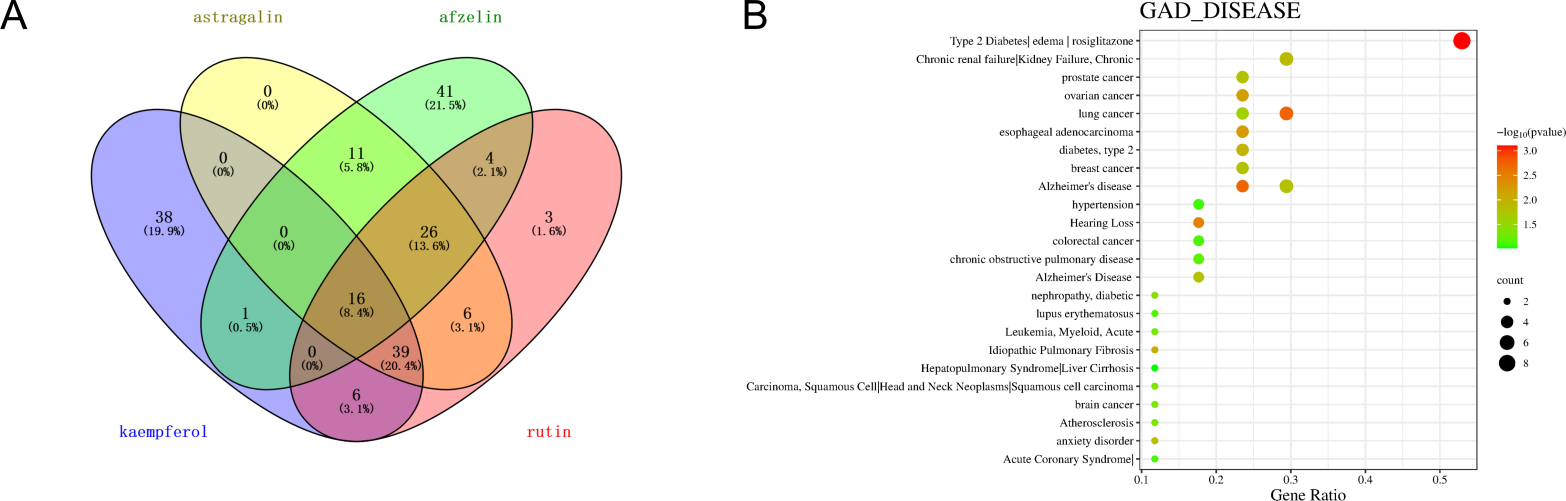
Overlapping targets of characteristic chemical components in TCT and their enrichment analysis. **(A)** Venn diagram illustrating the overlapping targets of four characteristic chemical components (Afzelin, Kaempferol, Rutin, and Astragalin) in TCT. The numbers within the overlapping regions indicate the shared targets among the components. **(B)** Enrichment analysis of the overlapping targets using the DAVID Knowledgebase platform. The size of the bubbles represents the count of genes, and the color gradient indicates the significance level (p-value).

Target prediction for the characteristic chemical components in TCT, namely Afzelin, Kaempferol, Rutin, and Astragalin, was further conducted. Using the SwissTargetPrediction platform, 100 targets were predicted for each component (**Supplementary File 5**). The intersection of these targets resulted in 16 common targets, as shown in **Figure 7A**. Subsequently, enrichment analysis of these overlapping targets was performed using the DAVID Knowledgebase platform (**Figure 7B**). It was revealed by the analysis that the targets are predominantly associated with pulmonary diseases, which aligns with the therapeutic indications of TCT. The enriched diseases are listed in **Supplementary File 6**.

## 4 Discussion

Our metabolomics approach has been demonstrated as a robust and reliable method for intraspecies discrimination and quality assessment of TCT samples from various geographical regions. The selection of 54 differential metabolites can be utilized for quality assessment of TCT. Our ultimate goal is to explore the differences in the compositional elements and biological activities of TCT from different geographical origins, providing a theoretical basis and reference for the scientific application of TCT in medicine.

A comparative analysis of TCT samples from seven provinces-Anhui, Jilin, Hubei, Shanxi, Shaanxi, Henan, and Gansu-was conducted in this study. Non-targeted metabolomics analysis revealed a strong correlation between the chemical composition of TCT from these regions and their geographical distribution. Concurrently, our *in vitro* assays for antioxidant and anti-inflammatory activities, along with the determination of total flavonoid content, further validated the accuracy of our non-targeted metabolomics approach. Through the map of China, we can learn that the TCT sampling locations in Henan, Shaanxi, Shanxi, and Gansu are all within a 200-kilometer radius circle, resulting in minimal differences in metabolite profiles. This phenomenon may be attributed to the similarity in climate, temperature, humidity, and other growth conditions in these closely situated areas. In contrast, TCT samples from Anhui, Hubei, and Jilin regions, which are more distant, exhibited larger differences in metabolites.

Analysis of metabolic pathways associated with differential metabolites indicated that the primary variations among TCT samples from the seven provinces occurred in the biosynthetic pathways of flavonoids and flavonols. Flavone and flavonol biosynthesis are crucial branches of the larger flavonoid biosynthetic pathway in higher plants. These pathways contribute significantly to plant development, defense, and secondary metabolism, including pigmentation, UV protection, pathogen resistance, and communication with symbiotic organisms. Flavone biosynthesis is primarily catalyzed by flavone synthase, which converts flavanones into flavones by forming a double bond between the C-2 and C-3 positions of flavanones’ ring C (Jiang et al., 2016). Flavonol synthesis involves the hydroxylation of dihydroflavonols at the C-3 position of ring C, primarily catalyzed by flavonol synthase, a key and rate-limiting enzyme in the pathway (Lei et al., 2023). Similar to flavone synthase, flavonol synthase is also an Fe²+/2-oxoglutarate-dependent dioxygenase (Wang et al., 2022). Dihydrokaempferol is converted to kaempferol, dihydroquercetin to quercetin, and dihydromyricetin to myricetin (Liu et al., 2021). Conversion of kaempferol to quercetin and myricetin is facilitated by flavanone 3′-hydroxylase (F3′H) and flavanone 3′,5′-hydroxylase (F3′5′H) (Jaegle et al., 2016). Further modification of both flavones and flavonols includes glycosylation, methylation, and acylation, which enhance their solubility, stability, and biological functions (Jiang et al., 2023). These modifications result in a broad diversity of flavonoid compounds with various structural and functional roles in plants (Tariq et al., 2023).

The quantification results of Rutin, Kaempferol, Afzelin, Astragalin, and TFC revealed that TCT from Anhui province had the highest levels of these characteristic chemical components, corresponding to the highest antioxidant and anti-inflammatory activities. Conversely, TCT from Jilin province exhibited the lowest levels of these components and the lowest antioxidant and anti-inflammatory activities. These findings suggest that TCT possesses significant antioxidant and anti-inflammatory activities, which are strongly correlated with TFC. Furthermore, TFC is highly dependent on the geographical region. Therefore, we hypothesize that the bioactivity and quality of TCT are closely related to the region and climate, with central regions being more conducive to the accumulation of flavonoid compounds.

The geographical locations and climatic conditions of the seven provinces were investigated, and it was found that the primary differences were related to variations in latitude, which were accompanied by changes in temperature, light intensity, duration of sunlight, and rainfall. These environmental factors significantly influence the accumulation of flavonoid compounds in plants. Lower latitude regions typically experience higher temperatures and stronger ultraviolet (UV) radiation, leading to higher accumulation of flavonoid compounds in plants. This is because flavonoids possess antioxidant properties and UV-protective functions, which help plants cope with the environmental stresses of high temperatures and intense UV radiation. Overall, the biosynthesis and accumulation of secondary metabolites are modulated by plants to optimize their survival in response to complex environmental conditions or abiotic stress (Ouerghemmi et al., 2016; Ribeiro et al., 2020). Our results indicate that the ecological and geographical factors of different regions may significantly impact the composition of flavonoid compounds and the *in vitro* antioxidant capacity of TCT.

However, this study has several limitations. Firstly, the experiment only selected TCT samples from central and northern regions of China, excluding samples from southern regions. Secondly, the study focused solely on the flavonoid components of TCT, neglecting the potential roles of other active constituents such as alkaloids and polysaccharides. Future research should investigate these other components to discover additional novel compounds.

## 5 Conclusion

In conclusion, our metabolomics study reveals significant variations in TCT across seven geographical regions in China. Through UPLC-Q-TOF-MS, we identified 54 differential metabolites and highlighted key variations in flavonoid and flavanol biosynthetic pathways that correlate with TCT’s bioactivity. Notably, TCT from Anhui province exhibited the highest total flavonoid content and strongest antioxidant and anti-inflammatory activities, while samples from Jilin province showed the lowest. These results underscore the importance of geographical origin in influencing TCT quality and efficacy, offering a framework for quality assessment and source tracking in traditional Chinese medicine. This work illustrates the potential of metabolomics to enhance the understanding of herbal medicine.

## Data Availability

The original contributions presented in the study are included in the article/**Supplementary Material**; further inquiries can be directed to the corresponding authors.

## Supplementary material

Supplementary File 1 The result of CCK-8 cell viability assay.
