# Supplementary material for: Geographical variation in metabolite profiles and bioactivity of Thesium chinense Turcz. revealed by UPLC-Q-TOF-MS-based metabolomics
Source: Front Plant Sci. 2025 Jan 10;15:1471729. doi: 10.3389/fpls.2024.1471729 (PMC11760594; doi:10.3389/fpls.2024.1471729)
Supplement: Supplementary file 2 [file SupplementaryFile3.pdf]

Supplementary File 3: The graphs of all batch sample TIC.

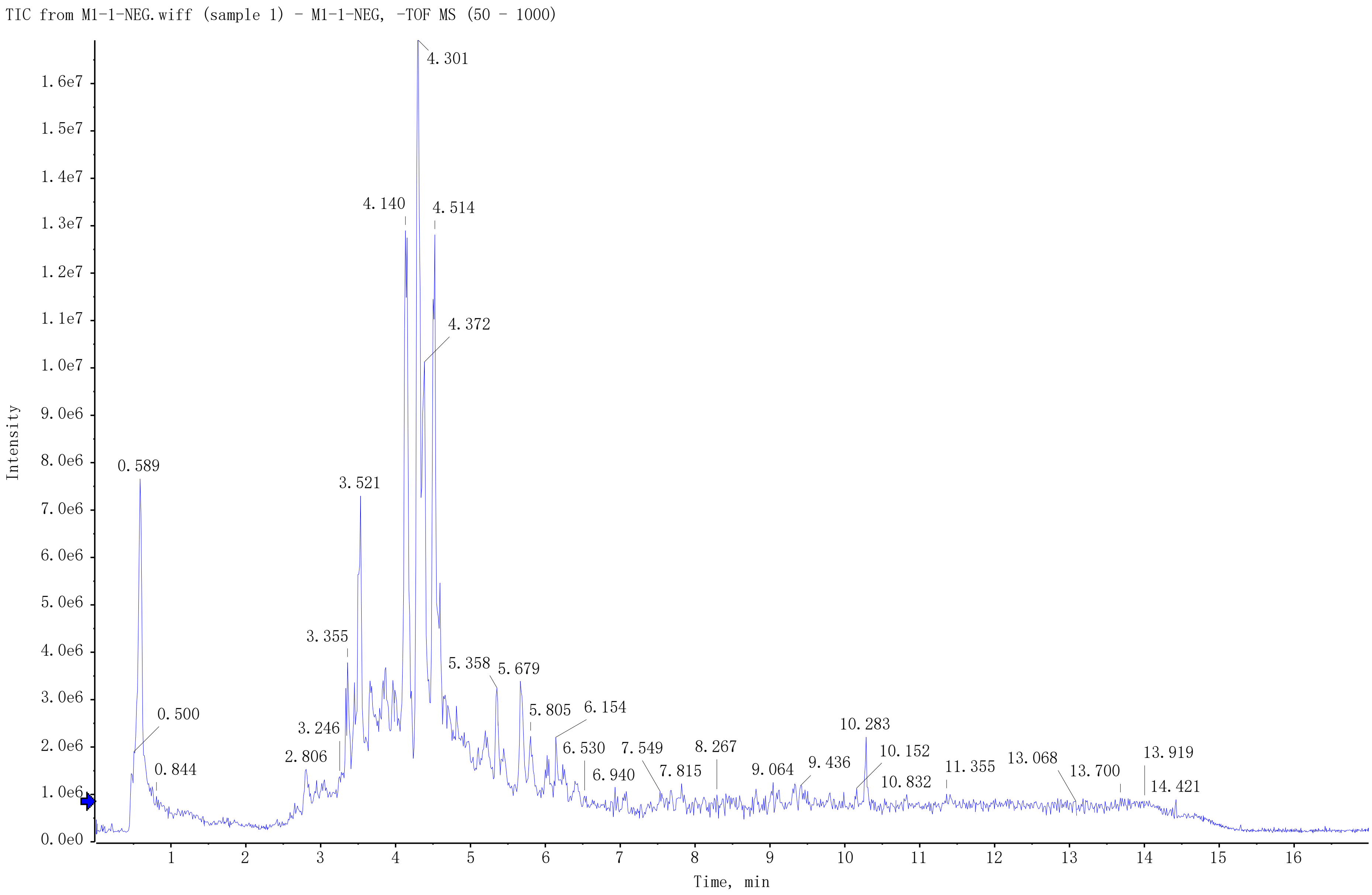

TIC from M1-2-NEG.wiff (sample 1) - M1-2-NEG, -TOF MS (50 - 1000)

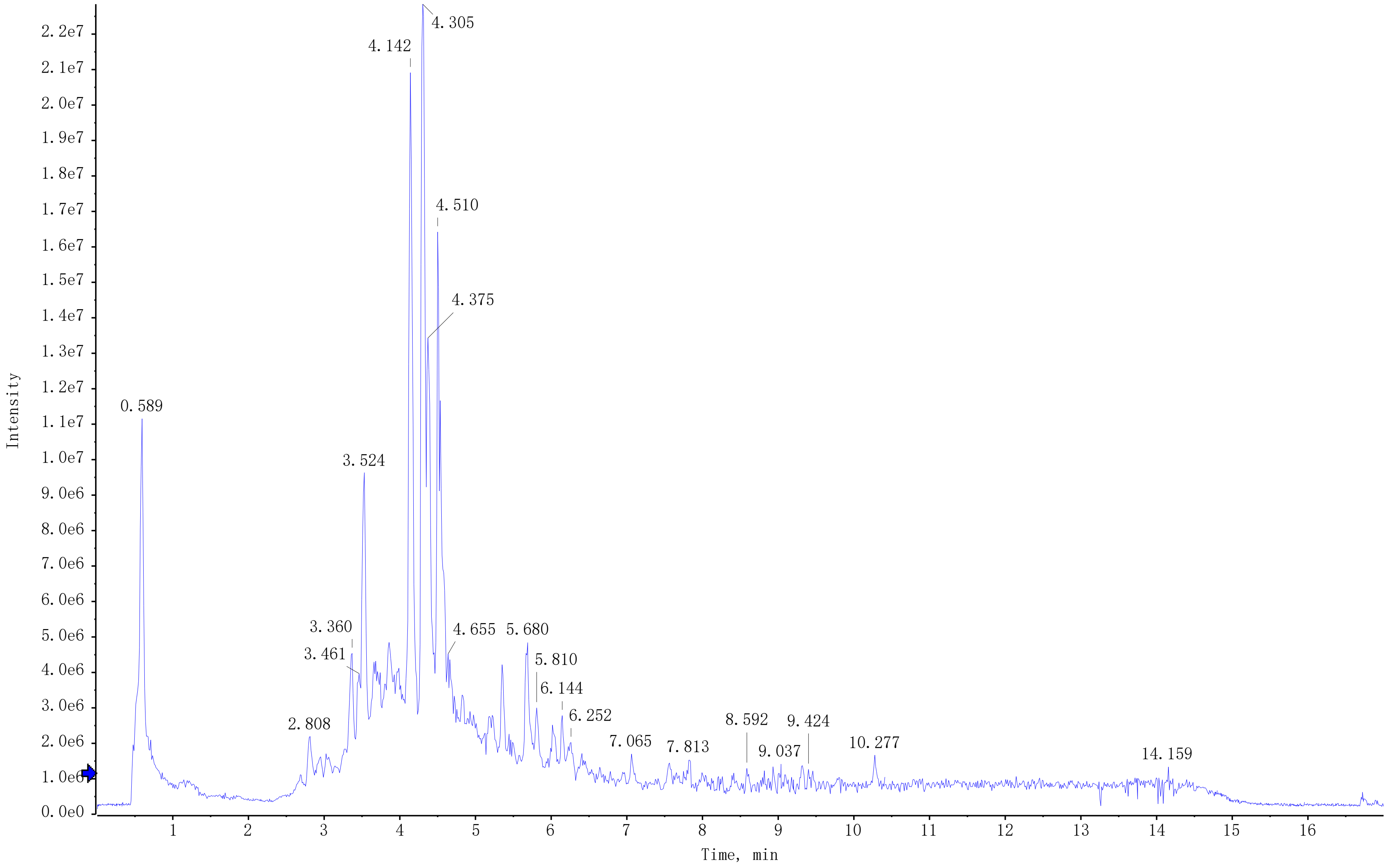

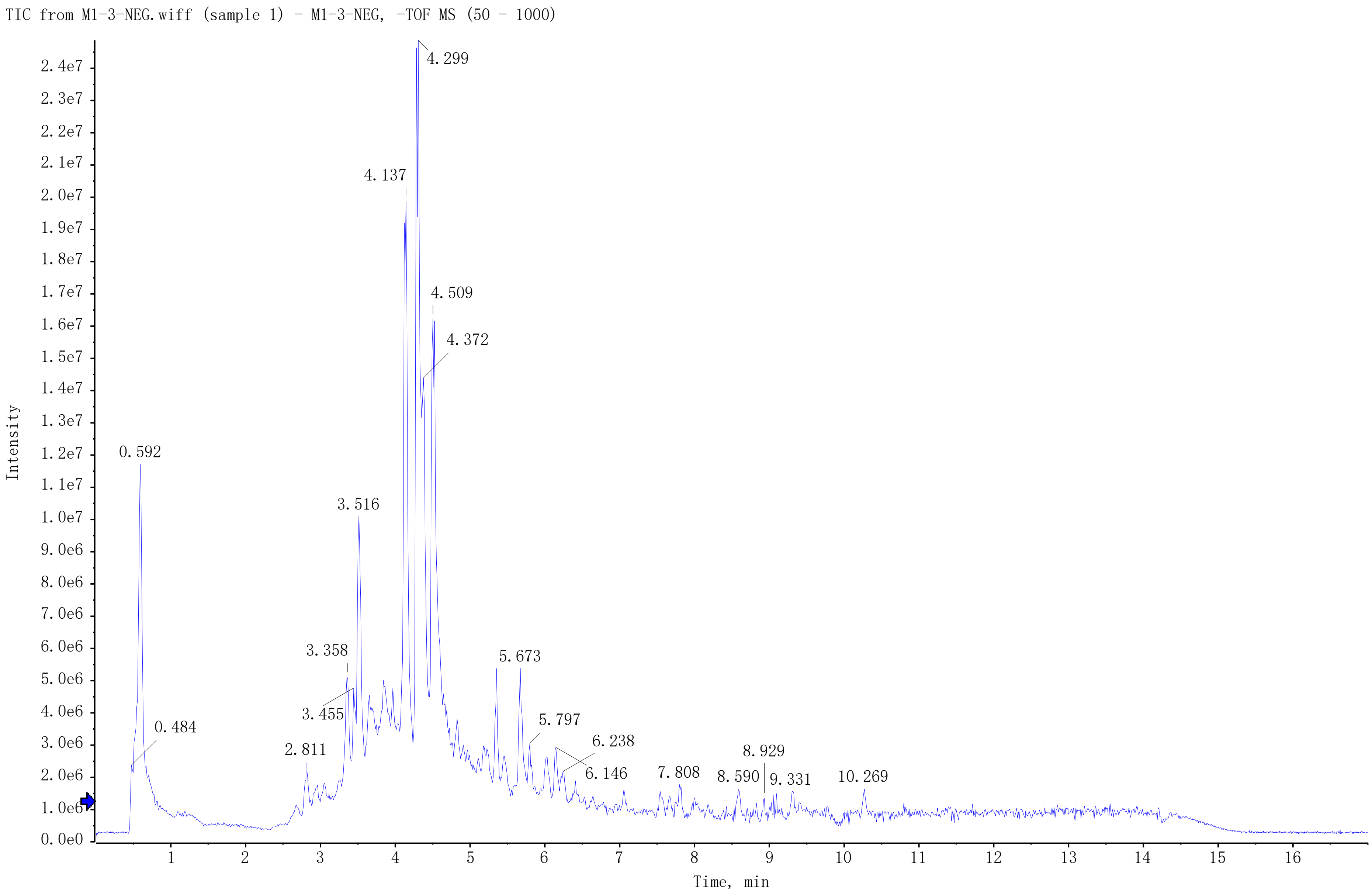
TIC from M1-3-NEG.wiff (sample 1) - M1-3-NEG, -TOF MS (50 - 1000)
Intensity
Time, min
0.592
0.484
2.811
3.358
3.455
3.516
4.137
4.299
4.509
4.372
5.673
5.797
6.238
6.146
7.808
8.590
8.929
9.331
10.269

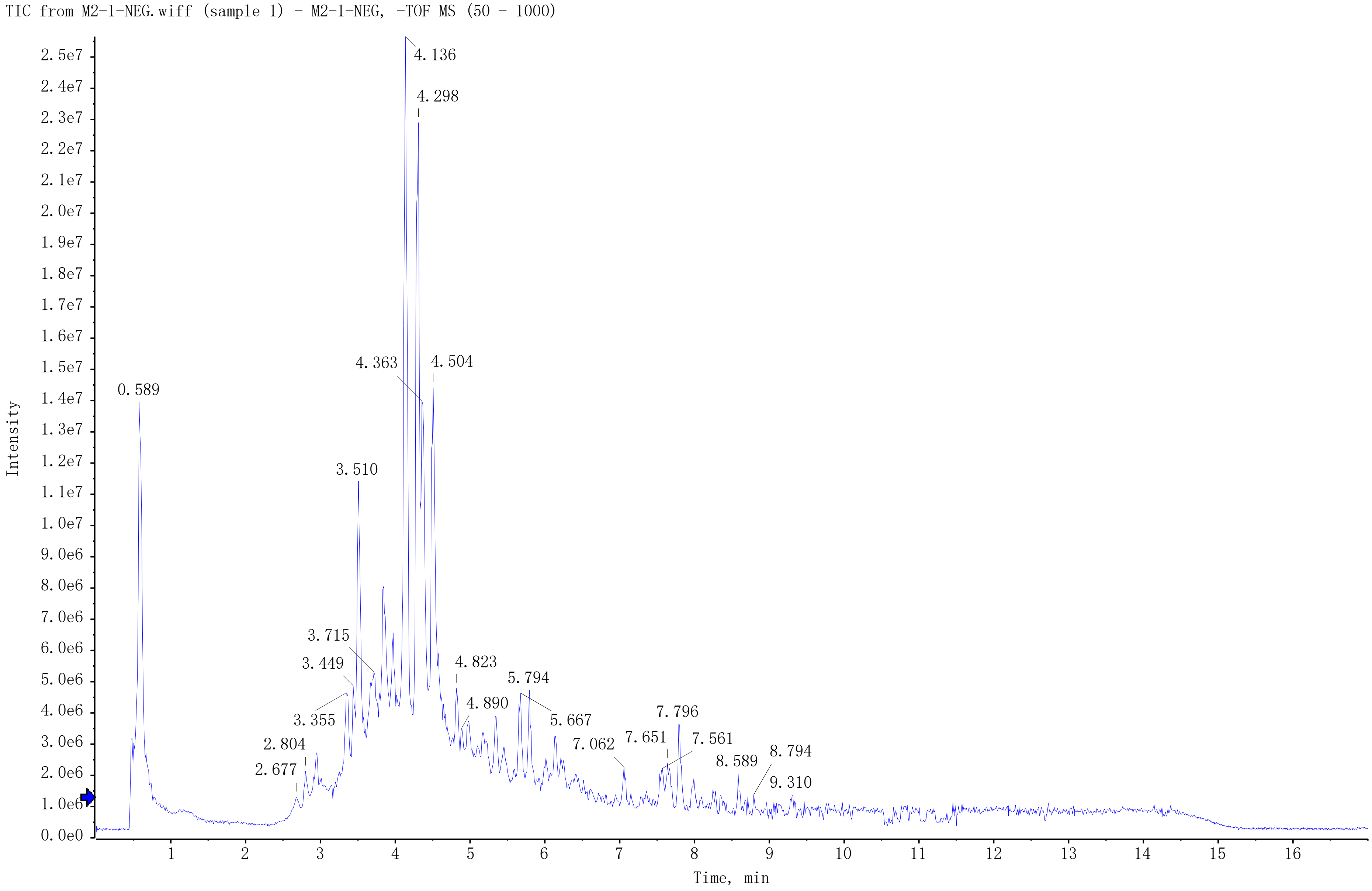
TIC from M2-1-NEG.wiff (sample 1) - M2-1-NEG, -TOF MS (50 - 1000)
Intensity
Time, min
0.589
2.677
2.804
3.355
3.449
3.510
3.715
4.136
4.298
4.363
4.504
4.823
4.890
5.794
5.667
7.062
7.651
7.796
7.561
8.589
8.794
9.310

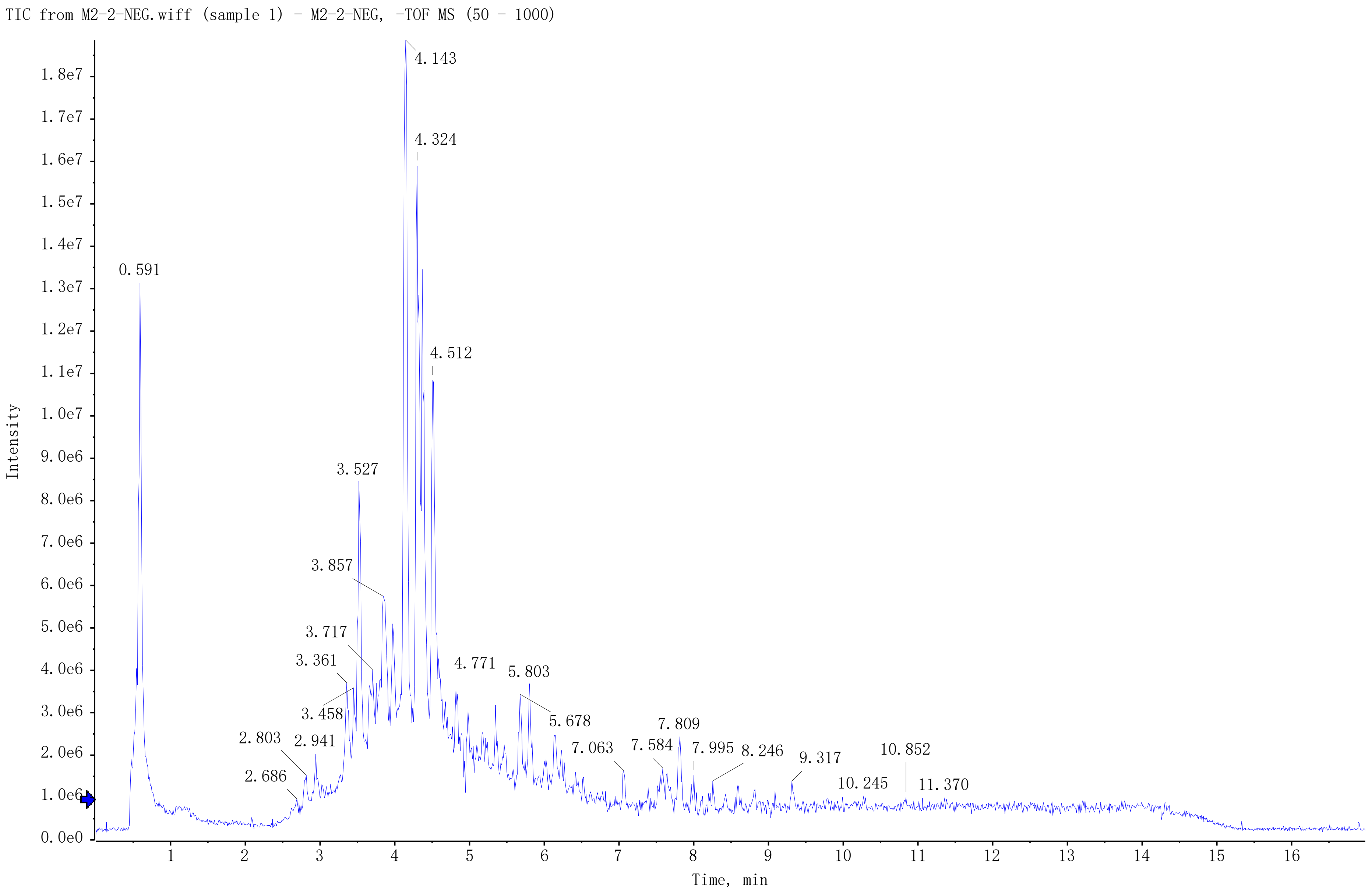
TIC from M2-2-NEG.wiff (sample 1) - M2-2-NEG, -TOF MS (50 - 1000)
Intensity
Time, min
0.591
2.686
2.803
2.941
3.361
3.458
3.527
3.717
3.857
4.143
4.324
4.512
4.771
5.803
5.678
7.063
7.584
7.809
7.995
8.246
9.317
10.245
10.852
11.370

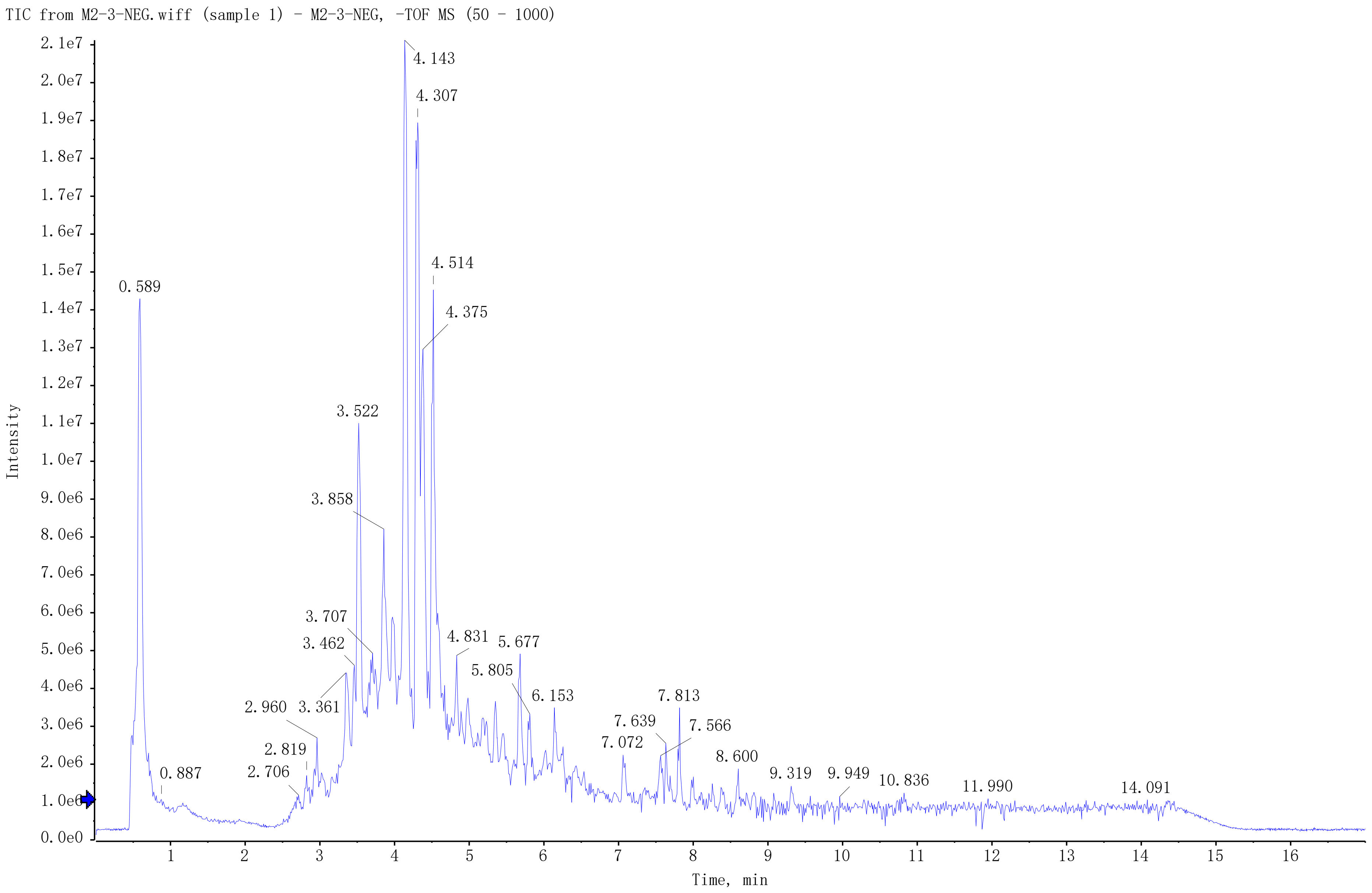

TIC from M2-3-NEG.wiff (sample 1) - M2-3-NEG, -TOF MS (50 - 1000)
Intensity
Time, min
0.589
0.887
2.706
2.819
2.960
3.361
3.462
3.522
3.707
3.858
4.143
4.307
4.375
4.514
4.831
5.677
5.805
6.153
7.072
7.566
7.639
7.813
8.600
9.319
9.949
10.836
11.990
14.091

TIC from M3-1-NEG.wiff (sample 1) - M3-1-NEG, -TOF MS (50 - 1000)

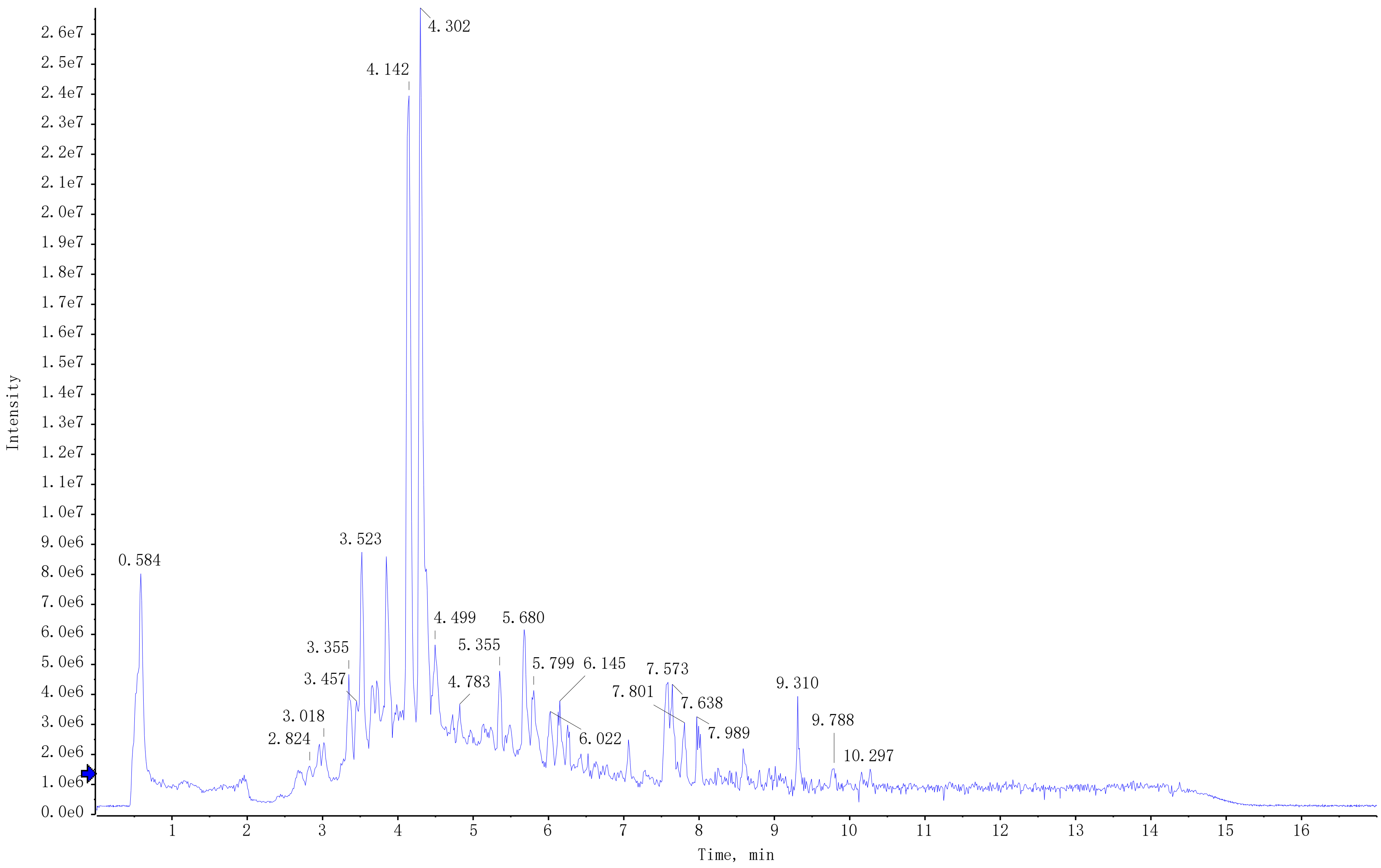

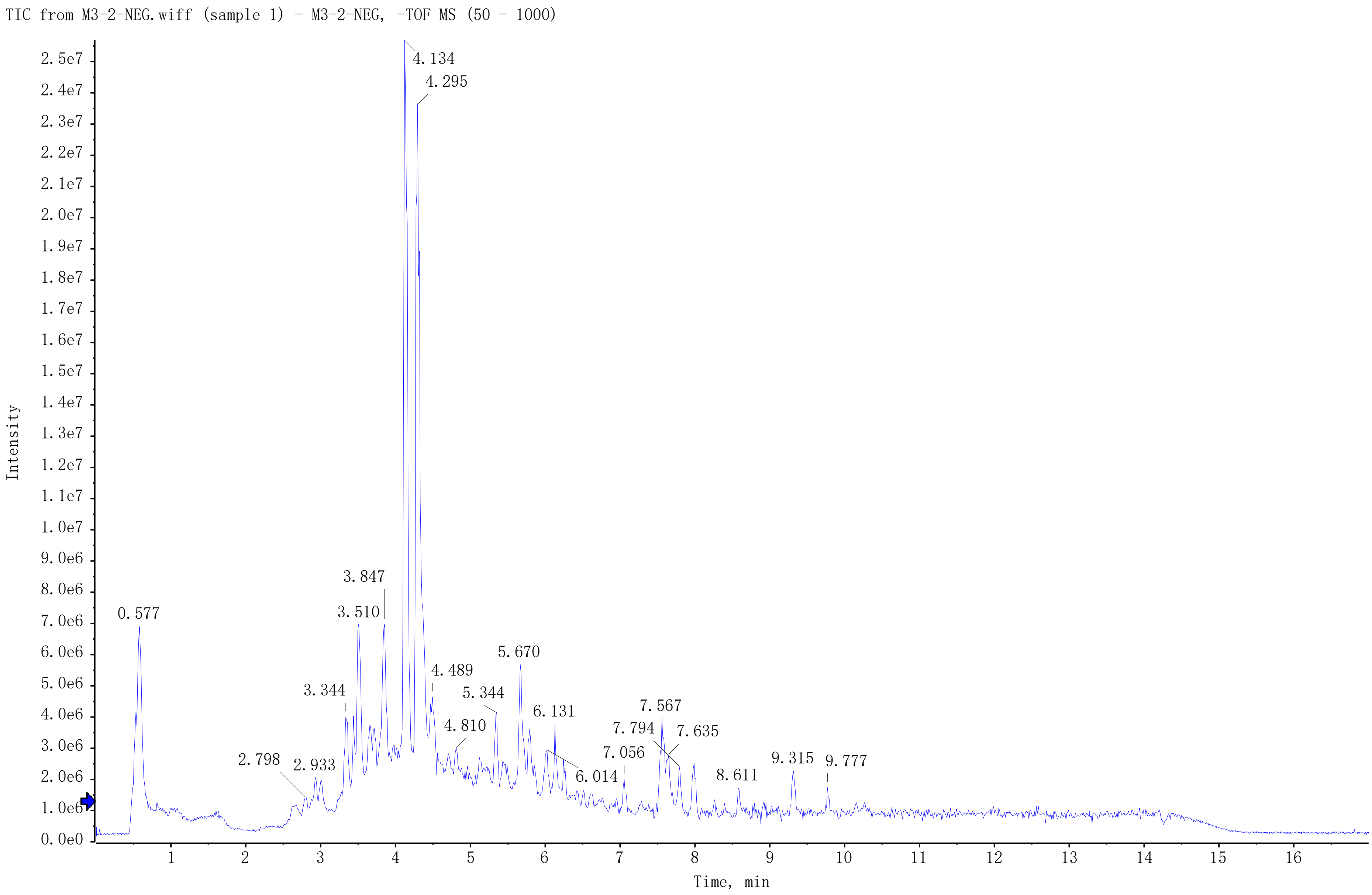

TIC from M3-2-NEG.wiff (sample 1) - M3-2-NEG, -TOF MS (50 - 1000)
Intensity
Time, min
0.577
2.798
2.933
3.344
3.510
3.847
4.134
4.295
4.489
4.810
5.344
5.670
6.131
6.014
7.056
7.794
7.567
7.635
8.611
9.315
9.777

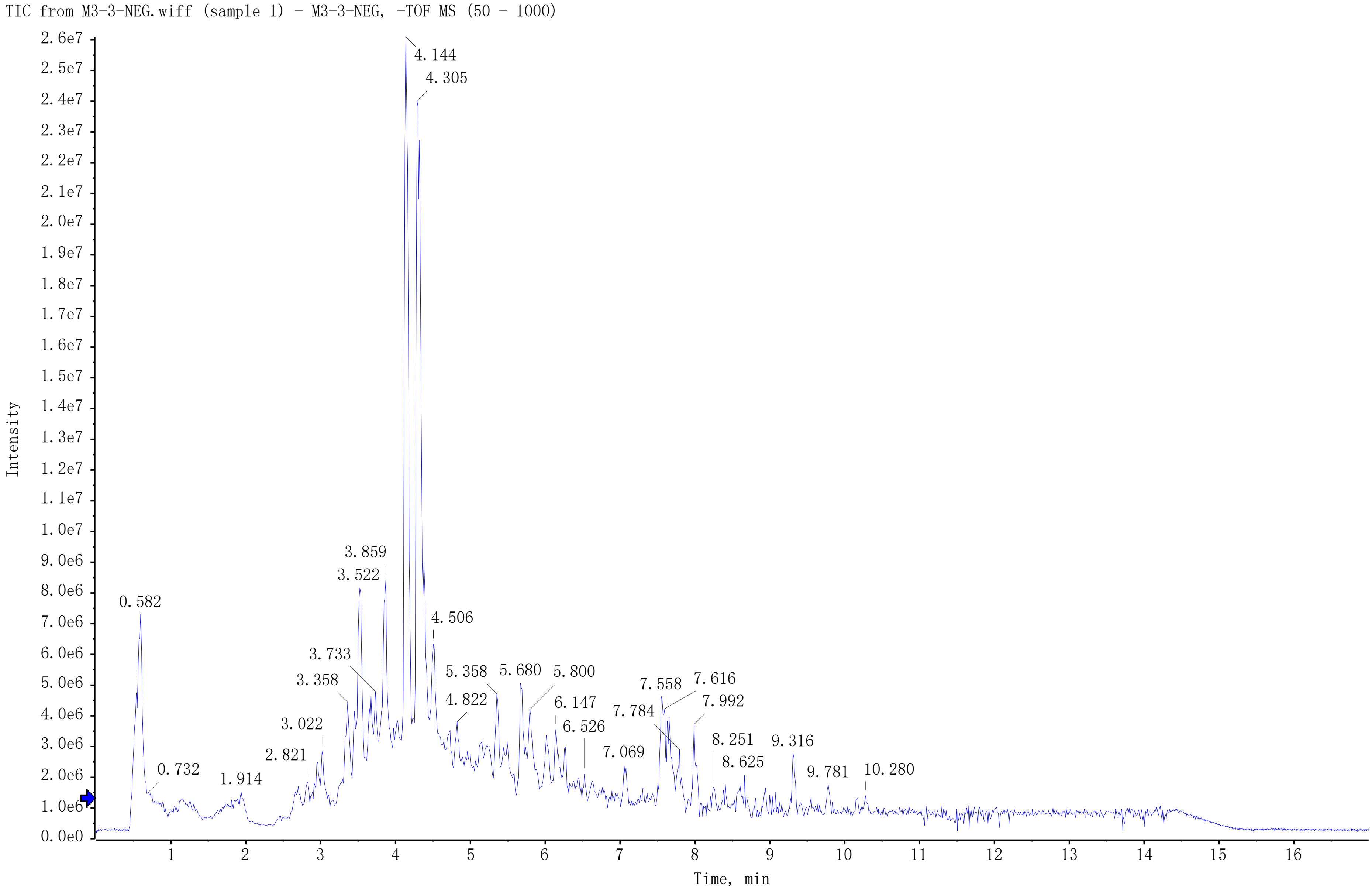
TIC from M3-3-NEG.wiff (sample 1) - M3-3-NEG, -TOF MS (50 - 1000)
Intensity
Time, min
0.582
0.732
1.914
2.821
3.022
3.358
3.522
3.733
3.859
4.144
4.305
4.506
4.822
5.358
5.680
5.800
6.147
6.526
7.069
7.558
7.616
7.784
7.992
8.251
8.625
9.316
9.781
10.280

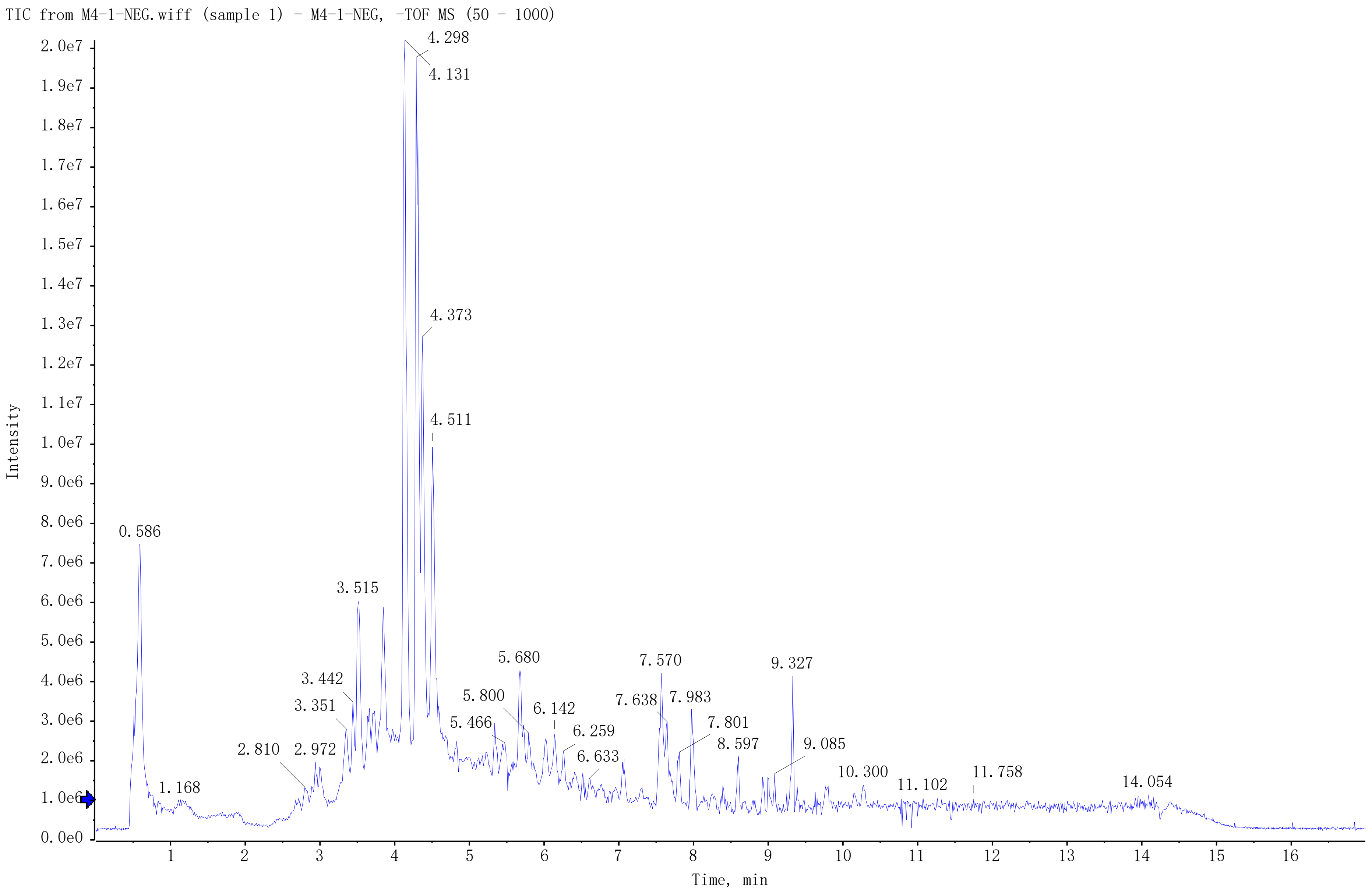

TIC from M4-1-NEG.wiff (sample 1) - M4-1-NEG, -TOF MS (50 - 1000)
Intensity
Time, min
2.0e7
1.9e7
1.8e7
1.7e7
1.6e7
1.5e7
1.4e7
1.3e7
1.2e7
1.1e7
1.0e7
9.0e6
8.0e6
7.0e6
6.0e6
5.0e6
4.0e6
3.0e6
2.0e6
1.0e6
0.0e0
1
2
3
4
5
6
7
8
9
10
11
12
13
14
15
16
4.298
4.131
4.373
4.511
0.586
3.515
3.442
3.351
2.810
2.972
1.168
5.680
5.800
5.466
6.142
6.259
6.633
7.570
7.638
7.983
7.801
8.597
9.327
9.085
10.300
11.102
11.758
14.054

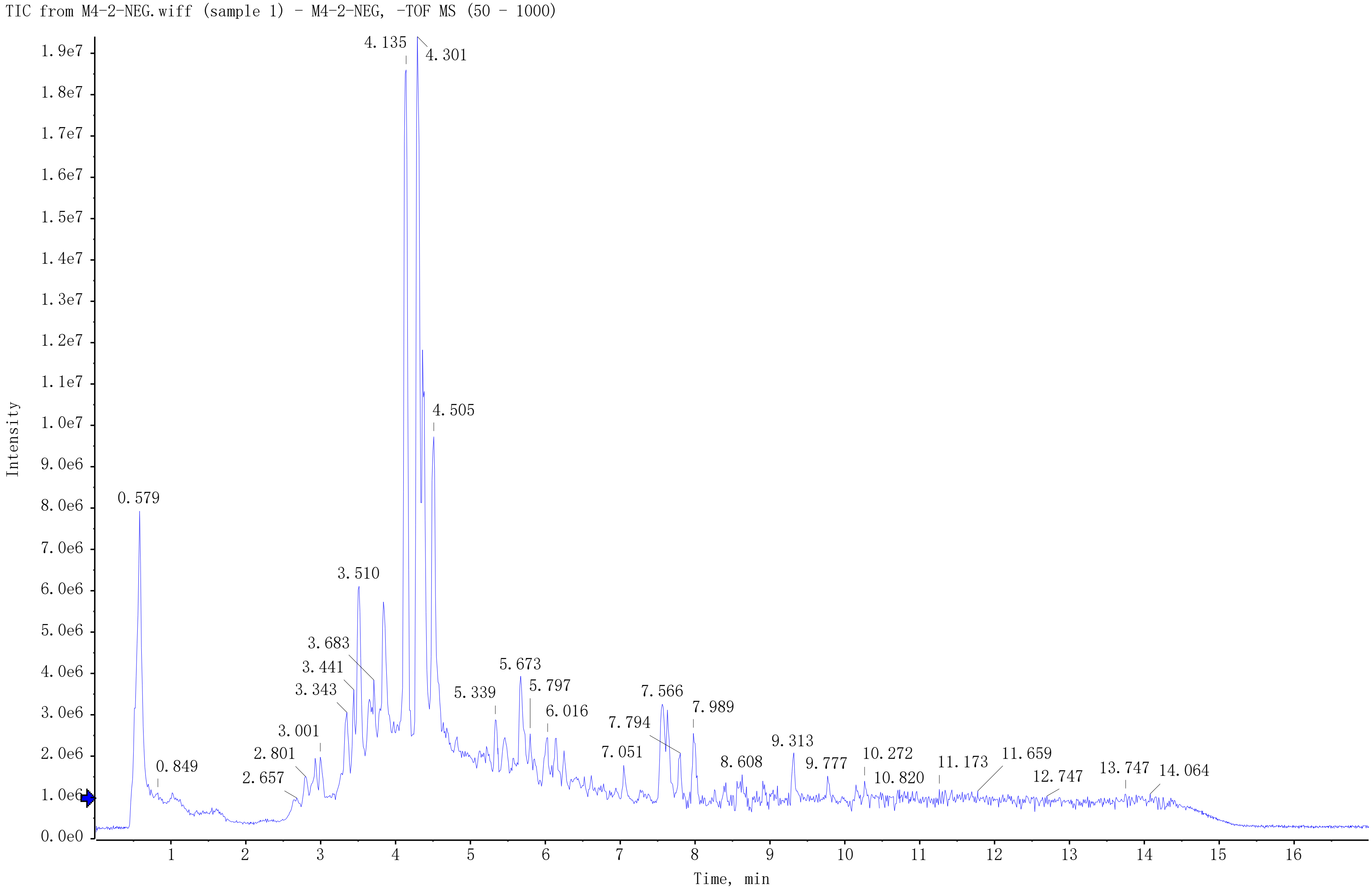
TIC from M4-2-NEG.wiff (sample 1) - M4-2-NEG, -TOF MS (50 - 1000)
Intensity
Time, min
0.579
0.849
2.657
2.801
3.001
3.343
3.441
3.683
3.510
4.135
4.301
4.505
5.339
5.673
5.797
6.016
7.051
7.794
7.566
7.989
8.608
9.313
9.777
10.272
10.820
11.173
11.659
12.747
13.747
14.064

TIC from M4-3-NEG.wiff (sample 1) - M4-3-NEG, -TOF MS (50 - 1000)

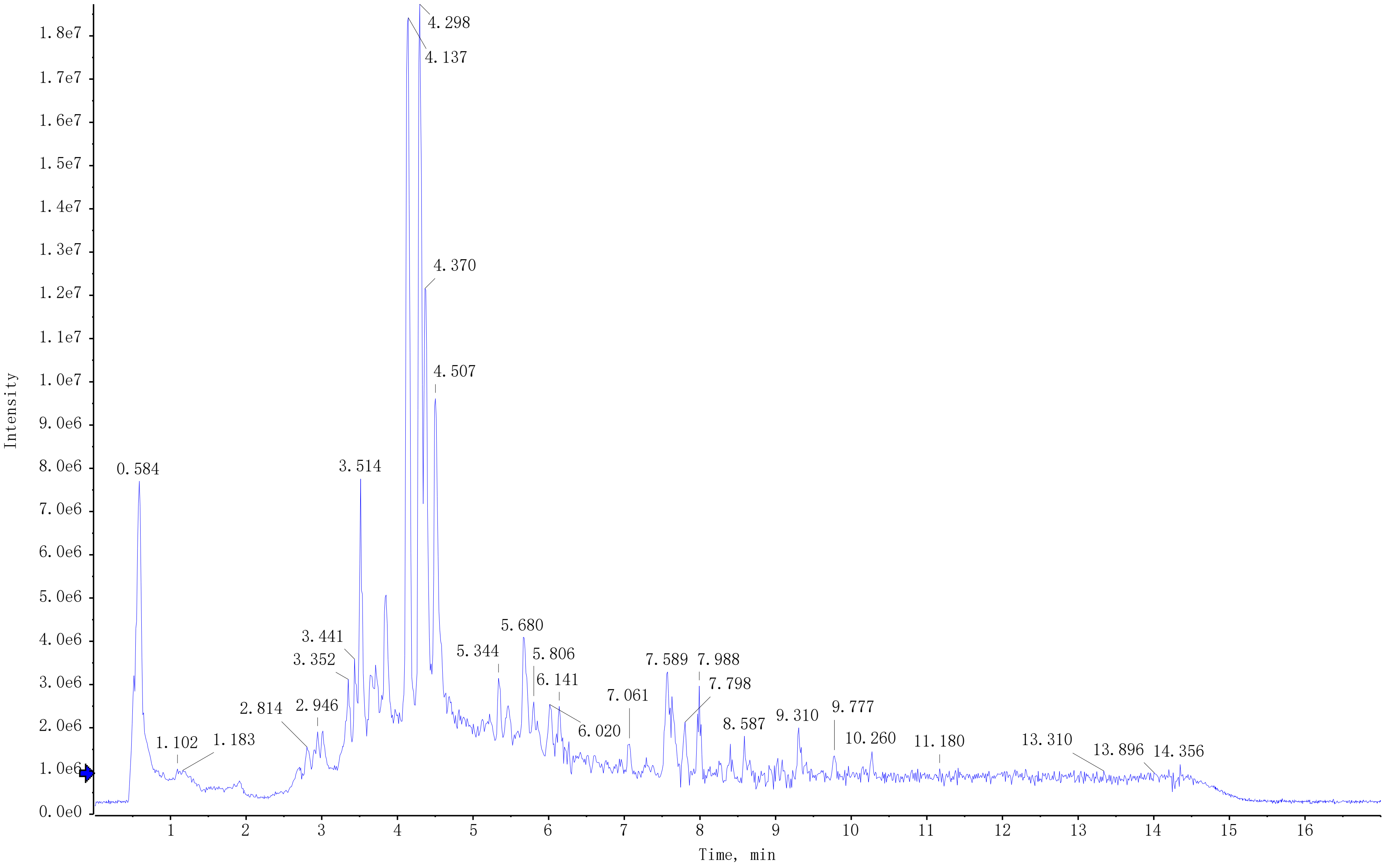

TIC from M5-1-NEG.wiff (sample 1) - M5-1-NEG, -TOF MS (50 - 1000)

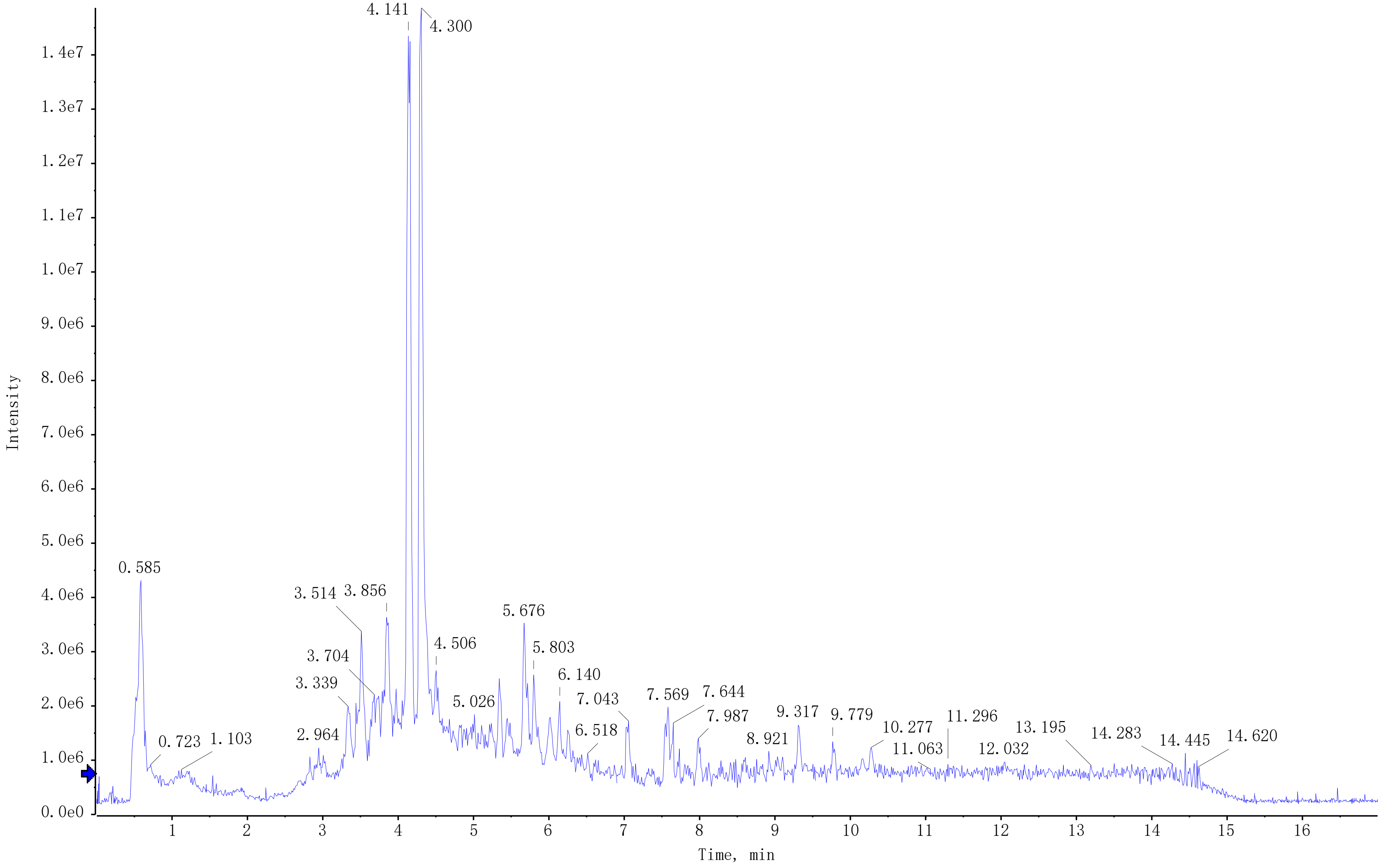

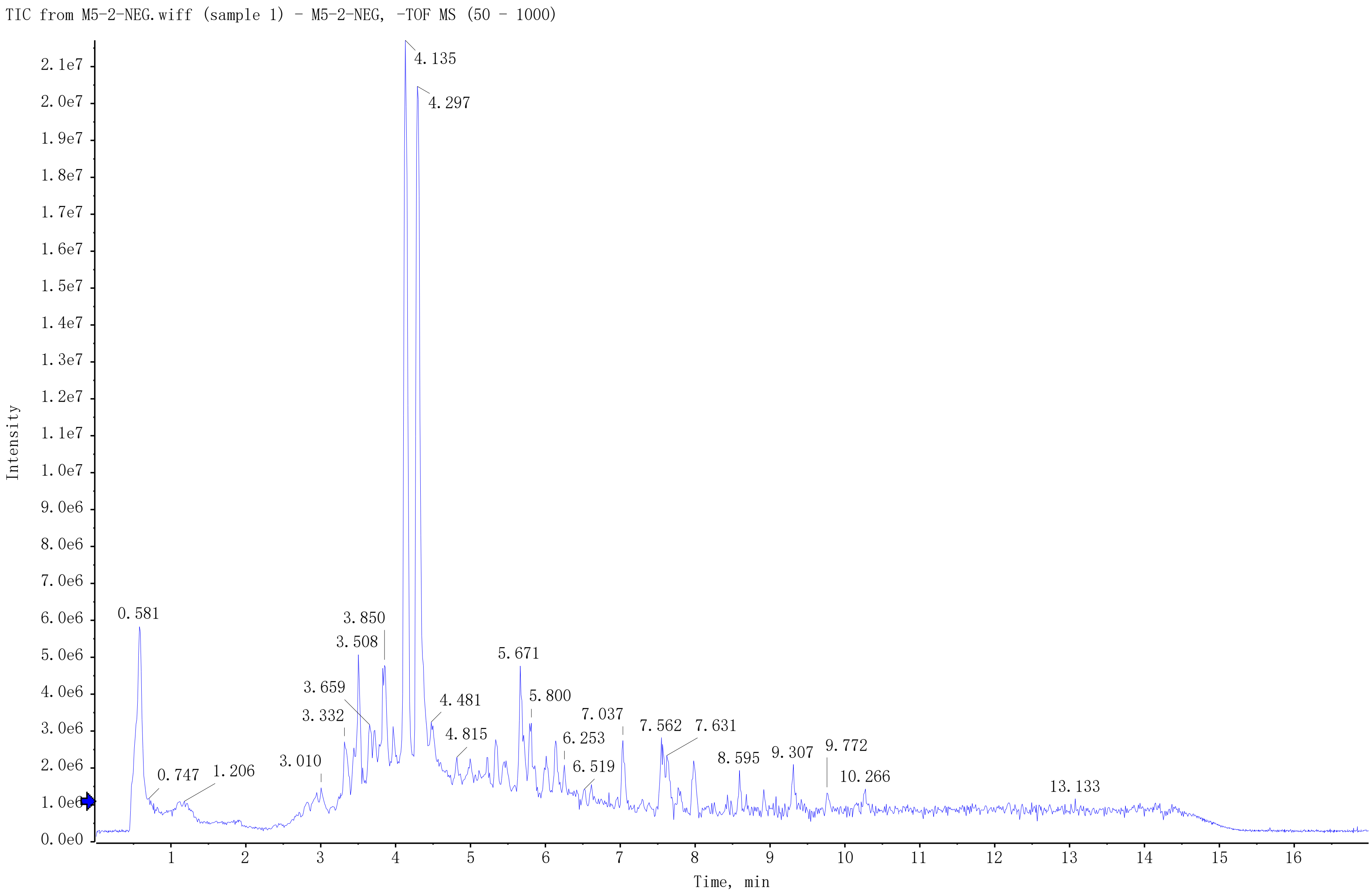
TIC from M5-2-NEG.wiff (sample 1) - M5-2-NEG, -TOF MS (50 - 1000)
Intensity
Time, min
0.581
0.747
1.206
3.010
3.332
3.659
3.508
3.850
4.135
4.297
4.481
4.815
5.671
5.800
6.253
6.519
7.037
7.562
7.631
8.595
9.307
9.772
10.266
13.133

TIC from M5-3-NEG.wiff (sample 1) - M5-3-NEG, -TOF MS (50 - 1000)

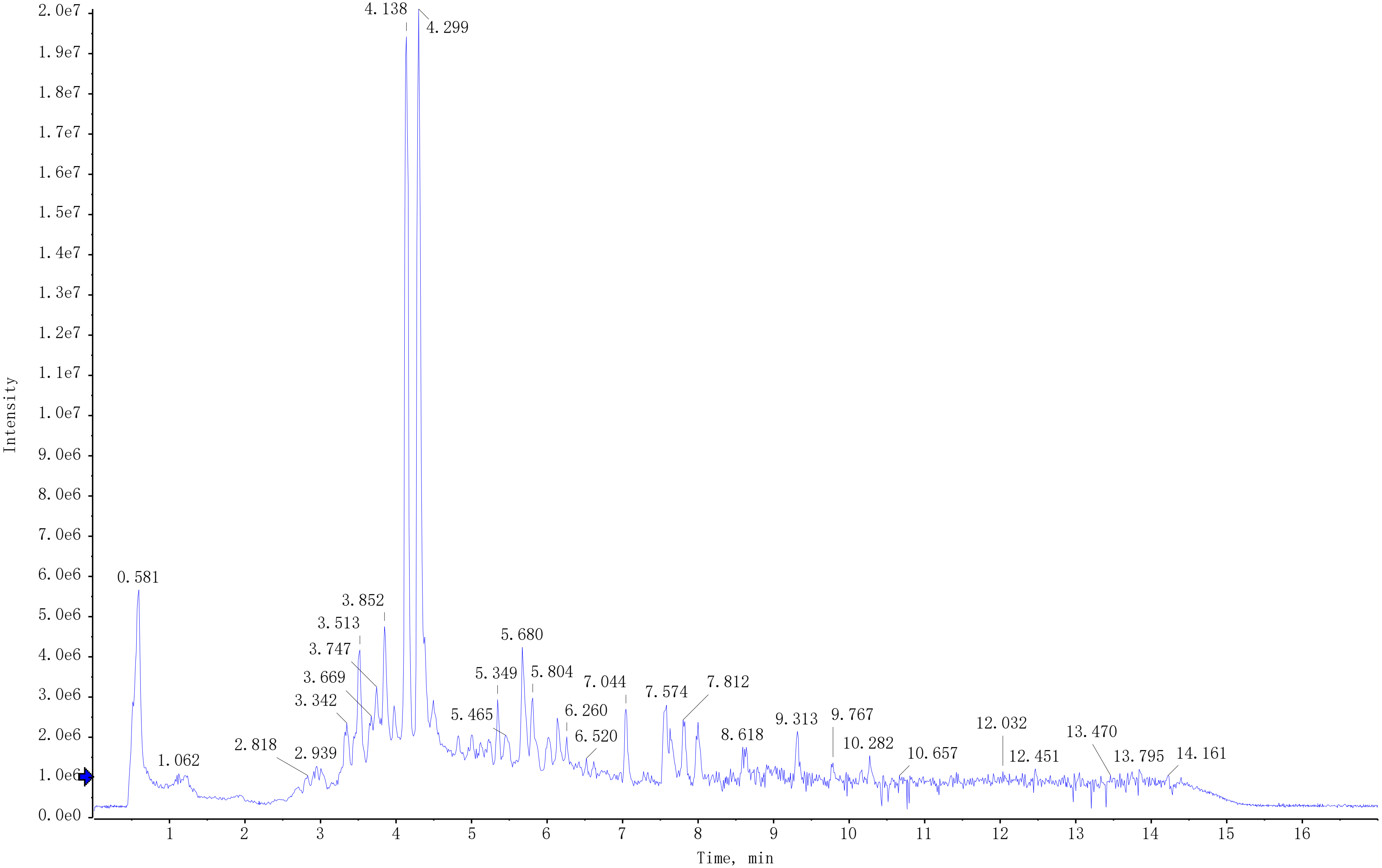

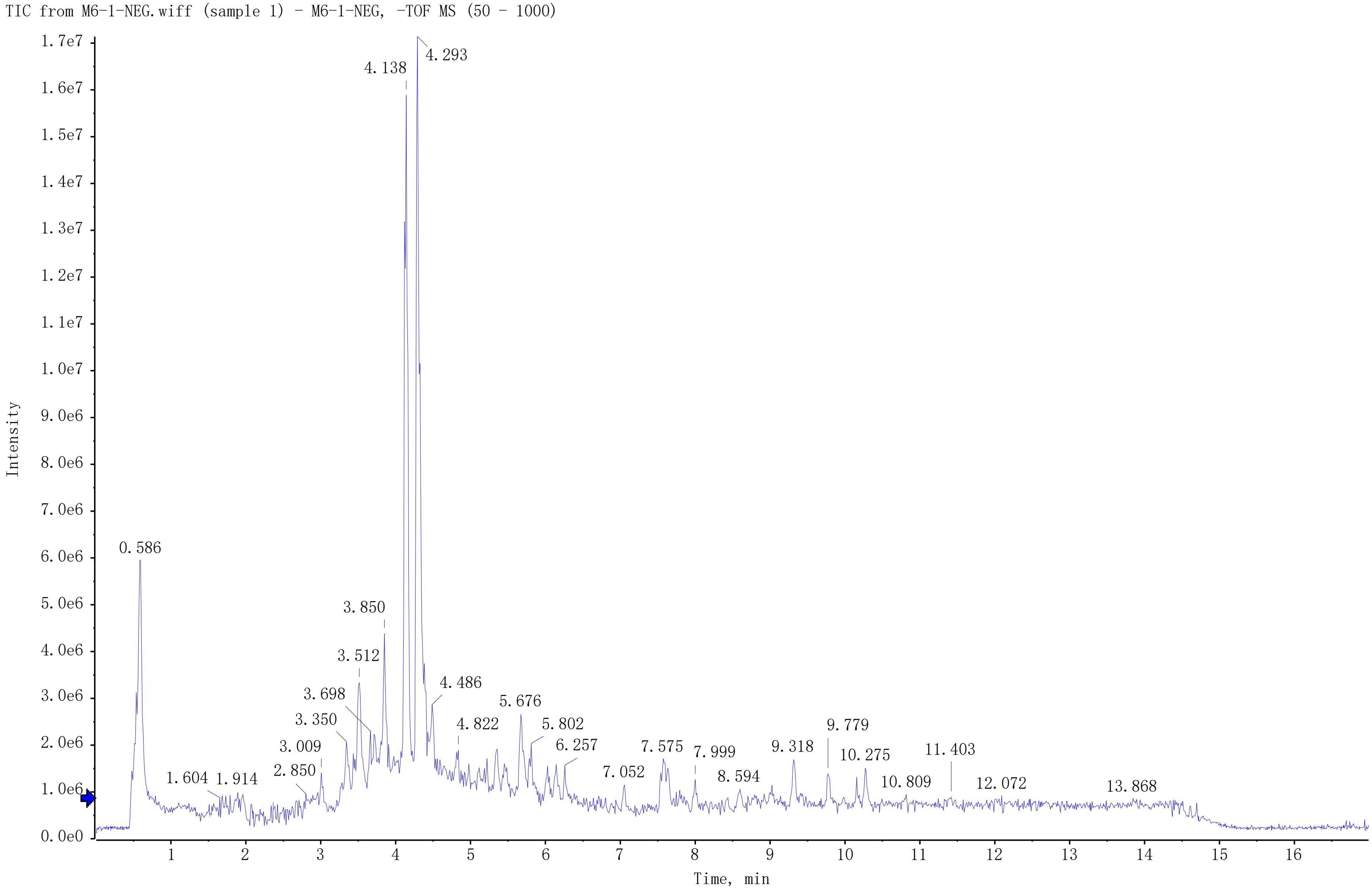
TIC from M6-1-NEG.wiff (sample 1) - M6-1-NEG, -TOF MS (50 - 1000)
Intensity
Time, min
1.7e7
1.6e7
1.5e7
1.4e7
1.3e7
1.2e7
1.1e7
1.0e7
9.0e6
8.0e6
7.0e6
6.0e6
5.0e6
4.0e6
3.0e6
2.0e6
1.0e6
0.0e0
1
2
3
4
5
6
7
8
9
10
11
12
13
14
15
16
0.586
1.604
1.914
2.850
3.009
3.350
3.698
3.512
3.850
4.138
4.293
4.486
4.822
5.676
5.802
6.257
7.052
7.575
7.999
8.594
9.318
9.779
10.275
10.809
11.403
12.072
13.868

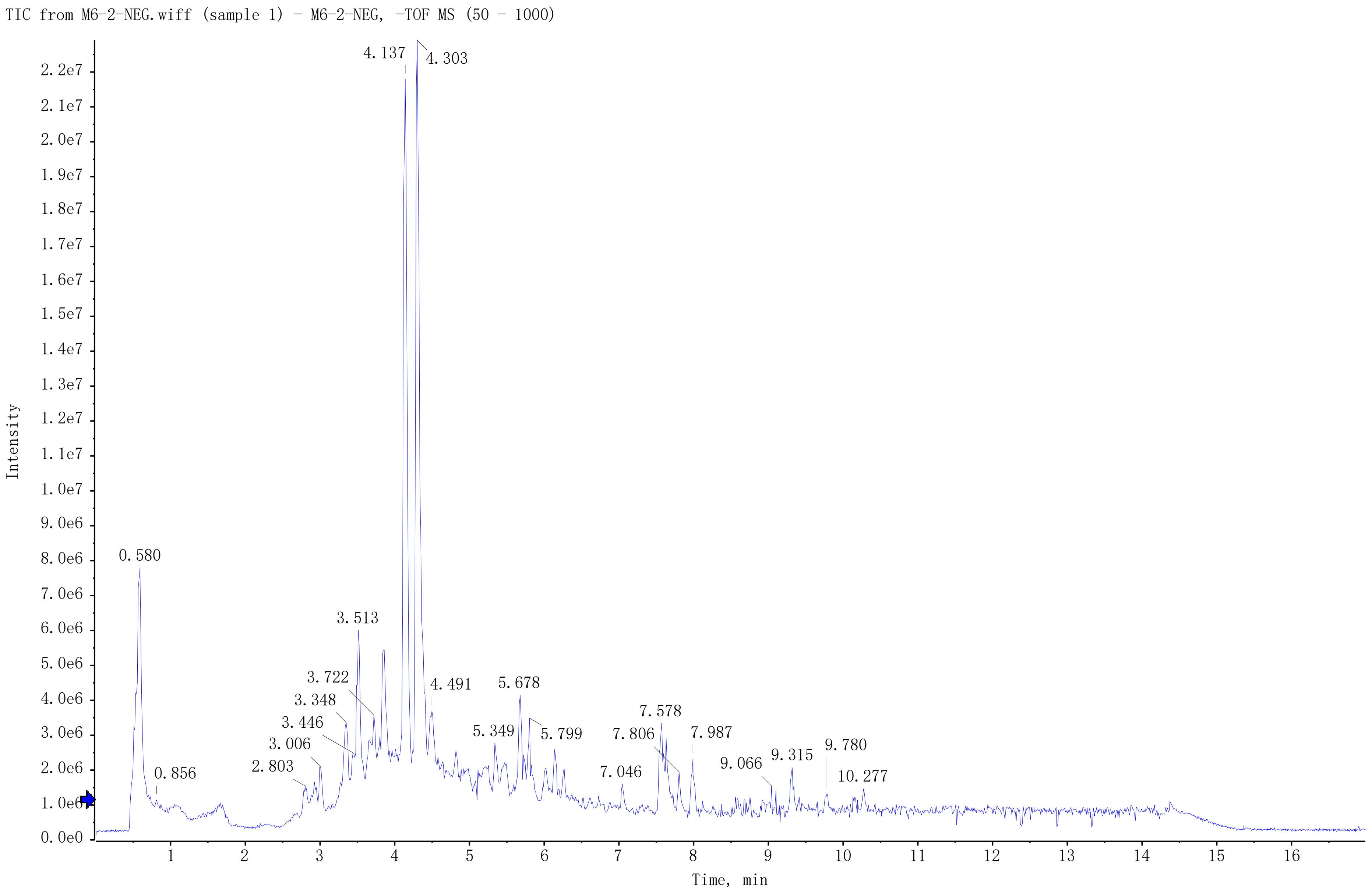

TIC from M6-2-NEG.wiff (sample 1) - M6-2-NEG, -TOF MS (50 - 1000)
Intensity
Time, min
4.137
4.303
0.580
0.856
2.803
3.006
3.446
3.348
3.722
3.513
4.491
5.349
5.678
5.799
7.046
7.806
7.578
7.987
9.066
9.315
9.780
10.277

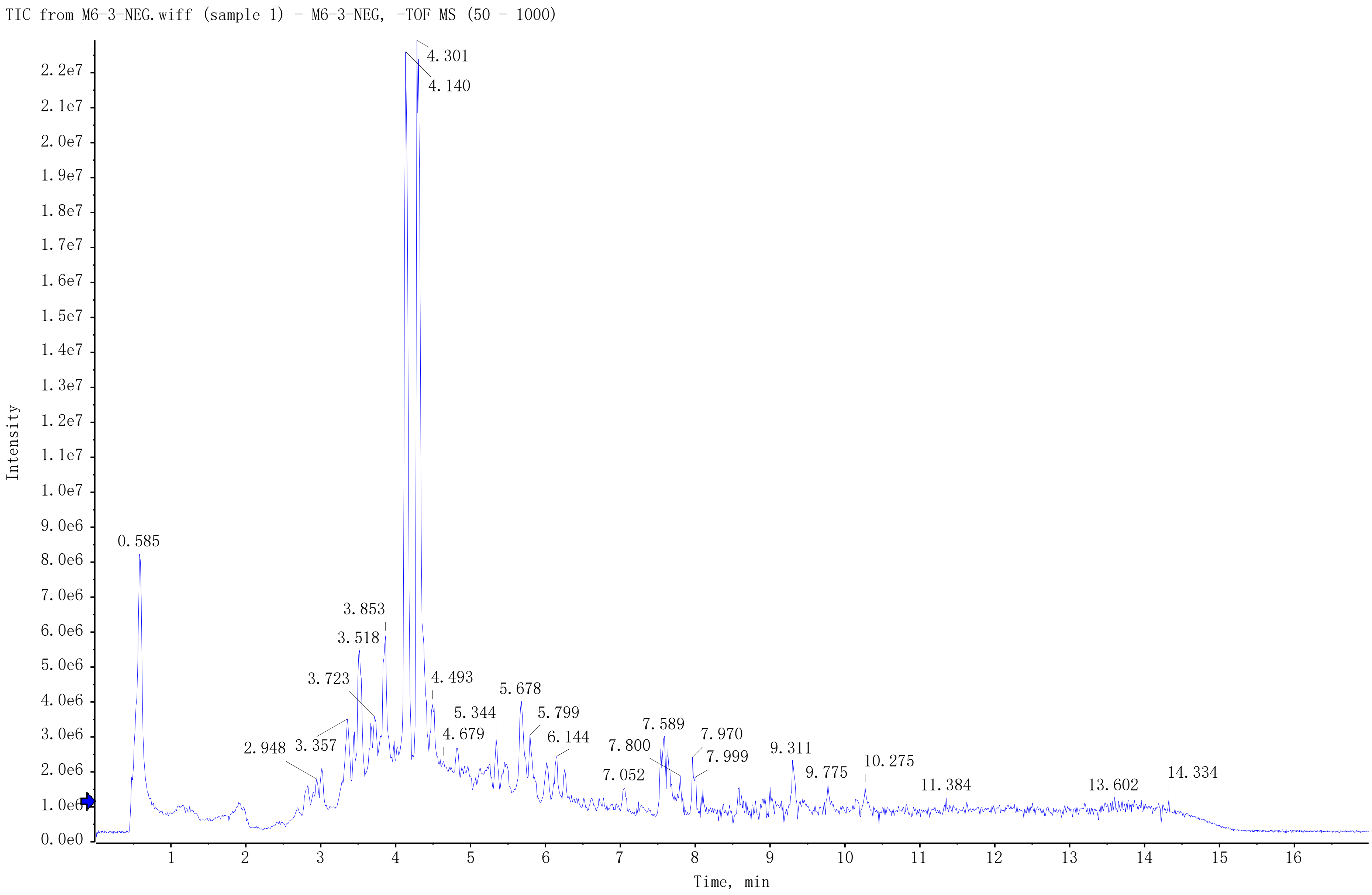
TIC from M6-3-NEG.wiff (sample 1) - M6-3-NEG, -TOF MS (50 - 1000)
Intensity
Time, min
0.585
2.948
3.357
3.518
3.723
3.853
4.140
4.301
4.493
4.679
5.344
5.678
5.799
6.144
7.052
7.589
7.800
7.970
7.999
9.311
9.775
10.275
11.384
13.602
14.334

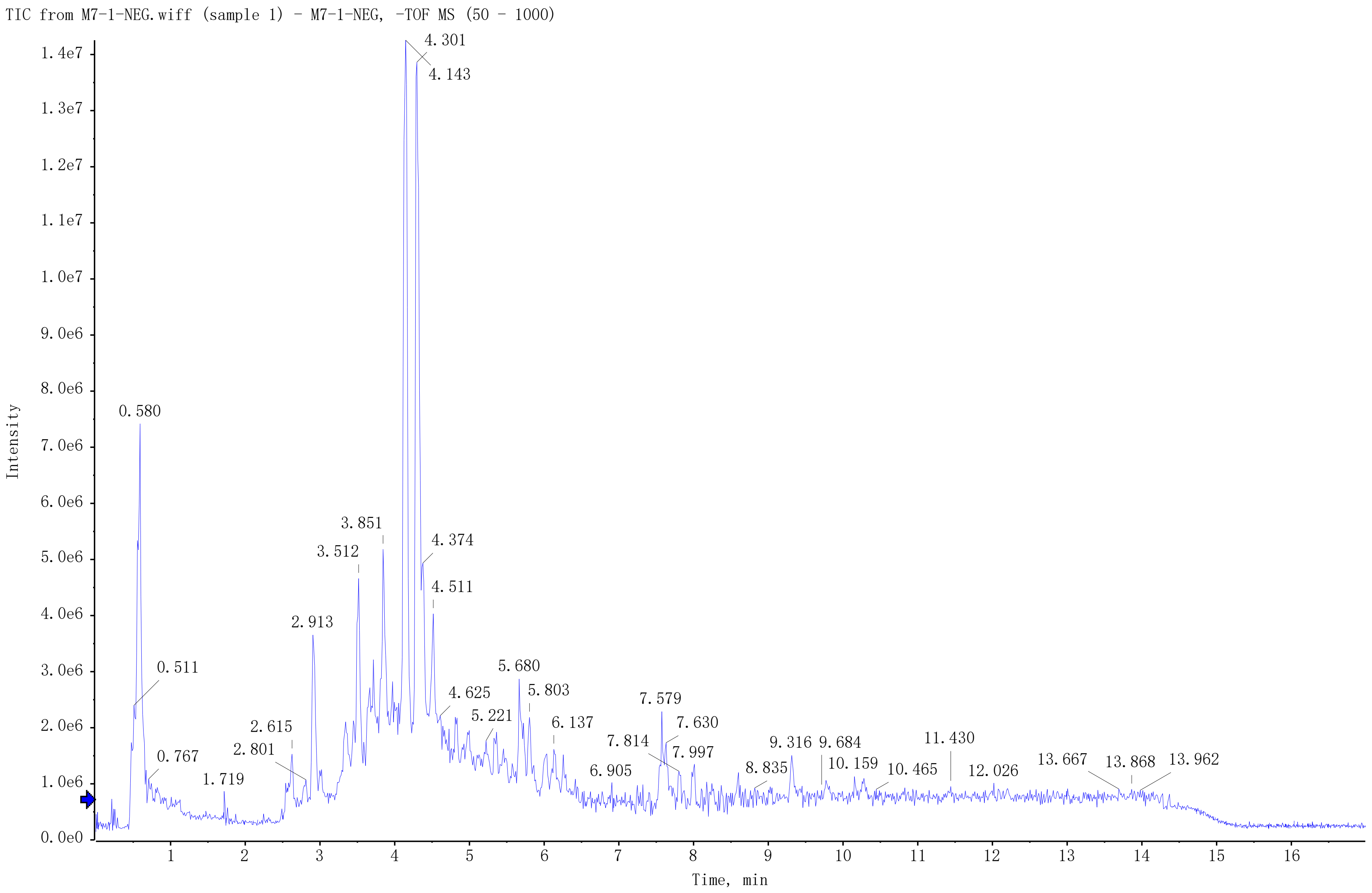

TIC from M7-1-NEG.wiff (sample 1) - M7-1-NEG, -TOF MS (50 - 1000)
Intensity
Time, min
1.4e7
1.3e7
1.2e7
1.1e7
1.0e7
9.0e6
8.0e6
7.0e6
6.0e6
5.0e6
4.0e6
3.0e6
2.0e6
1.0e6
0.0e0
1
2
3
4
5
6
7
8
9
10
11
12
13
14
15
16
4.301
4.143
0.580
3.851
4.374
3.512
4.511
2.913
0.511
5.680
5.803
4.625
7.579
5.221
2.615
6.137
7.630
7.814
2.801
9.316
9.684
11.430
7.997
0.767
10.159
13.667
13.868
13.962
1.719
6.905
8.835
10.465
12.026

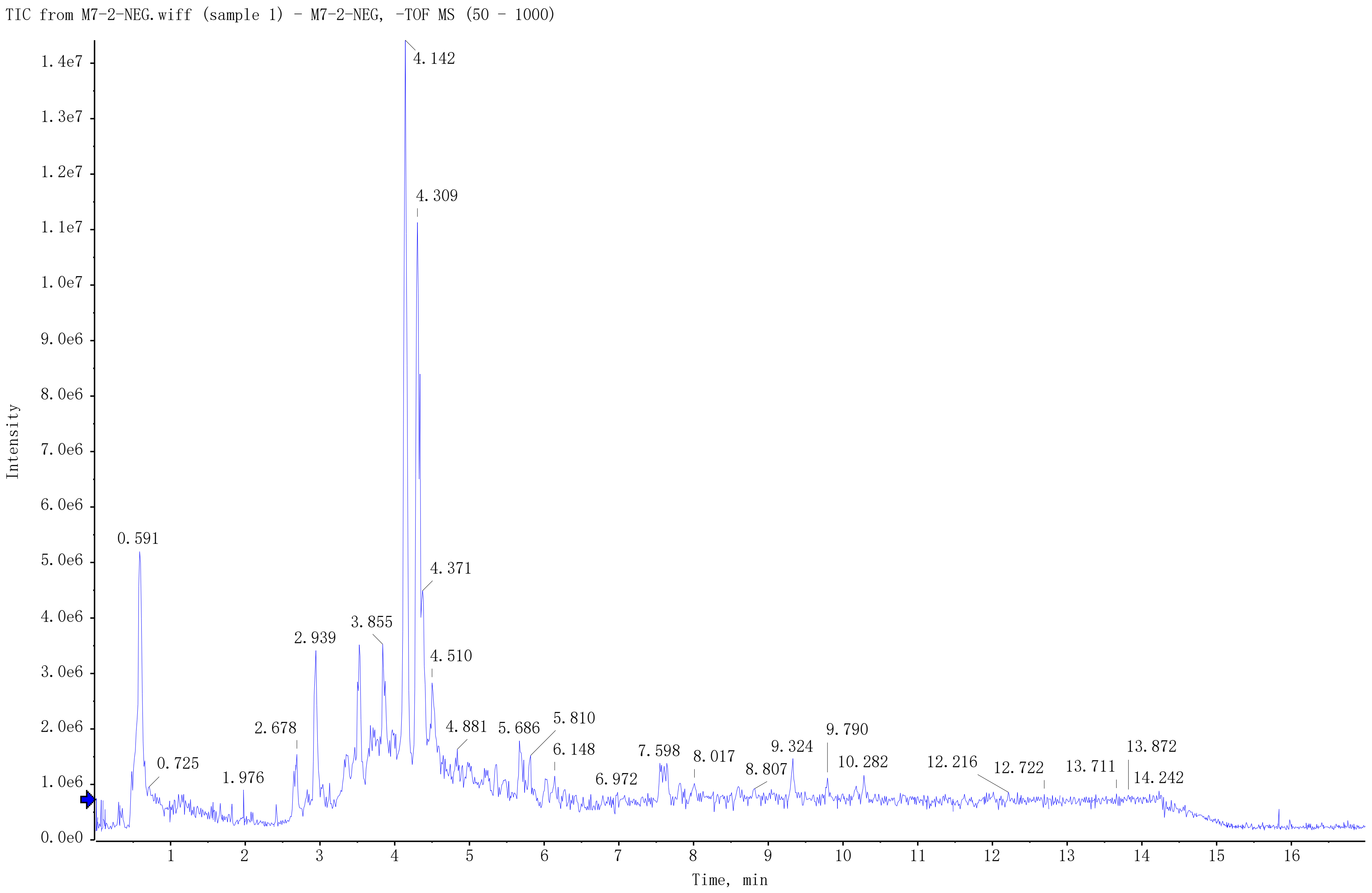
TIC from M7-2-NEG.wiff (sample 1) - M7-2-NEG, -TOF MS (50 - 1000)
Intensity
Time, min
4.142
4.309
4.371
4.510
0.591
0.725
1.976
2.678
2.939
3.855
4.881
5.686
5.810
6.148
6.972
7.598
8.017
8.807
9.324
9.790
10.282
12.216
12.722
13.711
13.872
14.242

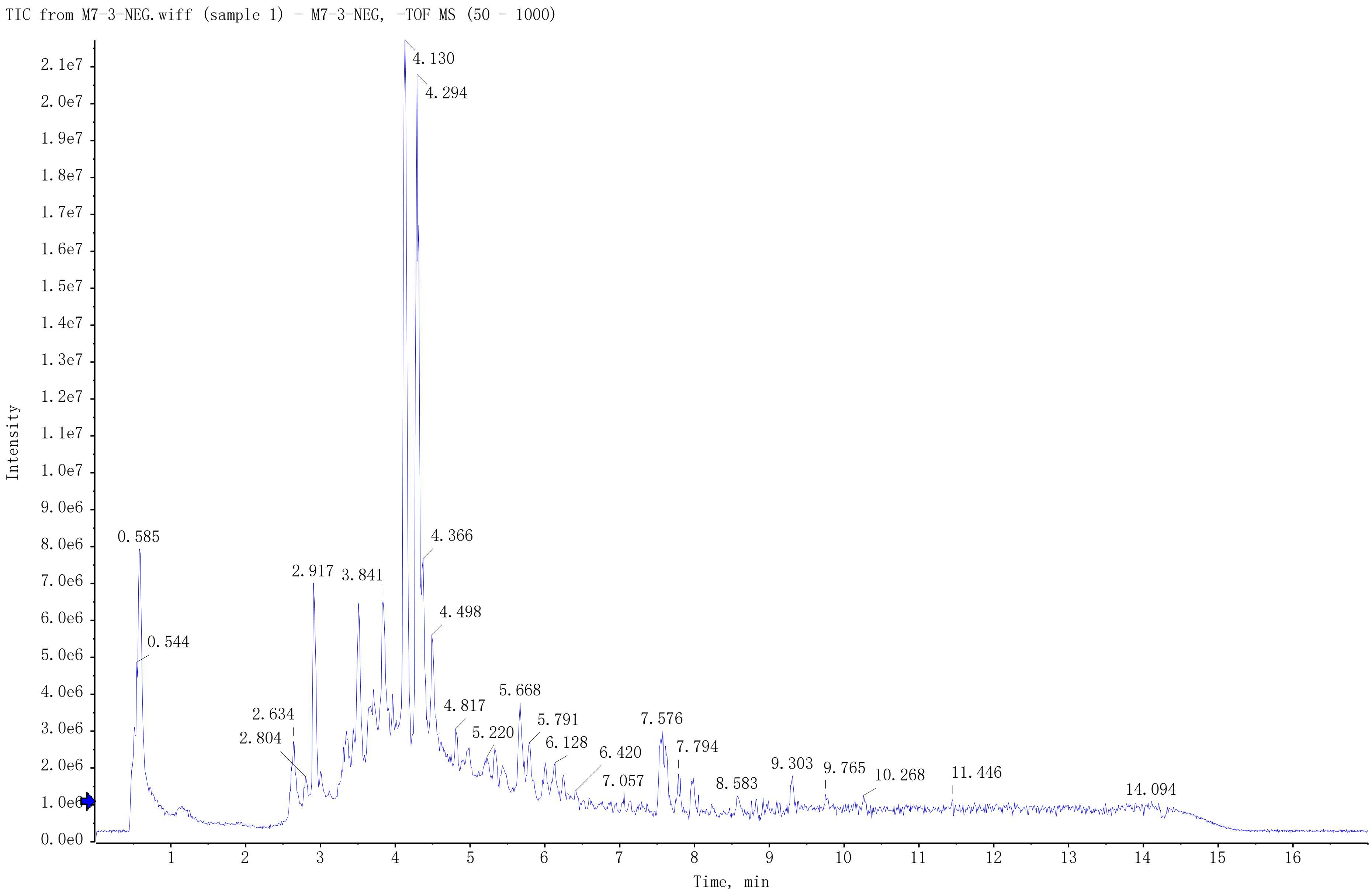

TIC from M7-3-NEG.wiff (sample 1) - M7-3-NEG, -TOF MS (50 - 1000)
Intensity
Time, min
0.585
0.544
2.634
2.804
2.917
3.841
4.130
4.294
4.366
4.498
4.817
5.220
5.668
5.791
6.128
6.420
7.057
7.576
7.794
8.583
9.303
9.765
10.268
11.446
14.094

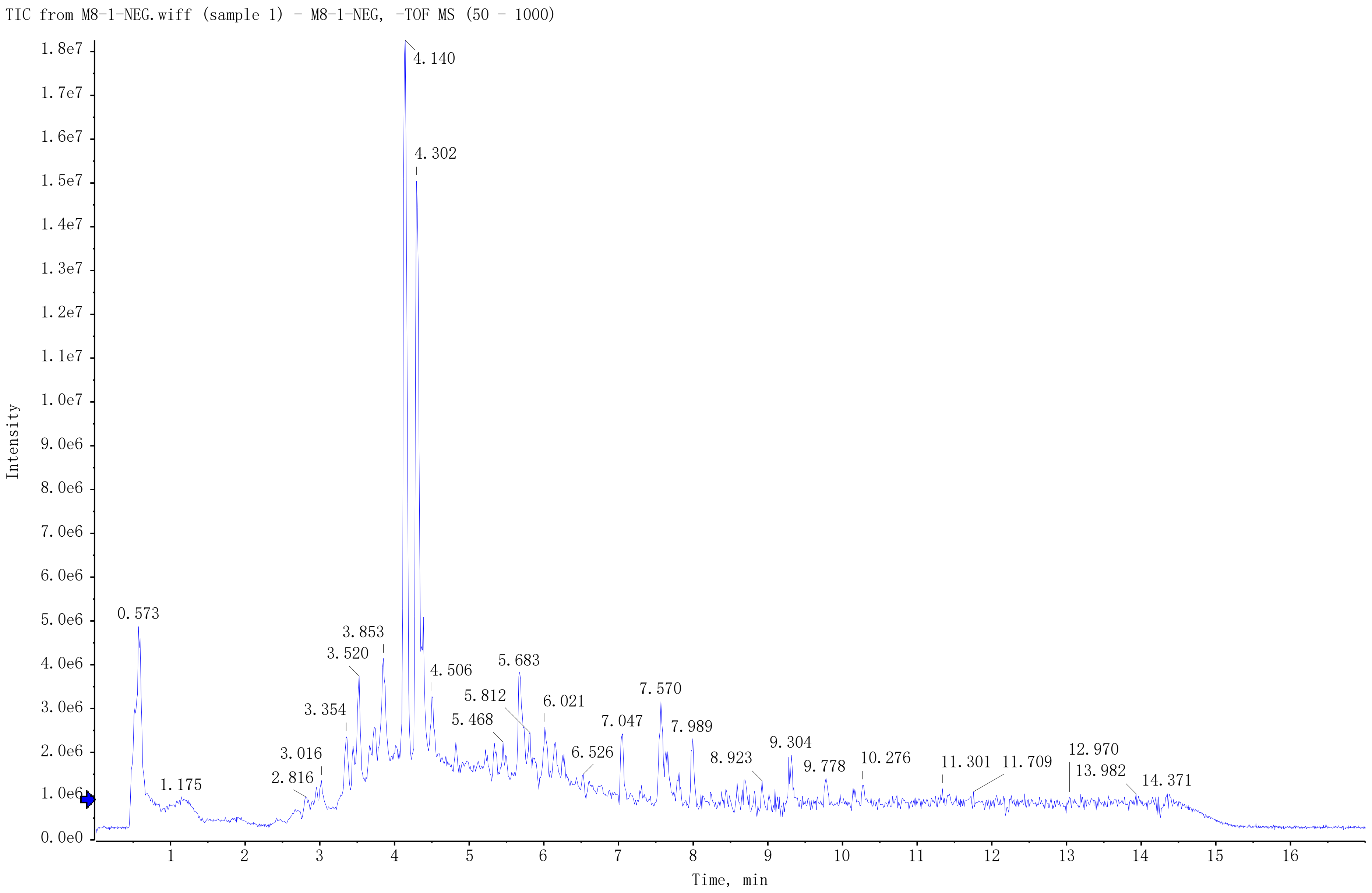
TIC from M8-1-NEG.wiff (sample 1) - M8-1-NEG, -TOF MS (50 - 1000)
Intensity
Time, min
4.140
4.302
0.573
1.175
2.816
3.016
3.354
3.520
3.853
4.506
5.468
5.812
5.683
6.021
6.526
7.047
7.570
7.989
8.923
9.304
9.778
10.276
11.301
11.709
12.970
13.982
14.371

TIC from M8-2-NEG.wiff (sample 1) - M8-2-NEG, -TOF MS (50 - 1000)

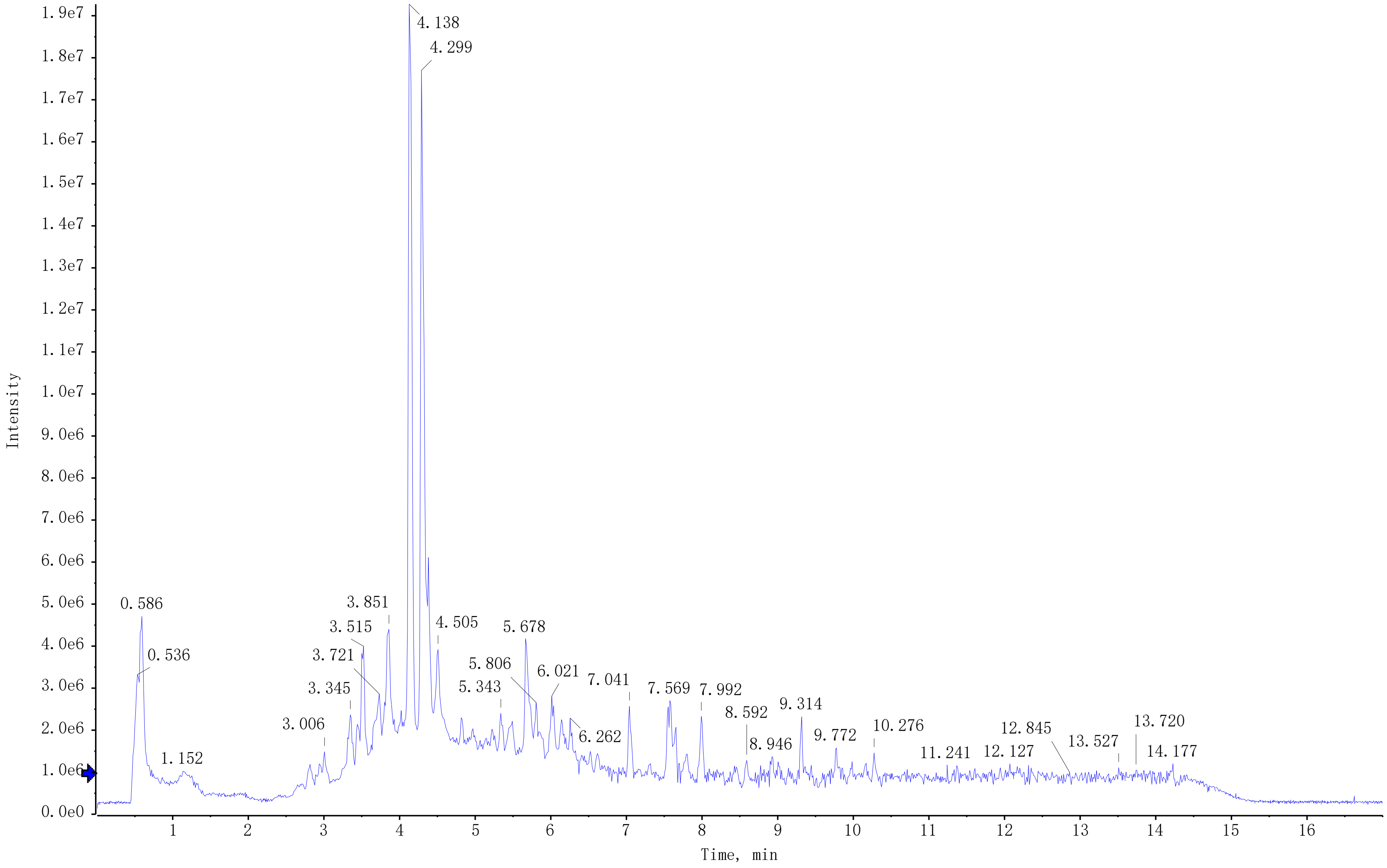

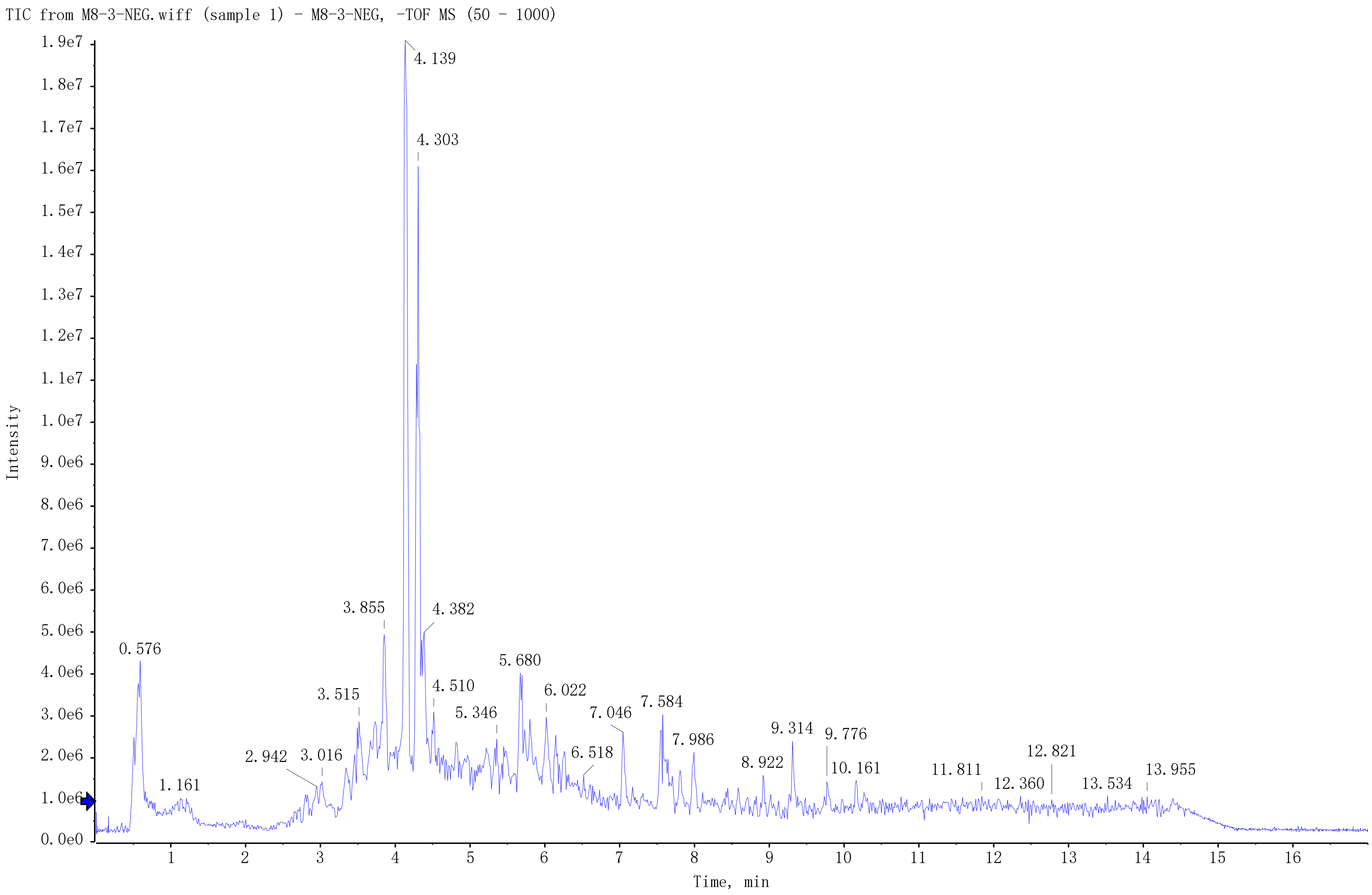
TIC from M8-3-NEG.wiff (sample 1) - M8-3-NEG, -TOF MS (50 - 1000)
Intensity
Time, min
4.139
4.303
3.855
4.382
0.576
5.680
3.515
4.510
6.022
5.346
7.046
7.584
2.942
3.016
6.518
7.986
9.314
9.776
12.821
8.922
10.161
11.811
13.955
1.161
12.360
13.534

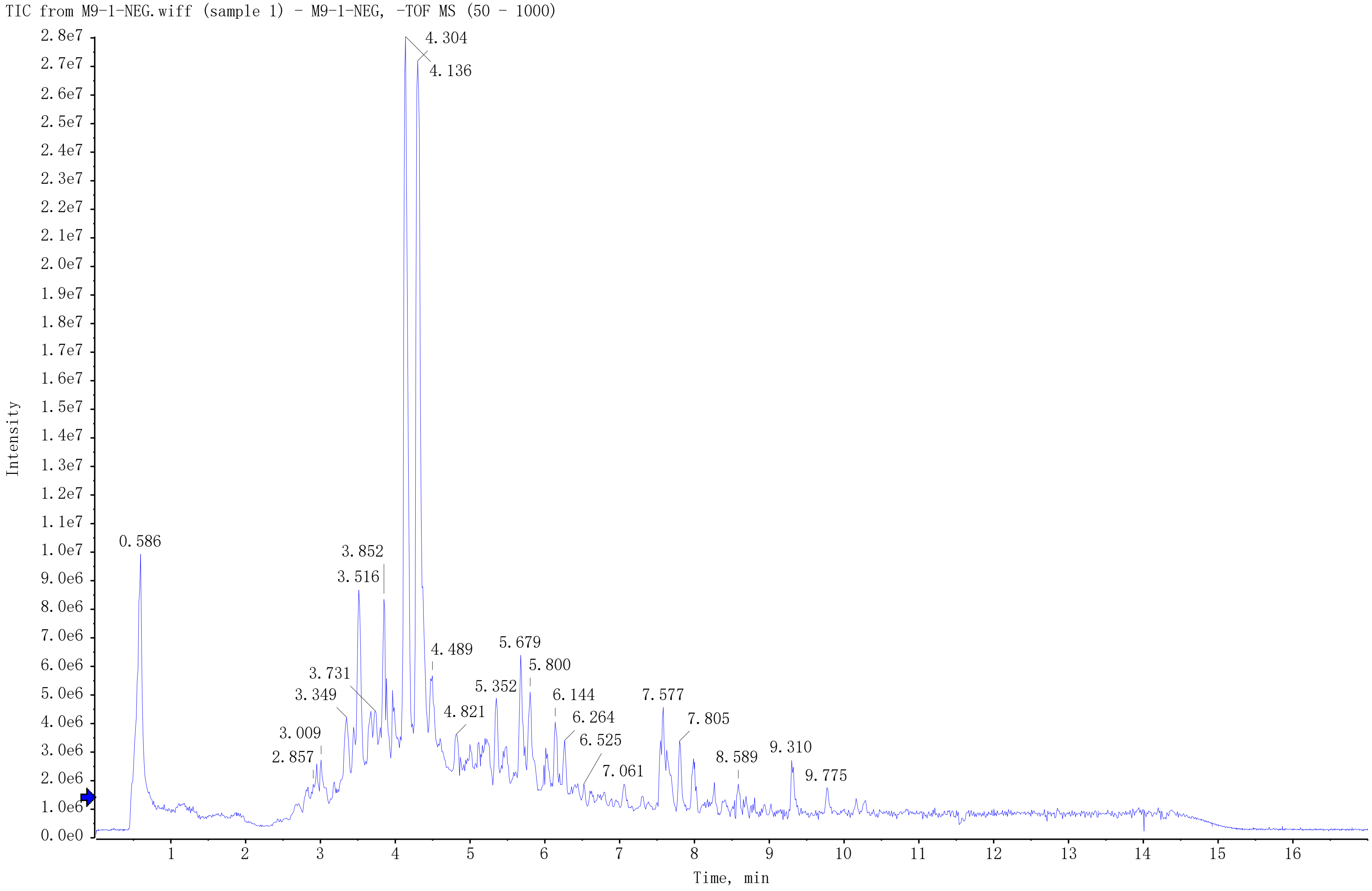

TIC from M9-1-NEG.wiff (sample 1) - M9-1-NEG, -TOF MS (50 - 1000)
Intensity
Time, min
4.304
4.136
0.586
3.852
3.516
3.731
3.349
3.009
2.857
4.489
4.821
5.352
5.679
5.800
6.144
6.264
6.525
7.061
7.577
7.805
8.589
9.310
9.775

TIC from M9-2-NEG.wiff (sample 1) - M9-2-NEG, -TOF MS (50 - 1000)

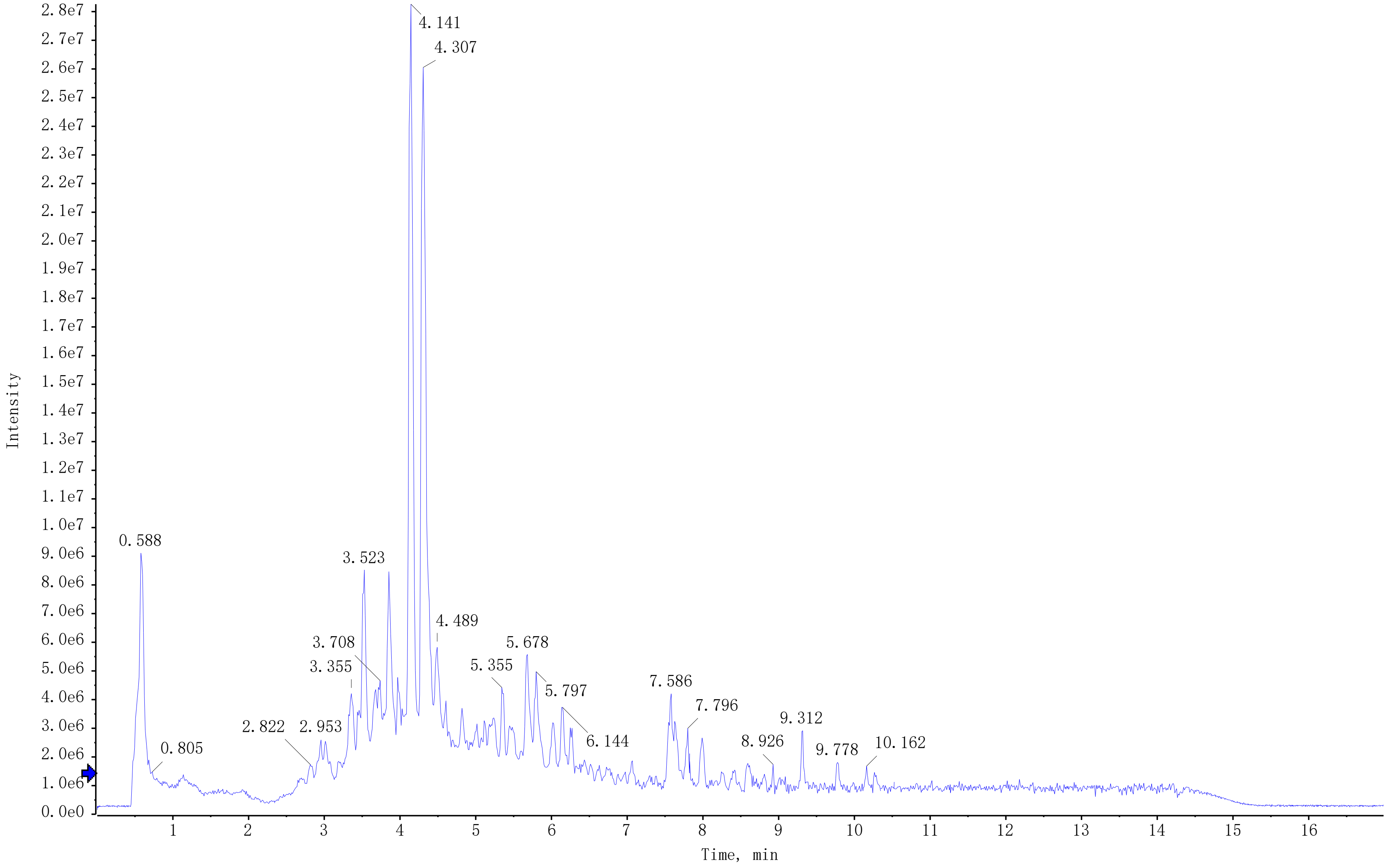

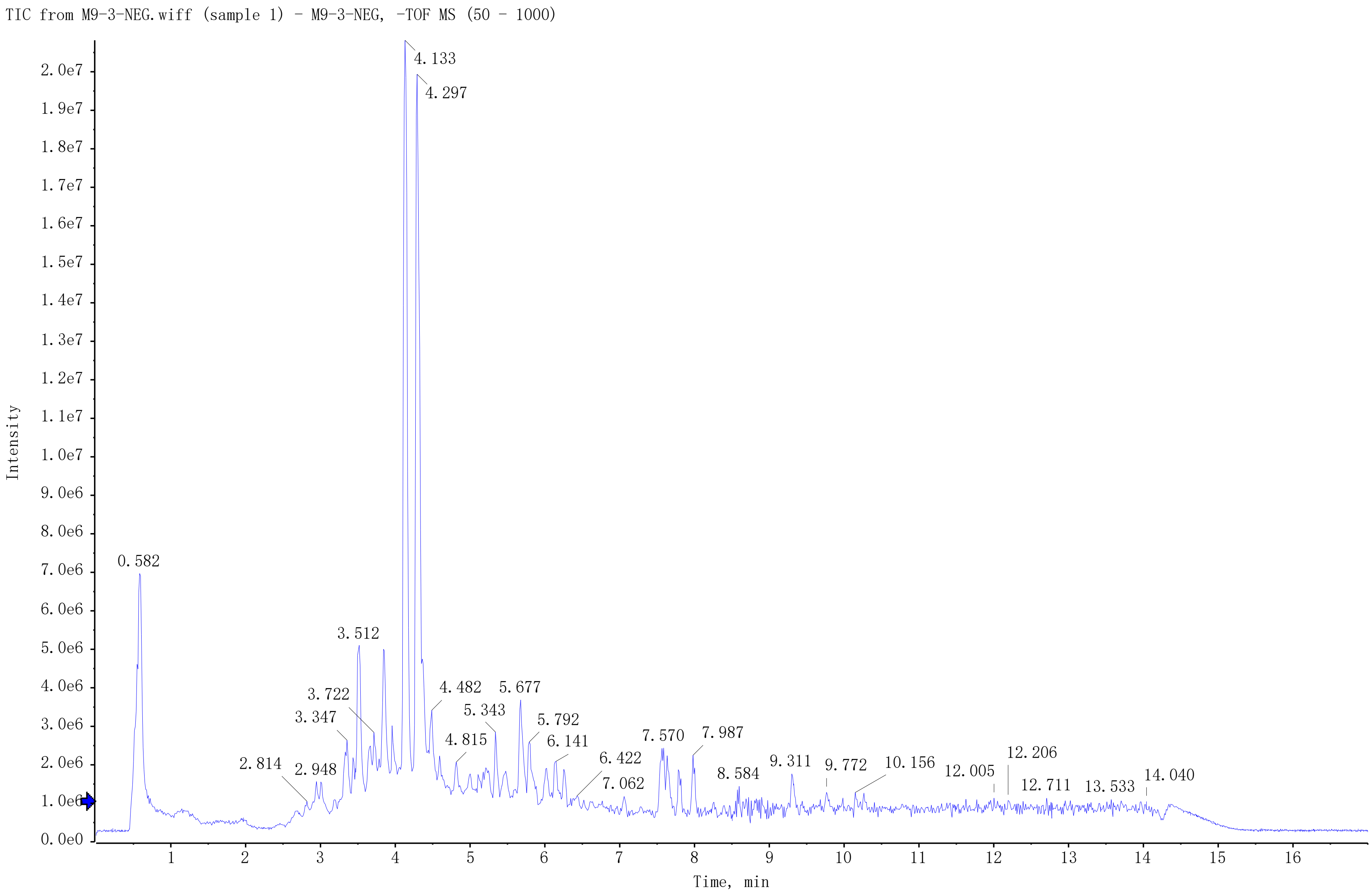

TIC from M9-3-NEG.wiff (sample 1) - M9-3-NEG, -TOF MS (50 - 1000)
Intensity
Time, min
0.582
2.814
2.948
3.347
3.512
3.722
4.133
4.297
4.482
4.815
5.343
5.677
5.792
6.141
6.422
7.062
7.570
7.987
8.584
9.311
9.772
10.156
12.005
12.206
12.711
13.533
14.040

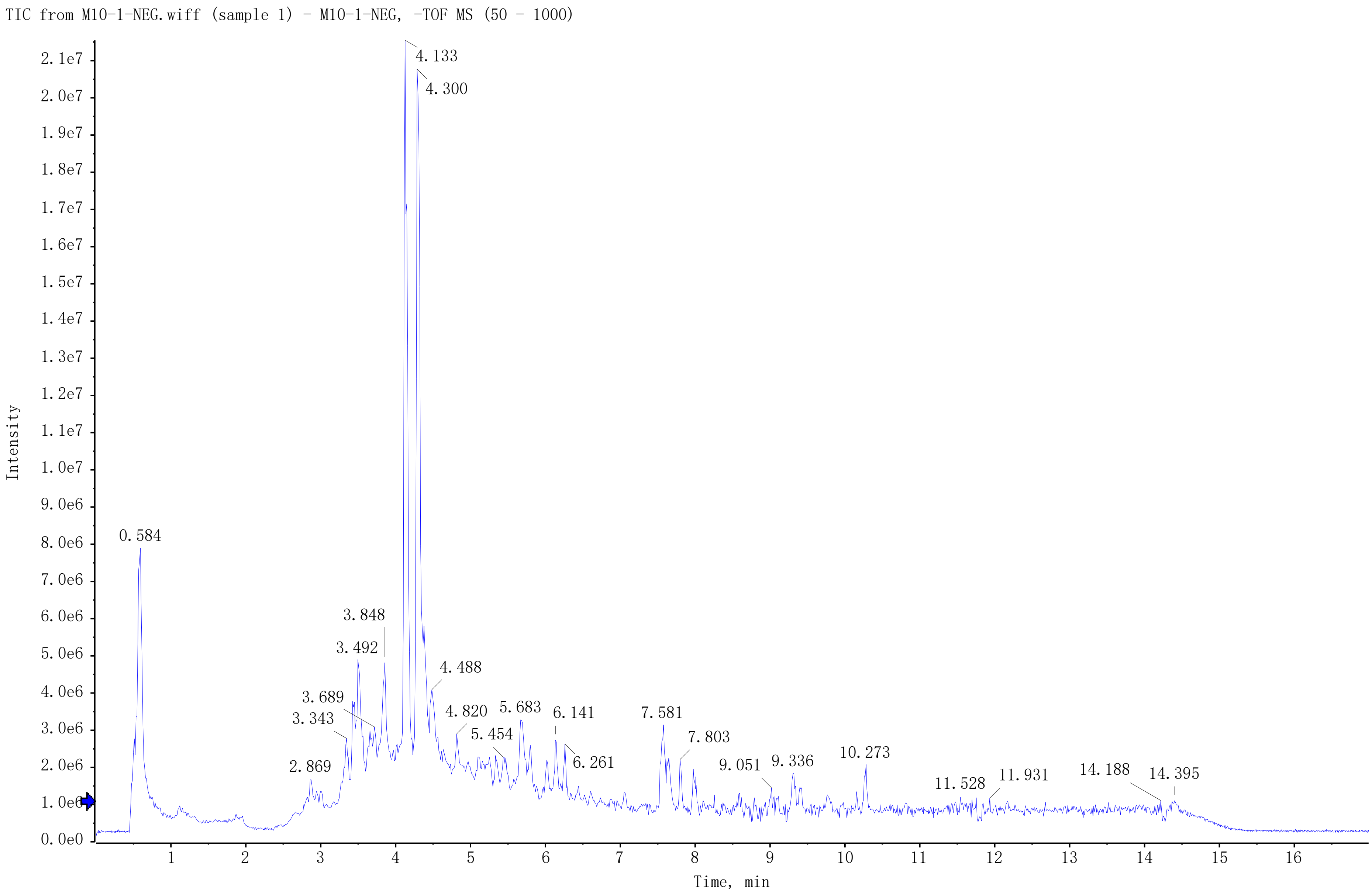
TIC from M10-1-NEG.wiff (sample 1) - M10-1-NEG, -TOF MS (50 - 1000)
Intensity
Time, min
0.584
2.869
3.343
3.492
3.689
3.848
4.133
4.300
4.488
4.820
5.454
5.683
6.141
6.261
7.581
7.803
9.051
9.336
10.273
11.528
11.931
14.188
14.395

TIC from M10-2-NEG.wiff (sample 1) - M10-2-NEG, -TOF MS (50 - 1000)

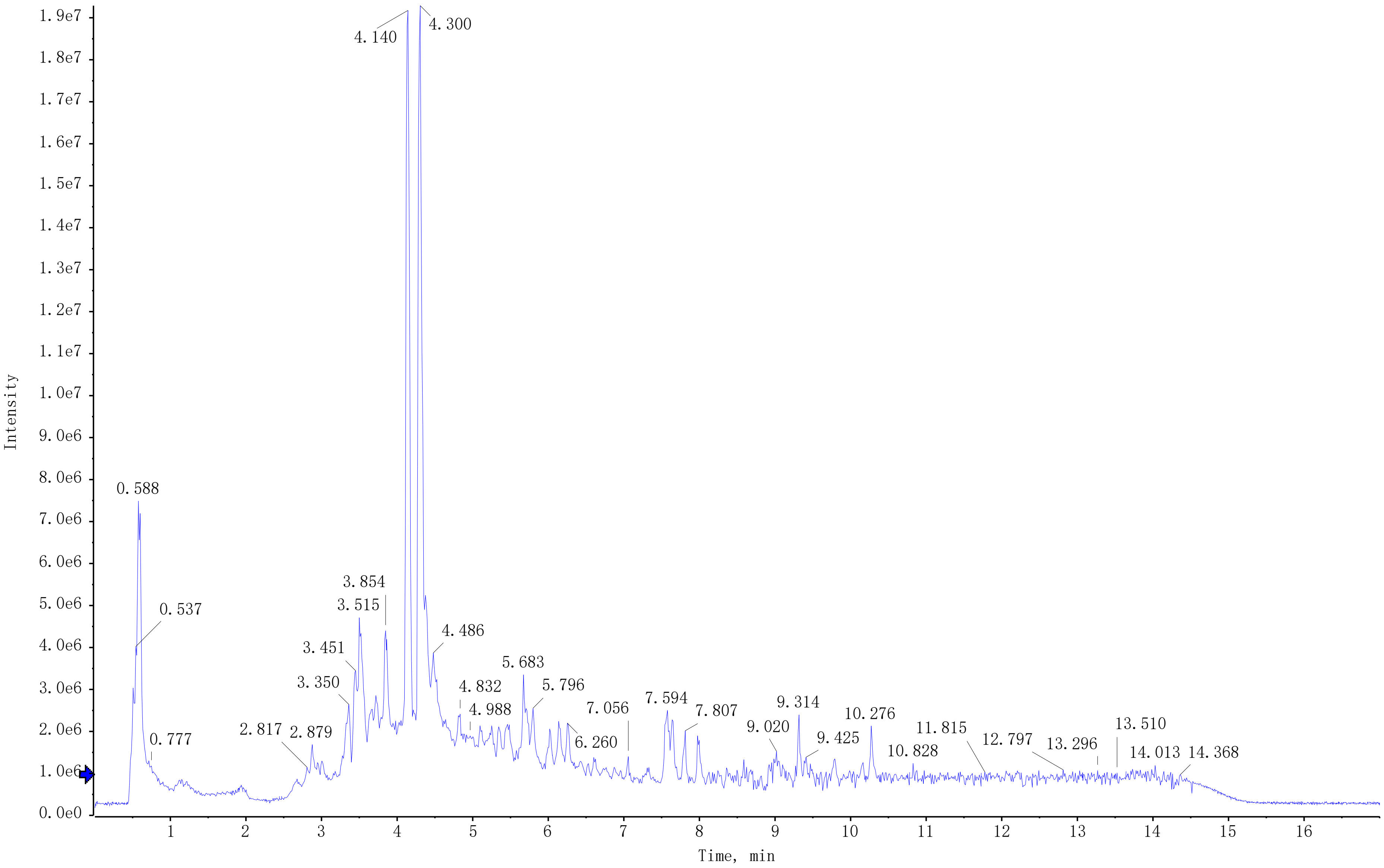

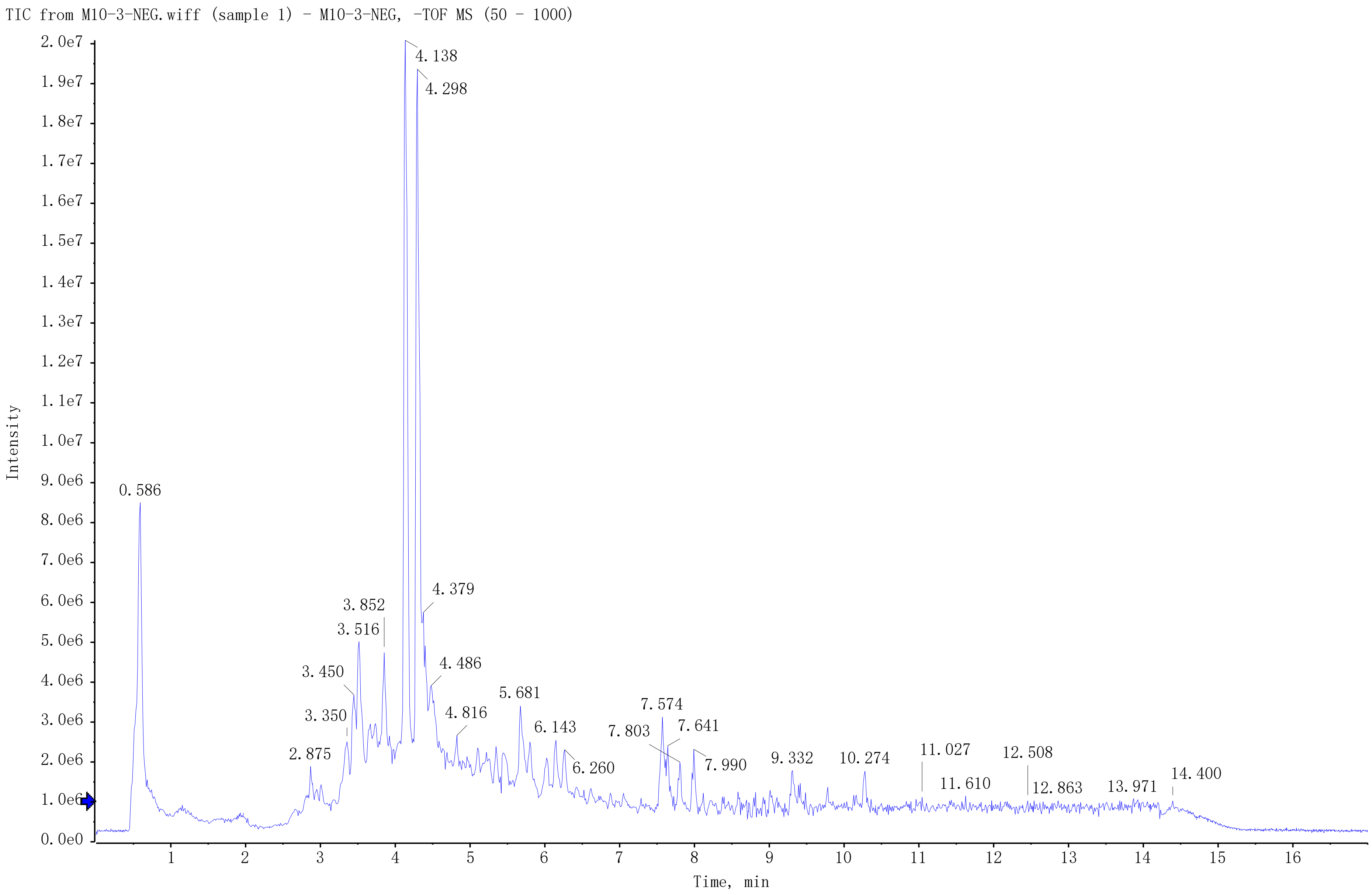

TIC from M10-3-NEG.wiff (sample 1) - M10-3-NEG, -TOF MS (50 - 1000)
Intensity
Time, min
2.0e7
1.9e7
1.8e7
1.7e7
1.6e7
1.5e7
1.4e7
1.3e7
1.2e7
1.1e7
1.0e7
9.0e6
8.0e6
7.0e6
6.0e6
5.0e6
4.0e6
3.0e6
2.0e6
1.0e6
0.0e0
1
2
3
4
5
6
7
8
9
10
11
12
13
14
15
16
0.586
2.875
3.350
3.450
3.516
3.852
4.138
4.298
4.379
4.486
4.816
5.681
6.143
6.260
7.574
7.641
7.803
7.990
9.332
10.274
11.027
11.610
12.508
12.863
13.971
14.400

TIC from M11-1-NEG.wiff (sample 1) - M11-1-NEG, -TOF MS (50 - 1000)

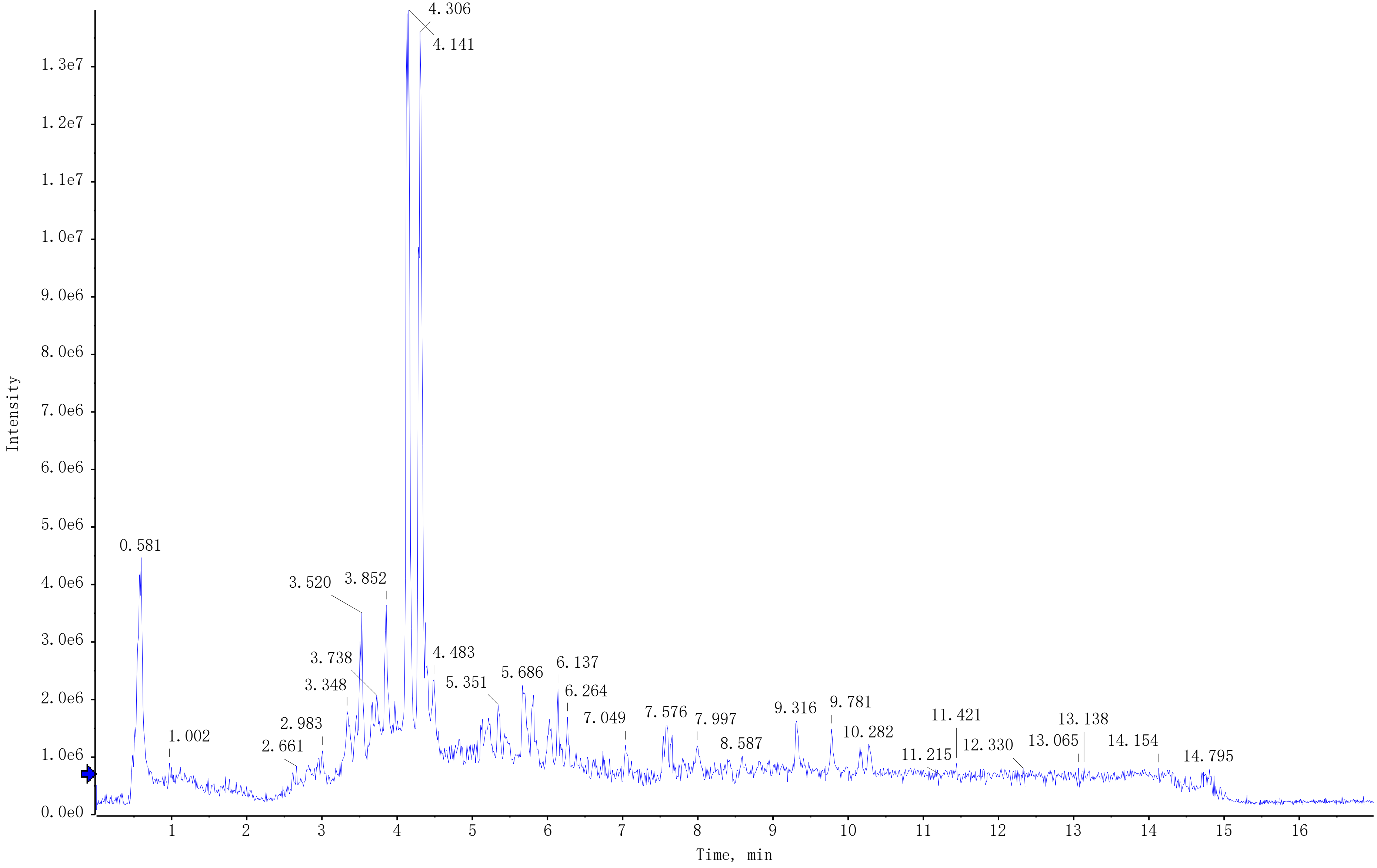

TIC from M11-2-NEG.wiff (sample 1) - M11-2-NEG, -TOF MS (50 - 1000)

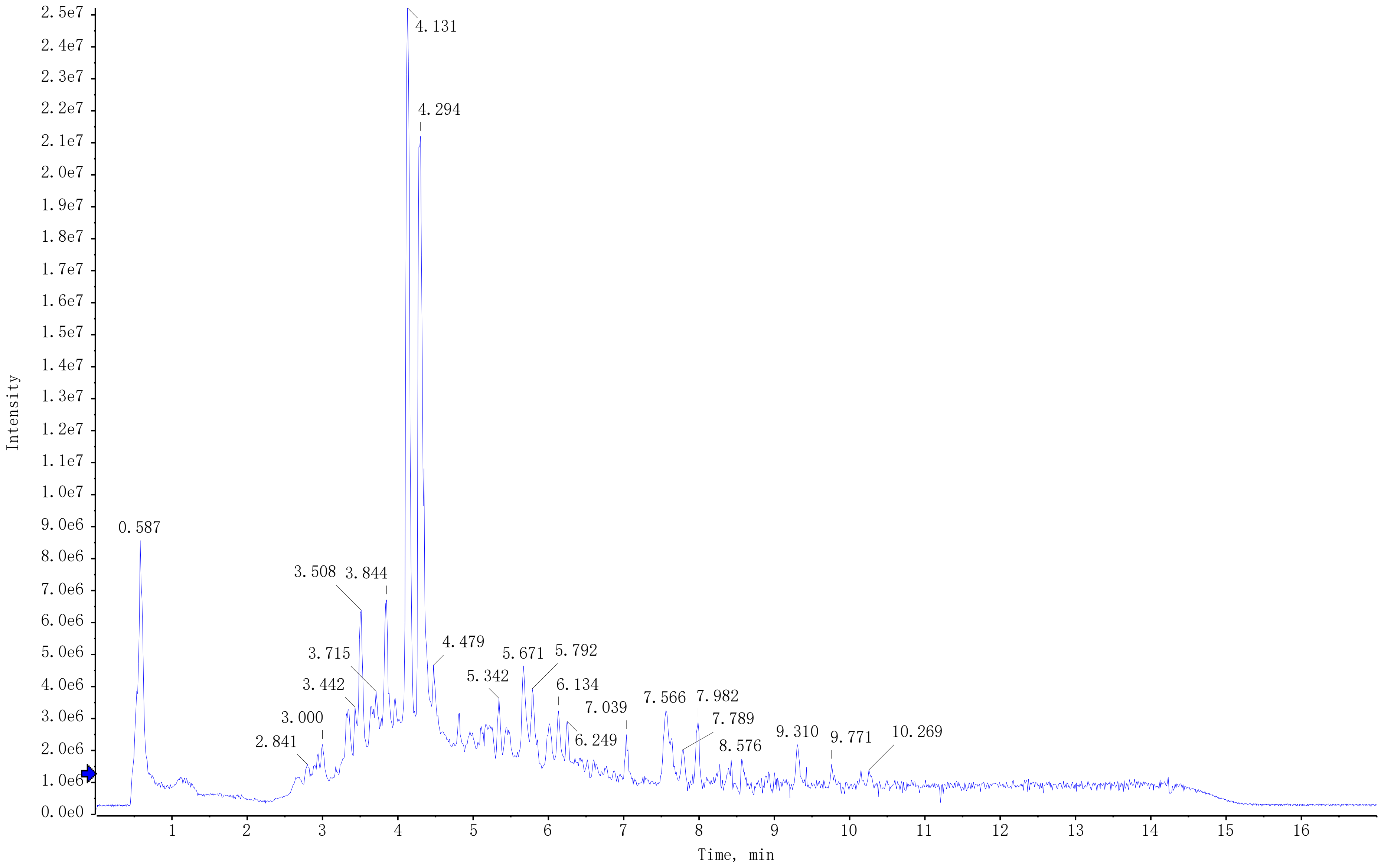

TIC from M11-3-NEG.wiff (sample 1) - M11-3-NEG, -TOF MS (50 - 1000)

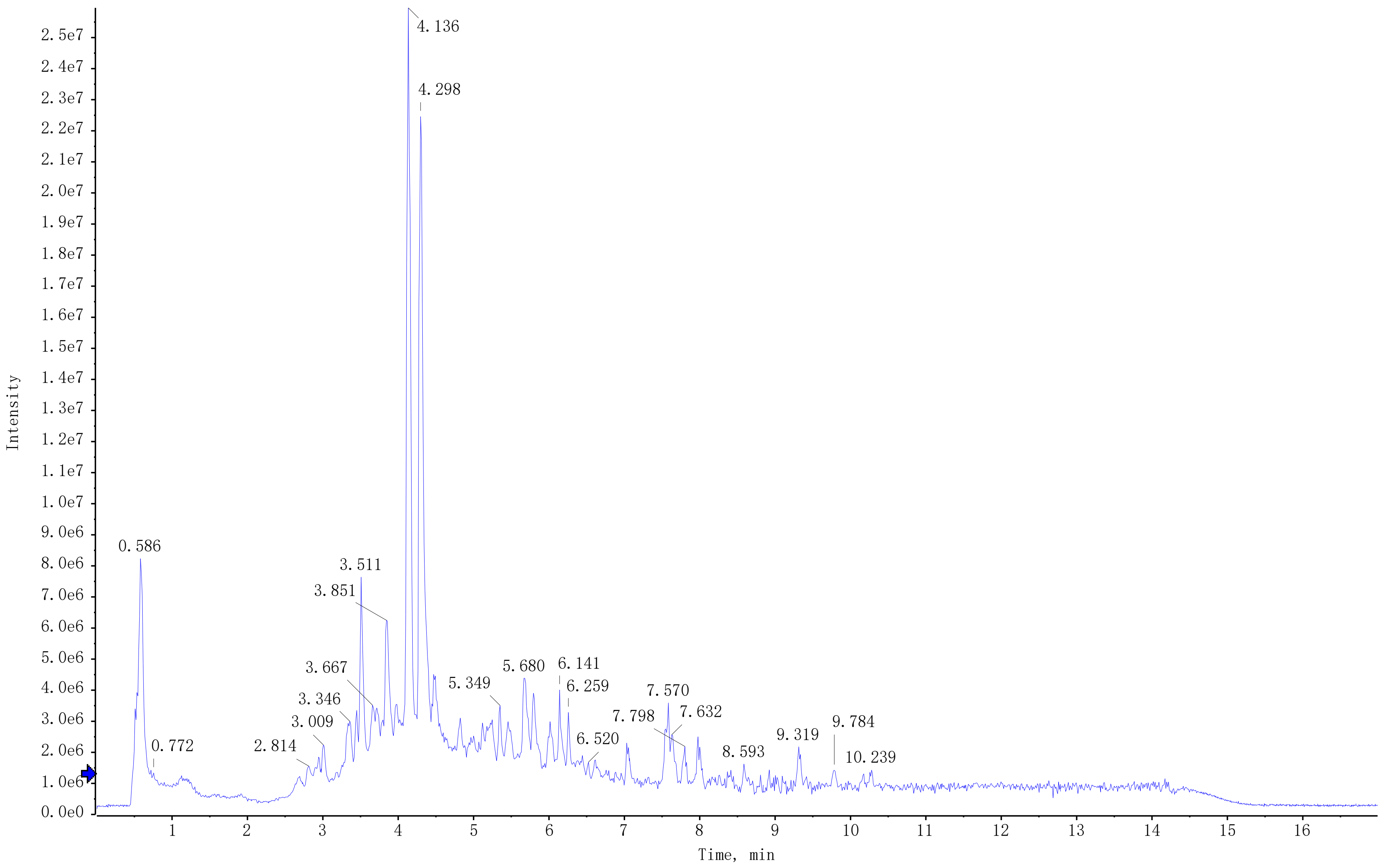

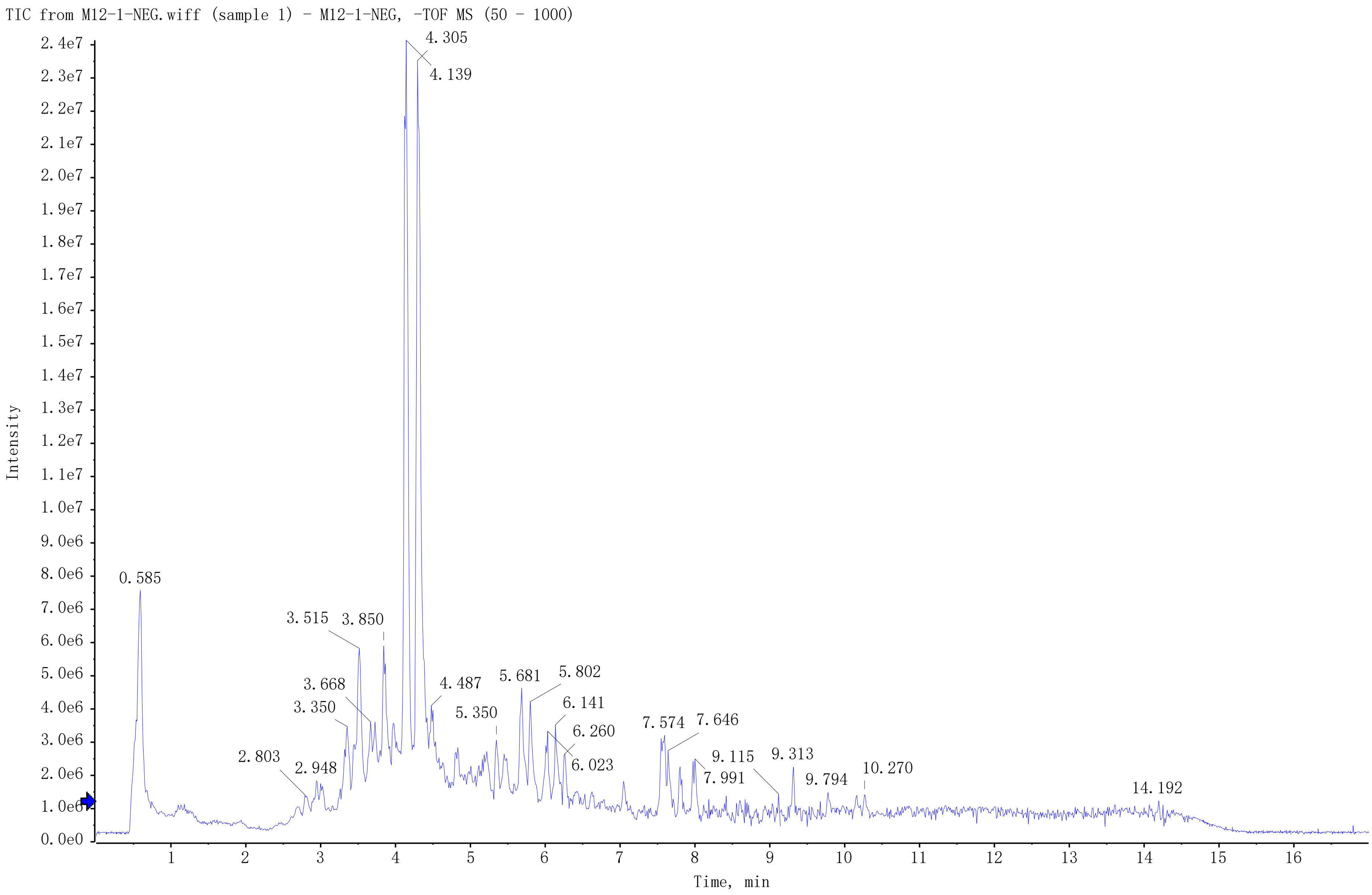
TIC from M12-1-NEG.wiff (sample 1) - M12-1-NEG, -TOF MS (50 - 1000)
Intensity
Time, min
0.585
2.803
2.948
3.350
3.515
3.668
3.850
4.305
4.139
4.487
5.350
5.681
5.802
6.141
6.260
6.023
7.574
7.646
7.991
9.115
9.313
9.794
10.270
14.192

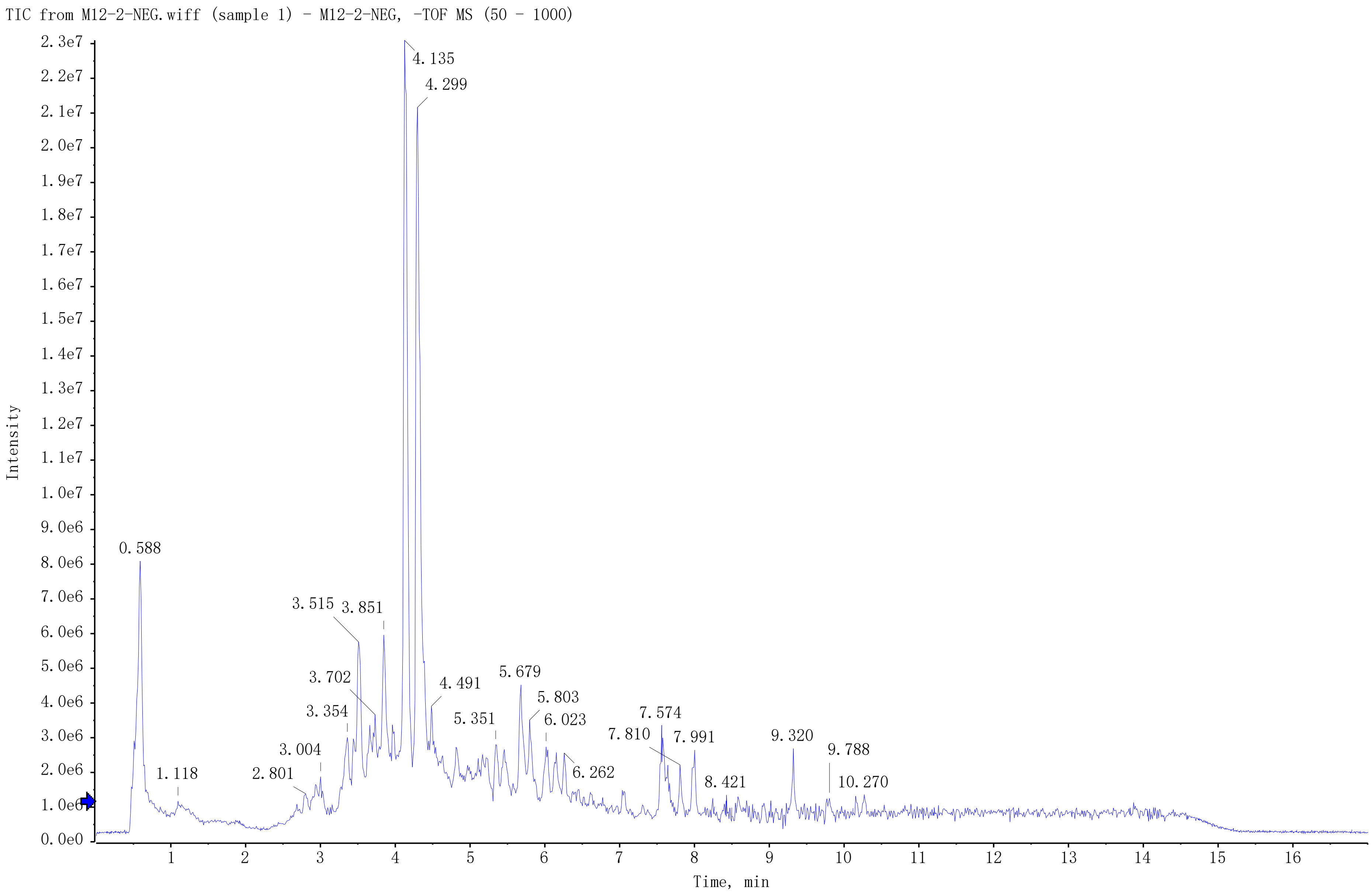

TIC from M12-2-NEG.wiff (sample 1) - M12-2-NEG, -TOF MS (50 - 1000)
Intensity
Time, min
4.135
4.299
0.588
1.118
2.801
3.004
3.354
3.515
3.702
3.851
4.491
5.351
5.679
5.803
6.023
6.262
7.574
7.810
7.991
8.421
9.320
9.788
10.270

TIC from M12-3-NEG.wiff (sample 1) - M12-3-NEG, -TOF MS (50 - 1000)

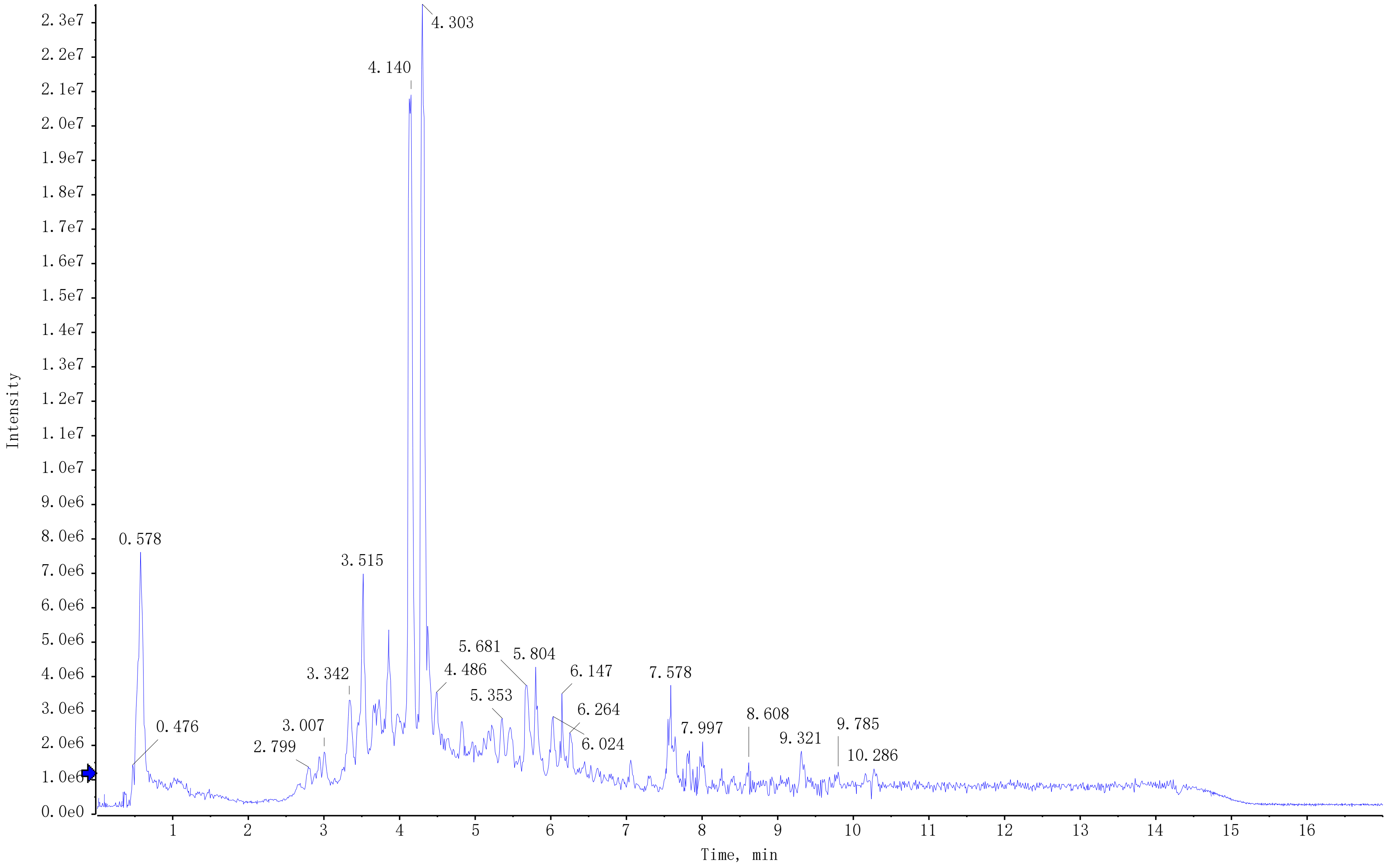

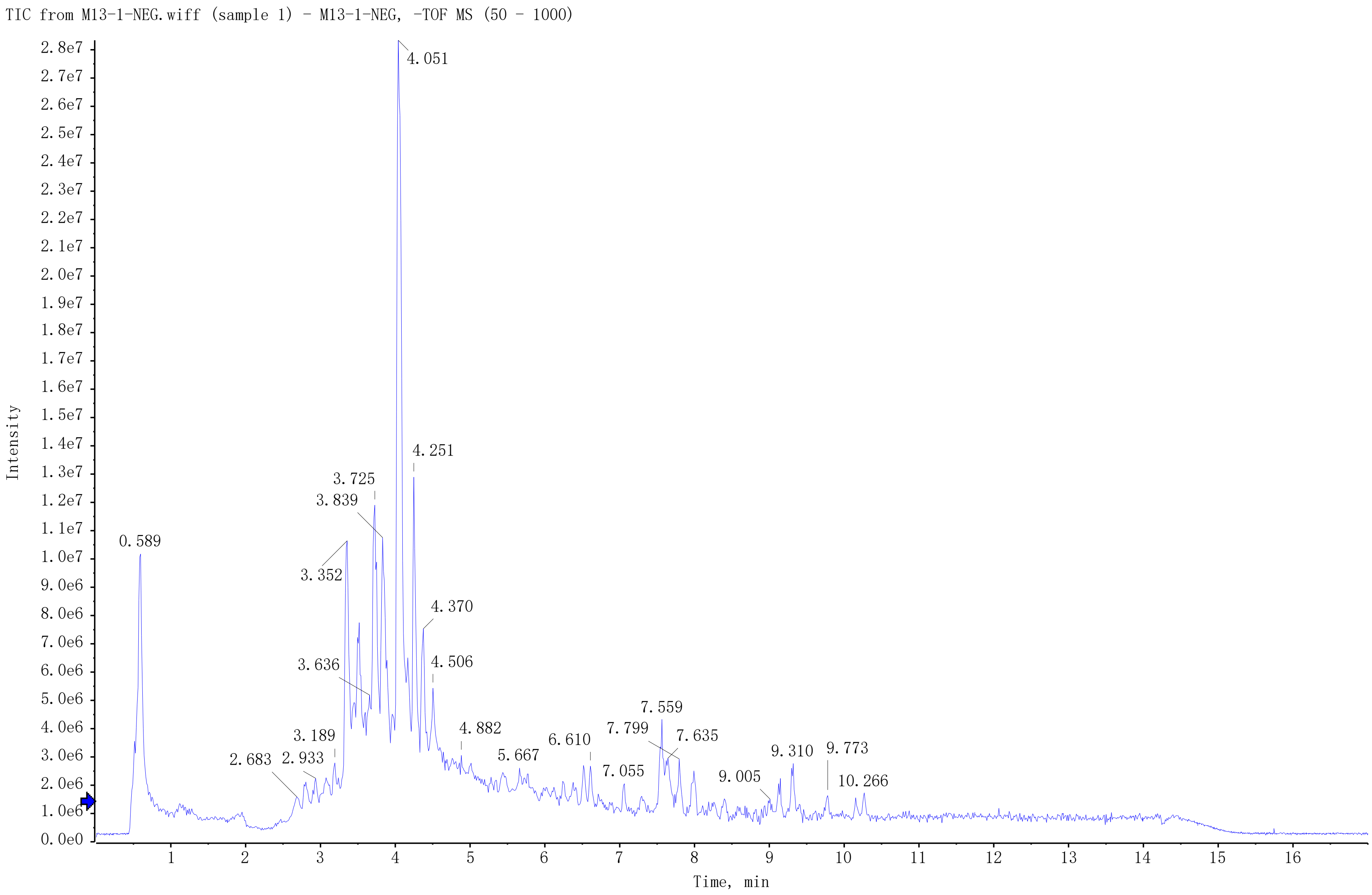
TIC from M13-1-NEG.wiff (sample 1) - M13-1-NEG, -TOF MS (50 - 1000)
Intensity
Time, min
4.051
4.251
3.725
3.839
0.589
3.352
4.370
3.636
4.506
7.559
4.882
7.799
7.635
6.610
9.310
9.773
5.667
2.683
2.933
3.189
7.055
9.005
10.266

TIC from M13-2-NEG.wiff (sample 1) - M13-2-NEG, -TOF MS (50 - 1000)

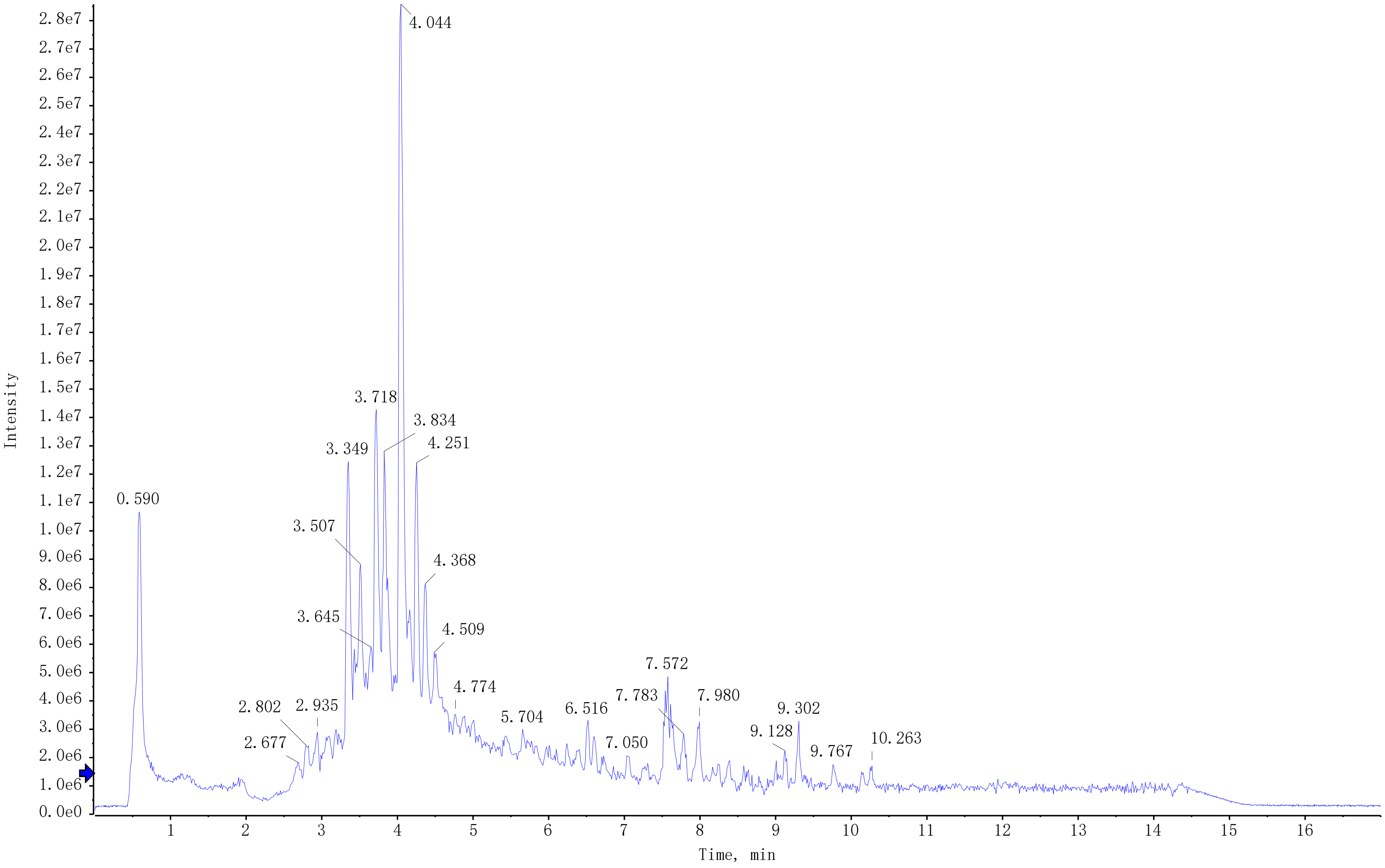

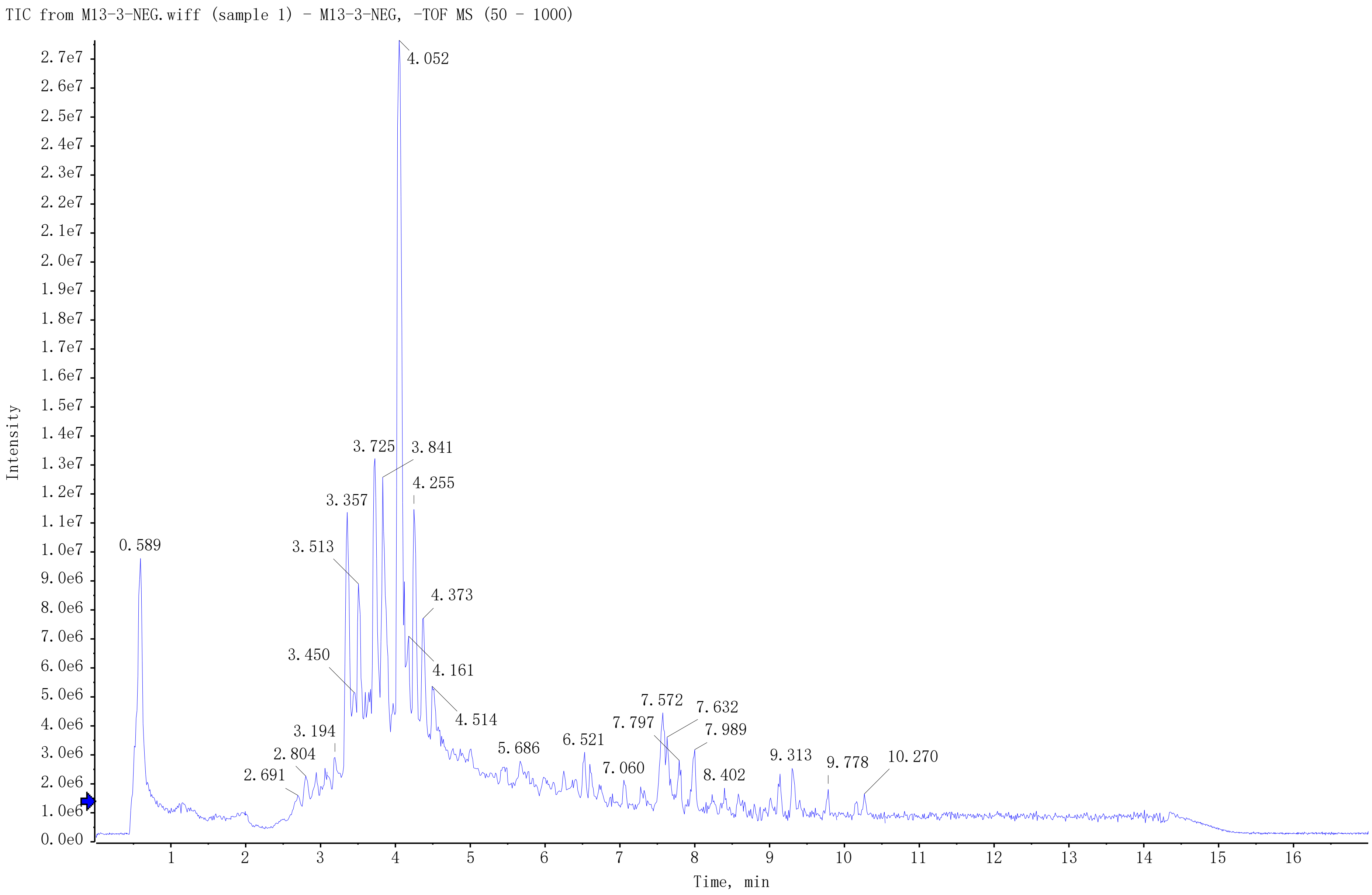
TIC from M13-3-NEG.wiff (sample 1) - M13-3-NEG, -TOF MS (50 - 1000)
Intensity
Time, min
0.589
2.691
2.804
3.194
3.357
3.450
3.513
3.725
3.841
4.052
4.161
4.255
4.373
4.514
5.686
6.521
7.060
7.572
7.632
7.797
7.989
8.402
9.313
9.778
10.270

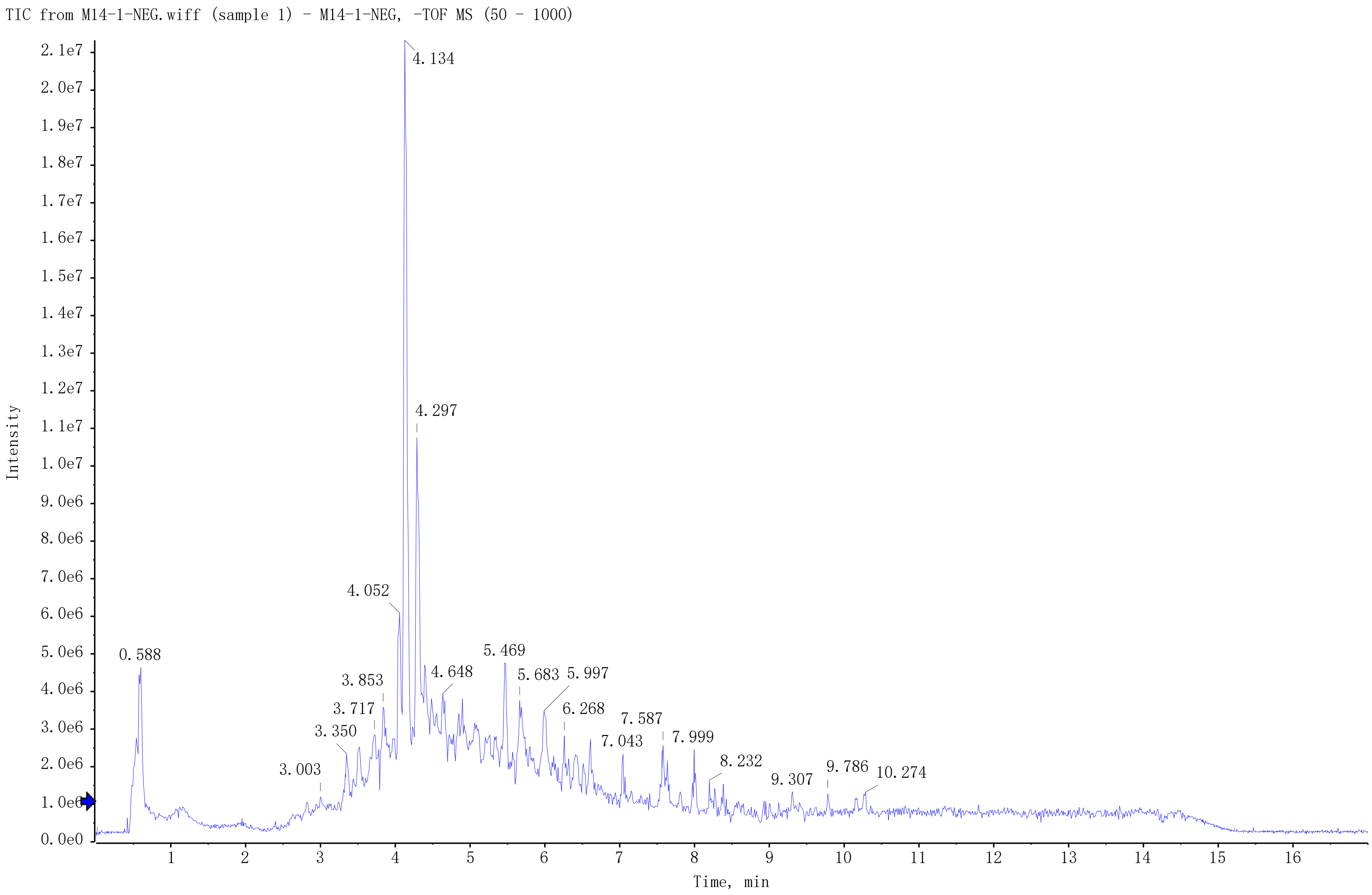
TIC from M14-1-NEG.wiff (sample 1) - M14-1-NEG, -TOF MS (50 - 1000)
Intensity
Time, min
4.134
4.297
4.052
0.588
5.469
5.683
5.997
4.648
3.853
3.717
3.350
3.003
6.268
7.587
7.043
7.999
8.232
9.307
9.786
10.274

TIC from M14-2-NEG.wiff (sample 1) - M14-2-NEG, -TOF MS (50 - 1000)

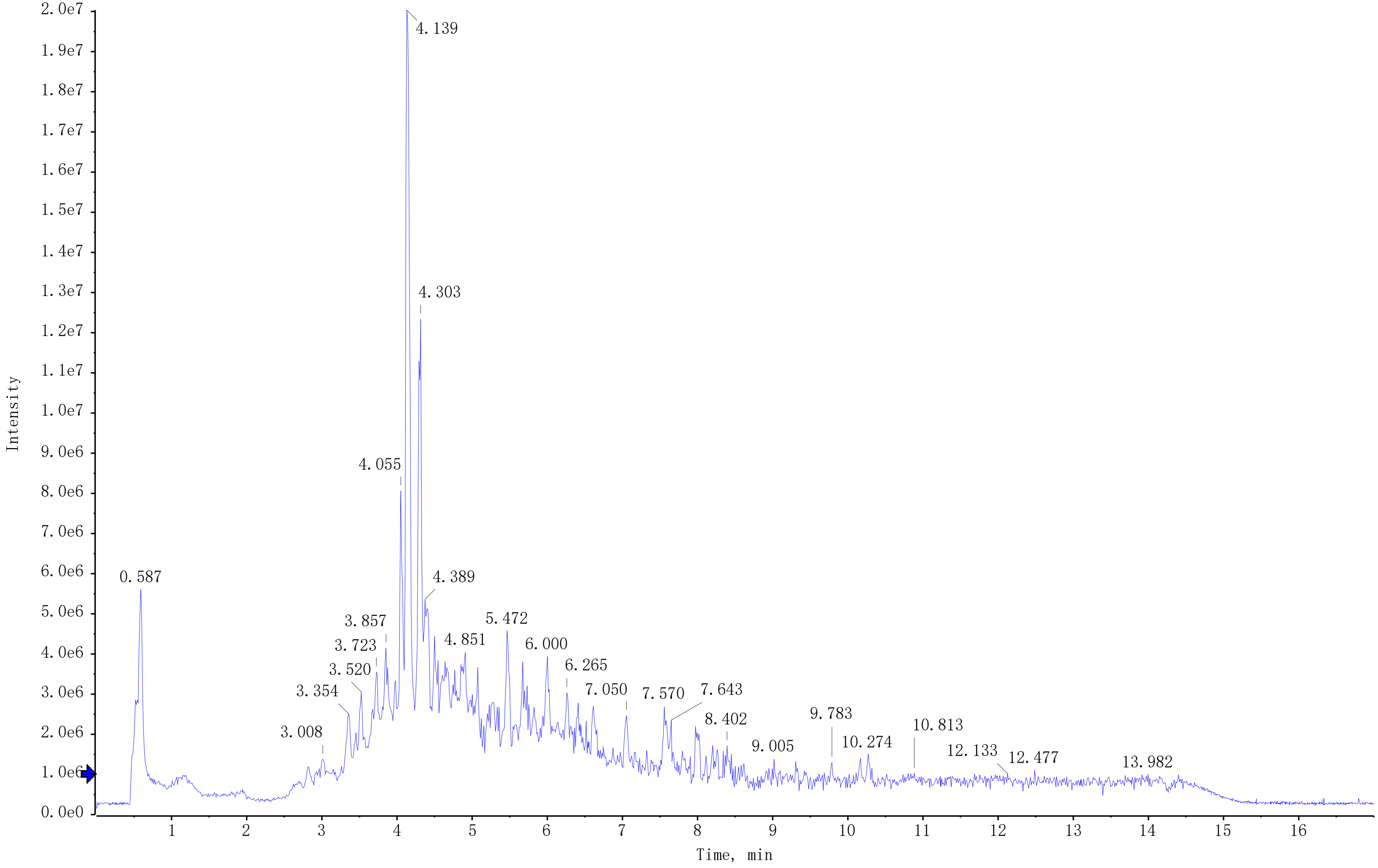

TIC from M14-3-NEG.wiff (sample 1) - M14-3-NEG, -TOF MS (50 - 1000)

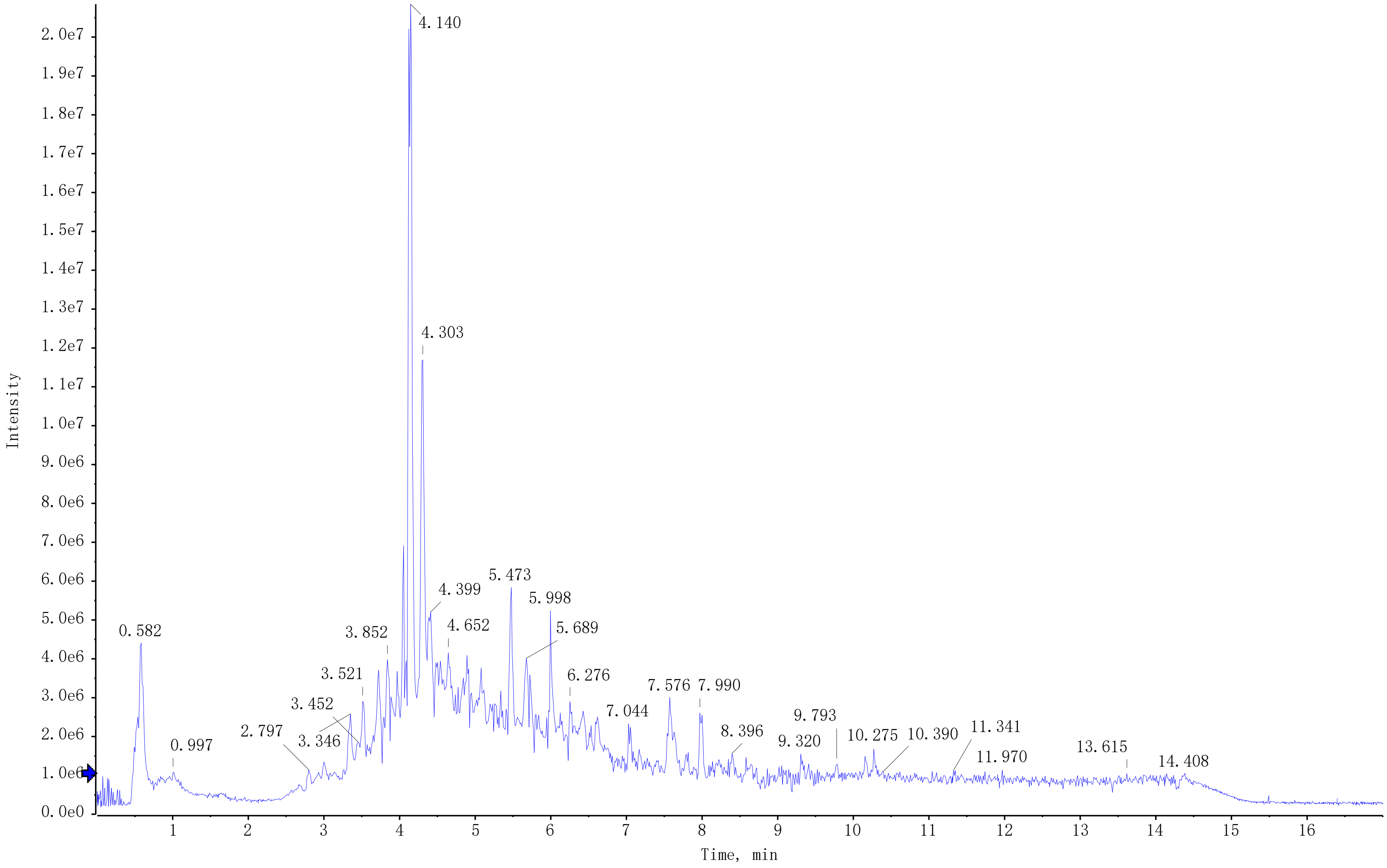

TIC from M15-1-NEG.wiff (sample 1) - M15-1-NEG, -TOF MS (50 - 1000)

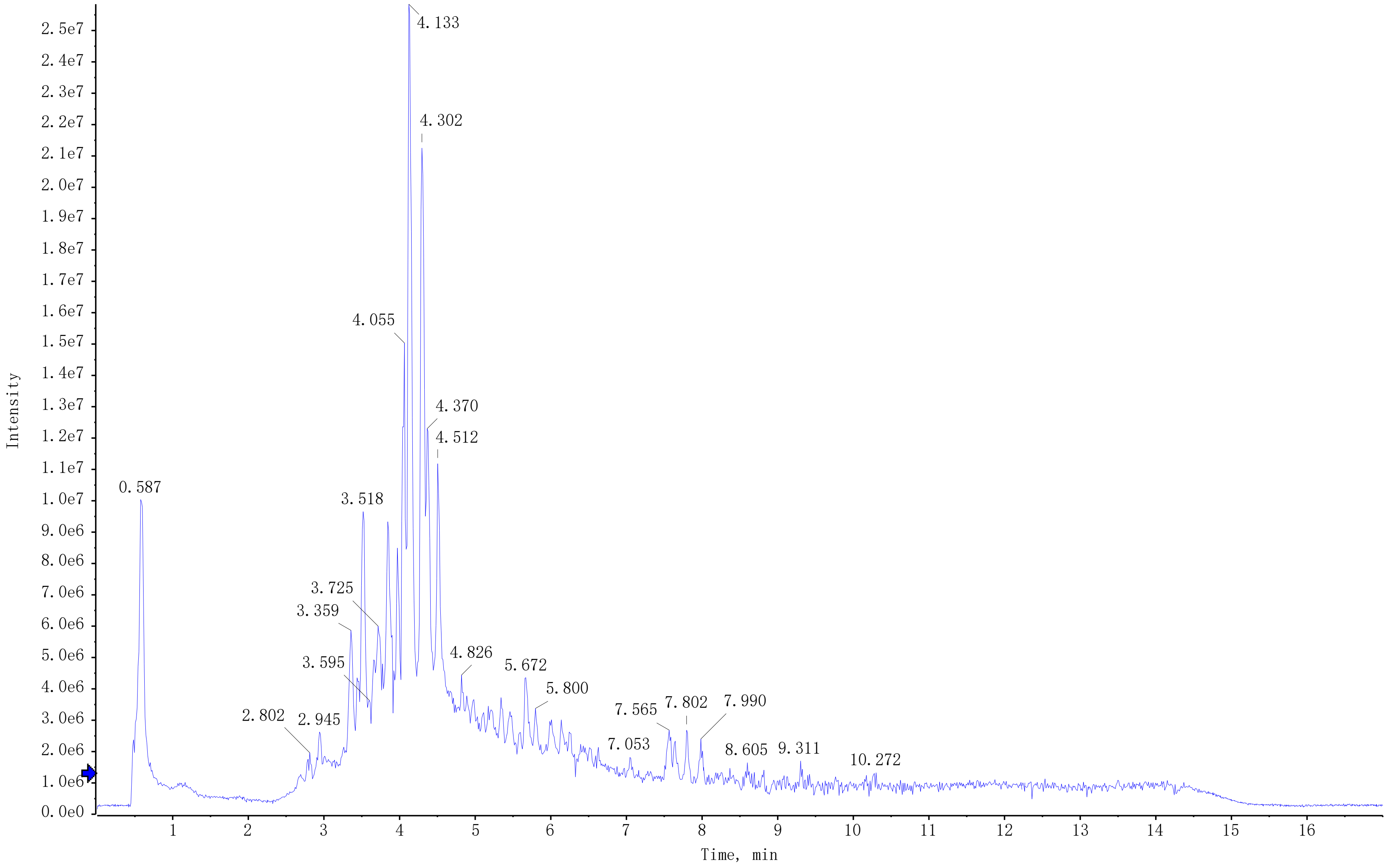

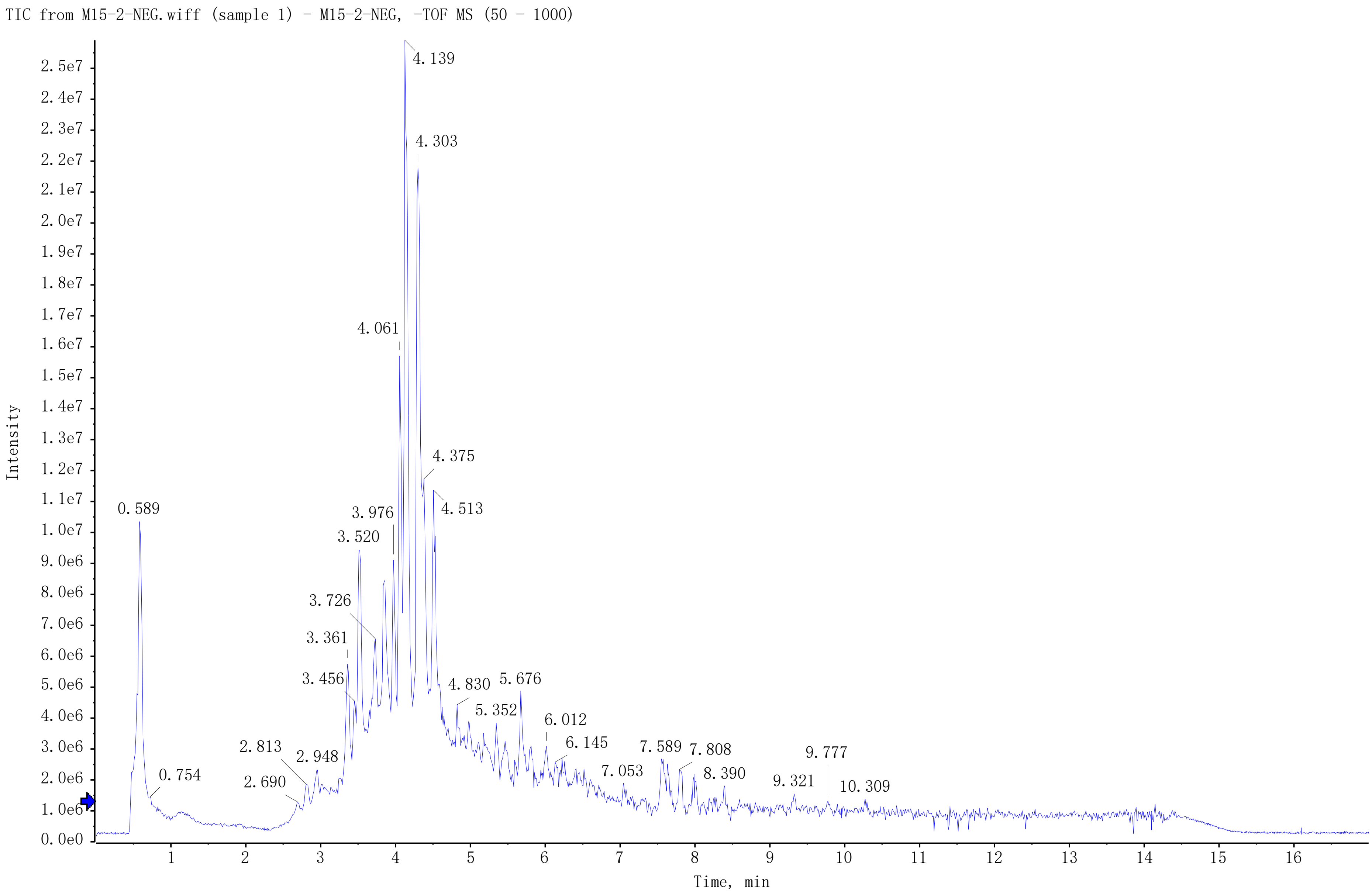

TIC from M15-2-NEG.wiff (sample 1) - M15-2-NEG, -TOF MS (50 - 1000)
Intensity
Time, min
0.589
0.754
2.690
2.813
2.948
3.361
3.456
3.520
3.726
3.976
4.061
4.139
4.303
4.375
4.513
4.830
5.352
5.676
6.012
6.145
7.053
7.589
7.808
8.390
9.321
9.777
10.309

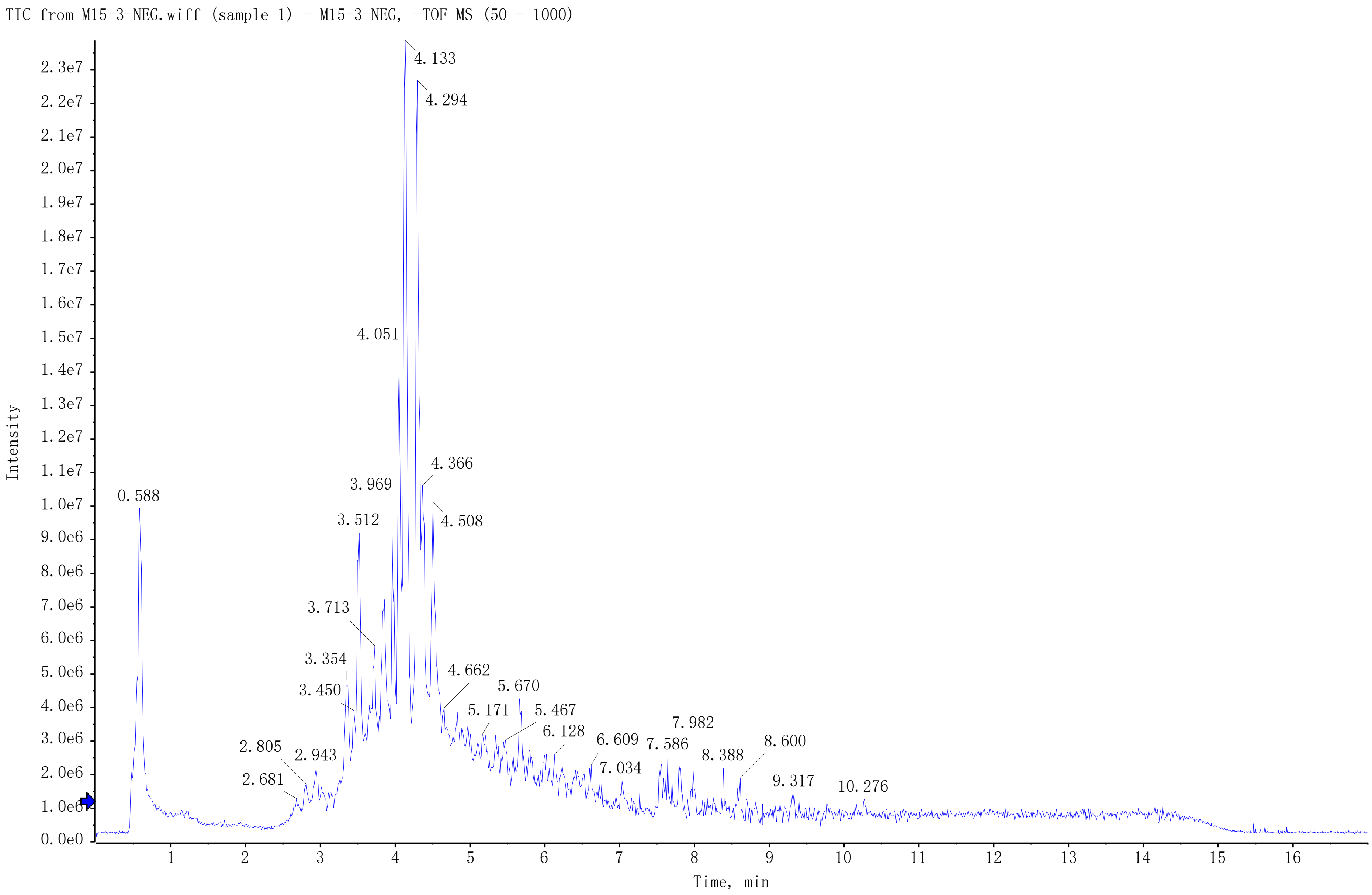

TIC from M15-3-NEG.wiff (sample 1) - M15-3-NEG, -TOF MS (50 - 1000)
Intensity
Time, min
0.588
2.681
2.805
2.943
3.354
3.450
3.512
3.713
3.969
4.051
4.133
4.294
4.366
4.508
4.662
5.171
5.467
5.670
6.128
6.609
7.034
7.586
7.982
8.388
8.600
9.317
10.276

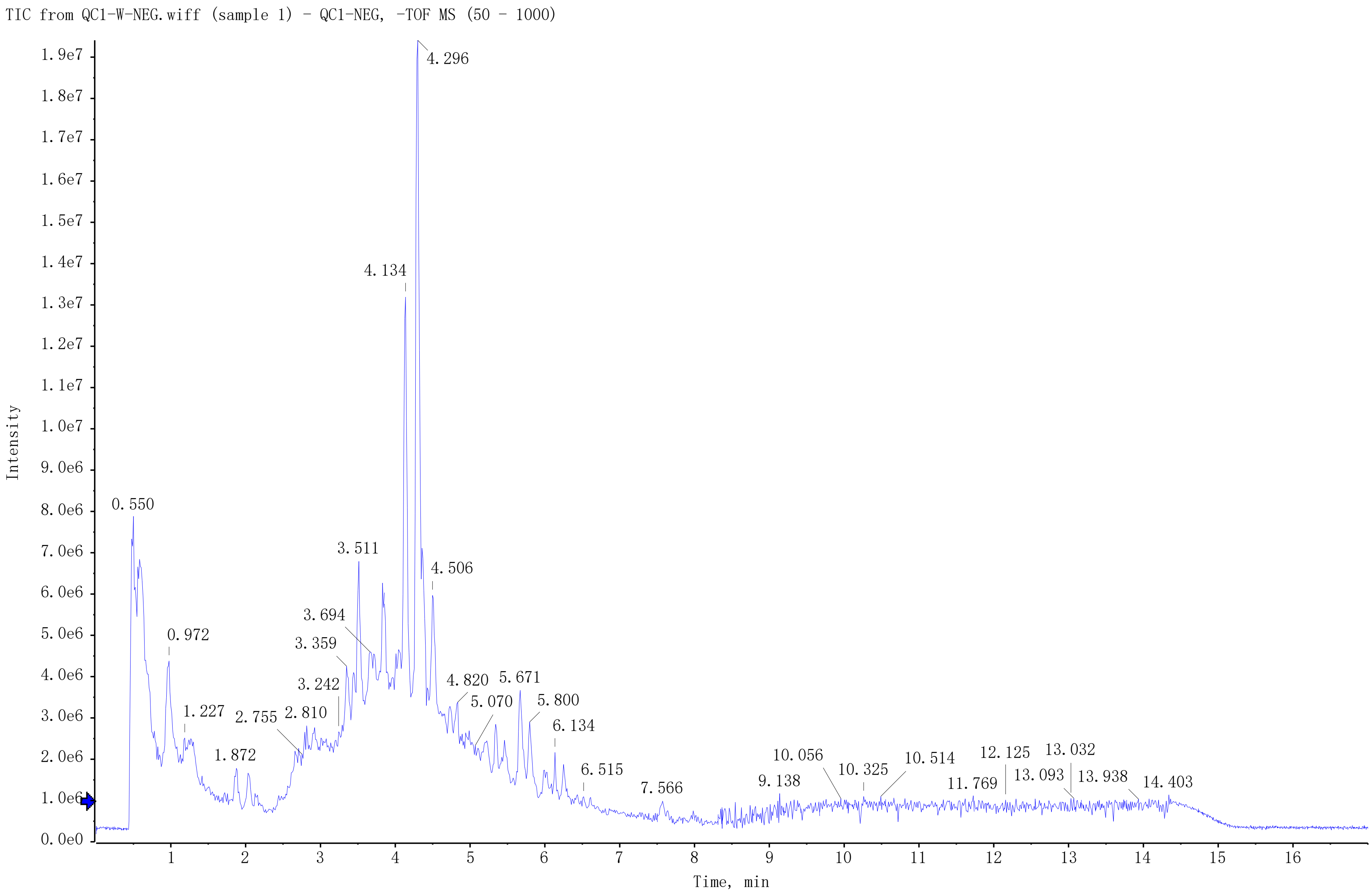
TIC from QC1-W-NEG.wiff (sample 1) - QC1-NEG, -TOF MS (50 - 1000)
Intensity
Time, min
4.296
4.134
0.550
3.511
4.506
3.694
0.972
3.359
3.242
4.820
5.671
5.070
5.800
1.227
2.755
2.810
6.134
1.872
6.515
7.566
9.138
10.056
10.325
10.514
11.769
12.125
13.032
13.093
13.938
14.403

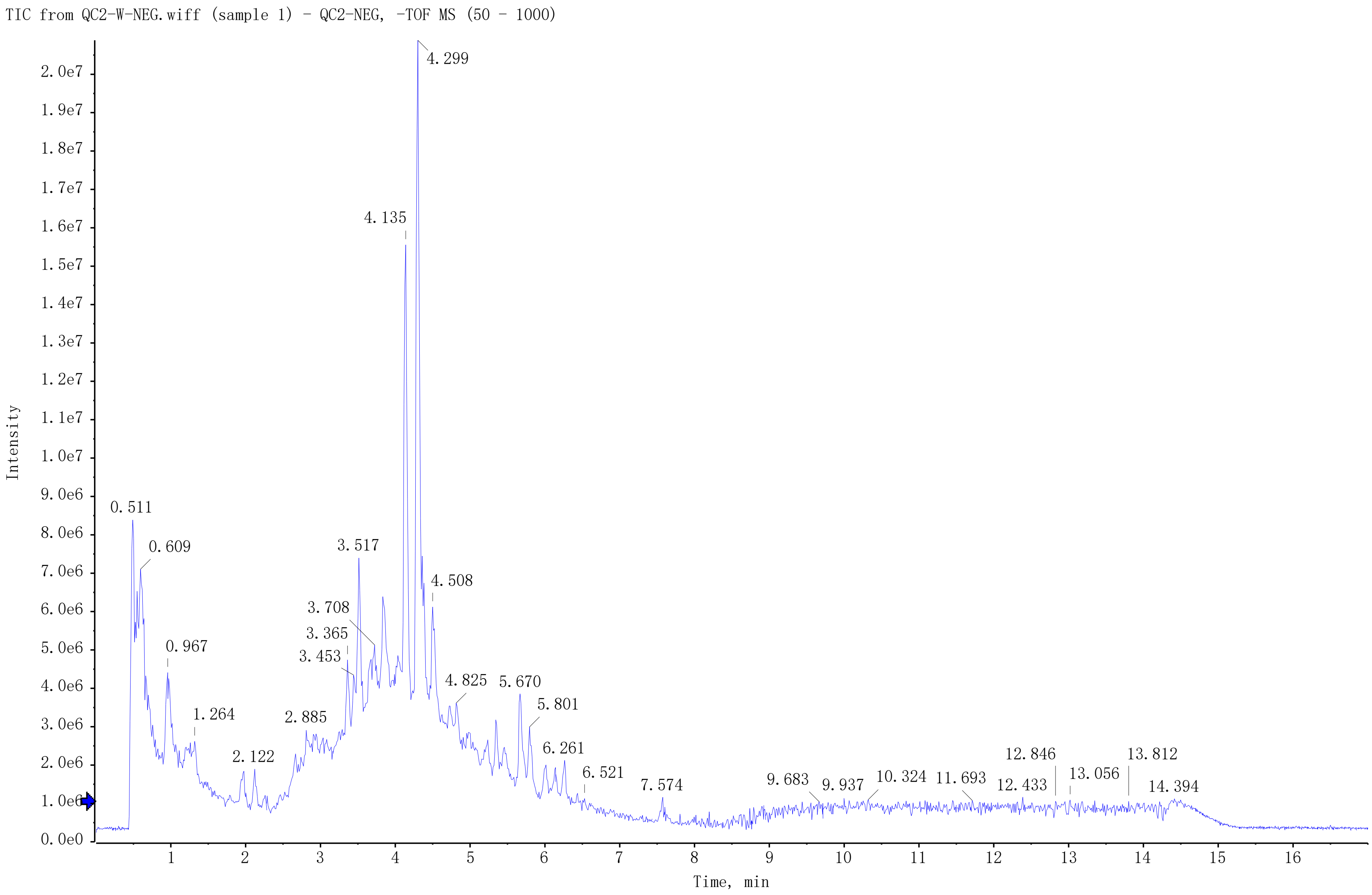
TIC from QC2-W-NEG.wiff (sample 1) - QC2-NEG, -TOF MS (50 - 1000)
Intensity
Time, min
2.0e7
1.9e7
1.8e7
1.7e7
1.6e7
1.5e7
1.4e7
1.3e7
1.2e7
1.1e7
1.0e7
9.0e6
8.0e6
7.0e6
6.0e6
5.0e6
4.0e6
3.0e6
2.0e6
1.0e6
0.0e0
1
2
3
4
5
6
7
8
9
10
11
12
13
14
15
16
4.299
4.135
0.511
0.609
3.517
4.508
3.708
3.365
3.453
0.967
4.825
5.670
5.801
1.264
2.885
2.122
6.261
6.521
7.574
9.683
9.937
10.324
11.693
12.846
12.433
13.056
13.812
14.394

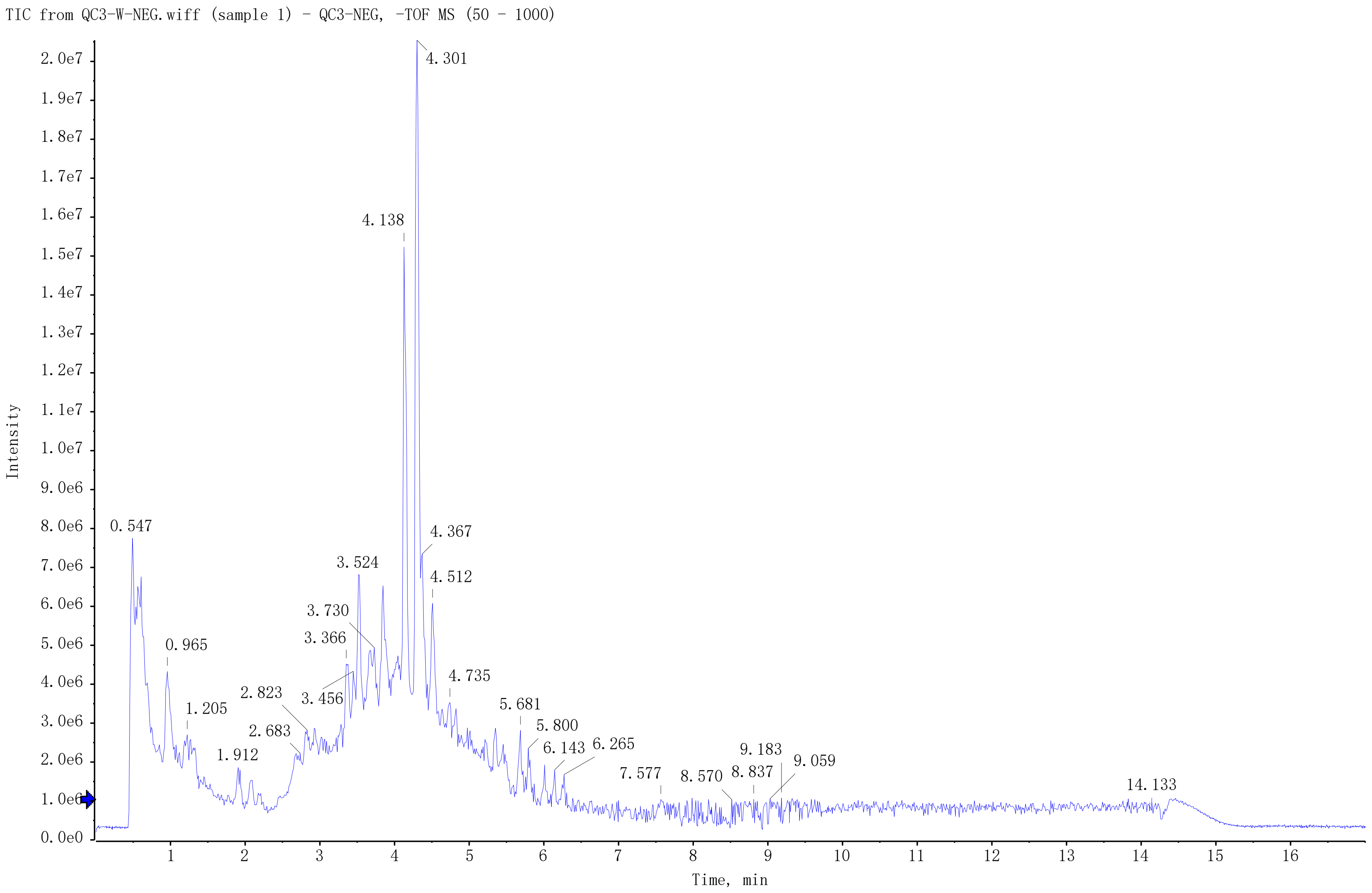
TIC from QC3-W-NEG.wiff (sample 1) - QC3-NEG, -TOF MS (50 - 1000)
Intensity
Time, min
0.547
0.965
1.205
1.912
2.683
2.823
3.366
3.456
3.524
3.730
4.138
4.301
4.367
4.512
4.735
5.681
5.800
6.143
6.265
7.577
8.570
8.837
9.183
9.059
14.133

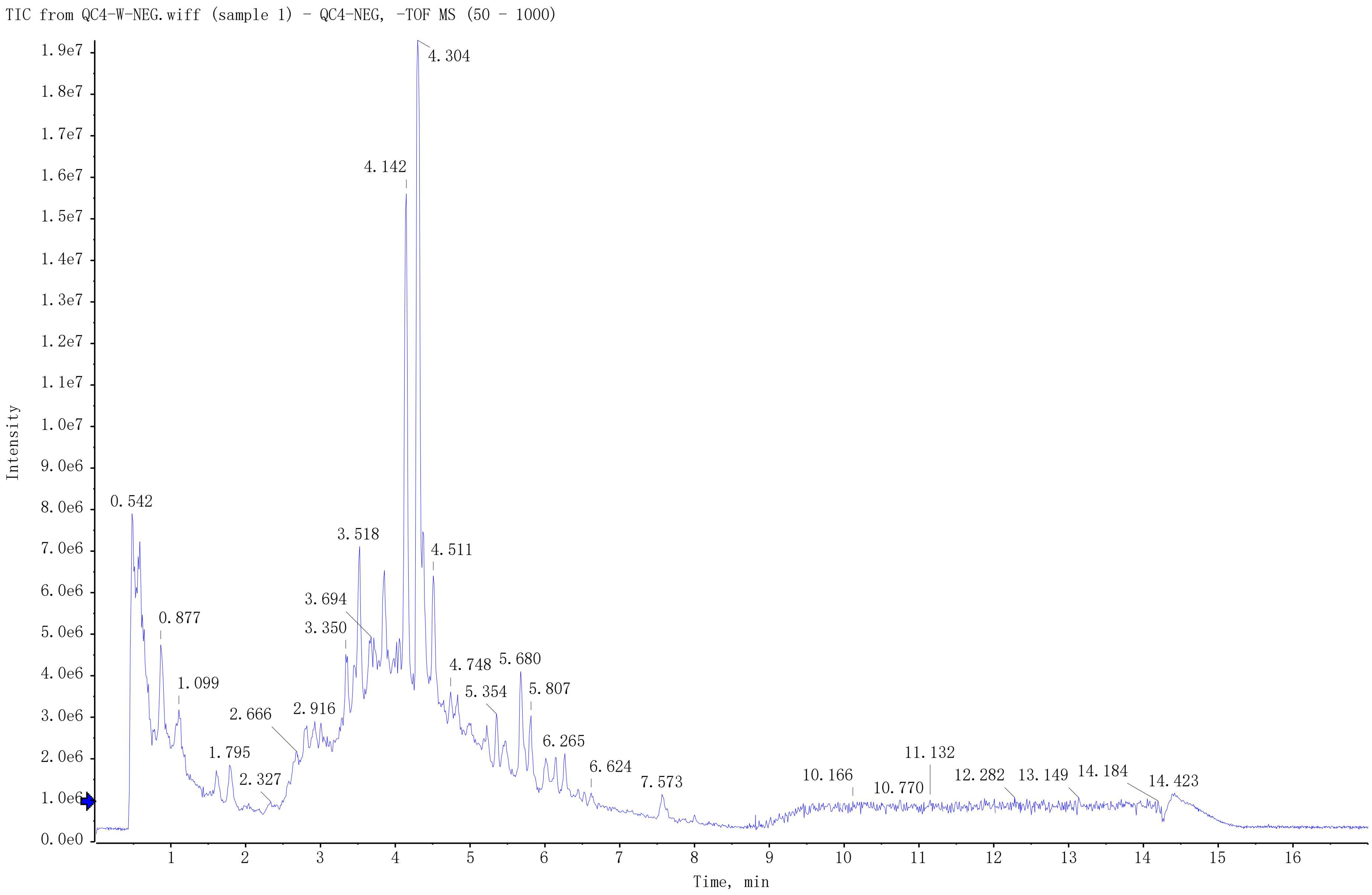

TIC from QC4-W-NEG.wiff (sample 1) - QC4-NEG, -TOF MS (50 - 1000)
Intensity
1.9e7
1.8e7
1.7e7
1.6e7
1.5e7
1.4e7
1.3e7
1.2e7
1.1e7
1.0e7
9.0e6
8.0e6
7.0e6
6.0e6
5.0e6
4.0e6
3.0e6
2.0e6
1.0e6
0.0e0
4.304
4.142
0.542
3.518
4.511
3.694
0.877
3.350
5.680
4.748
1.099
5.354
5.807
2.916
2.666
6.265
1.795
6.624
11.132
2.327
7.573
10.166
12.282
13.149
14.184
14.423
10.770
1
2
3
4
5
6
7
8
9
10
11
12
13
14
15
16
Time, min

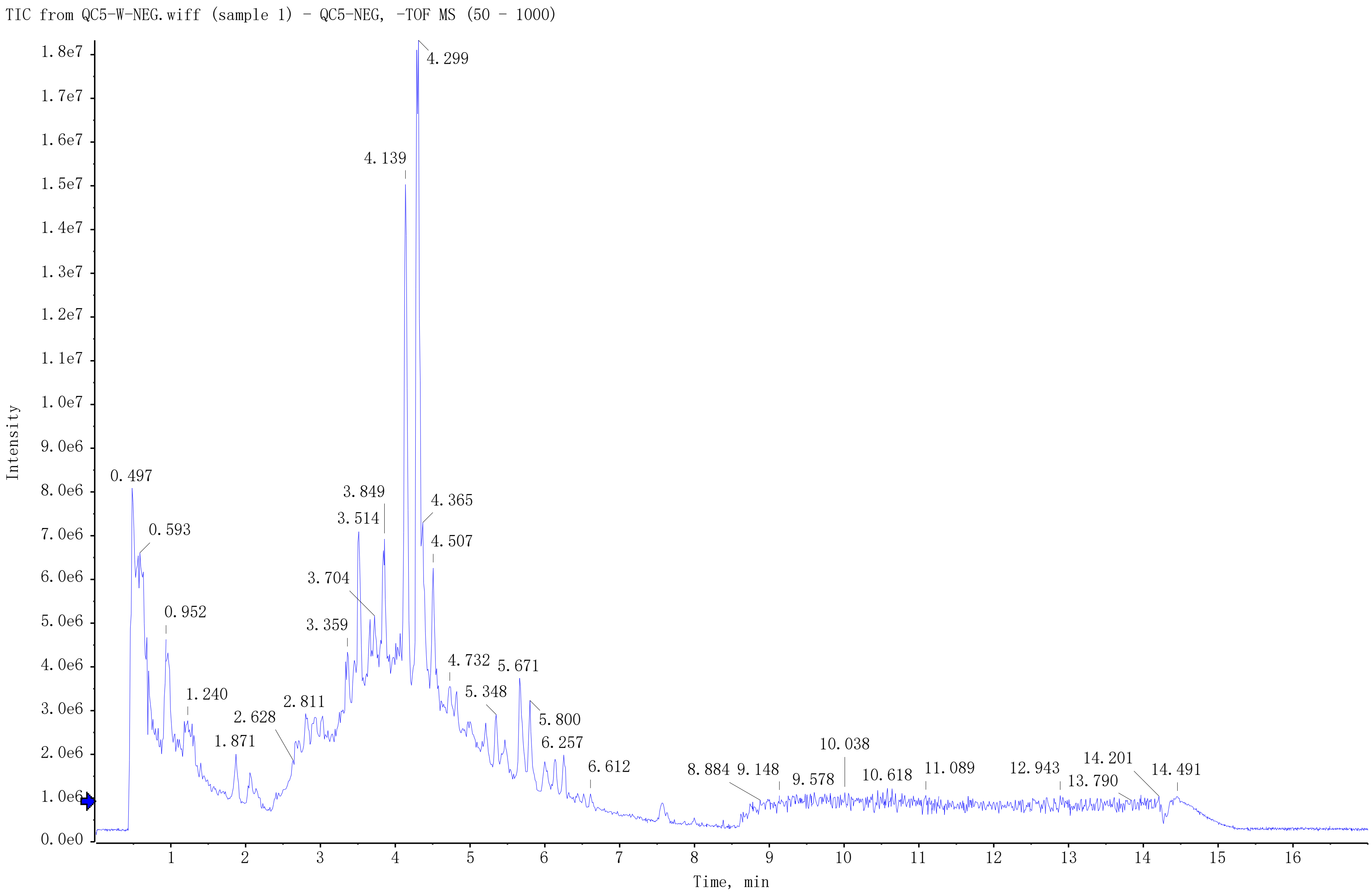
TIC from QC5-W-NEG.wiff (sample 1) - QC5-NEG, -TOF MS (50 - 1000)
Intensity
Time, min
1.8e7
1.7e7
1.6e7
1.5e7
1.4e7
1.3e7
1.2e7
1.1e7
1.0e7
9.0e6
8.0e6
7.0e6
6.0e6
5.0e6
4.0e6
3.0e6
2.0e6
1.0e6
0.0e0
1
2
3
4
5
6
7
8
9
10
11
12
13
14
15
16
4.299
4.139
0.497
0.593
0.952
1.240
1.871
2.628
2.811
3.359
3.514
3.704
3.849
4.365
4.507
4.732
5.348
5.671
5.800
6.257
6.612
8.884
9.148
9.578
10.038
10.618
11.089
12.943
13.790
14.201
14.491

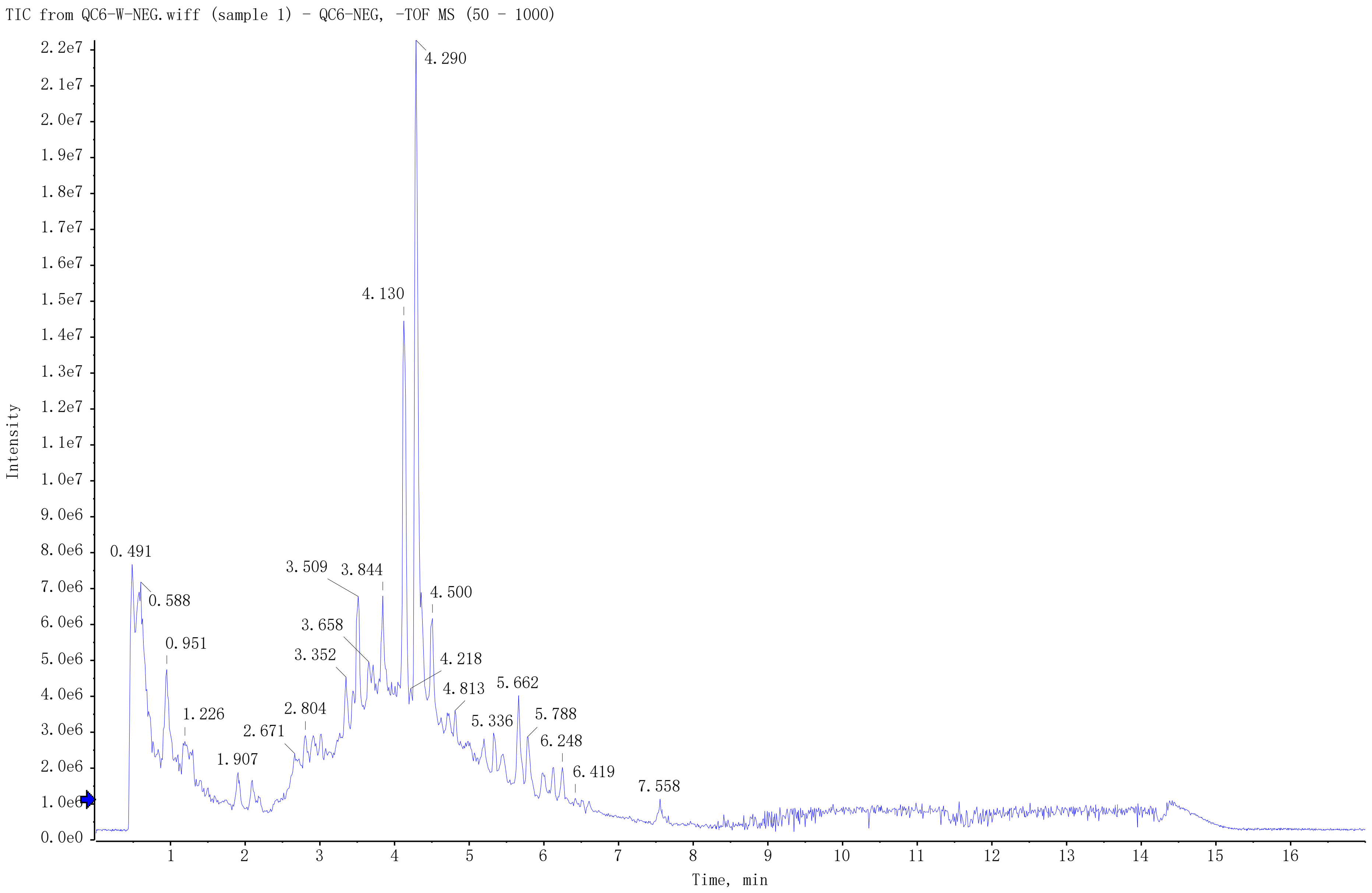

TIC from QC6-W-NEG.wiff (sample 1) - QC6-NEG, -TOF MS (50 - 1000)
Intensity
Time, min
4.290
4.130
0.491
0.588
0.951
1.226
1.907
2.671
2.804
3.352
3.509
3.658
3.844
4.218
4.500
4.813
5.336
5.662
5.788
6.248
6.419
7.558

TIC from M1-1-POS.wiff (sample 1) - M1-1-POS, +TOF MS (50 - 1000)

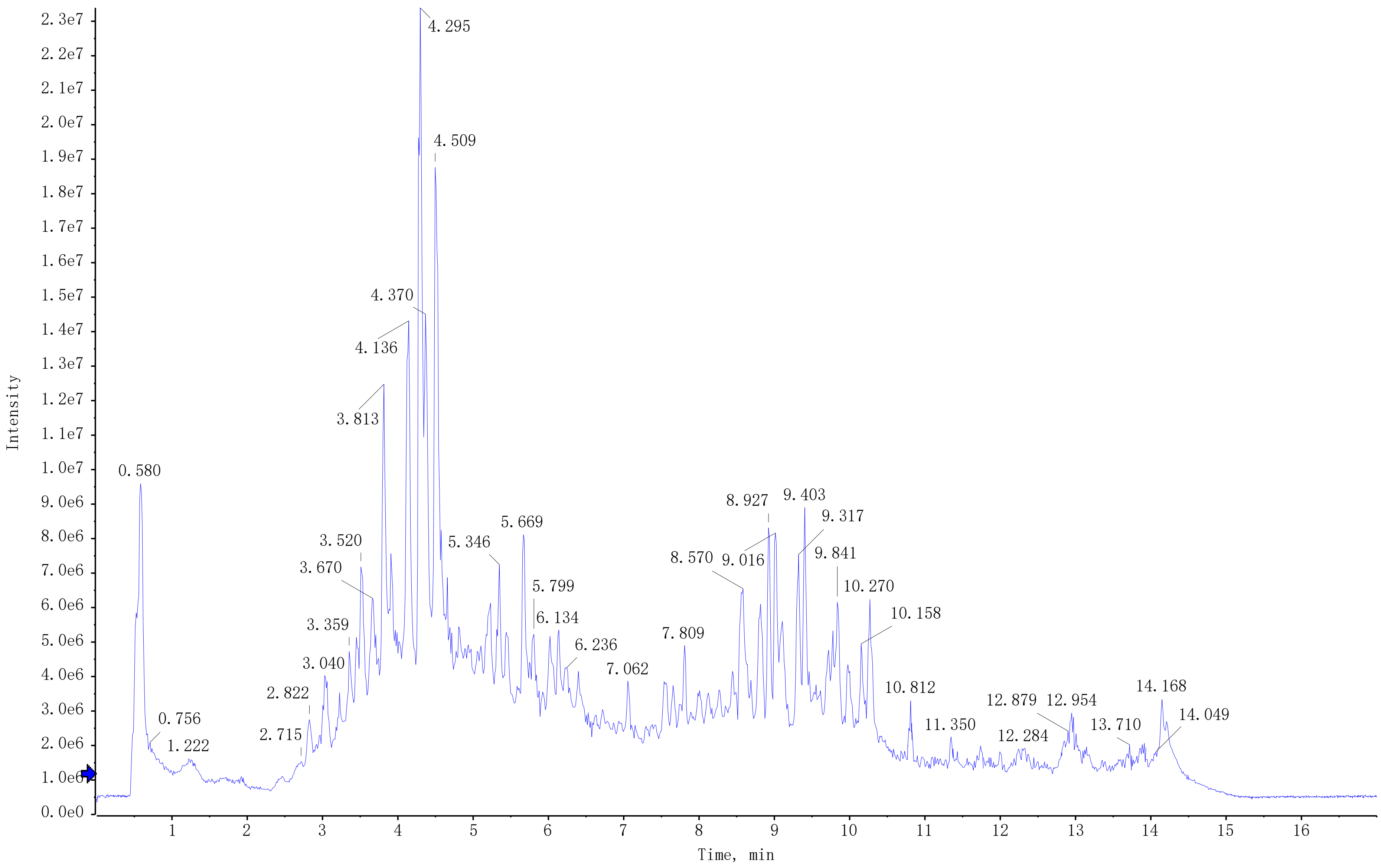

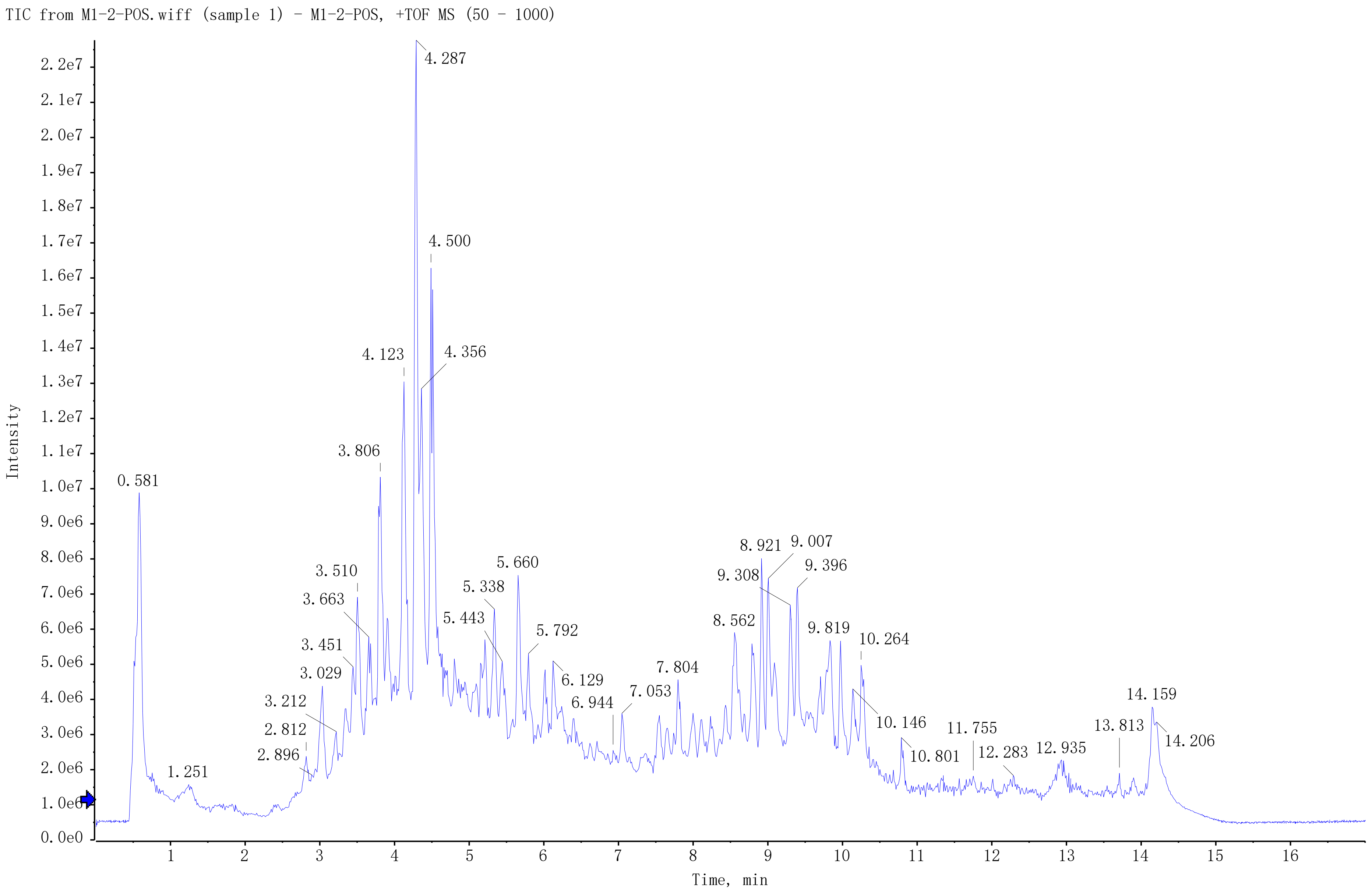
TIC from M1-2-POS.wiff (sample 1) - M1-2-POS, +TOF MS (50 - 1000)
Intensity
Time, min
0.581
1.251
2.812
2.896
3.029
3.212
3.451
3.510
3.663
3.806
4.123
4.287
4.356
4.500
5.338
5.443
5.660
5.792
6.129
6.944
7.053
7.804
8.562
8.921
9.007
9.308
9.396
9.819
10.264
10.146
10.801
11.755
12.283
12.935
13.813
14.159
14.206

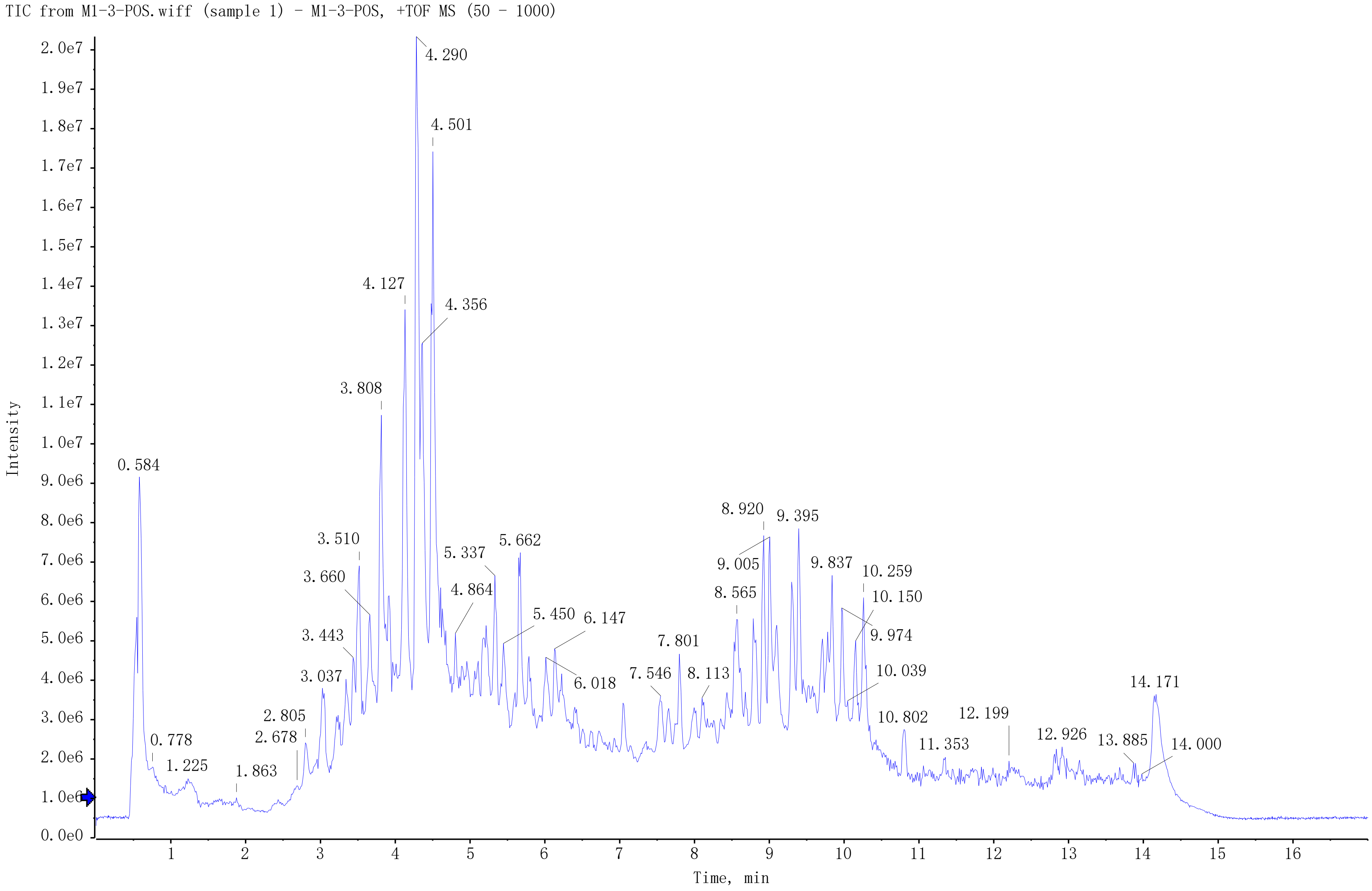
TIC from M1-3-POS.wiff (sample 1) - M1-3-POS, +TOF MS (50 - 1000)
Intensity
Time, min
0.584
0.778
1.225
1.863
2.678
2.805
3.037
3.443
3.510
3.660
3.808
4.127
4.290
4.356
4.501
4.864
5.337
5.450
5.662
6.018
6.147
7.546
7.801
8.113
8.565
8.920
9.005
9.395
9.837
9.974
10.039
10.150
10.259
10.802
11.353
12.199
12.926
13.885
14.000
14.171

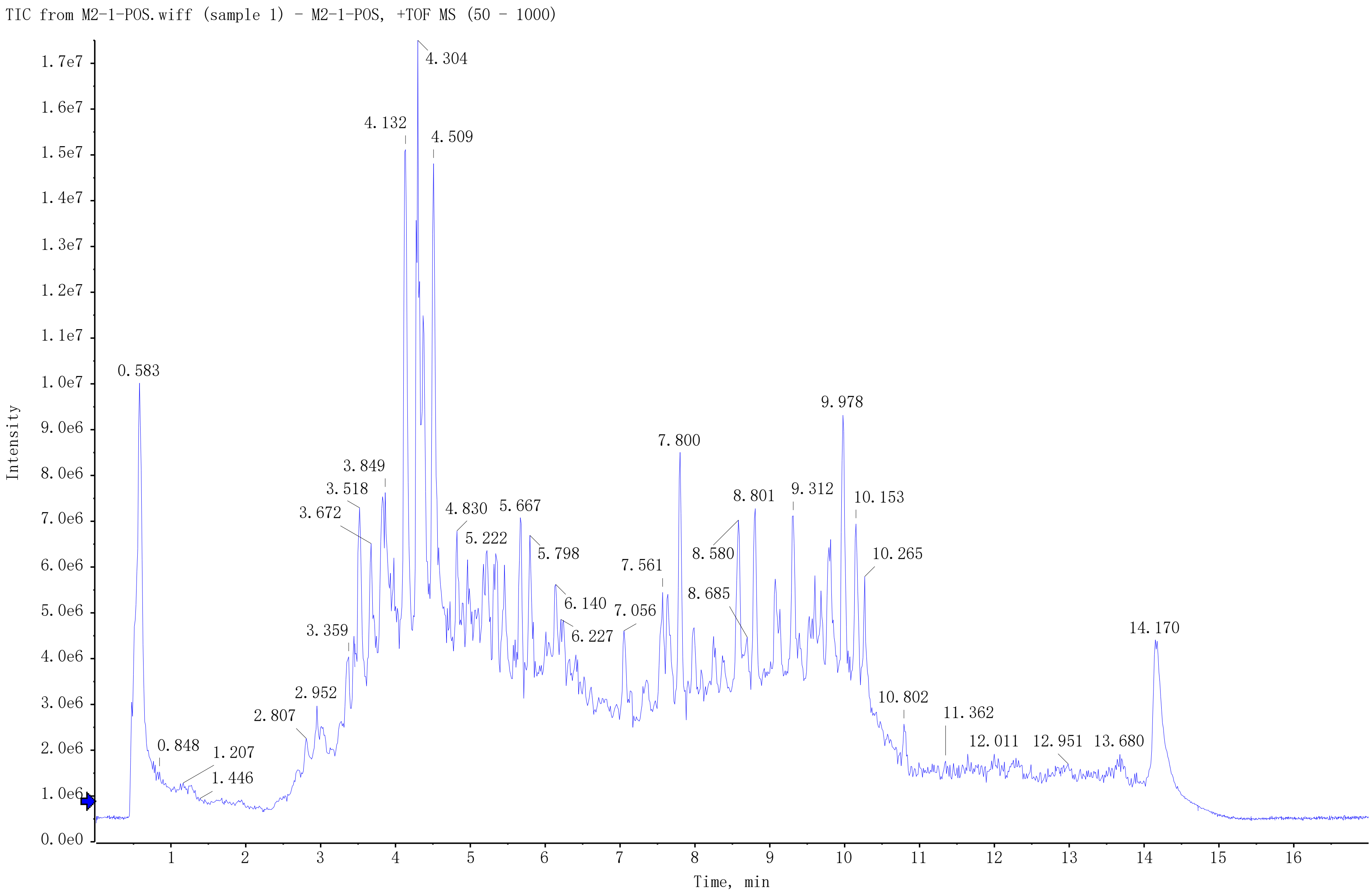
TIC from M2-1-POS.wiff (sample 1) - M2-1-POS, +TOF MS (50 - 1000)
Intensity
Time, min
0.583
0.848
1.207
1.446
2.807
2.952
3.359
3.518
3.672
3.849
4.132
4.304
4.509
4.830
5.222
5.667
5.798
6.140
6.227
7.056
7.561
7.800
8.580
8.685
8.801
9.312
9.978
10.153
10.265
10.802
11.362
12.011
12.951
13.680
14.170

TIC from M2-2-POS.wiff (sample 1) - M2-2-POS, +TOF MS (50 - 1000)

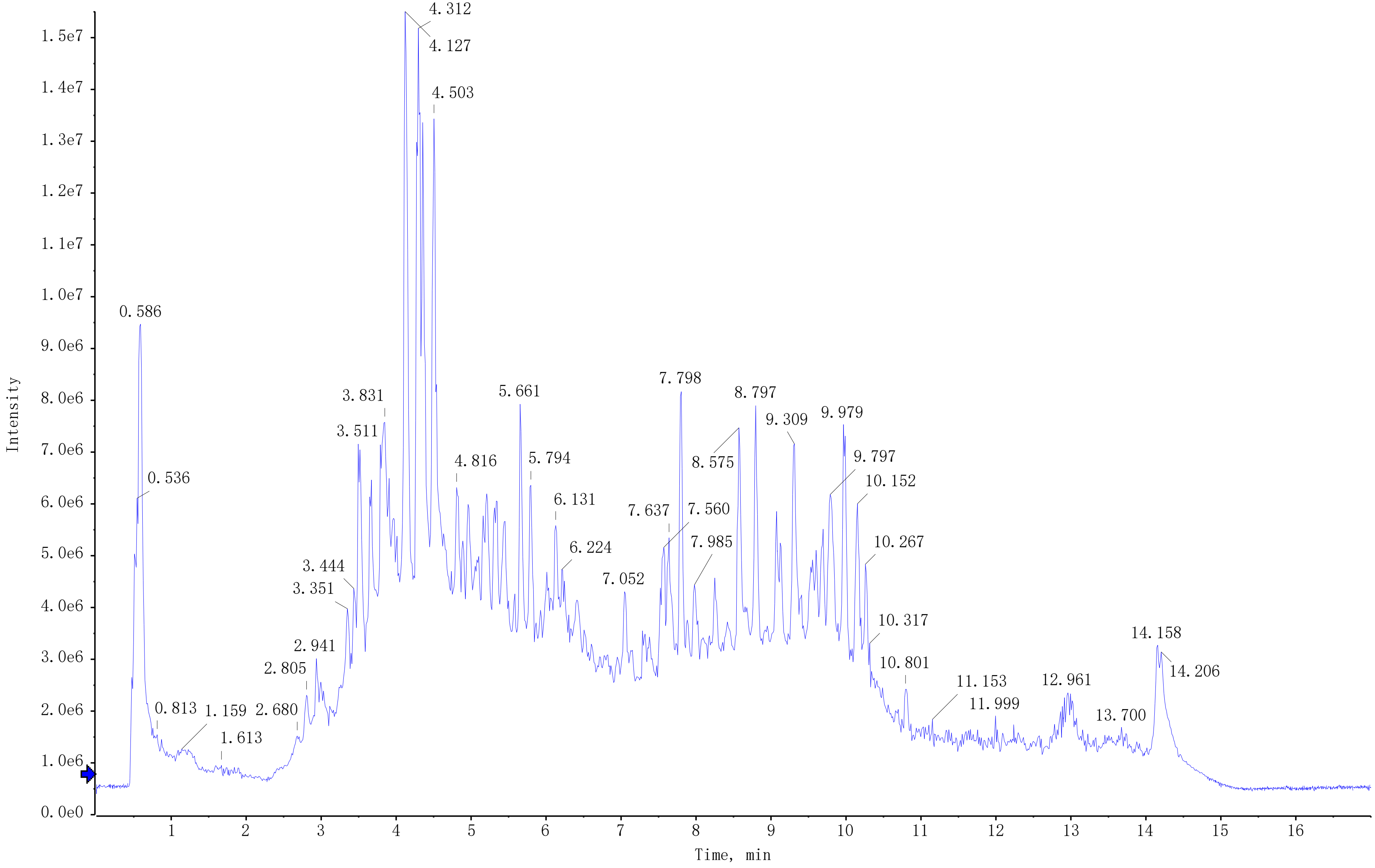

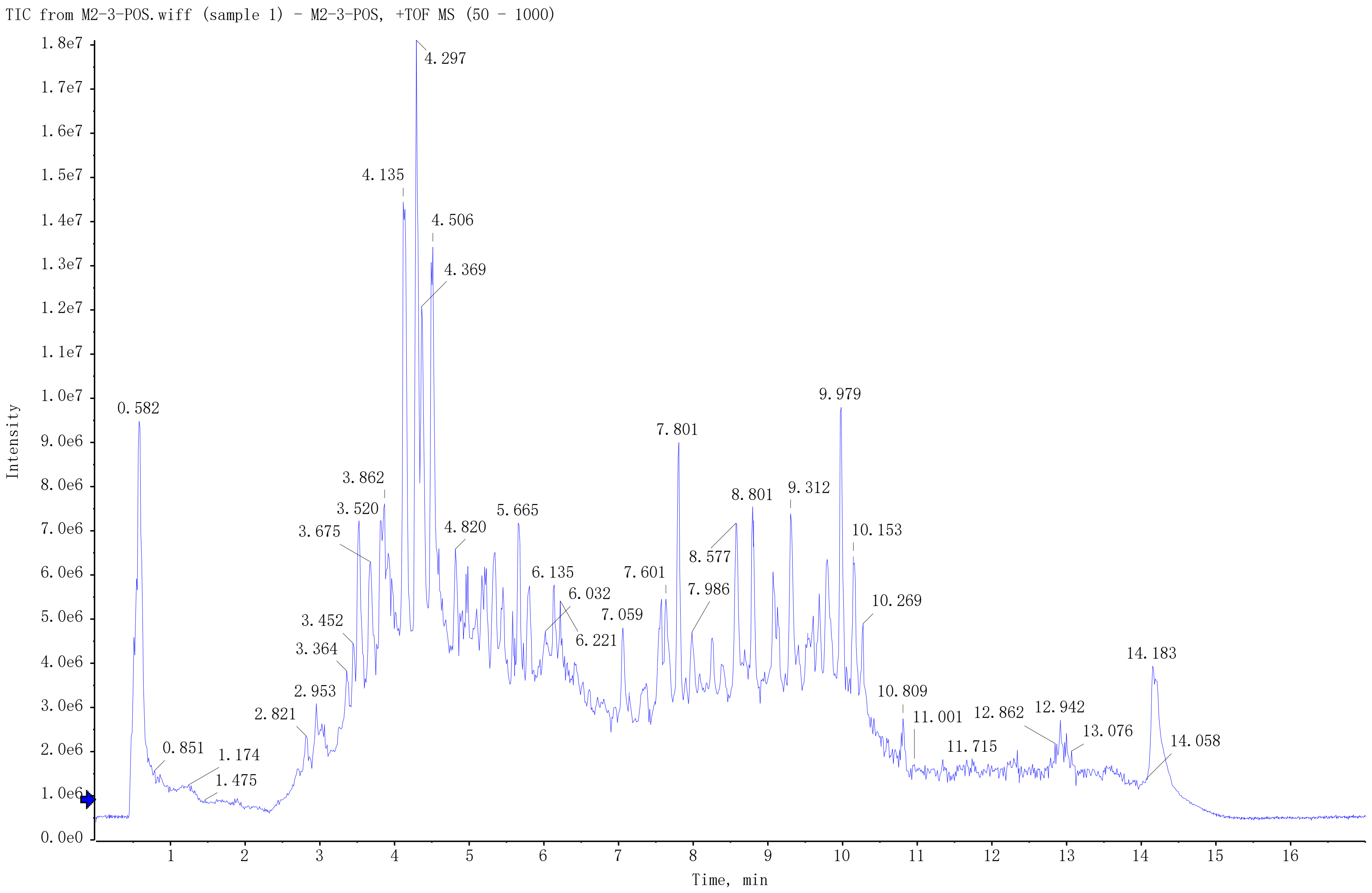

TIC from M2-3-POS.wiff (sample 1) - M2-3-POS, +TOF MS (50 - 1000)
Intensity
Time, min
0.582
0.851
1.174
1.475
2.821
2.953
3.364
3.452
3.520
3.675
3.862
4.135
4.297
4.369
4.506
4.820
5.665
6.032
6.135
6.221
7.059
7.601
7.801
7.986
8.577
8.801
9.312
9.979
10.153
10.269
10.809
11.001
11.715
12.862
12.942
13.076
14.058
14.183

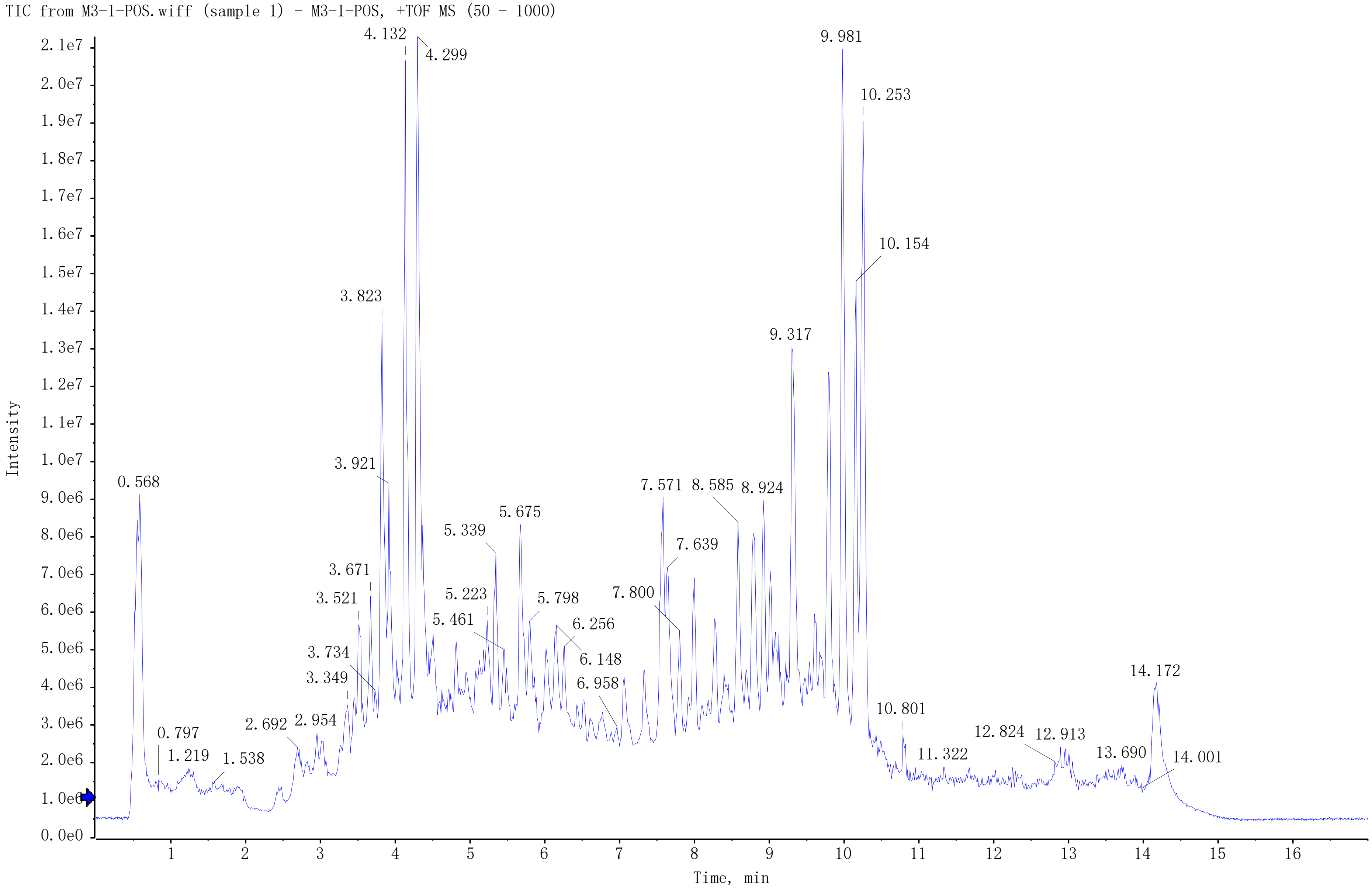

TIC from M3-1-POS.wiff (sample 1) - M3-1-POS, +TOF MS (50 - 1000)
Intensity
Time, min
2.1e7
2.0e7
1.9e7
1.8e7
1.7e7
1.6e7
1.5e7
1.4e7
1.3e7
1.2e7
1.1e7
1.0e7
9.0e6
8.0e6
7.0e6
6.0e6
5.0e6
4.0e6
3.0e6
2.0e6
1.0e6
0.0e0
1
2
3
4
5
6
7
8
9
10
11
12
13
14
15
16
0.568
0.797
1.219
1.538
2.692
2.954
3.349
3.521
3.671
3.734
3.823
3.921
4.132
4.299
5.223
5.339
5.461
5.675
5.798
6.148
6.256
6.958
7.571
7.639
7.800
8.585
8.924
9.317
9.981
10.154
10.253
10.801
11.322
12.824
12.913
13.690
14.001
14.172

TIC from M3-2-POS.wiff (sample 1) - M3-2-POS, +TOF MS (50 - 1000)

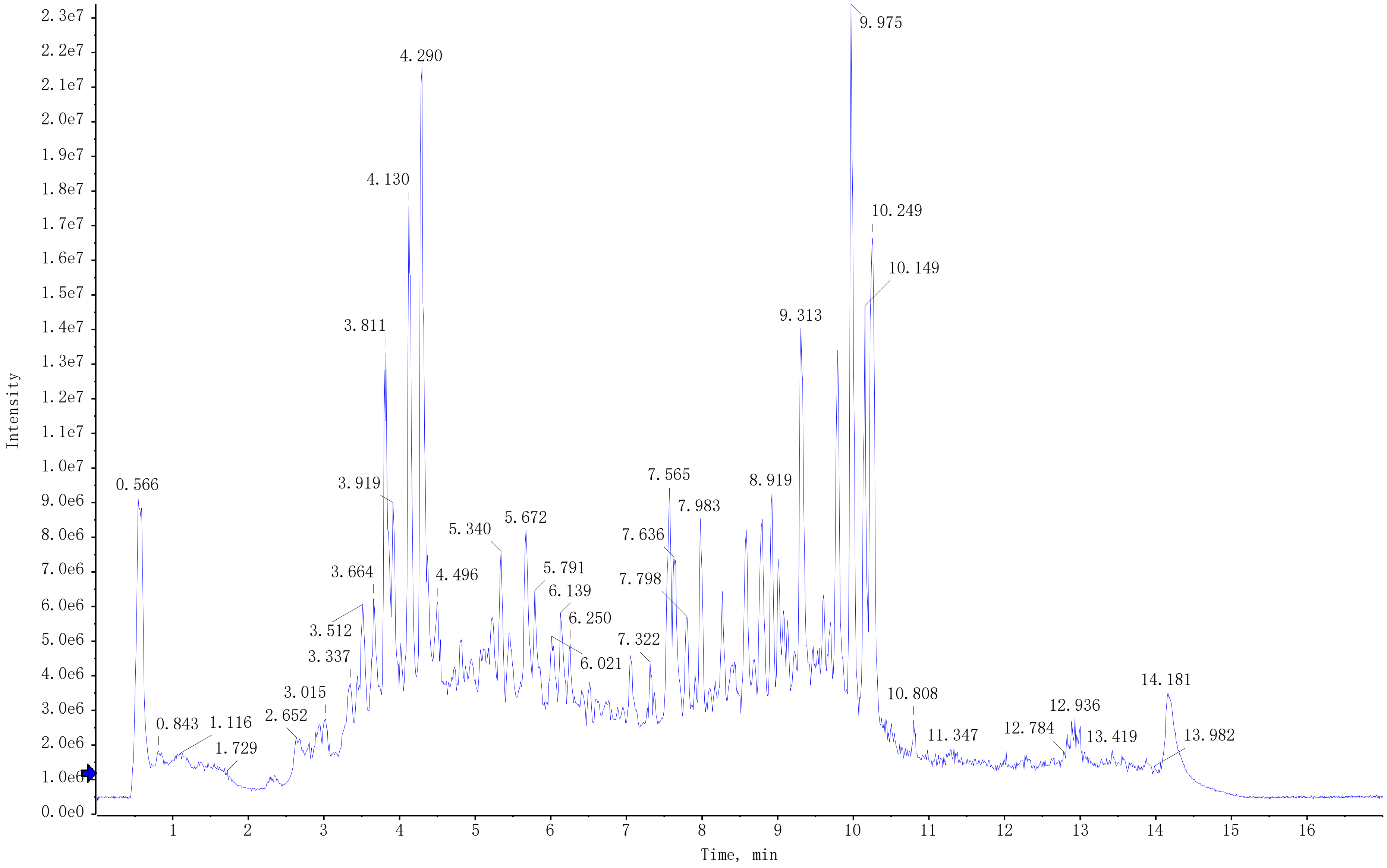

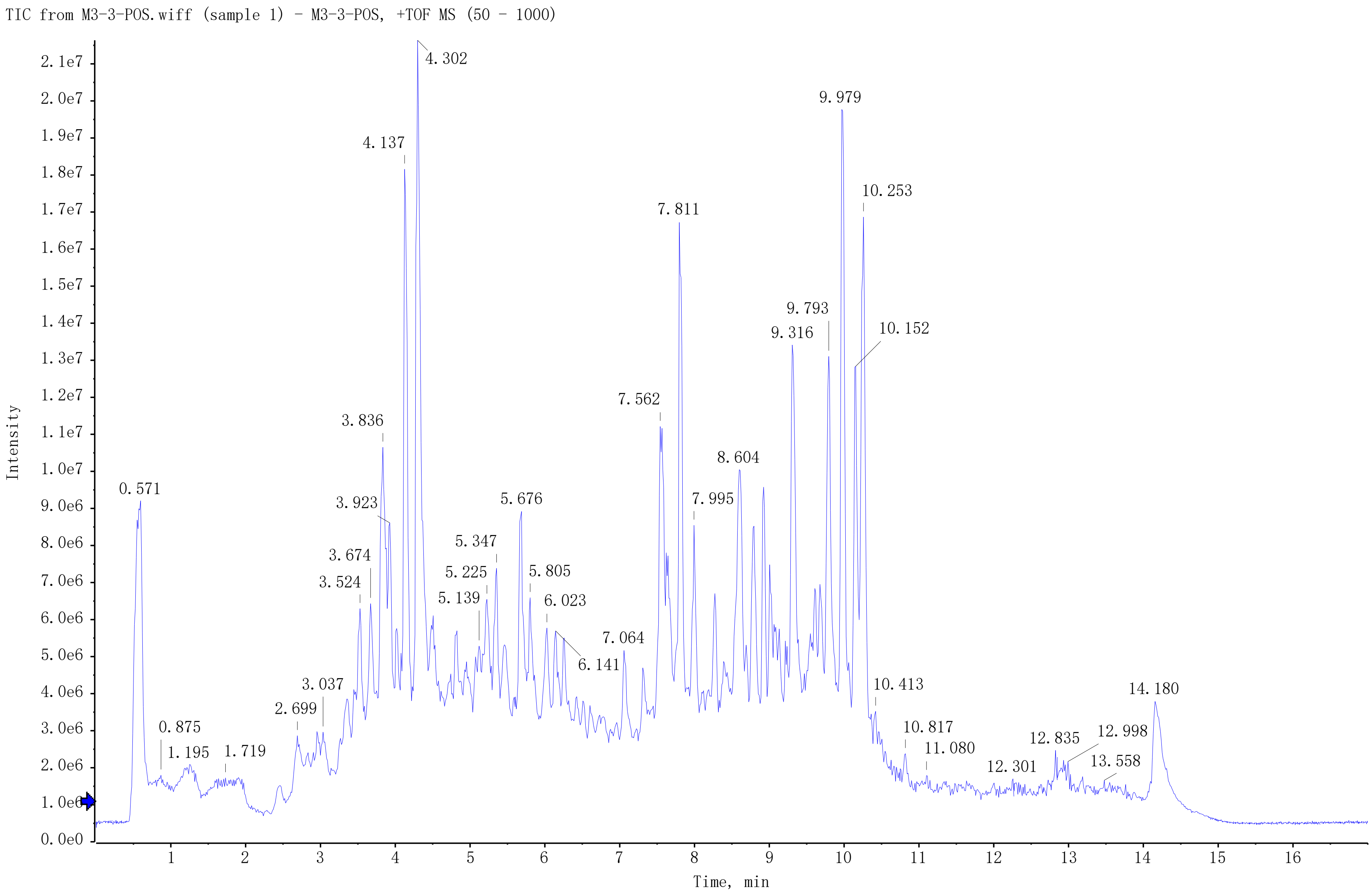
TIC from M3-3-POS.wiff (sample 1) - M3-3-POS, +TOF MS (50 - 1000)
Intensity
Time, min
4.302
9.979
4.137
10.253
7.811
9.793
10.152
9.316
7.562
3.836
8.604
0.571
3.923
7.995
5.676
5.347
3.674
5.225
5.805
3.524
5.139
6.023
7.064
6.141
10.413
14.180
3.037
2.699
0.875
1.195
1.719
10.817
11.080
12.835
12.998
12.301
13.558

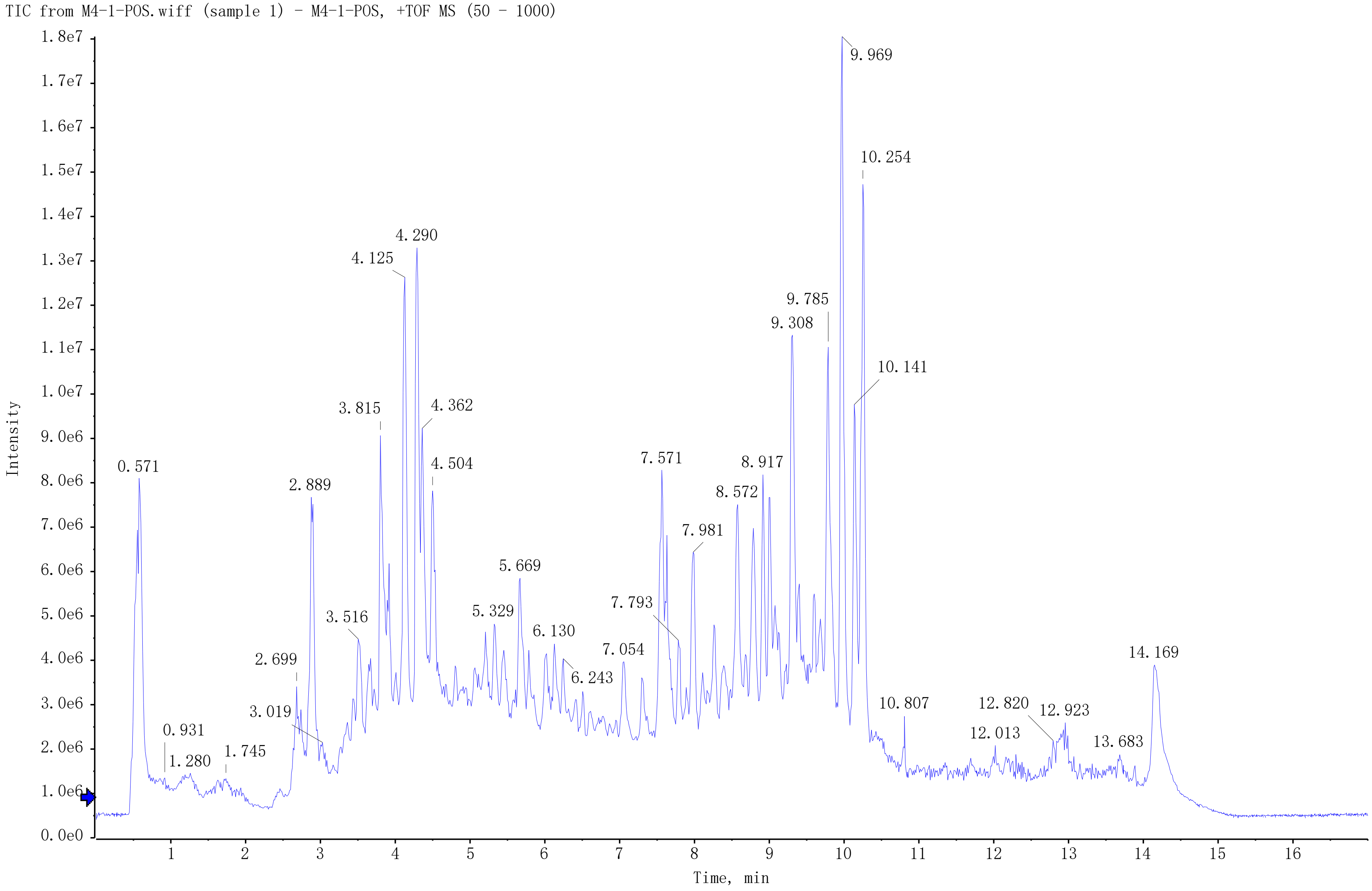

TIC from M4-1-POS.wiff (sample 1) - M4-1-POS, +TOF MS (50 - 1000)
Intensity
Time, min
0.571
0.931
1.280
1.745
2.699
2.889
3.019
3.516
3.815
4.125
4.290
4.362
4.504
5.329
5.669
6.130
6.243
7.054
7.571
7.793
7.981
8.572
8.917
9.308
9.785
9.969
10.141
10.254
10.807
12.013
12.820
12.923
13.683
14.169

TIC from M4-2-POS.wiff (sample 1) - M4-2-POS, +TOF MS (50 - 1000)

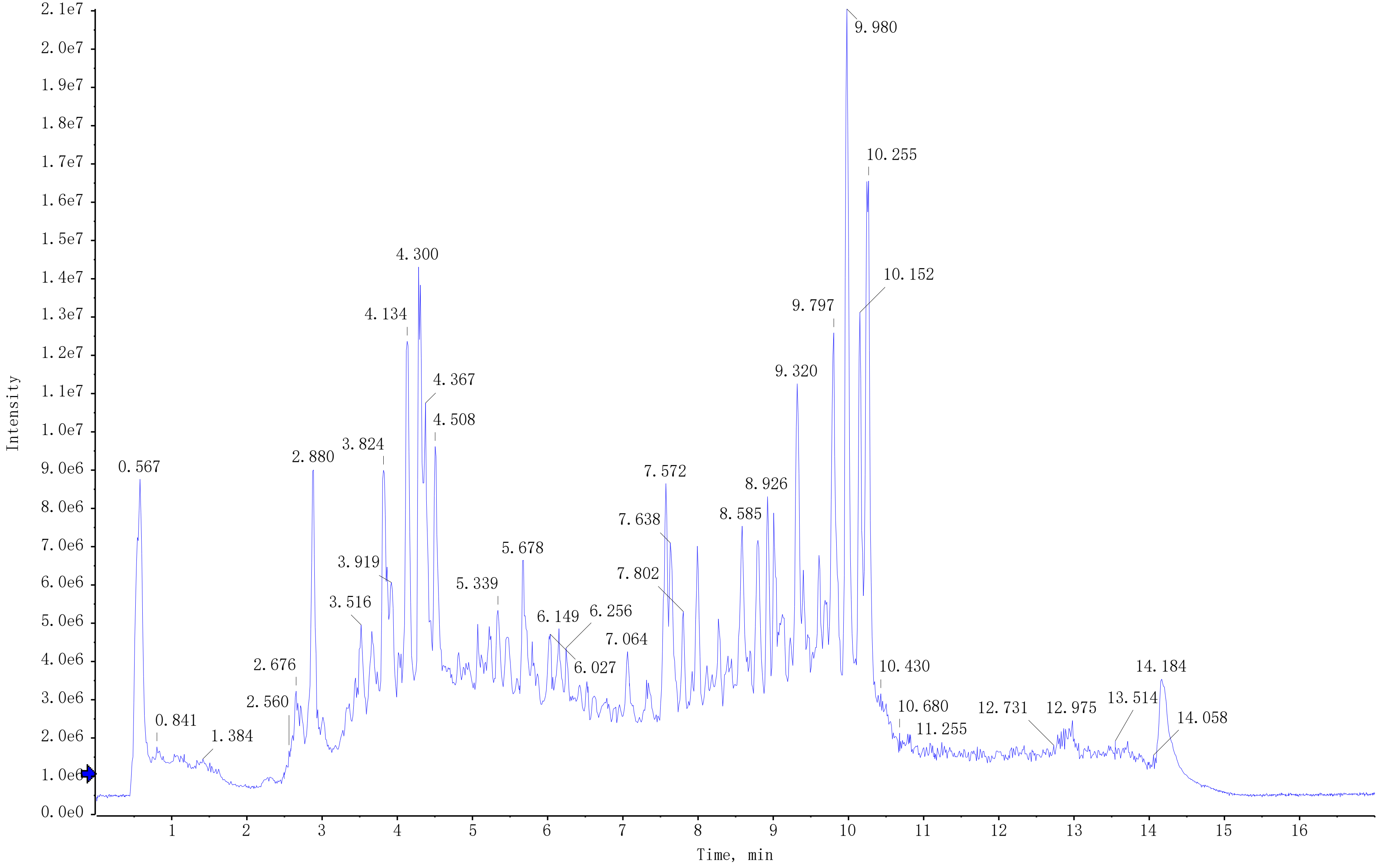

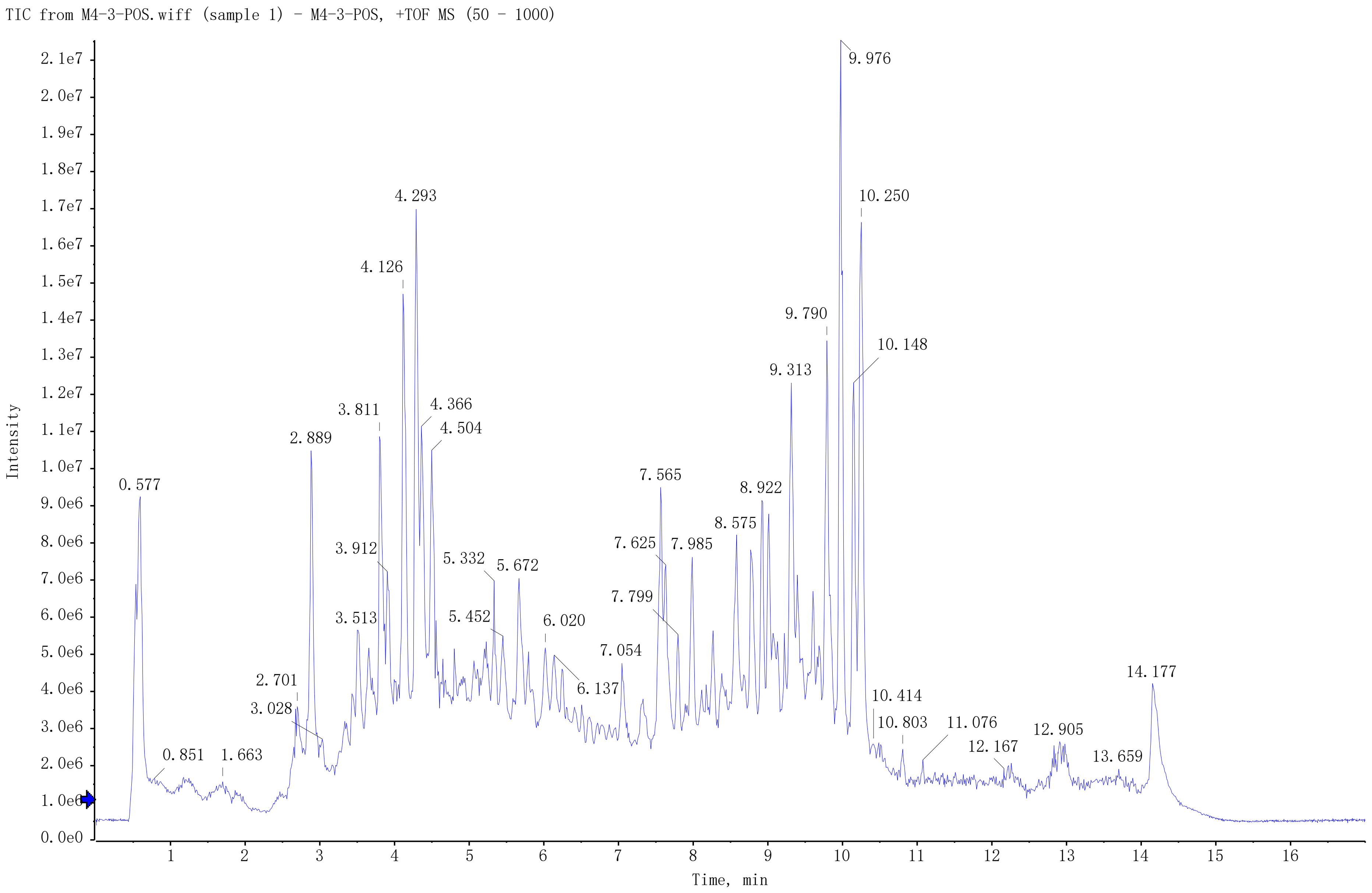
TIC from M4-3-POS.wiff (sample 1) - M4-3-POS, +TOF MS (50 - 1000)
Intensity
Time, min
0.577
0.851
1.663
2.701
2.889
3.028
3.513
3.811
3.912
4.126
4.293
4.366
4.504
5.332
5.452
5.672
6.020
6.137
7.054
7.565
7.625
7.799
7.985
8.575
8.922
9.313
9.790
9.976
10.148
10.250
10.414
10.803
11.076
12.167
12.905
13.659
14.177

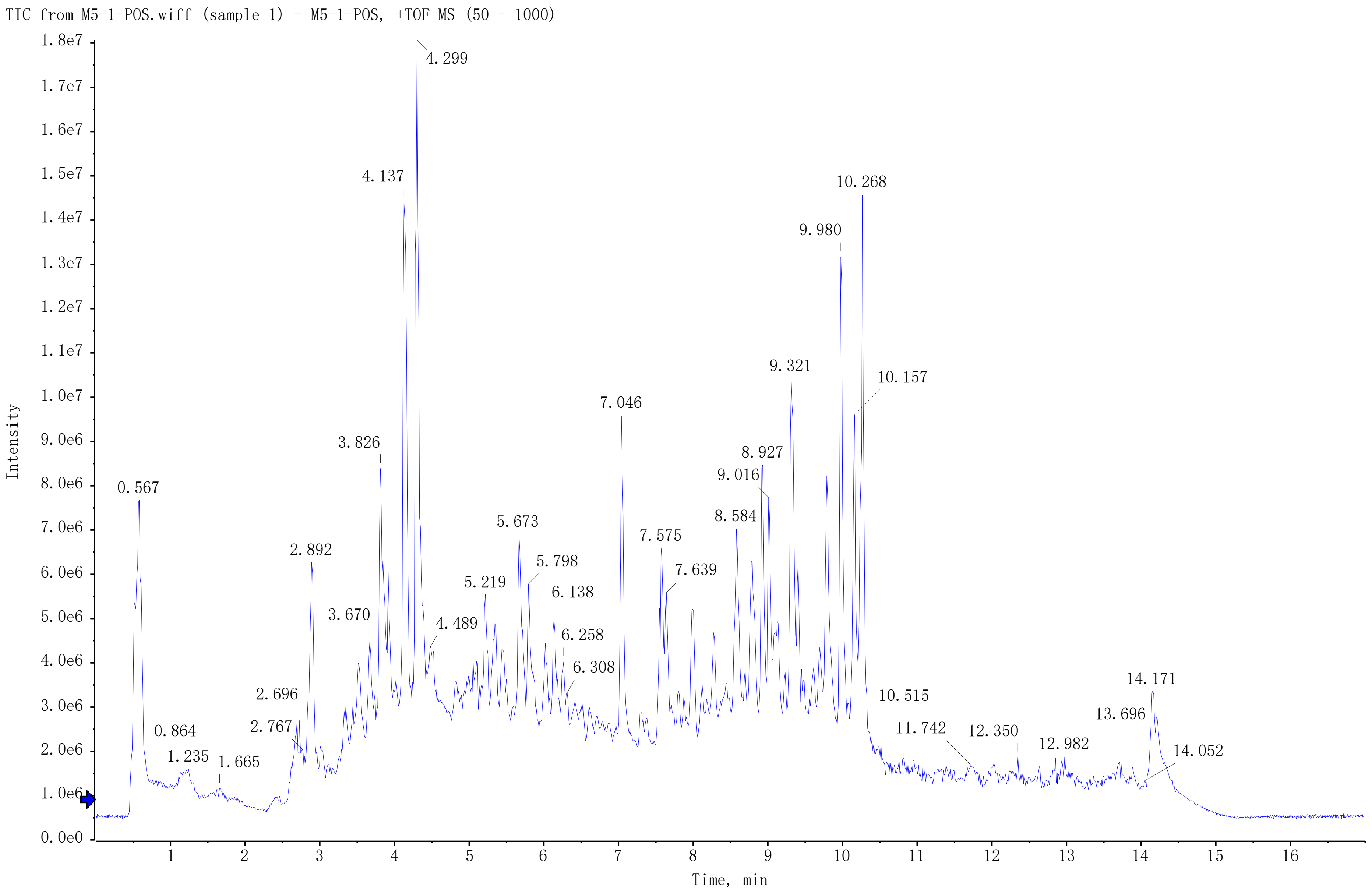
TIC from M5-1-POS.wiff (sample 1) - M5-1-POS, +TOF MS (50 - 1000)
Intensity
Time, min
0.567
0.864
1.235
1.665
2.696
2.767
2.892
3.670
3.826
4.137
4.299
4.489
5.219
5.673
5.798
6.138
6.258
6.308
7.046
7.575
7.639
8.584
8.927
9.016
9.321
9.980
10.268
10.157
10.515
11.742
12.350
12.982
13.696
14.171
14.052

TIC from M5-2-POS.wiff (sample 1) - M5-2-POS, +TOF MS (50 - 1000)

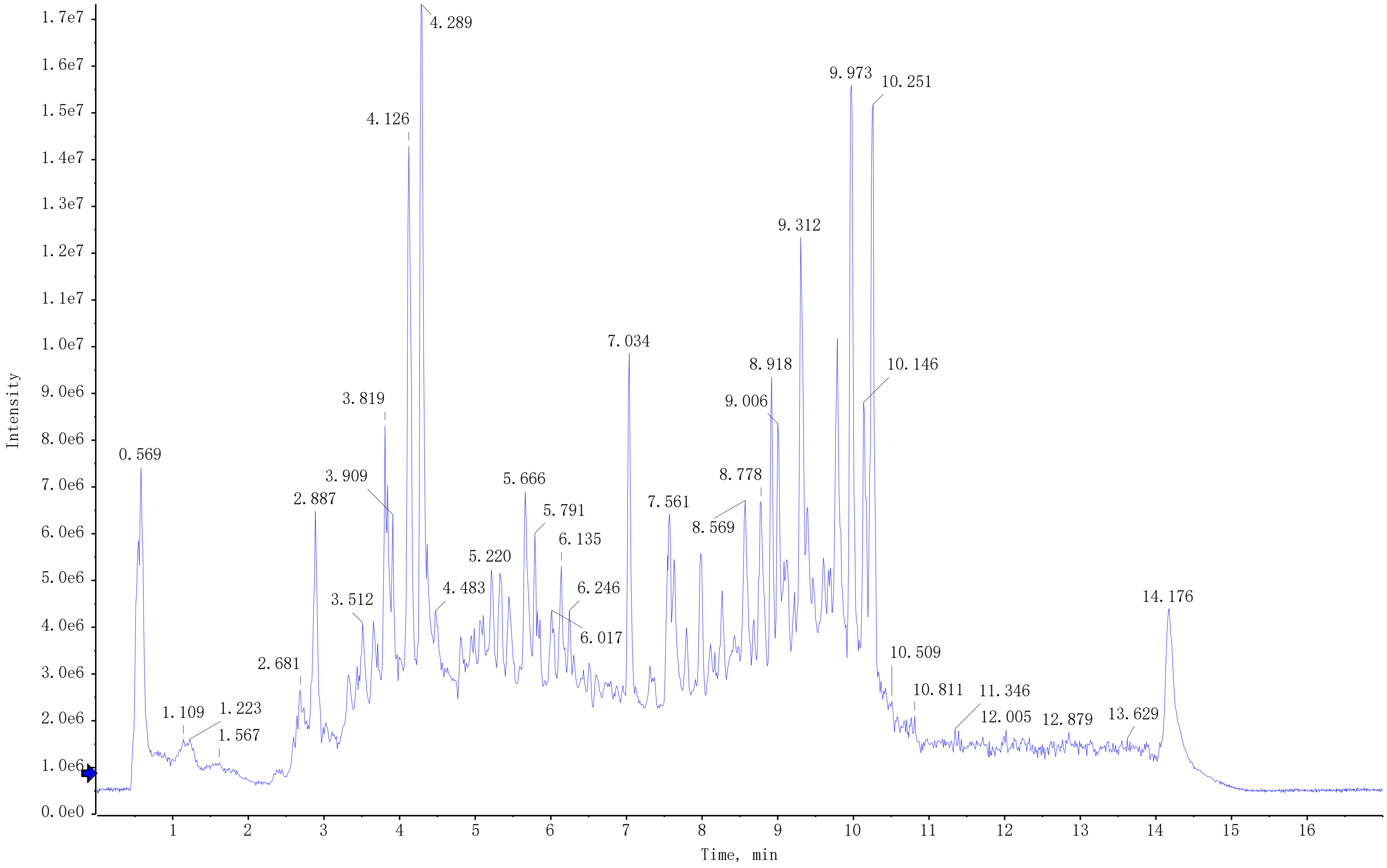

TIC from M5-3-POS.wiff (sample 1) - M5-3-POS, +TOF MS (50 - 1000)

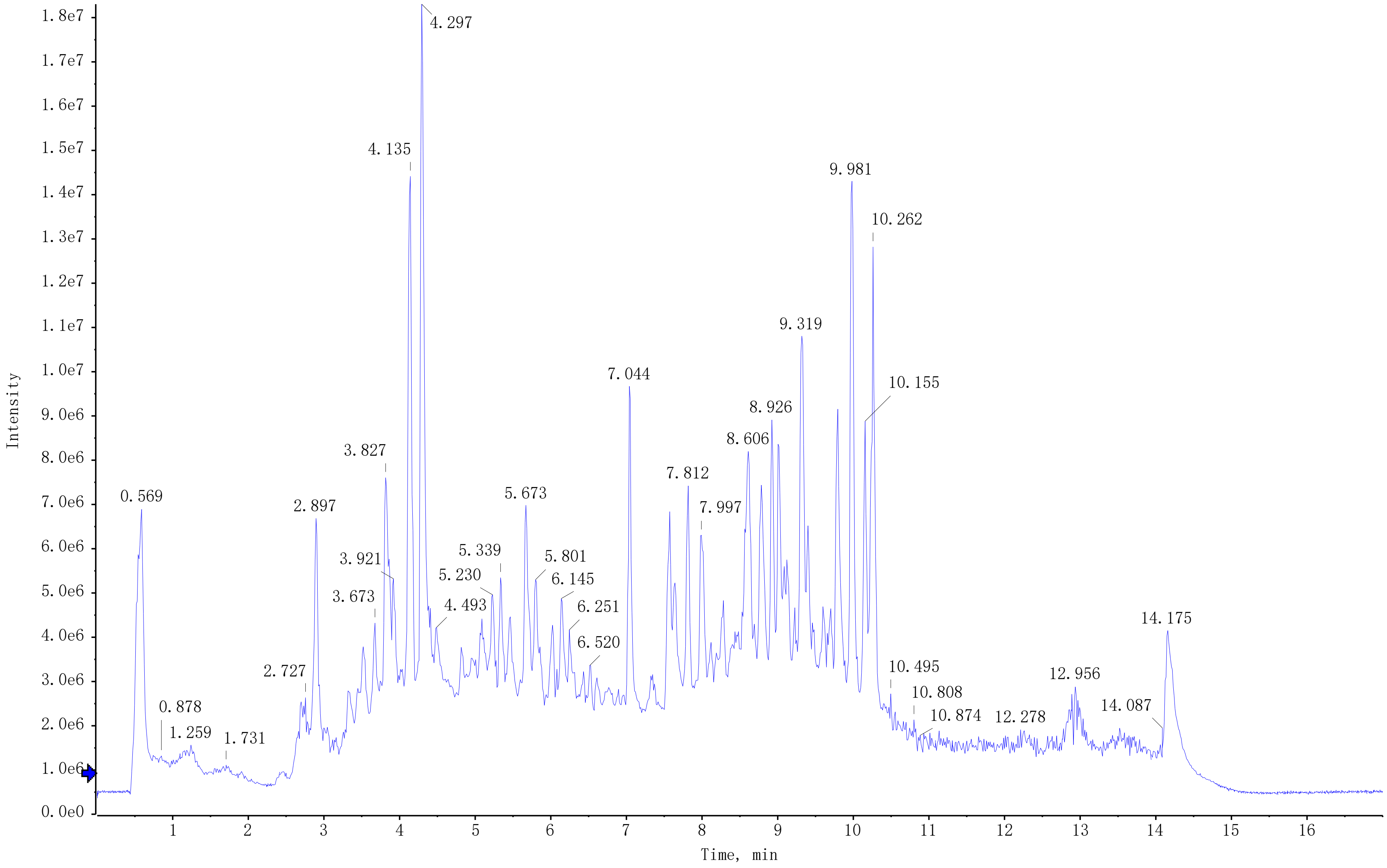

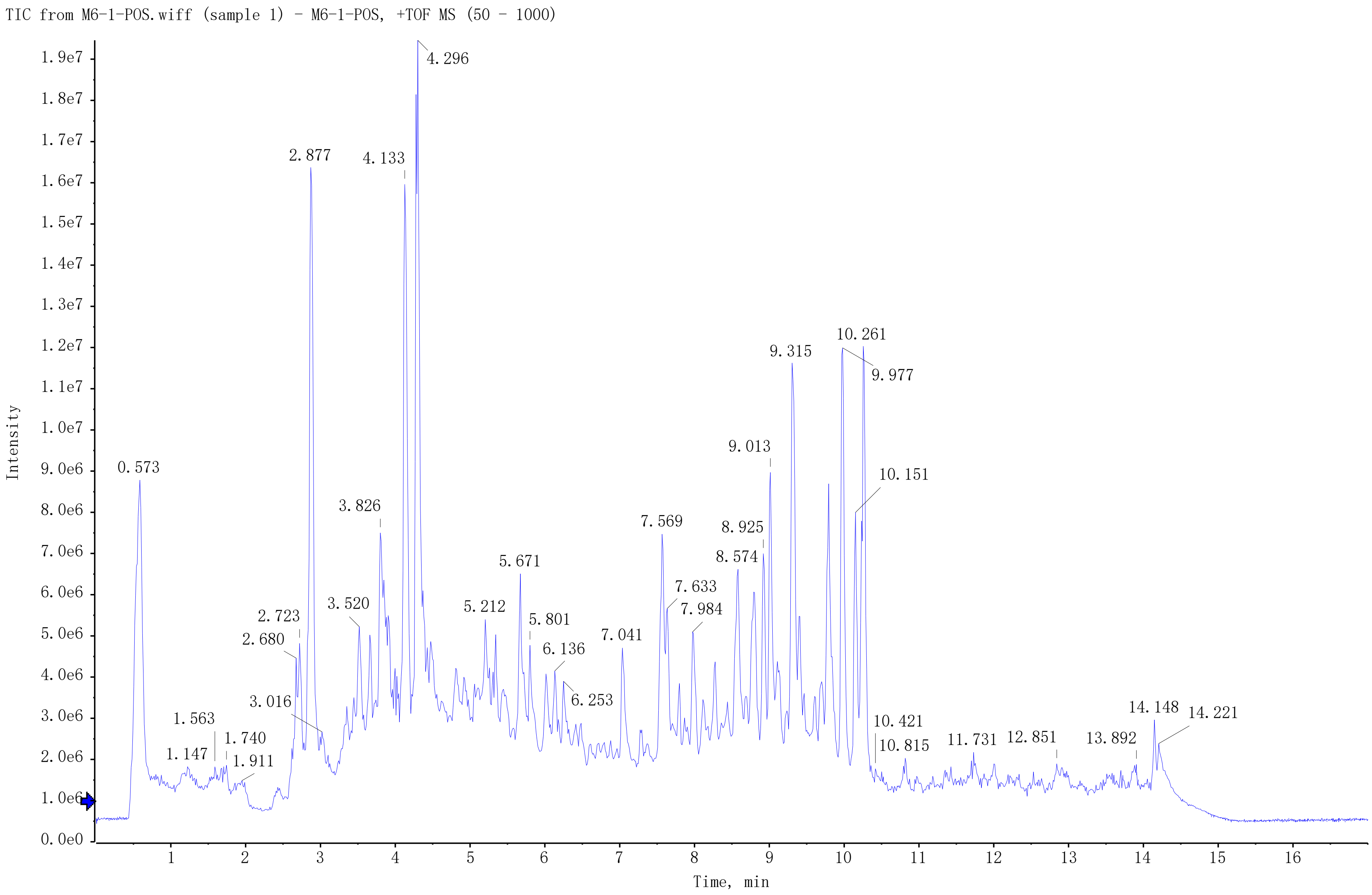
TIC from M6-1-POS.wiff (sample 1) - M6-1-POS, +TOF MS (50 - 1000)
Intensity
Time, min
1.9e7
1.8e7
1.7e7
1.6e7
1.5e7
1.4e7
1.3e7
1.2e7
1.1e7
1.0e7
9.0e6
8.0e6
7.0e6
6.0e6
5.0e6
4.0e6
3.0e6
2.0e6
1.0e6
0.0e0
1
2
3
4
5
6
7
8
9
10
11
12
13
14
15
16
0.573
1.147
1.563
1.740
1.911
2.680
2.723
2.877
3.016
3.520
3.826
4.133
4.296
5.212
5.671
5.801
6.136
6.253
7.041
7.569
7.633
7.984
8.574
8.925
9.013
9.315
9.977
10.261
10.151
10.421
10.815
11.731
12.851
13.892
14.148
14.221

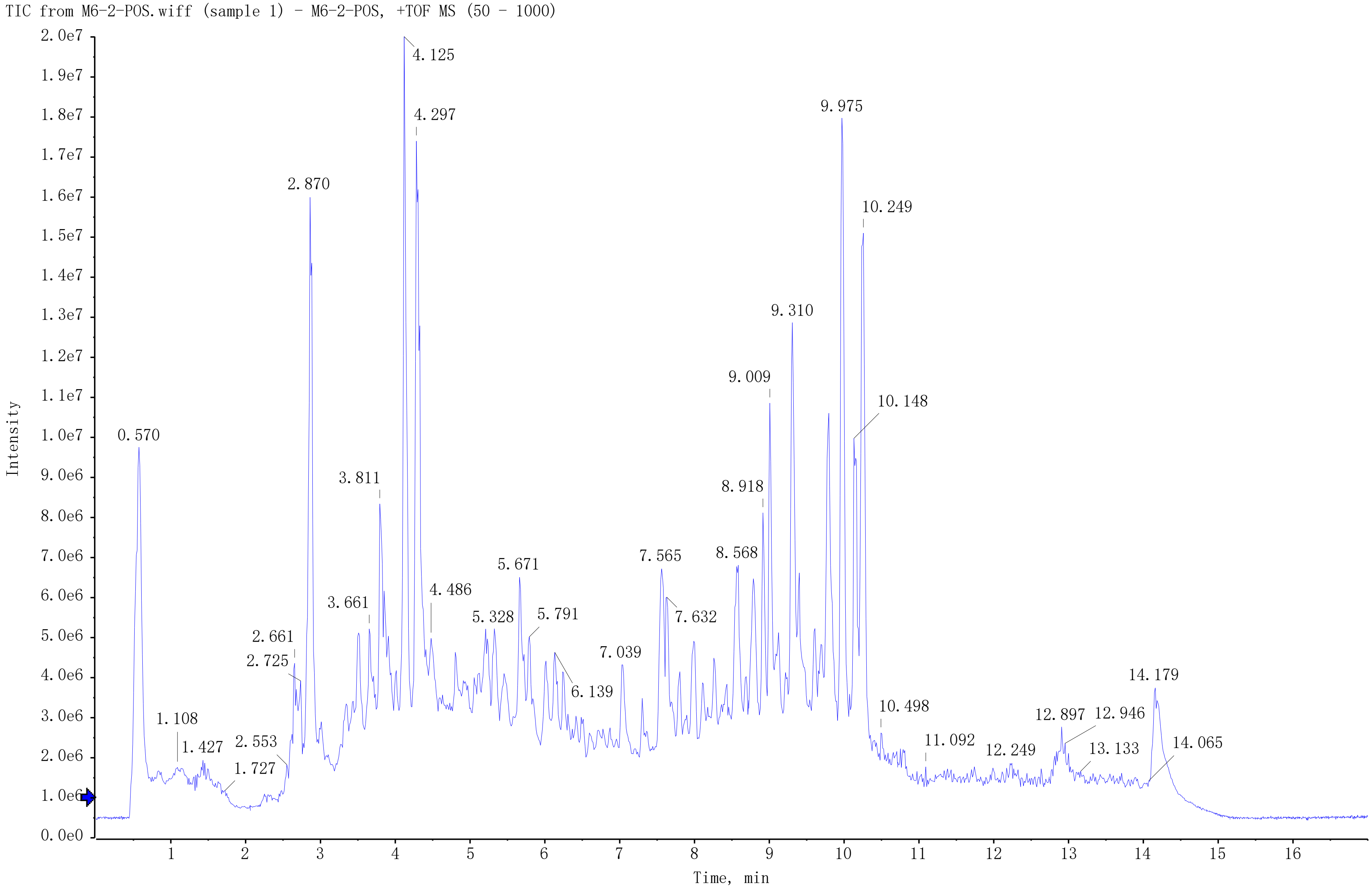
TIC from M6-2-POS.wiff (sample 1) - M6-2-POS, +TOF MS (50 - 1000)
Intensity
Time, min
0.570
1.108
1.427
1.727
2.553
2.661
2.725
2.870
3.661
3.811
4.125
4.297
4.486
5.328
5.671
5.791
6.139
7.039
7.565
7.632
8.568
8.918
9.009
9.310
9.975
10.148
10.249
10.498
11.092
12.249
12.897
12.946
13.133
14.065
14.179

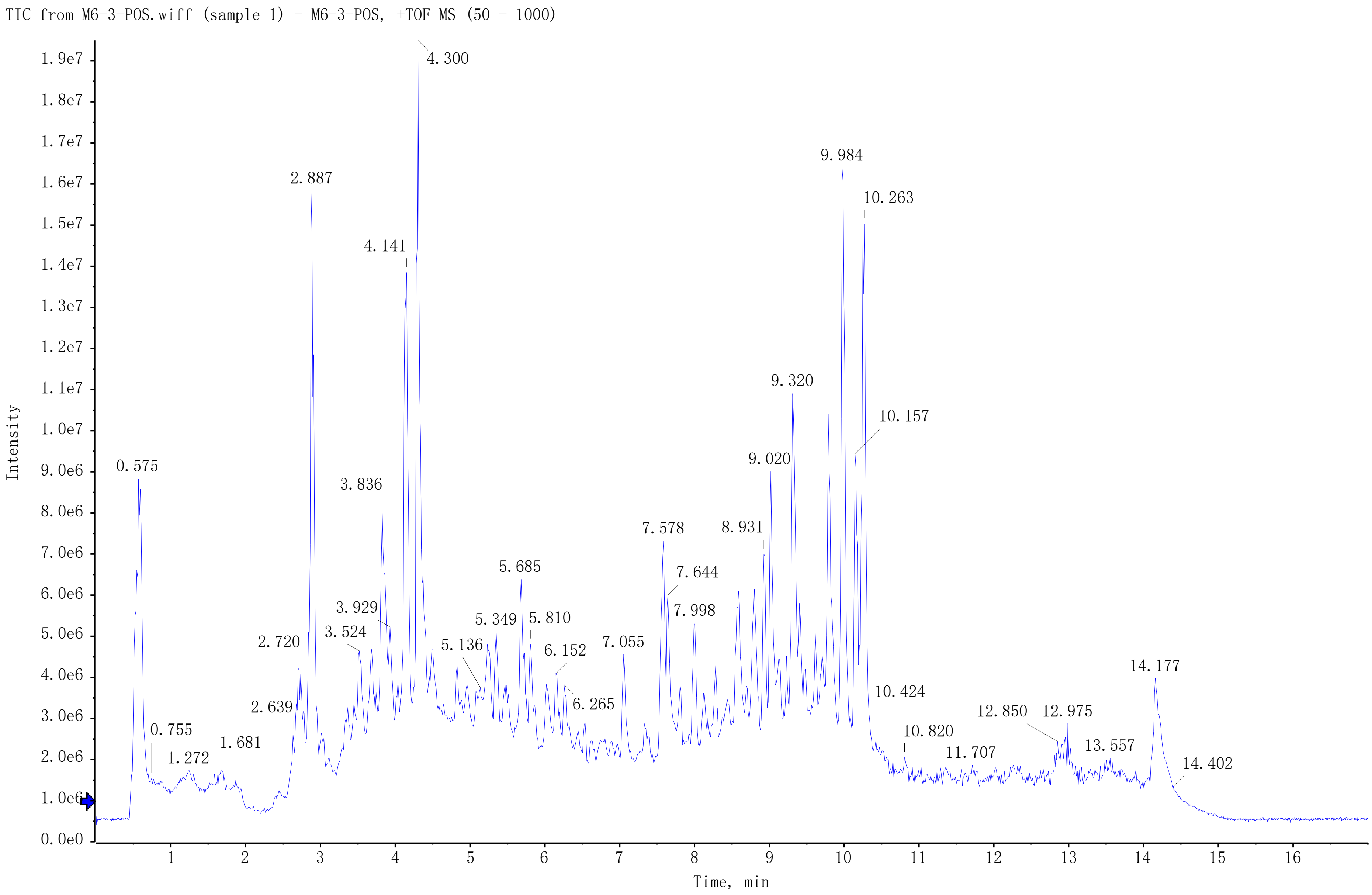

TIC from M6-3-POS.wiff (sample 1) - M6-3-POS, +TOF MS (50 - 1000)
Intensity
Time, min
4.300
9.984
10.263
2.887
4.141
9.320
10.157
9.020
0.575
3.836
7.578
8.931
5.685
7.644
3.929
7.998
5.349
5.810
3.524
2.720
5.136
6.152
7.055
6.265
2.639
14.177
10.424
12.850
12.975
0.755
1.681
10.820
1.272
11.707
13.557
14.402

TIC from M7-1-POS.wiff (sample 1) - M7-1-POS, +TOF MS (50 - 1000)

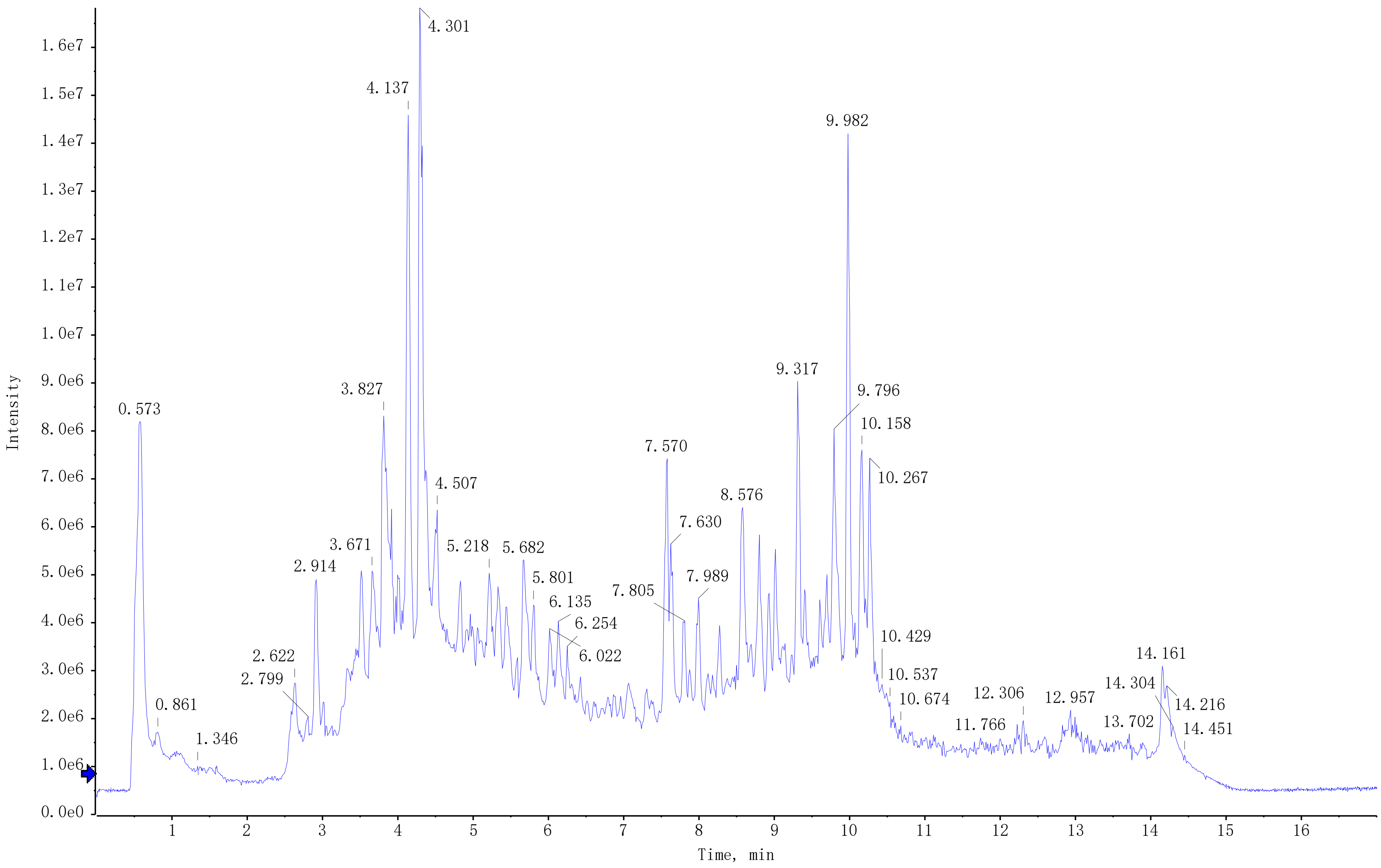

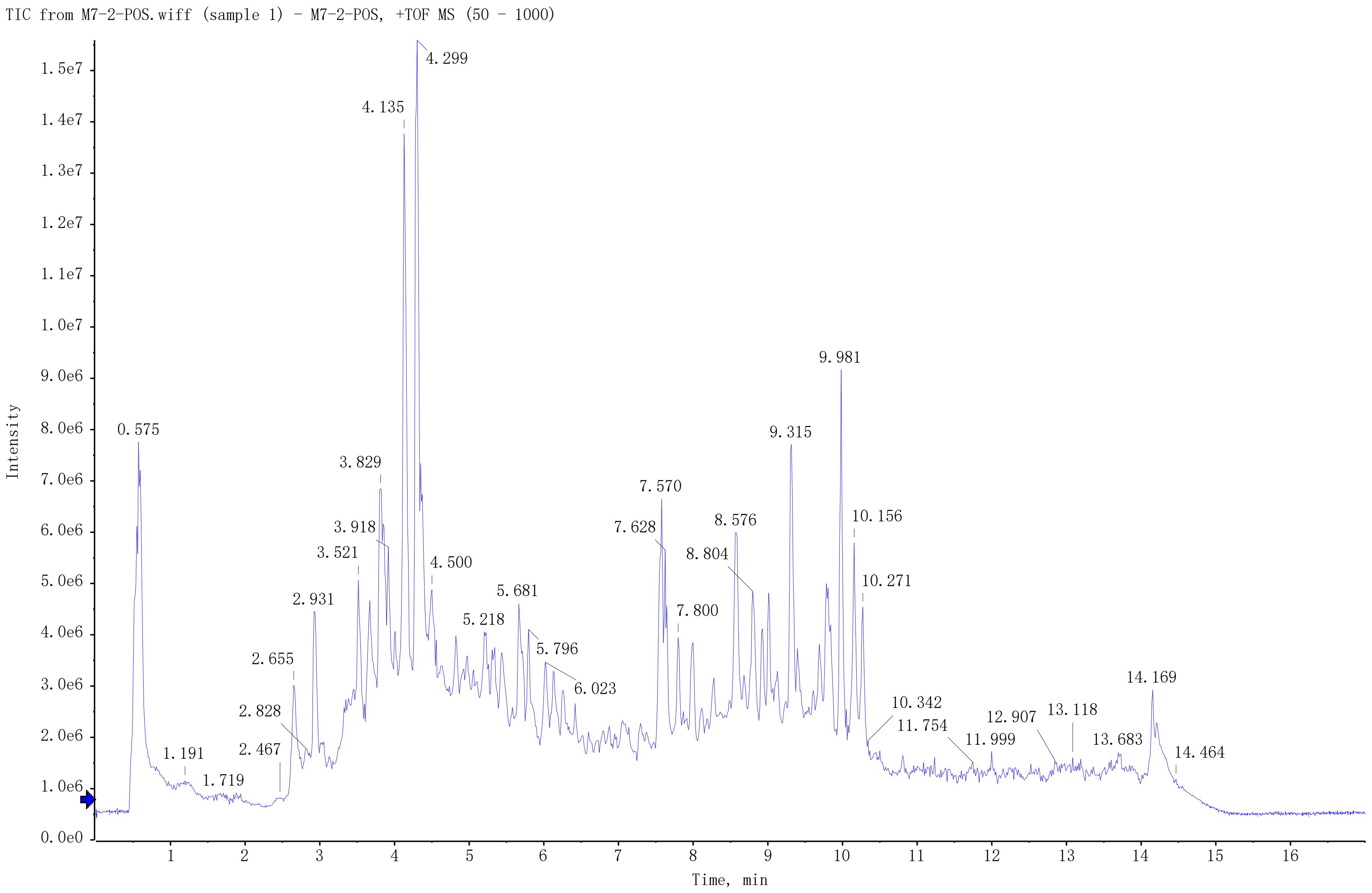
TIC from M7-2-POS.wiff (sample 1) - M7-2-POS, +TOF MS (50 - 1000)
Intensity
Time, min
1.5e7
1.4e7
1.3e7
1.2e7
1.1e7
1.0e7
9.0e6
8.0e6
7.0e6
6.0e6
5.0e6
4.0e6
3.0e6
2.0e6
1.0e6
0.0e0
1
2
3
4
5
6
7
8
9
10
11
12
13
14
15
16
4.299
4.135
9.981
0.575
9.315
3.829
7.570
3.918
7.628
8.576
10.156
3.521
8.804
4.500
10.271
2.931
5.681
5.218
7.800
5.796
2.655
6.023
14.169
10.342
2.828
11.754
12.907
13.118
11.999
13.683
2.467
1.191
14.464
1.719

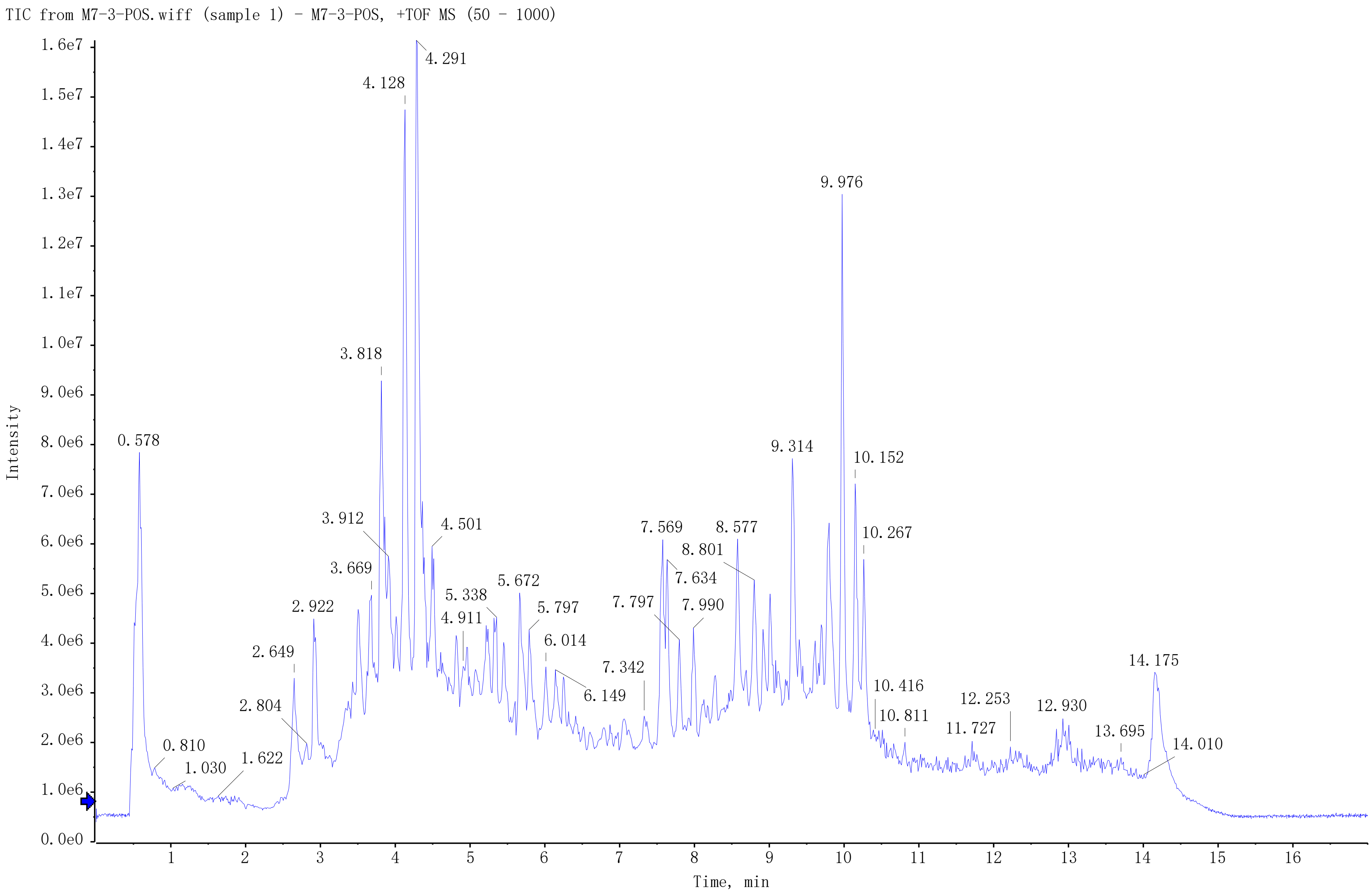

TIC from M7-3-POS.wiff (sample 1) - M7-3-POS, +TOF MS (50 - 1000)
Intensity
Time, min
1.6e7
1.5e7
1.4e7
1.3e7
1.2e7
1.1e7
1.0e7
9.0e6
8.0e6
7.0e6
6.0e6
5.0e6
4.0e6
3.0e6
2.0e6
1.0e6
0.0e0
1
2
3
4
5
6
7
8
9
10
11
12
13
14
15
16
0.578
0.810
1.030
1.622
2.649
2.804
2.922
3.669
3.818
3.912
4.128
4.291
4.501
4.911
5.338
5.672
5.797
6.014
6.149
7.342
7.569
7.634
7.797
7.990
8.577
8.801
9.314
9.976
10.152
10.267
10.416
10.811
11.727
12.253
12.930
13.695
14.010
14.175

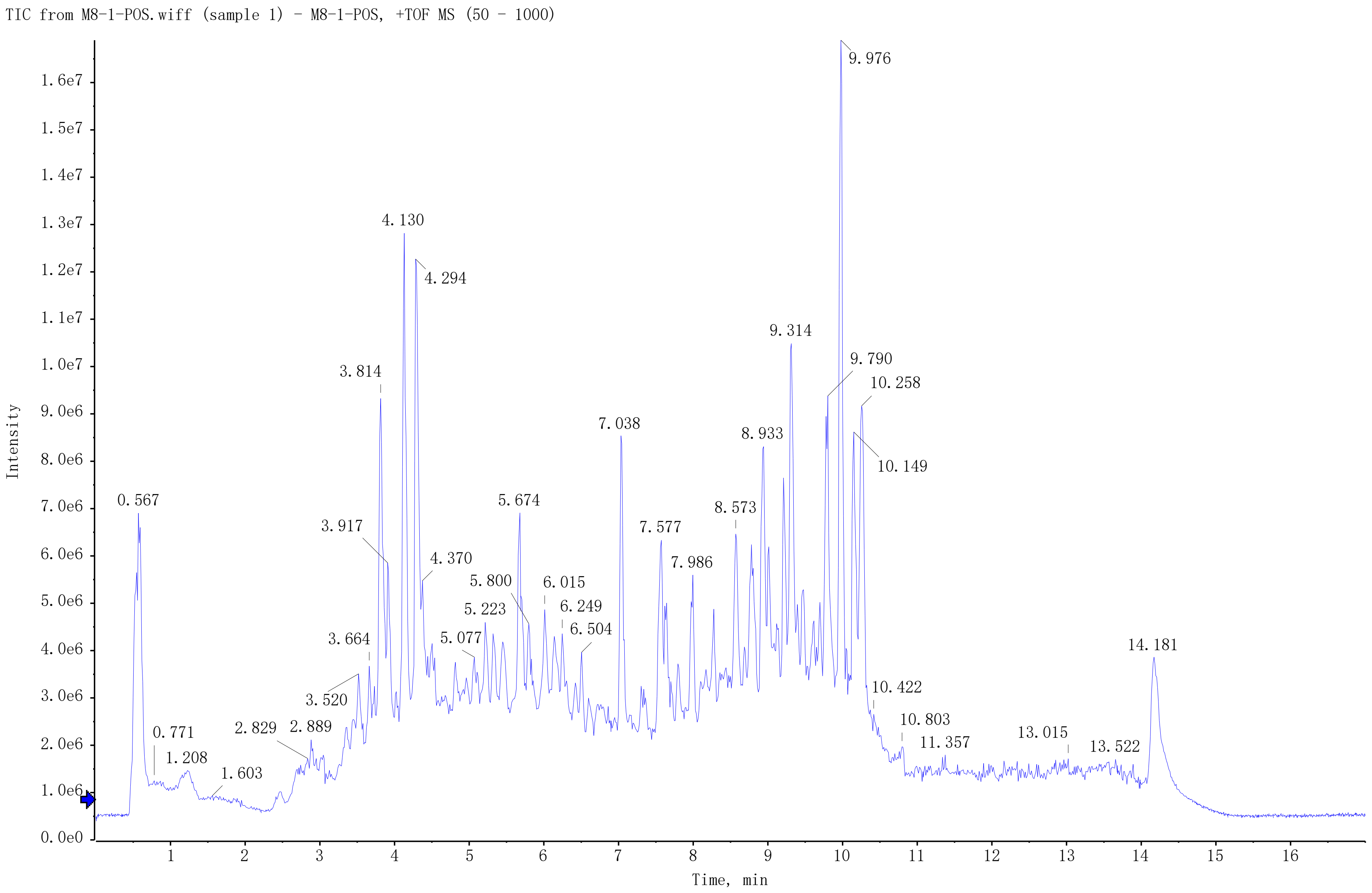

TIC from M8-1-POS.wiff (sample 1) - M8-1-POS, +TOF MS (50 - 1000)
Intensity
Time, min
1.6e7
1.5e7
1.4e7
1.3e7
1.2e7
1.1e7
1.0e7
9.0e6
8.0e6
7.0e6
6.0e6
5.0e6
4.0e6
3.0e6
2.0e6
1.0e6
0.0e0
1
2
3
4
5
6
7
8
9
10
11
12
13
14
15
16
9.976
4.130
4.294
9.314
3.814
9.790
10.258
7.038
8.933
10.149
0.567
5.674
8.573
3.917
7.577
4.370
7.986
5.800
6.015
6.249
5.223
6.504
3.664
5.077
14.181
3.520
10.422
0.771
2.829
2.889
10.803
11.357
13.015
1.208
13.522
1.603

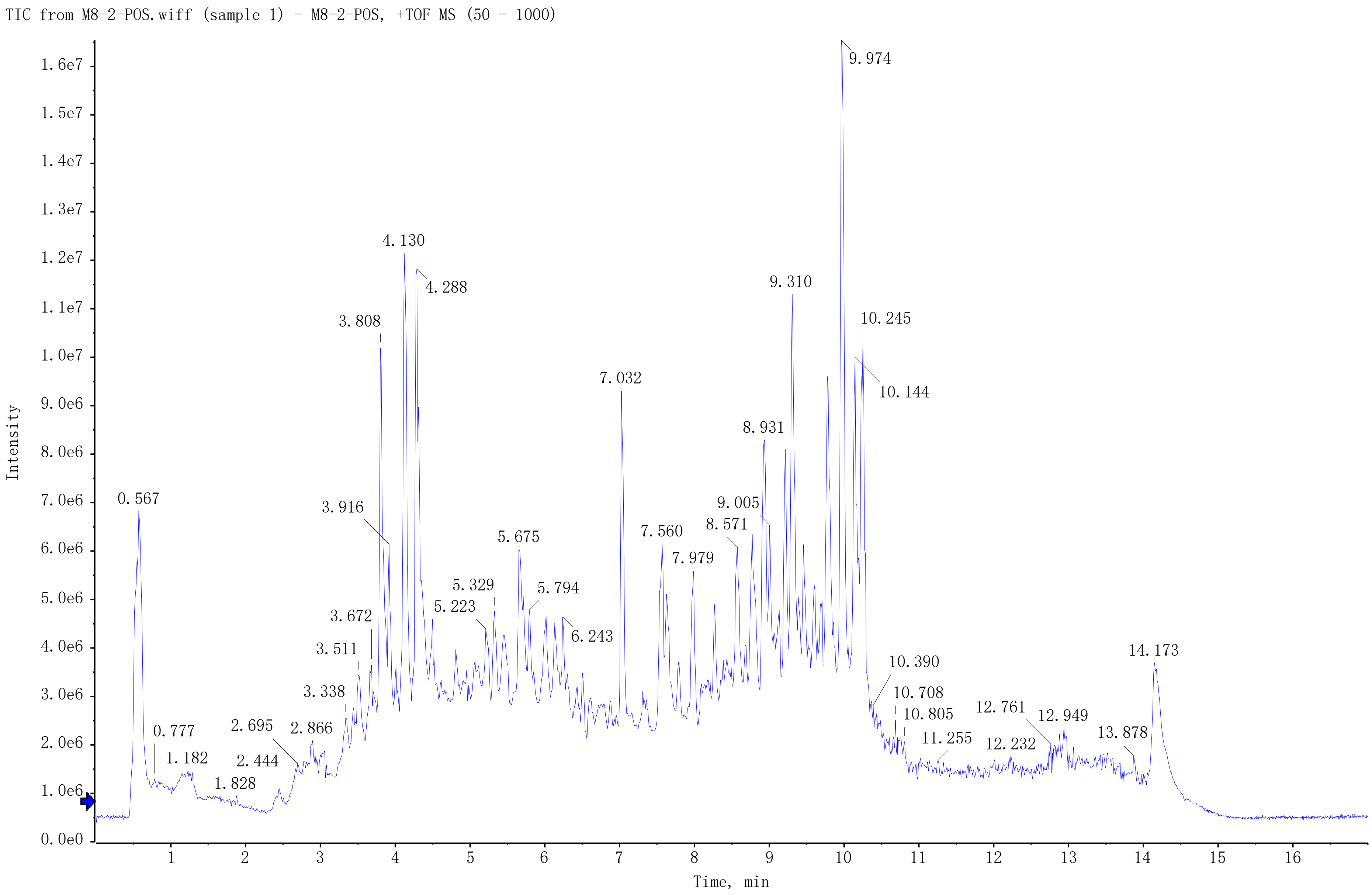
TIC from M8-2-POS.wiff (sample 1) - M8-2-POS, +TOF MS (50 - 1000)
Intensity
Time, min
0.567
0.777
1.182
1.828
2.444
2.695
2.866
3.338
3.511
3.672
3.808
3.916
4.130
4.288
5.223
5.329
5.675
5.794
6.243
7.032
7.560
7.979
8.571
8.931
9.005
9.310
9.974
10.144
10.245
10.390
10.708
10.805
11.255
12.232
12.761
12.949
13.878
14.173

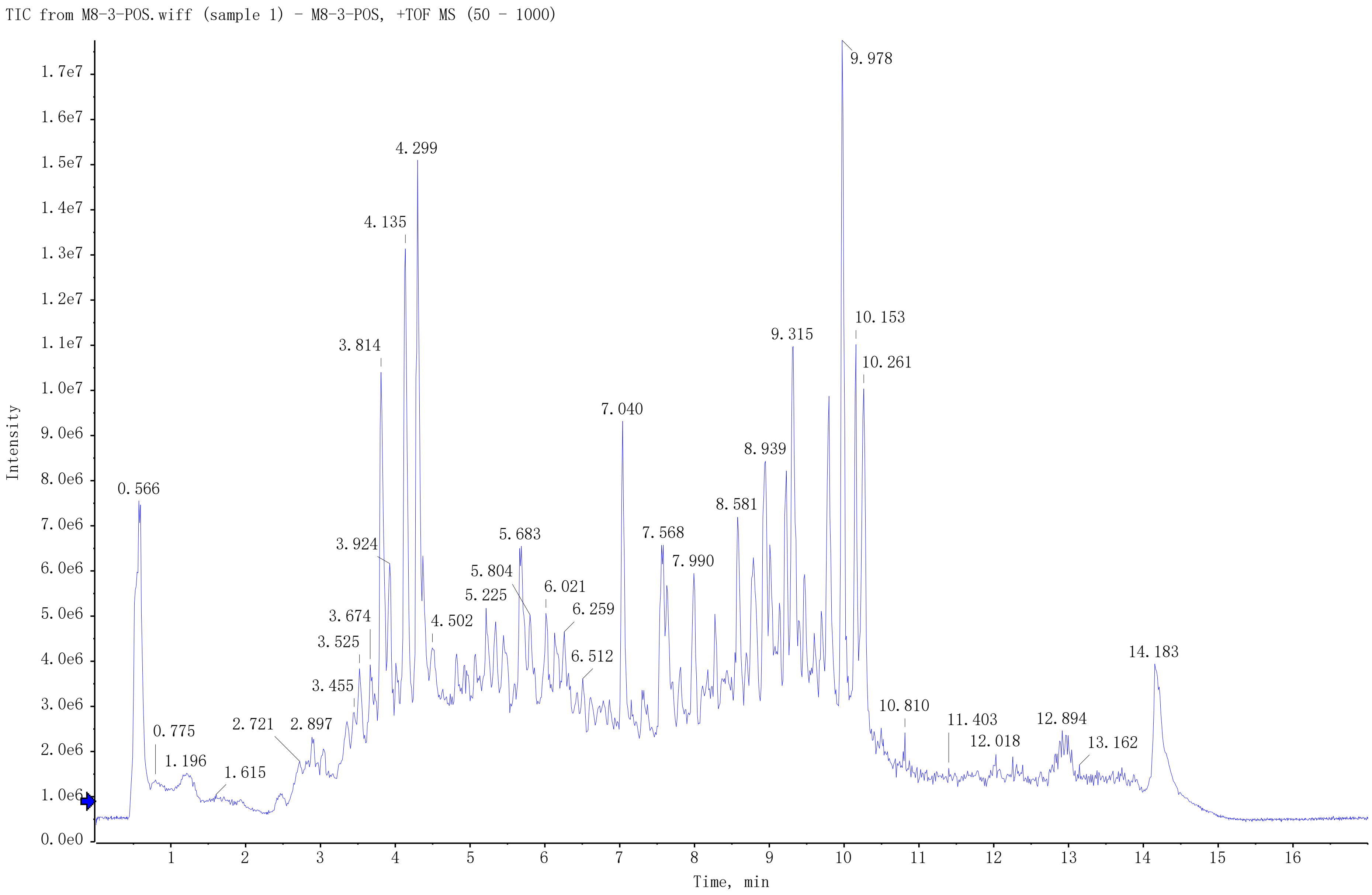

TIC from M8-3-POS.wiff (sample 1) - M8-3-POS, +TOF MS (50 - 1000)
Intensity
Time, min
9.978
4.299
4.135
3.814
3.924
3.674
3.525
3.455
0.566
0.775
1.196
1.615
2.721
2.897
4.502
5.225
5.683
5.804
6.021
6.259
6.512
7.040
7.568
7.990
8.581
8.939
9.315
10.153
10.261
10.810
11.403
12.018
12.894
13.162
14.183

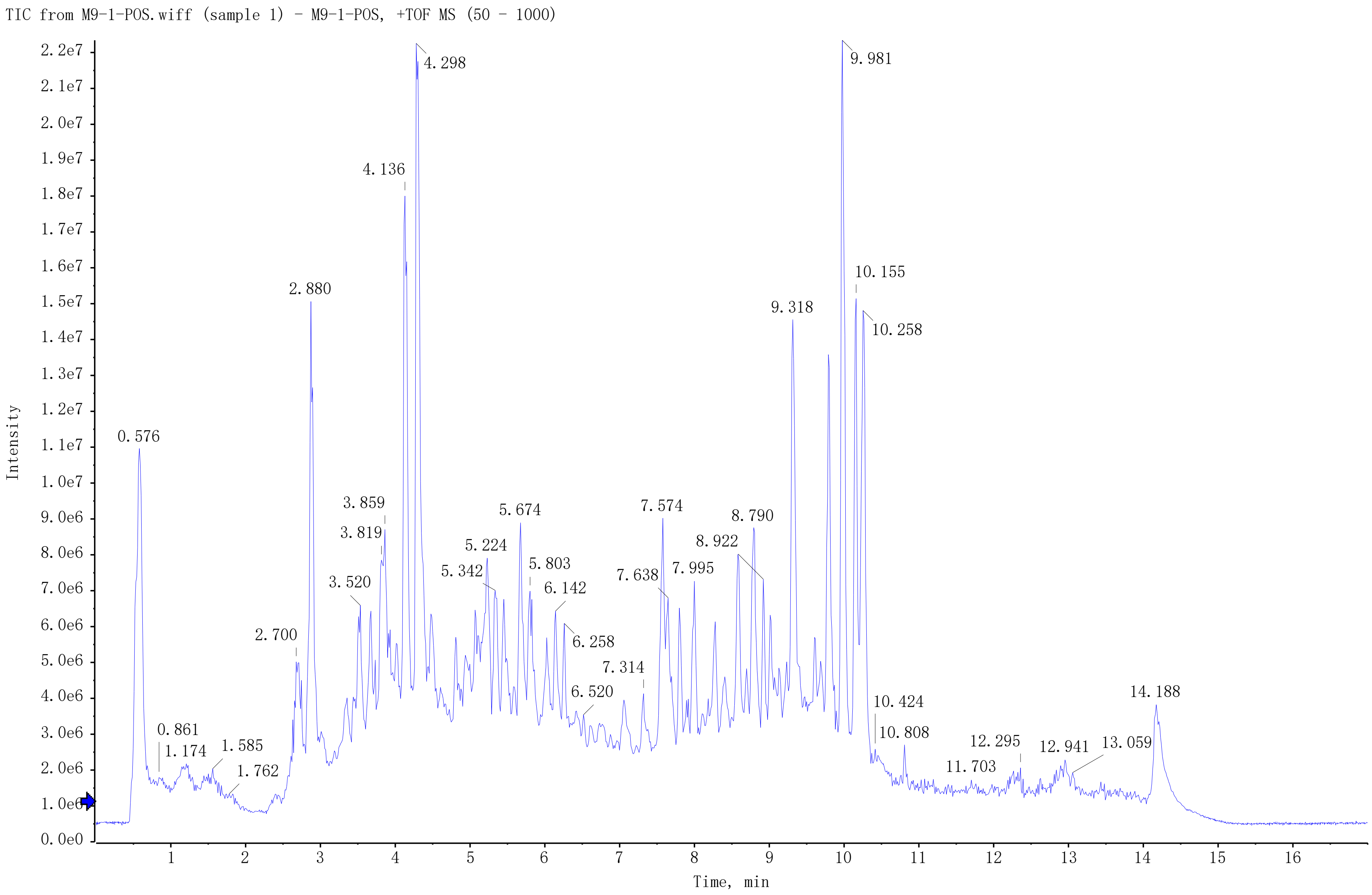
TIC from M9-1-POS.wiff (sample 1) - M9-1-POS, +TOF MS (50 - 1000)
Intensity
Time, min
0.576
0.861
1.174
1.585
1.762
2.700
2.880
3.520
3.819
3.859
4.136
4.298
5.224
5.342
5.674
5.803
6.142
6.258
6.520
7.314
7.574
7.638
7.995
8.922
8.790
9.318
9.981
10.155
10.258
10.424
10.808
11.703
12.295
12.941
13.059
14.188

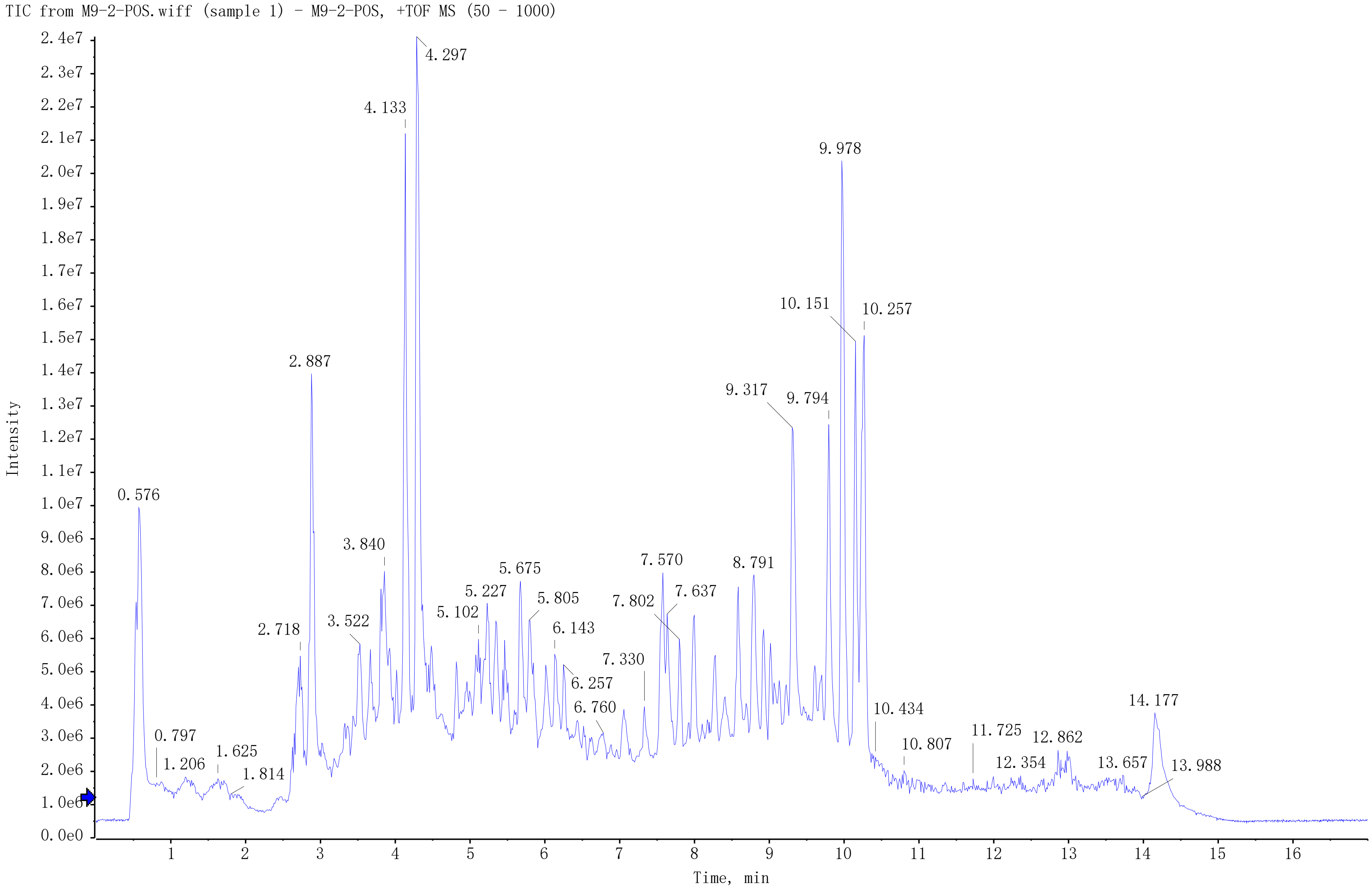
TIC from M9-2-POS.wiff (sample 1) - M9-2-POS, +TOF MS (50 - 1000)
Intensity
Time, min
0.576
0.797
1.206
1.625
1.814
2.718
2.887
3.522
3.840
4.133
4.297
5.102
5.227
5.675
5.805
6.143
6.257
6.760
7.330
7.570
7.802
7.637
8.791
9.317
9.794
9.978
10.151
10.257
10.434
10.807
11.725
12.354
12.862
13.657
13.988
14.177

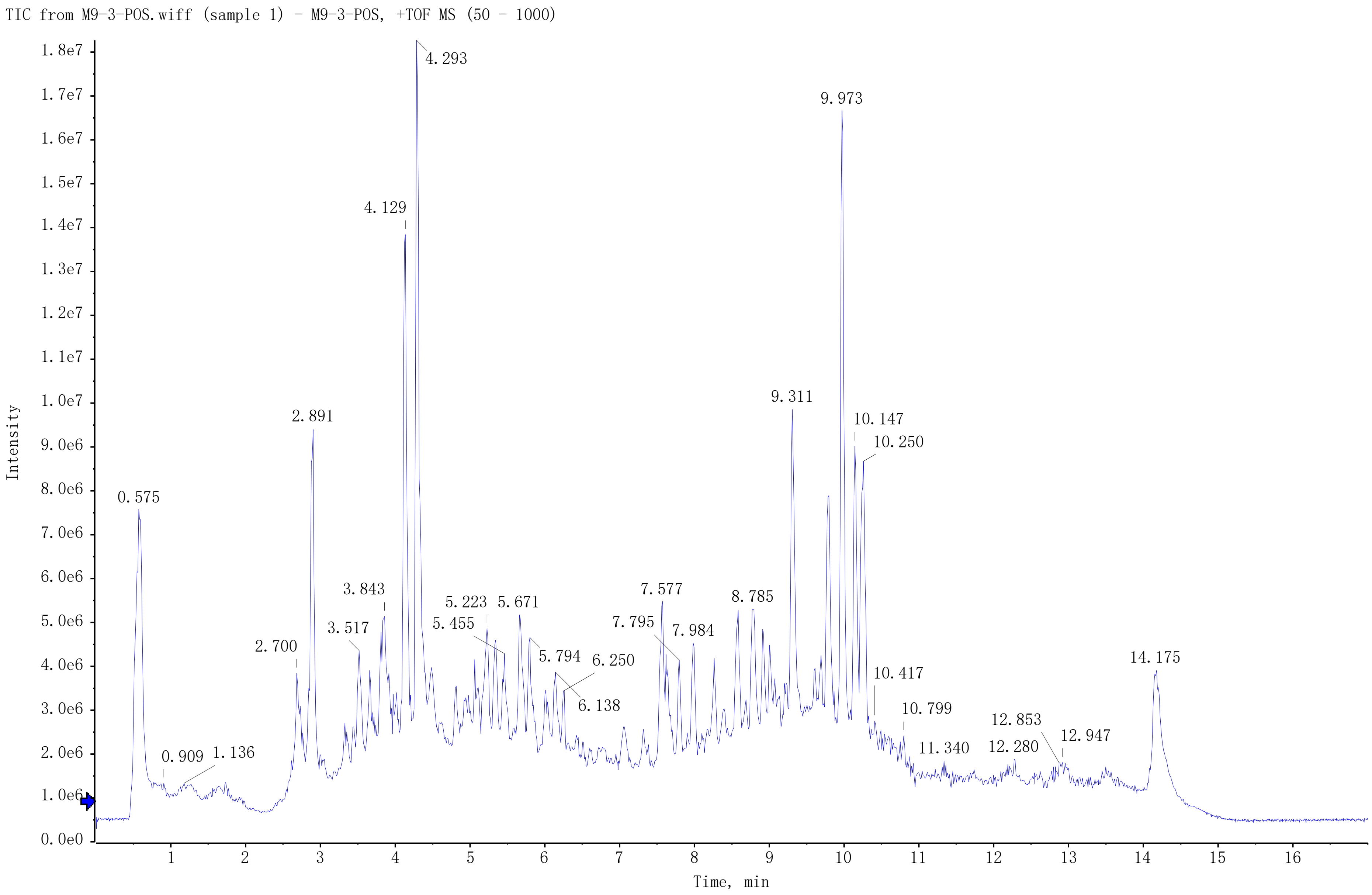
TIC from M9-3-POS.wiff (sample 1) - M9-3-POS, +TOF MS (50 - 1000)
Intensity
Time, min
4.293
9.973
4.129
9.311
2.891
10.147
10.250
0.575
3.843
7.577
8.785
5.223
5.671
3.517
5.455
7.795
7.984
2.700
5.794
6.250
14.175
10.417
6.138
10.799
12.853
12.947
0.909
1.136
11.340
12.280

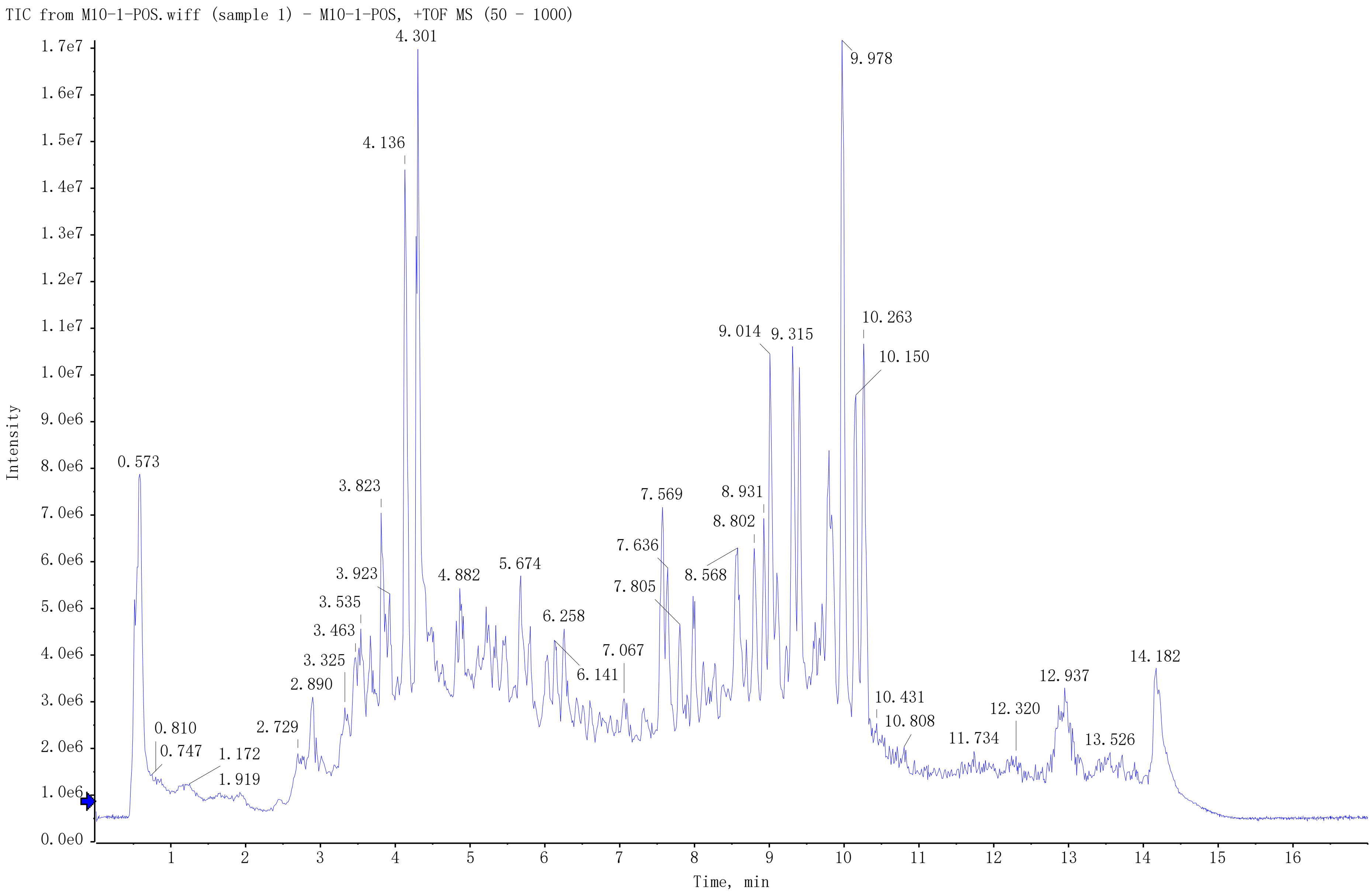
TIC from M10-1-POS.wiff (sample 1) - M10-1-POS, +TOF MS (50 - 1000)
Intensity
Time, min
0.573
0.810
0.747
1.172
1.919
2.729
2.890
3.325
3.463
3.535
3.823
3.923
4.136
4.301
4.882
5.674
6.258
6.141
7.067
7.569
7.636
7.805
8.568
8.802
8.931
9.014
9.315
9.978
10.263
10.150
10.431
10.808
11.734
12.320
12.937
13.526
14.182

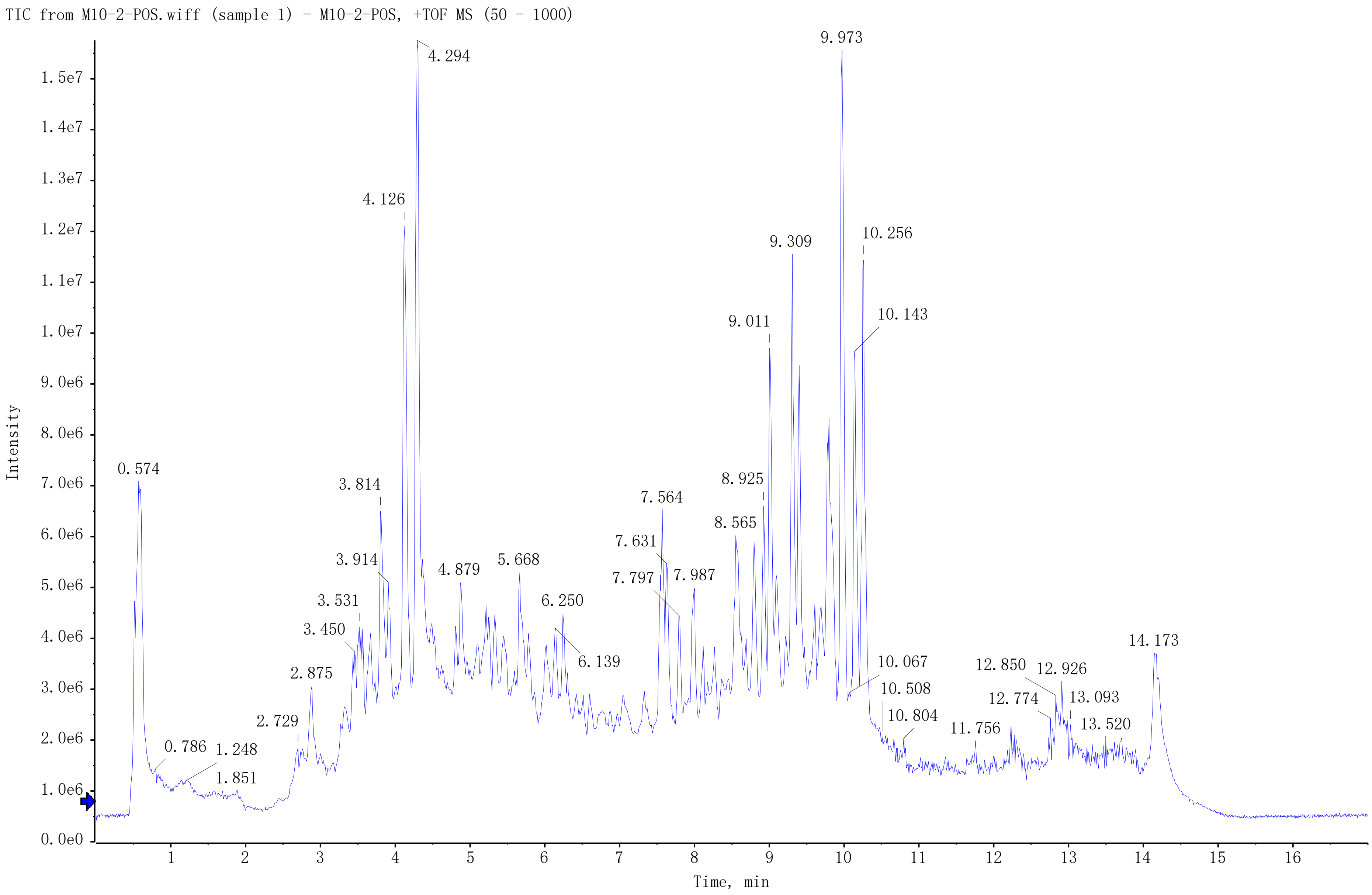

TIC from M10-2-POS.wiff (sample 1) - M10-2-POS, +TOF MS (50 - 1000)
Intensity
Time, min
0.574
0.786
1.248
1.851
2.729
2.875
3.450
3.531
3.814
3.914
4.126
4.294
4.879
5.668
6.250
6.139
7.564
7.631
7.797
7.987
8.565
8.925
9.011
9.309
9.973
10.256
10.143
10.067
10.508
10.804
11.756
12.850
12.926
12.774
13.093
13.520
14.173

TIC from M10-3-POS.wiff (sample 1) - M10-3-POS, +TOF MS (50 - 1000)

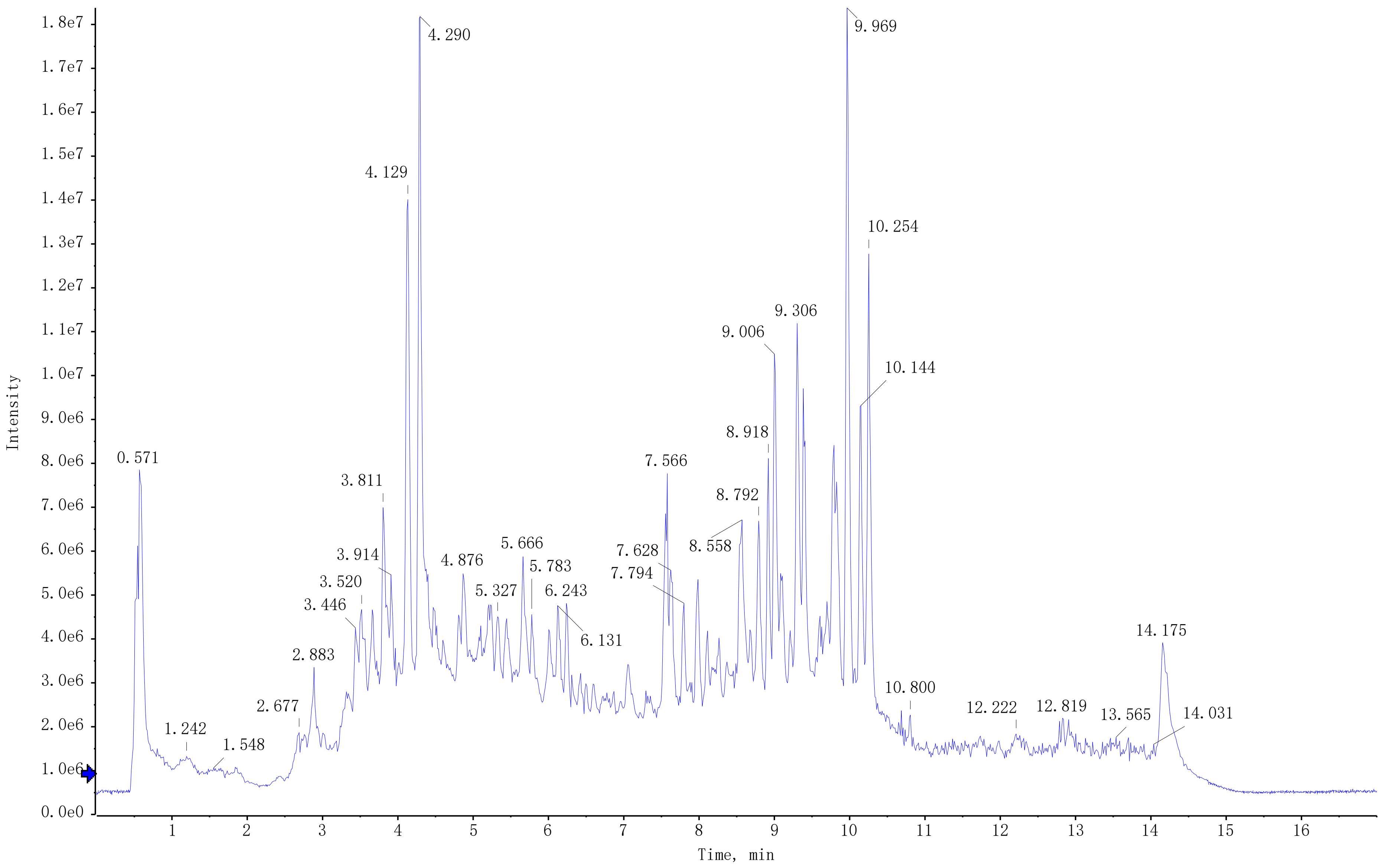

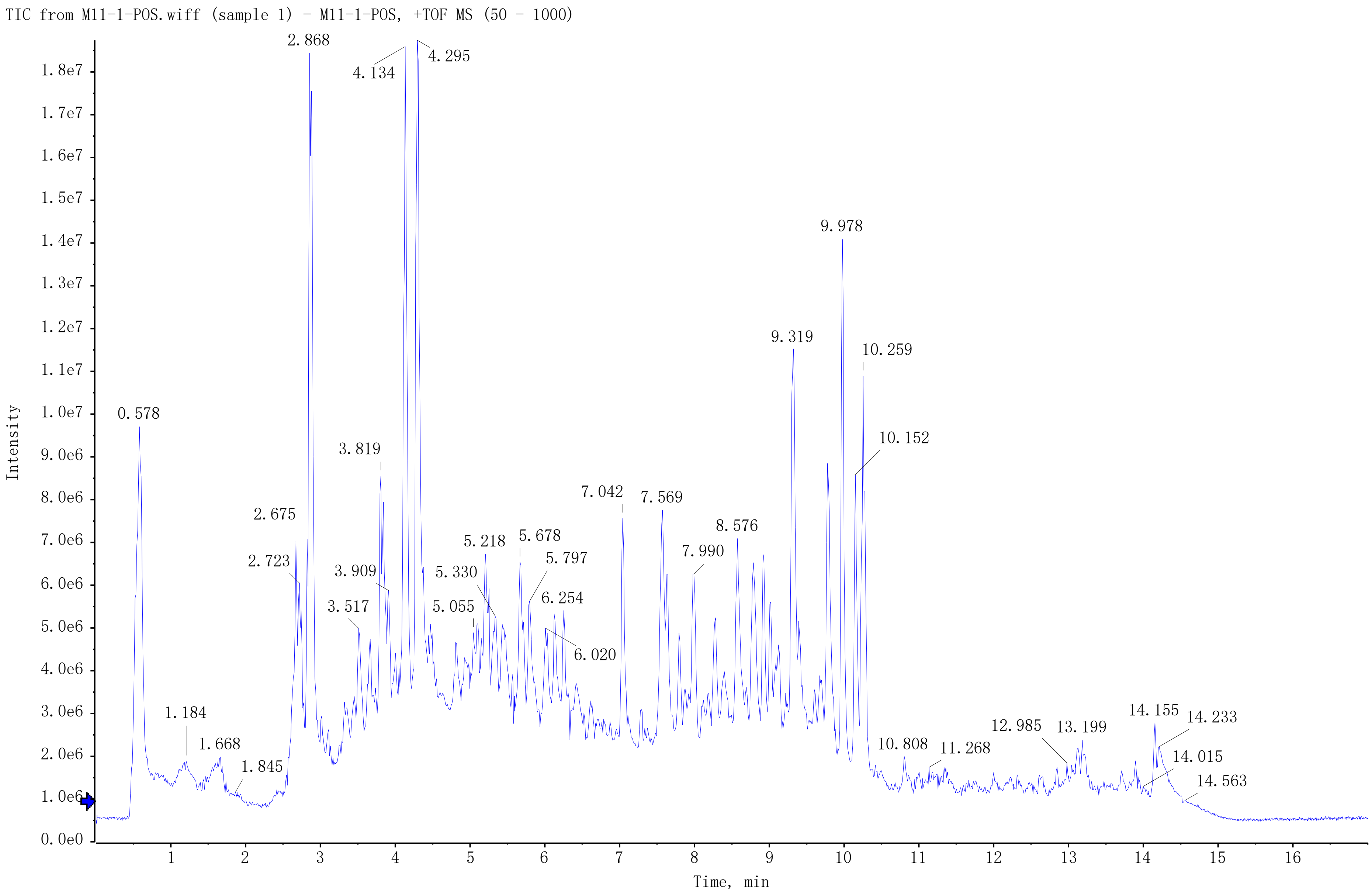
TIC from M11-1-POS.wiff (sample 1) - M11-1-POS, +TOF MS (50 - 1000)
Intensity
Time, min
0.578
1.184
1.668
1.845
2.675
2.723
2.868
3.517
3.819
3.909
4.134
4.295
5.055
5.218
5.330
5.678
5.797
6.020
6.254
7.042
7.569
7.990
8.576
9.319
9.978
10.152
10.259
10.808
11.268
12.985
13.199
14.015
14.155
14.233
14.563

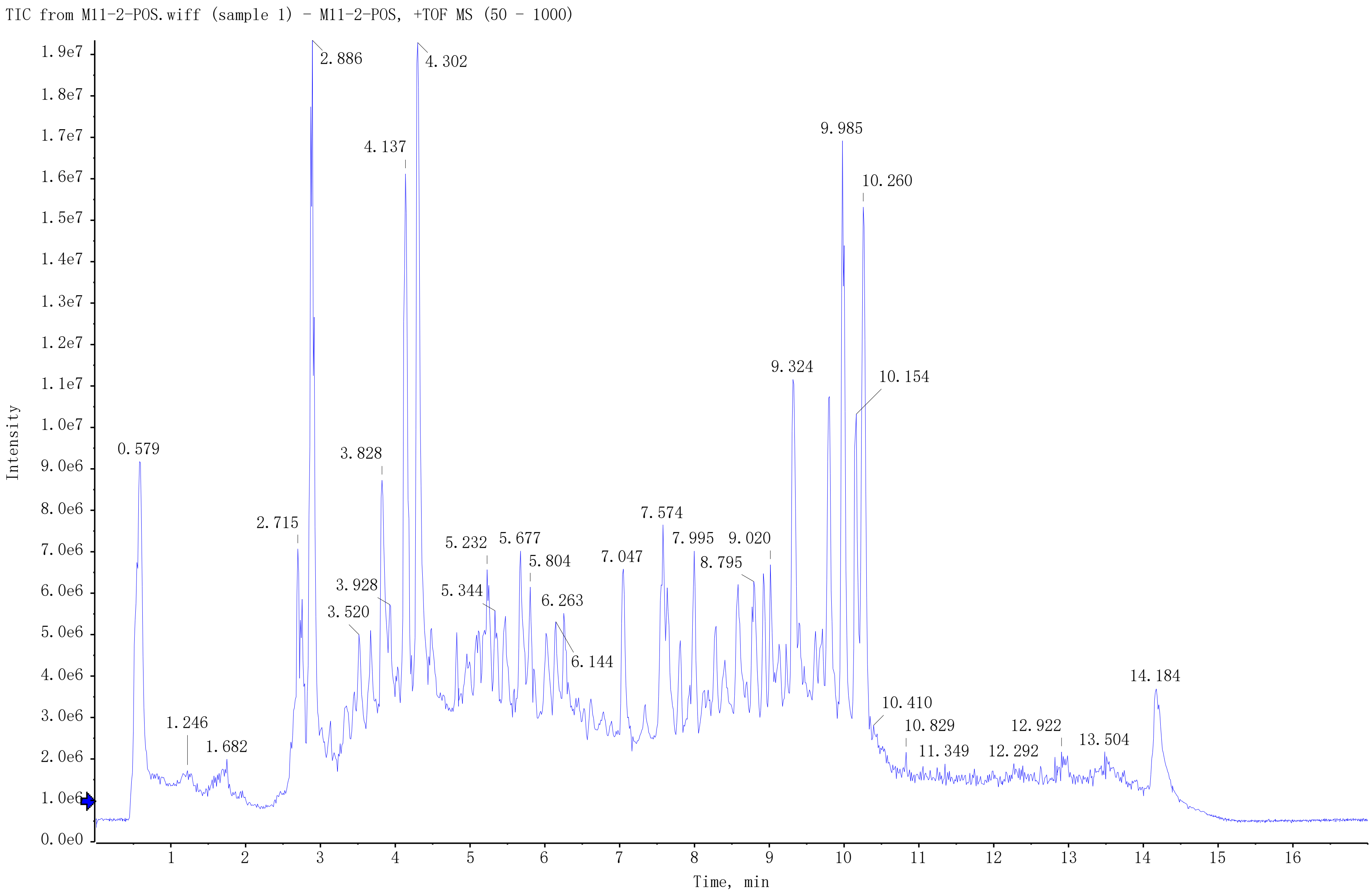
TIC from M11-2-POS.wiff (sample 1) - M11-2-POS, +TOF MS (50 - 1000)
Intensity
Time, min
0.579
1.246
1.682
2.715
2.886
3.520
3.828
3.928
4.137
4.302
5.232
5.344
5.677
5.804
6.144
6.263
7.047
7.574
7.995
8.795
9.020
9.324
9.985
10.154
10.260
10.410
10.829
11.349
12.292
12.922
13.504
14.184

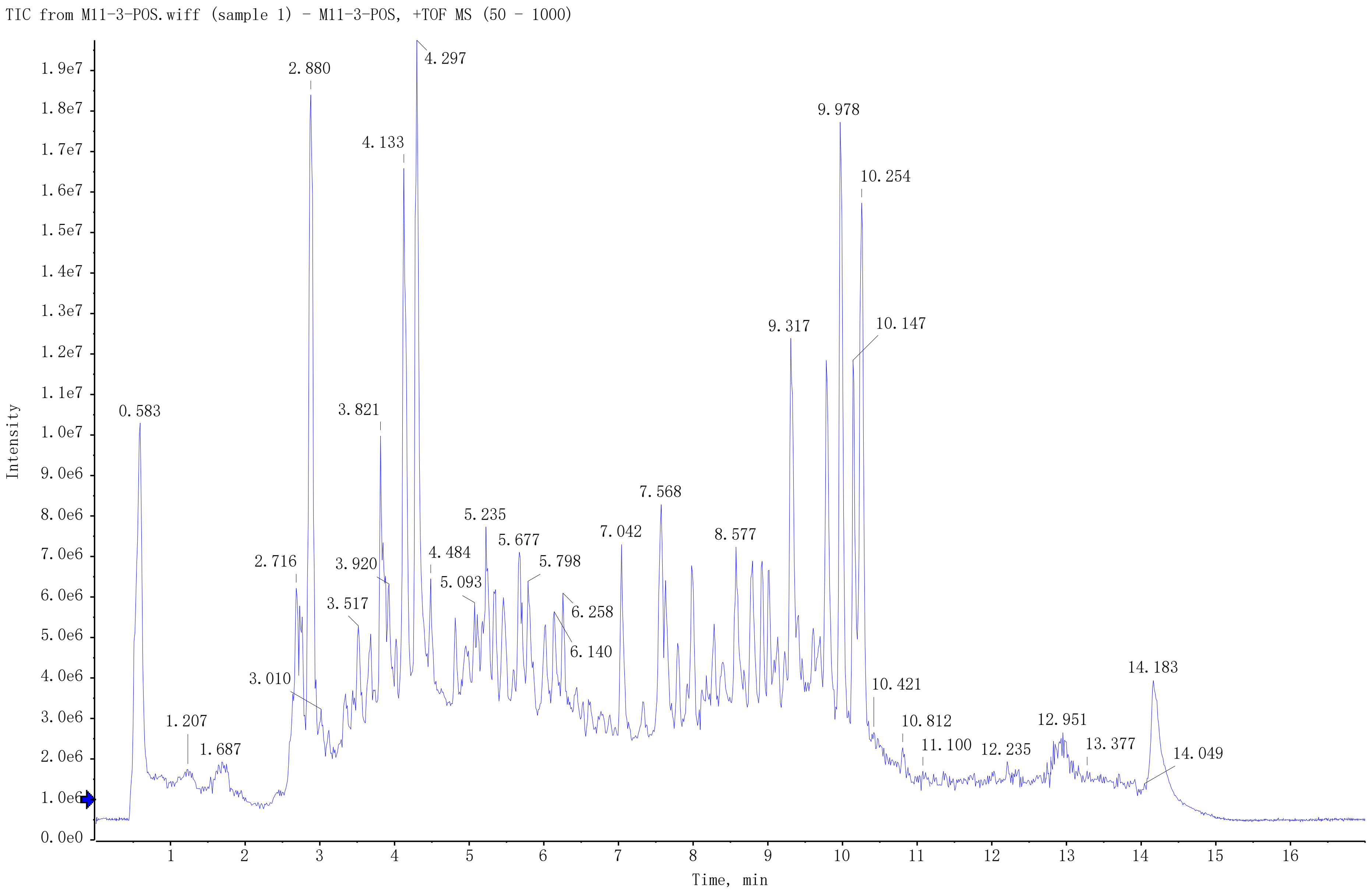
TIC from M11-3-POS.wiff (sample 1) - M11-3-POS, +TOF MS (50 - 1000)
Intensity
Time, min
0.583
1.207
1.687
2.716
2.880
3.010
3.517
3.821
3.920
4.133
4.297
4.484
5.093
5.235
5.677
5.798
6.140
6.258
7.042
7.568
8.577
9.317
9.978
10.147
10.254
10.421
10.812
11.100
12.235
12.951
13.377
14.049
14.183

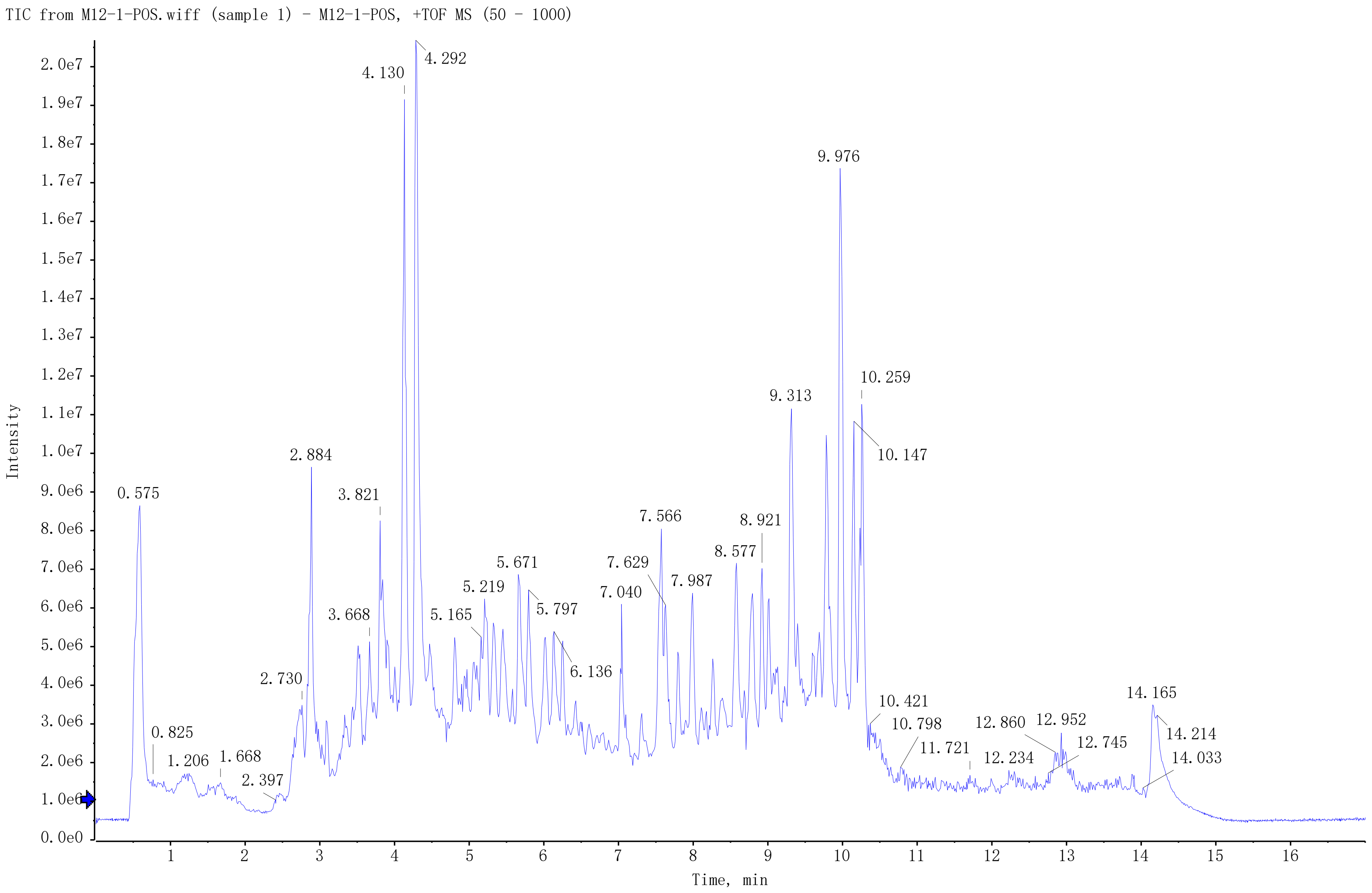
TIC from M12-1-POS.wiff (sample 1) - M12-1-POS, +TOF MS (50 - 1000)
Intensity
Time, min
0.575
0.825
1.206
1.668
2.397
2.730
2.884
3.668
3.821
4.130
4.292
5.165
5.219
5.671
5.797
6.136
7.040
7.629
7.566
7.987
8.577
8.921
9.313
9.976
10.259
10.147
10.421
10.798
11.721
12.234
12.860
12.952
12.745
14.165
14.214
14.033

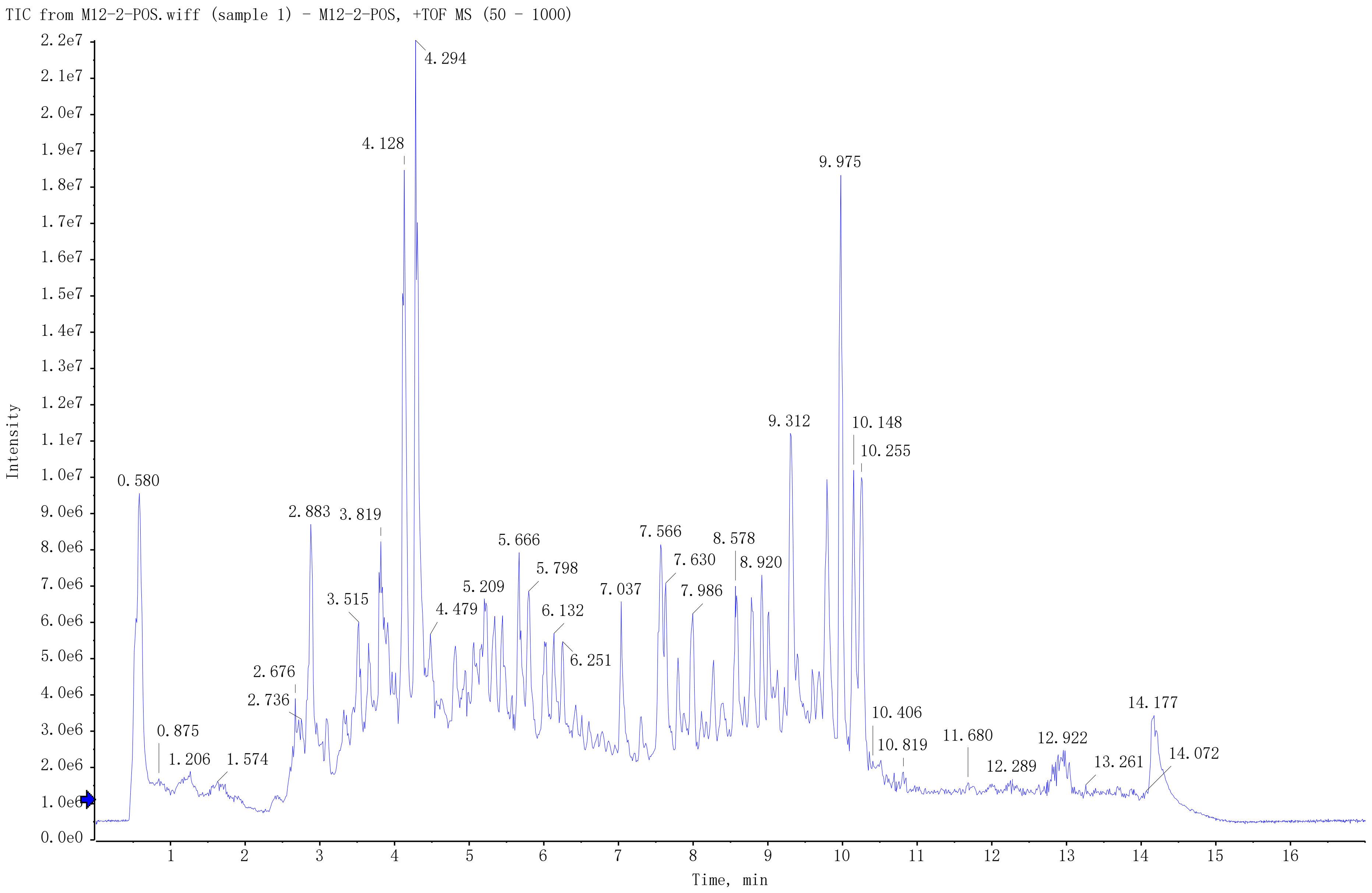
TIC from M12-2-POS.wiff (sample 1) - M12-2-POS, +TOF MS (50 - 1000)
Intensity
Time, min
4.294
4.128
9.975
9.312
10.148
10.255
0.580
2.883
3.819
5.666
7.566
8.578
5.798
7.630
8.920
5.209
7.037
7.986
3.515
4.479
6.132
6.251
2.676
2.736
0.875
1.206
1.574
10.406
10.819
11.680
12.289
12.922
13.261
14.177
14.072

TIC from M12-3-POS.wiff (sample 1) - M12-3-POS, +TOF MS (50 - 1000)

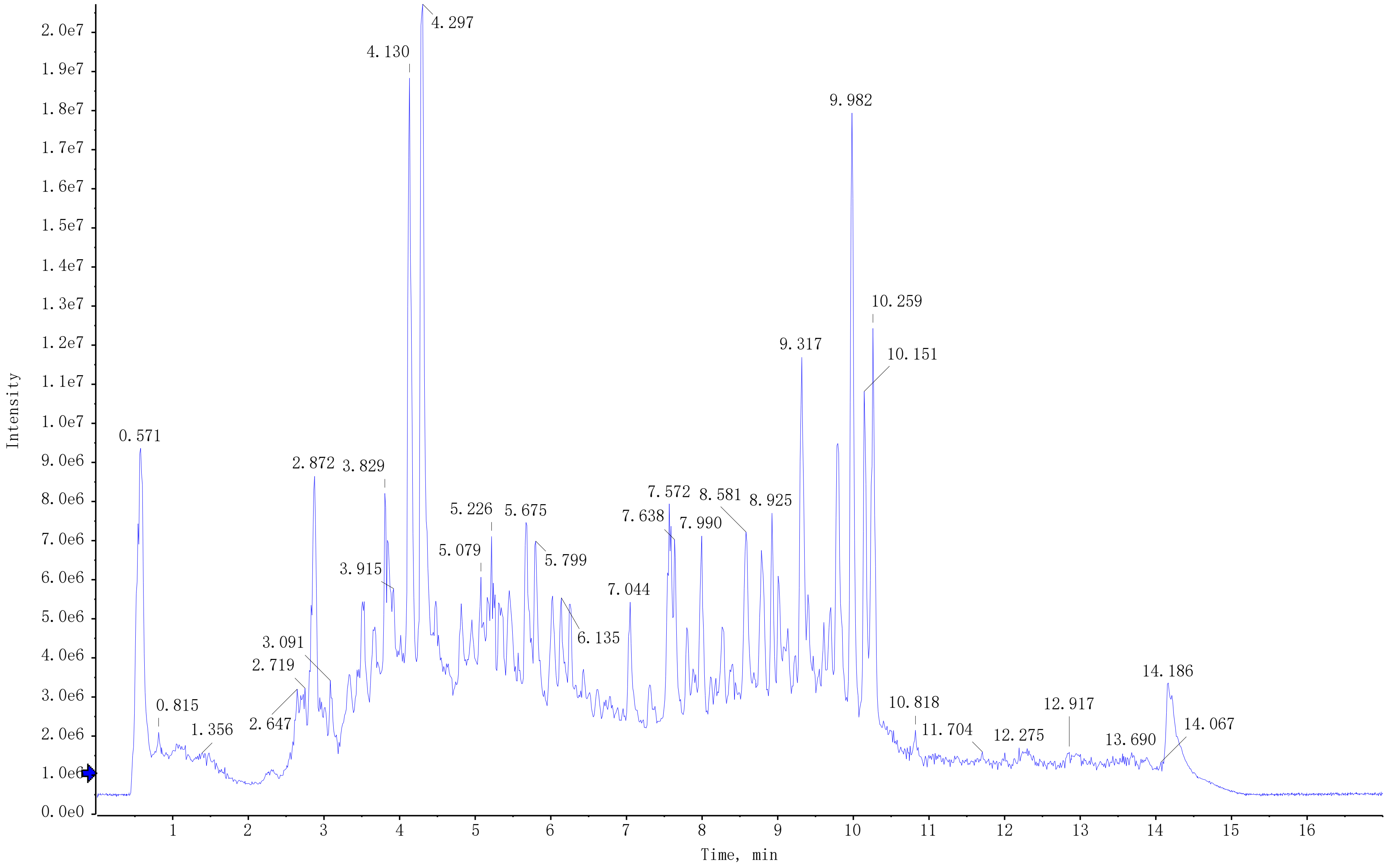

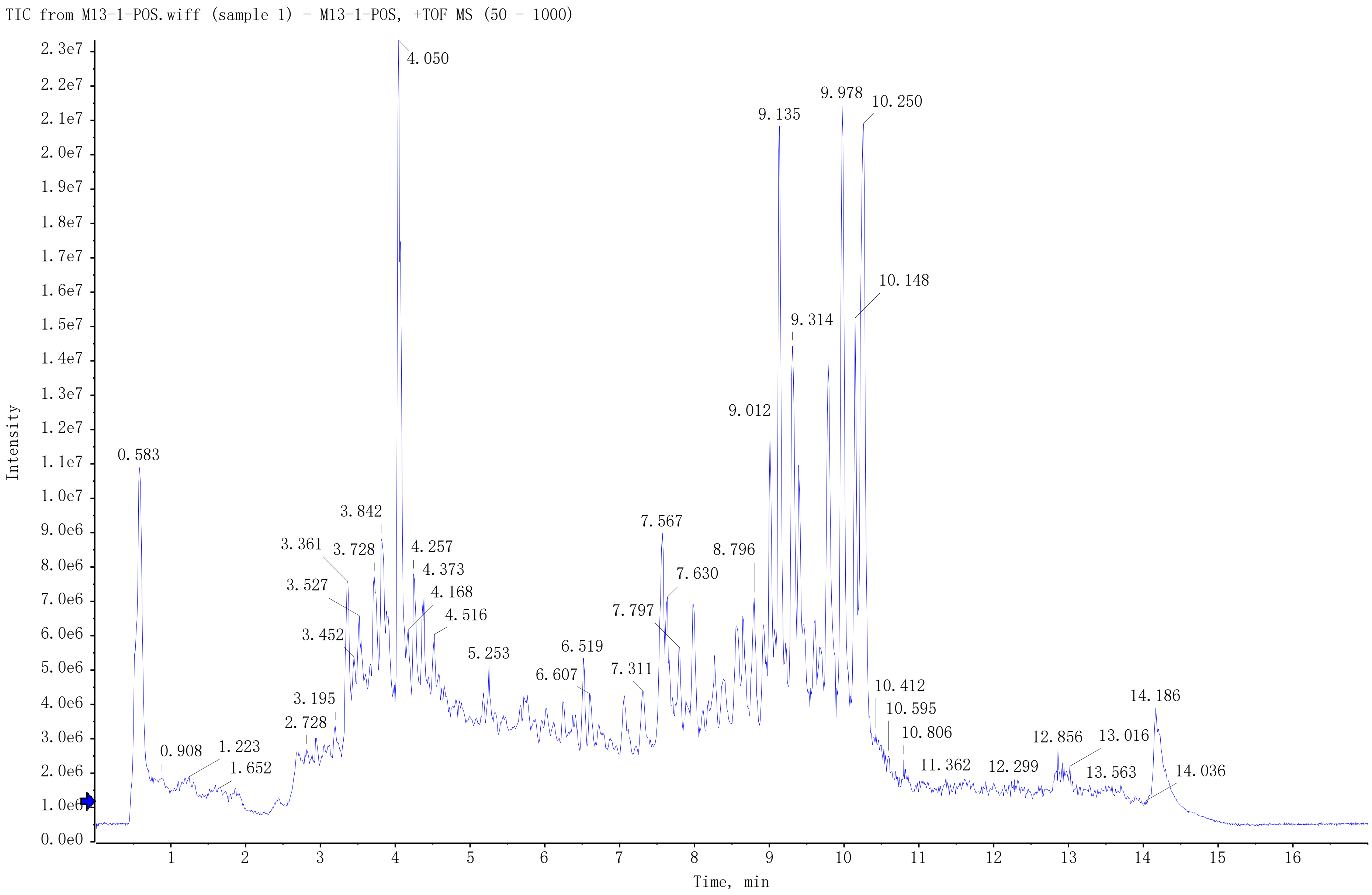
TIC from M13-1-POS.wiff (sample 1) - M13-1-POS, +TOF MS (50 - 1000)
Intensity
Time, min
0.583
0.908
1.223
1.652
2.728
3.195
3.361
3.452
3.527
3.728
3.842
4.050
4.168
4.257
4.373
4.516
5.253
6.519
6.607
7.311
7.567
7.630
7.797
8.796
9.012
9.135
9.314
9.978
10.148
10.250
10.412
10.595
10.806
11.362
12.299
12.856
13.016
13.563
14.036
14.186

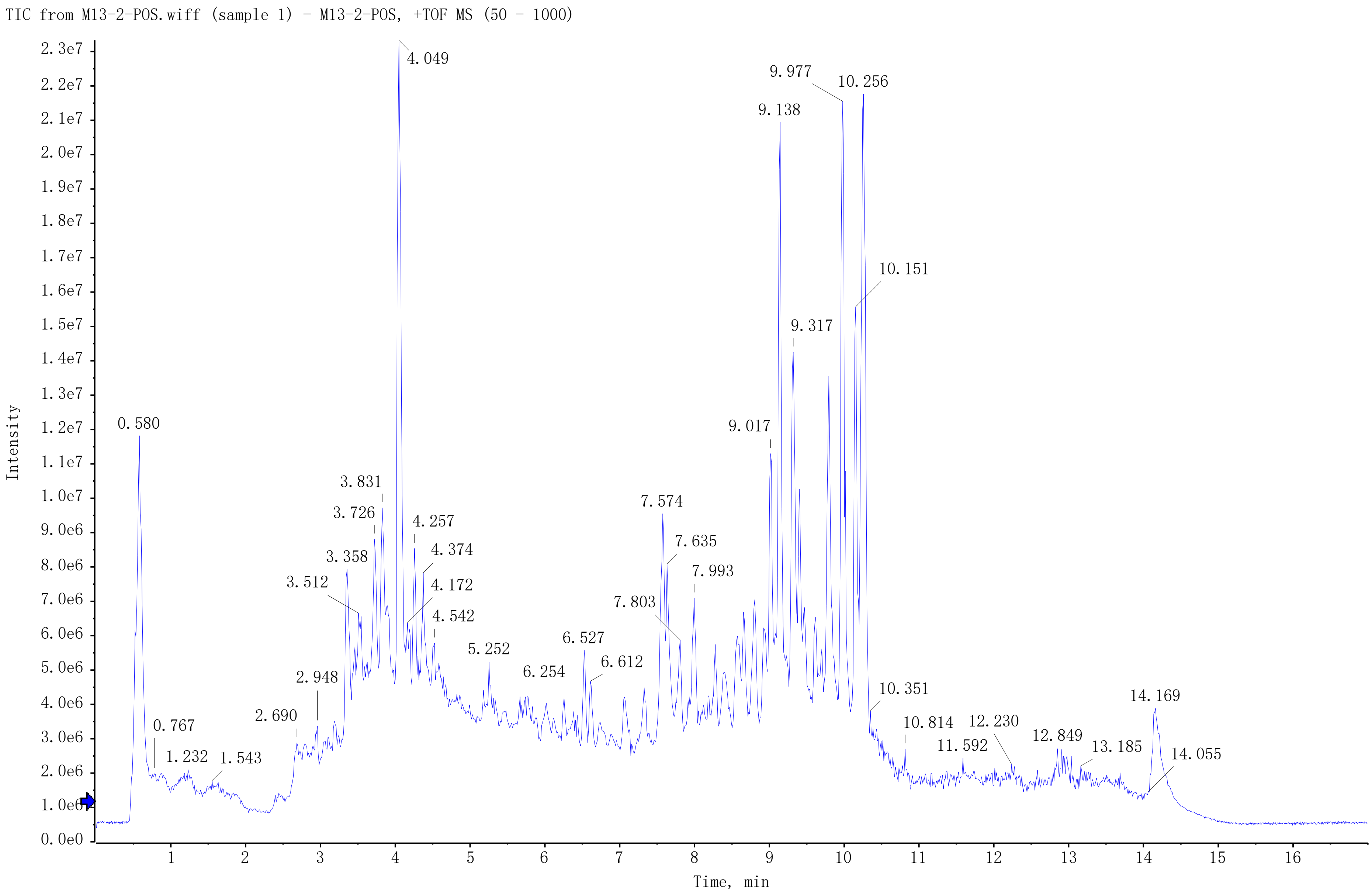

TIC from M13-2-POS.wiff (sample 1) - M13-2-POS, +TOF MS (50 - 1000)
Intensity
Time, min
0.580
0.767
1.232
1.543
2.690
2.948
3.358
3.512
3.726
3.831
4.049
4.172
4.257
4.374
4.542
5.252
6.254
6.527
6.612
7.574
7.635
7.803
7.993
9.017
9.138
9.317
9.977
10.151
10.256
10.351
10.814
11.592
12.230
12.849
13.185
14.055
14.169

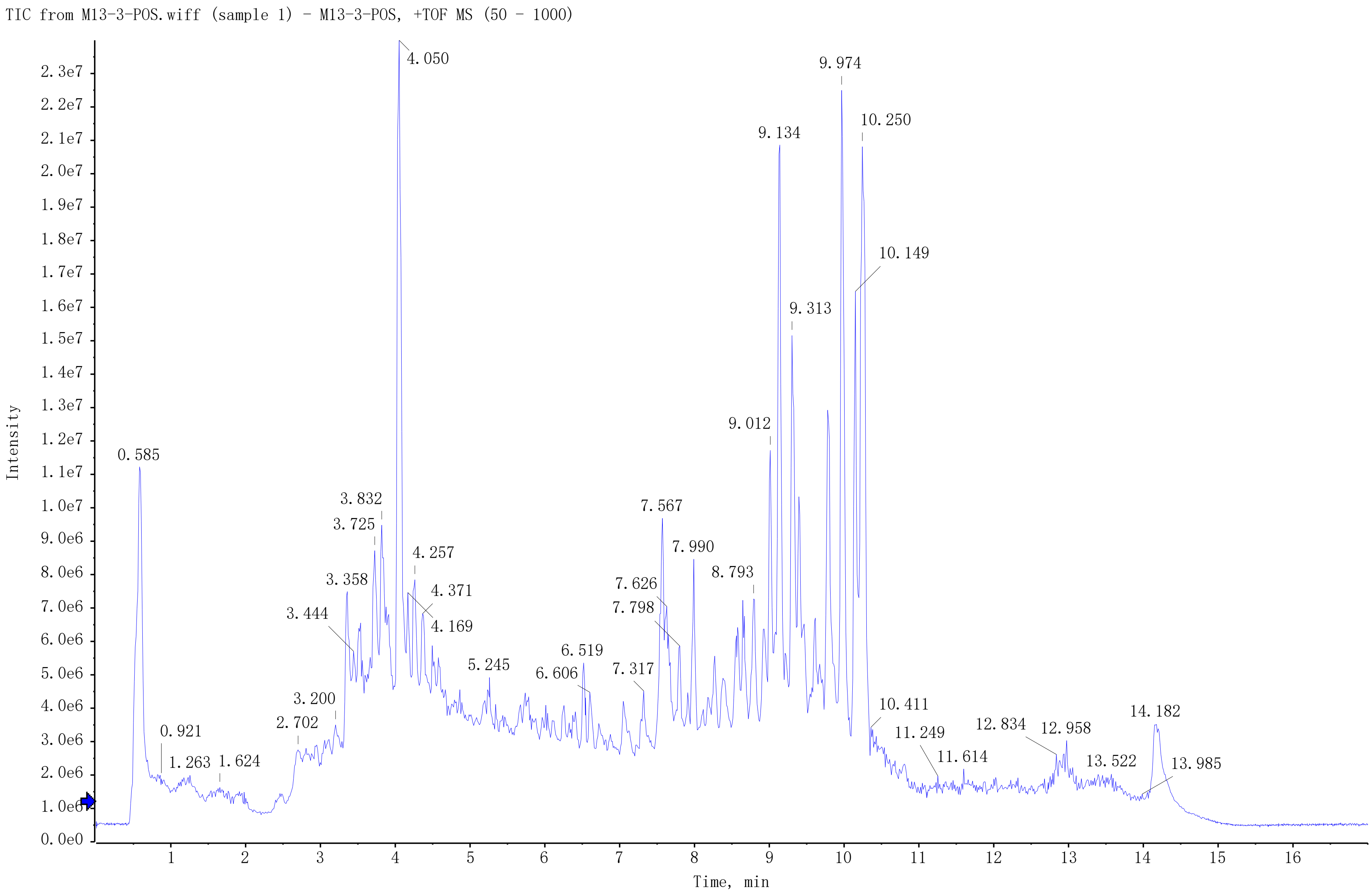
TIC from M13-3-POS.wiff (sample 1) - M13-3-POS, +TOF MS (50 - 1000)
Intensity
Time, min
4.050
9.974
10.250
9.134
10.149
9.313
9.012
0.585
3.832
3.725
7.567
7.990
4.257
8.793
3.358
4.371
7.626
3.444
4.169
7.798
6.519
5.245
6.606
7.317
3.200
10.411
2.702
0.921
11.249
12.834
12.958
14.182
11.614
1.263
1.624
13.522
13.985

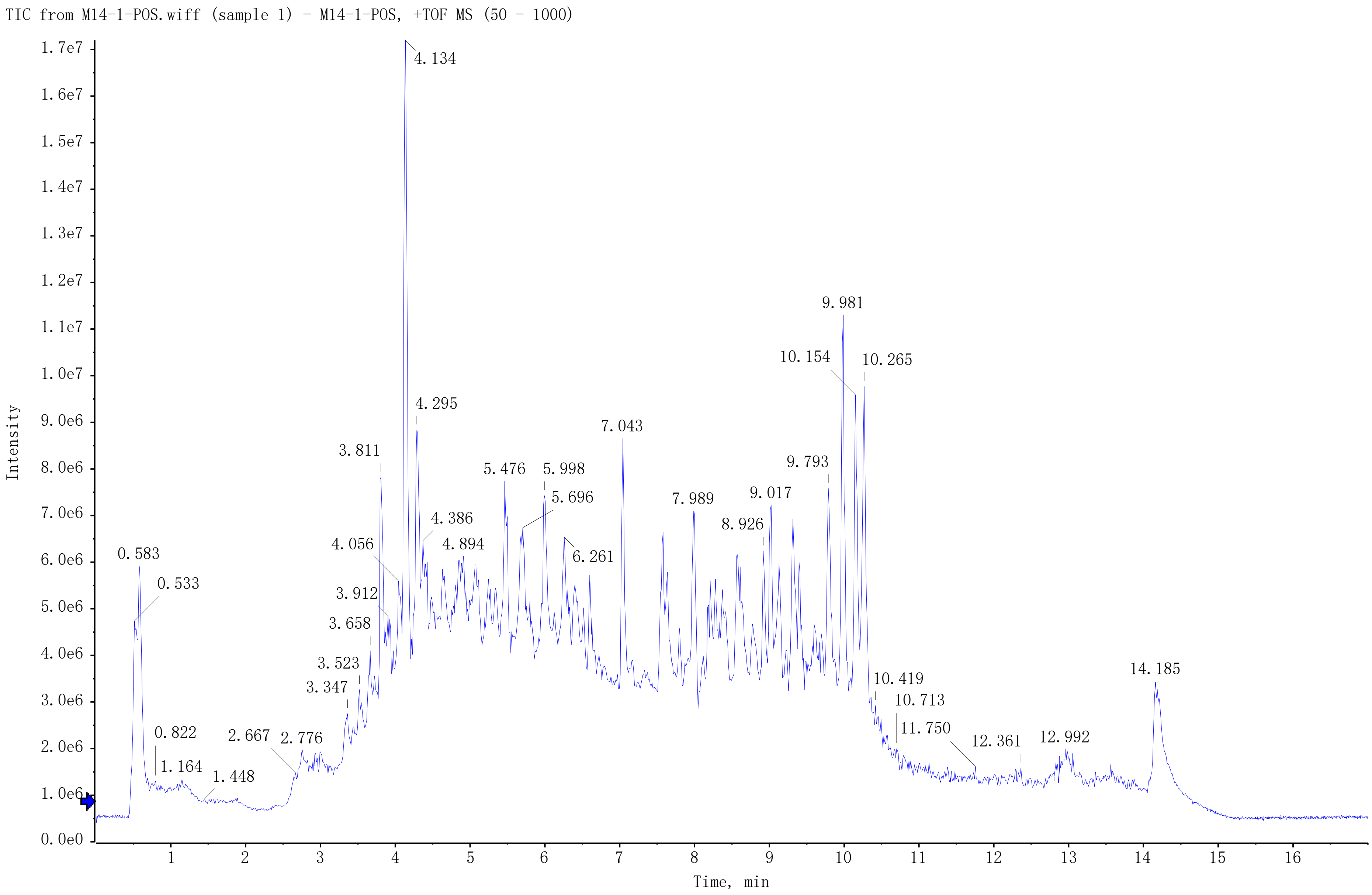

TIC from M14-1-POS.wiff (sample 1) - M14-1-POS, +TOF MS (50 - 1000)
Intensity
Time, min
0.0e0
1.0e6
2.0e6
3.0e6
4.0e6
5.0e6
6.0e6
7.0e6
8.0e6
9.0e6
1.0e7
1.1e7
1.2e7
1.3e7
1.4e7
1.5e7
1.6e7
1.7e7
1
2
3
4
5
6
7
8
9
10
11
12
13
14
15
16
4.134
9.981
10.154
10.265
4.295
7.043
3.811
5.476
5.998
9.793
5.696
7.989
9.017
4.386
8.926
4.056
4.894
0.583
6.261
0.533
3.912
3.658
3.523
3.347
10.419
10.713
14.185
11.750
12.361
12.992
0.822
2.667
2.776
1.164
1.448

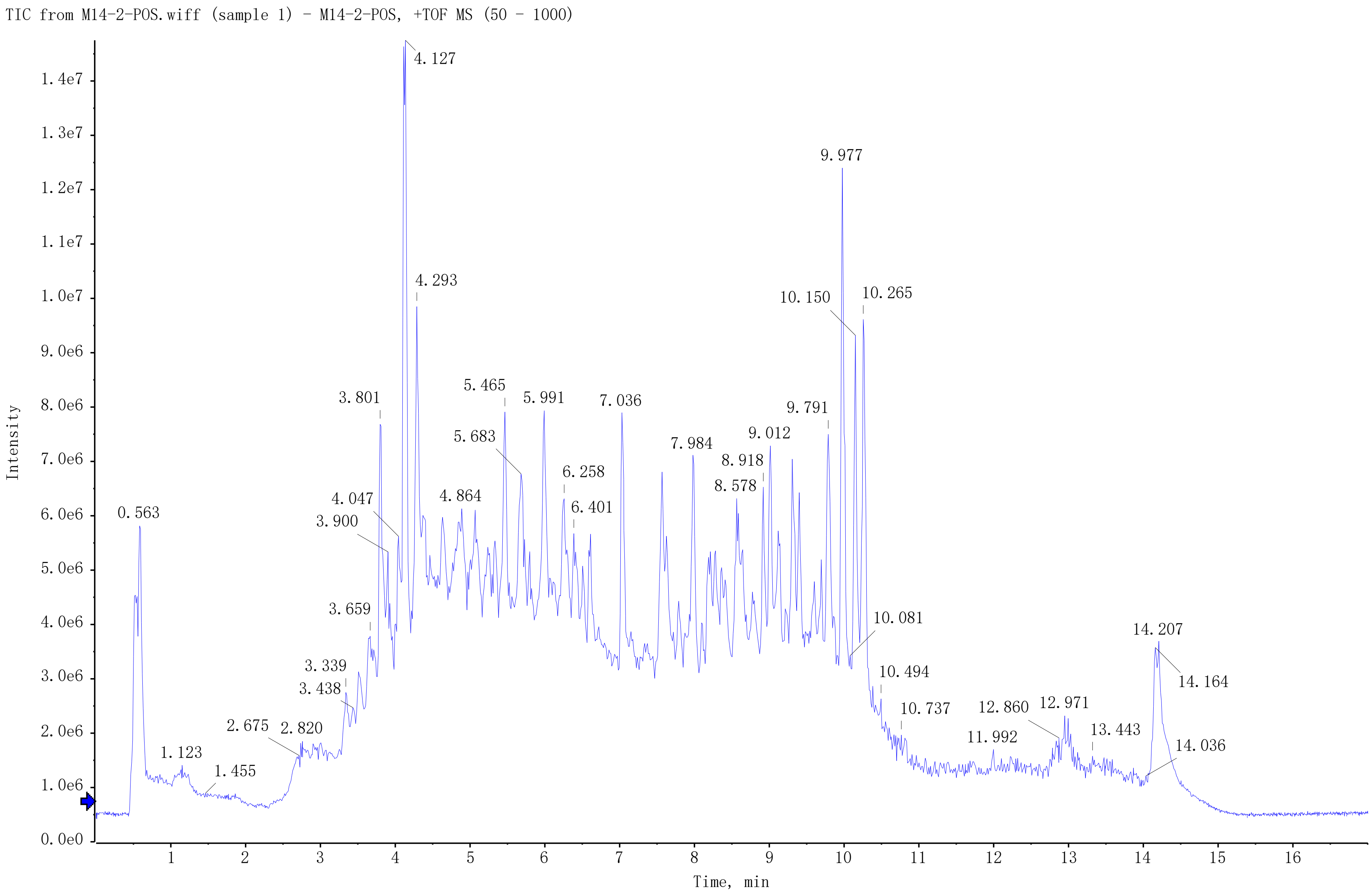
TIC from M14-2-POS.wiff (sample 1) - M14-2-POS, +TOF MS (50 - 1000)
Intensity
Time, min
1.4e7
1.3e7
1.2e7
1.1e7
1.0e7
9.0e6
8.0e6
7.0e6
6.0e6
5.0e6
4.0e6
3.0e6
2.0e6
1.0e6
0.0e0
1
2
3
4
5
6
7
8
9
10
11
12
13
14
15
16
4.127
9.977
4.293
10.150
10.265
5.465
5.991
3.801
7.036
9.791
5.683
7.984
9.012
8.918
6.258
8.578
4.864
6.401
4.047
0.563
3.900
3.659
10.081
14.207
3.339
10.494
14.164
3.438
10.737
12.860
12.971
2.675
2.820
13.443
11.992
1.123
14.036
1.455

TIC from M14-3-POS.wiff (sample 1) - M14-3-POS, +TOF MS (50 - 1000)

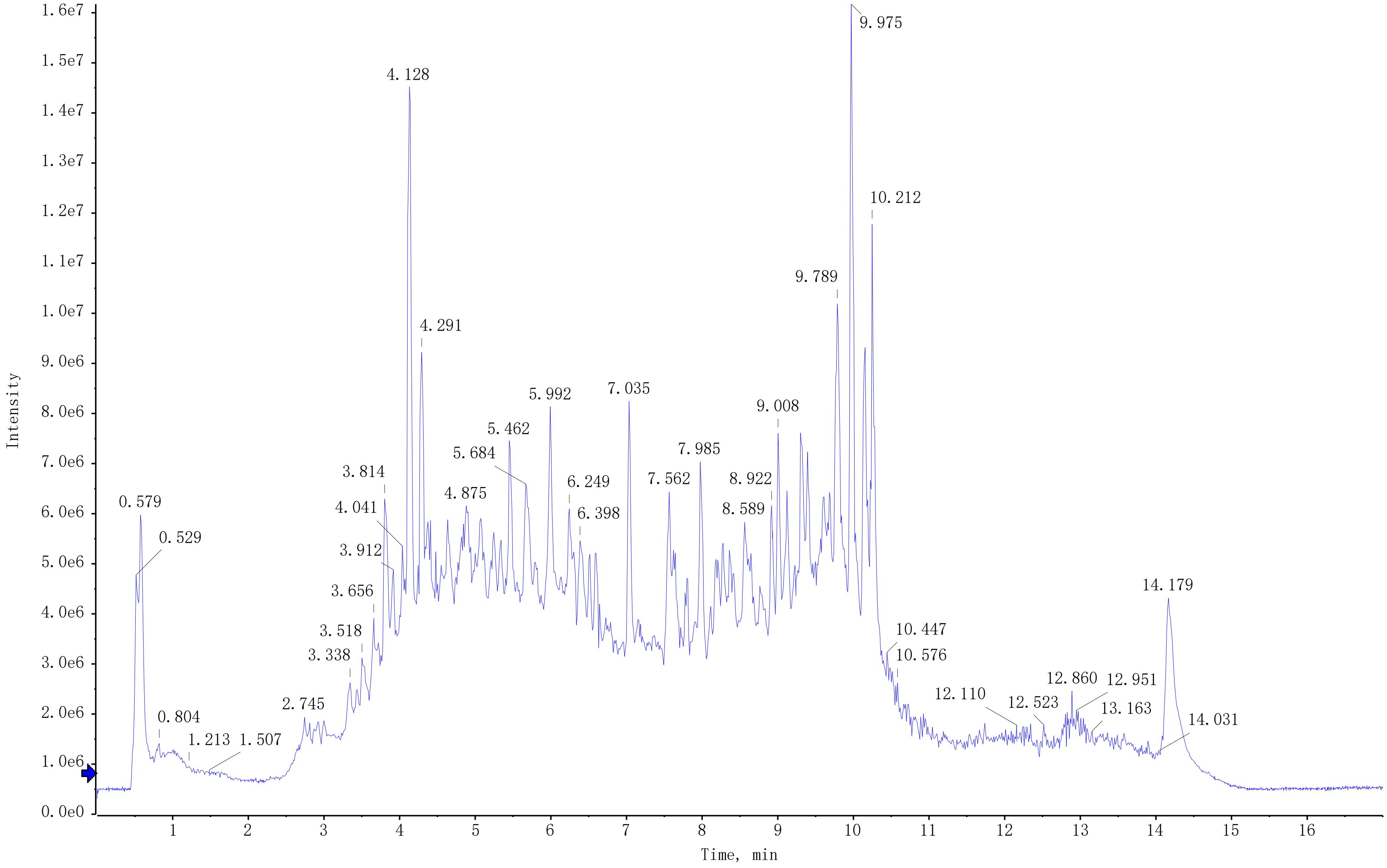

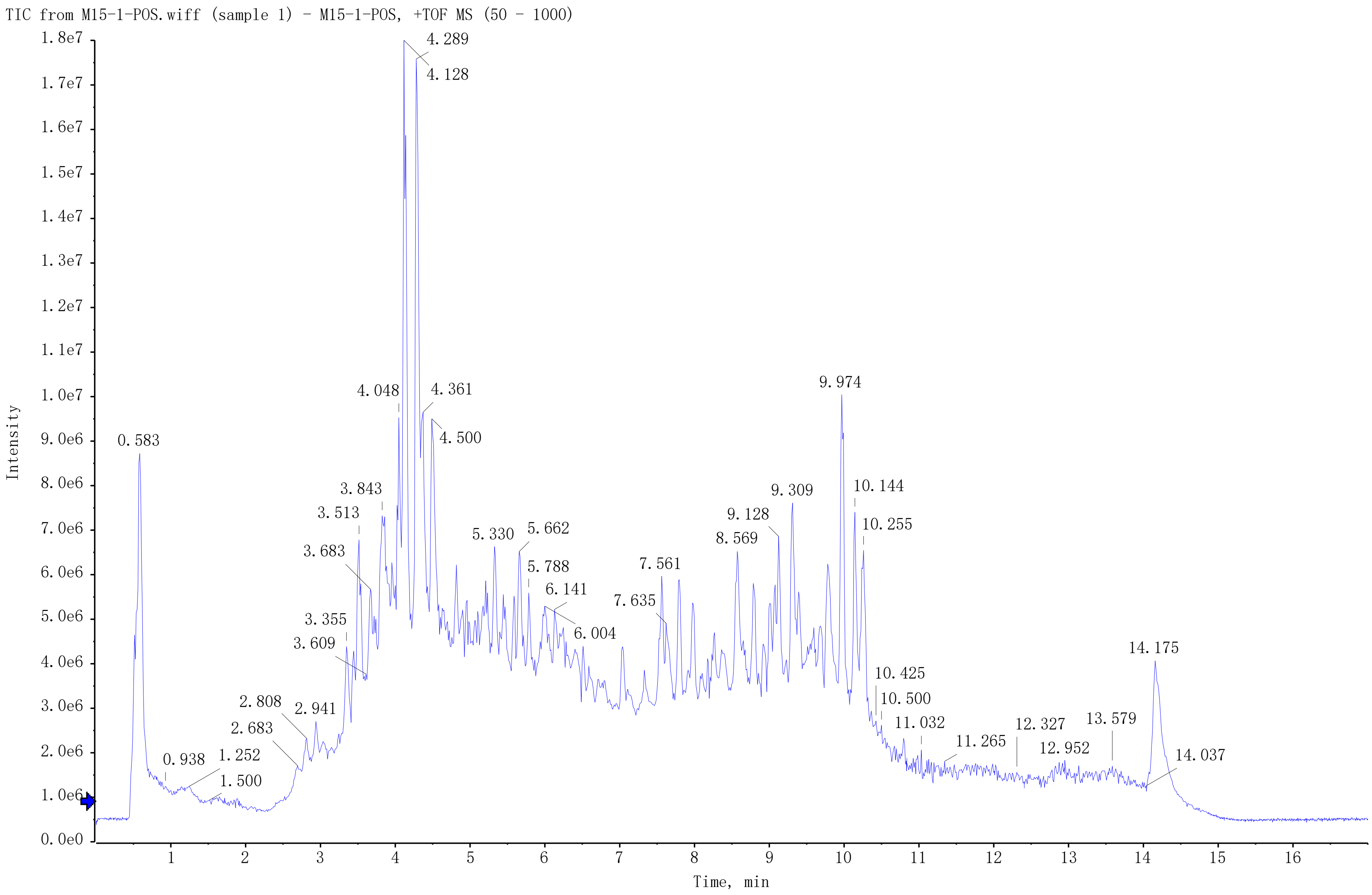

TIC from M15-1-POS.wiff (sample 1) - M15-1-POS, +TOF MS (50 - 1000)
Intensity
Time, min
0.583
0.938
1.252
1.500
2.683
2.808
2.941
3.355
3.609
3.683
3.513
3.843
4.048
4.289
4.128
4.361
4.500
5.330
5.662
5.788
6.141
6.004
7.635
7.561
8.569
9.128
9.309
9.974
10.144
10.255
10.425
10.500
11.032
11.265
12.327
12.952
13.579
14.037
14.175

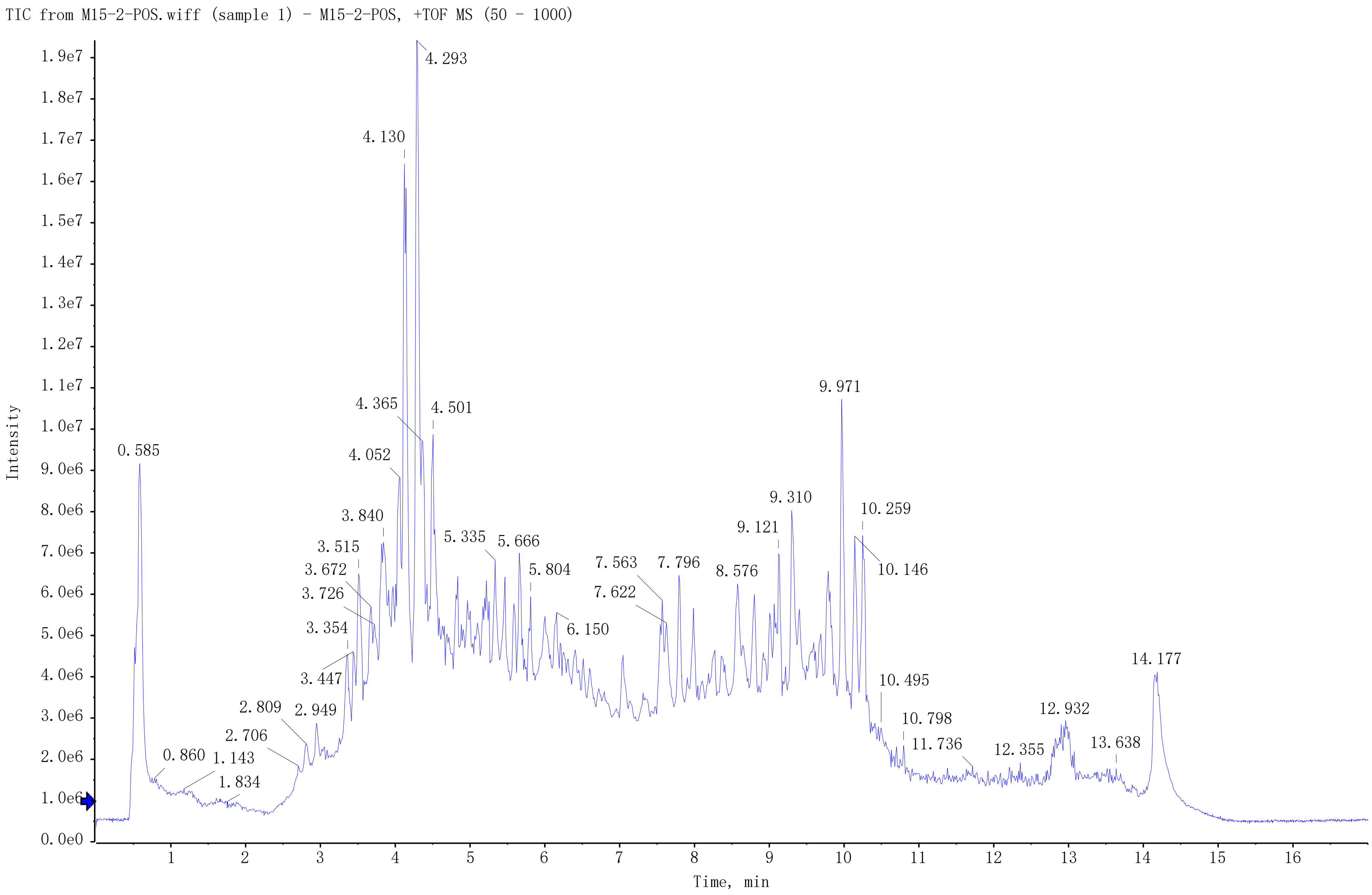

TIC from M15-2-POS.wiff (sample 1) - M15-2-POS, +TOF MS (50 - 1000)
Intensity
Time, min
0.585
0.860
1.143
1.834
2.706
2.809
2.949
3.354
3.447
3.515
3.672
3.726
3.840
4.052
4.130
4.293
4.365
4.501
5.335
5.666
5.804
6.150
7.563
7.622
7.796
8.576
9.121
9.310
9.971
10.146
10.259
10.495
10.798
11.736
12.355
12.932
13.638
14.177

TIC from M15-3-POS.wiff (sample 1) - M15-3-POS, +TOF MS (50 - 1000)

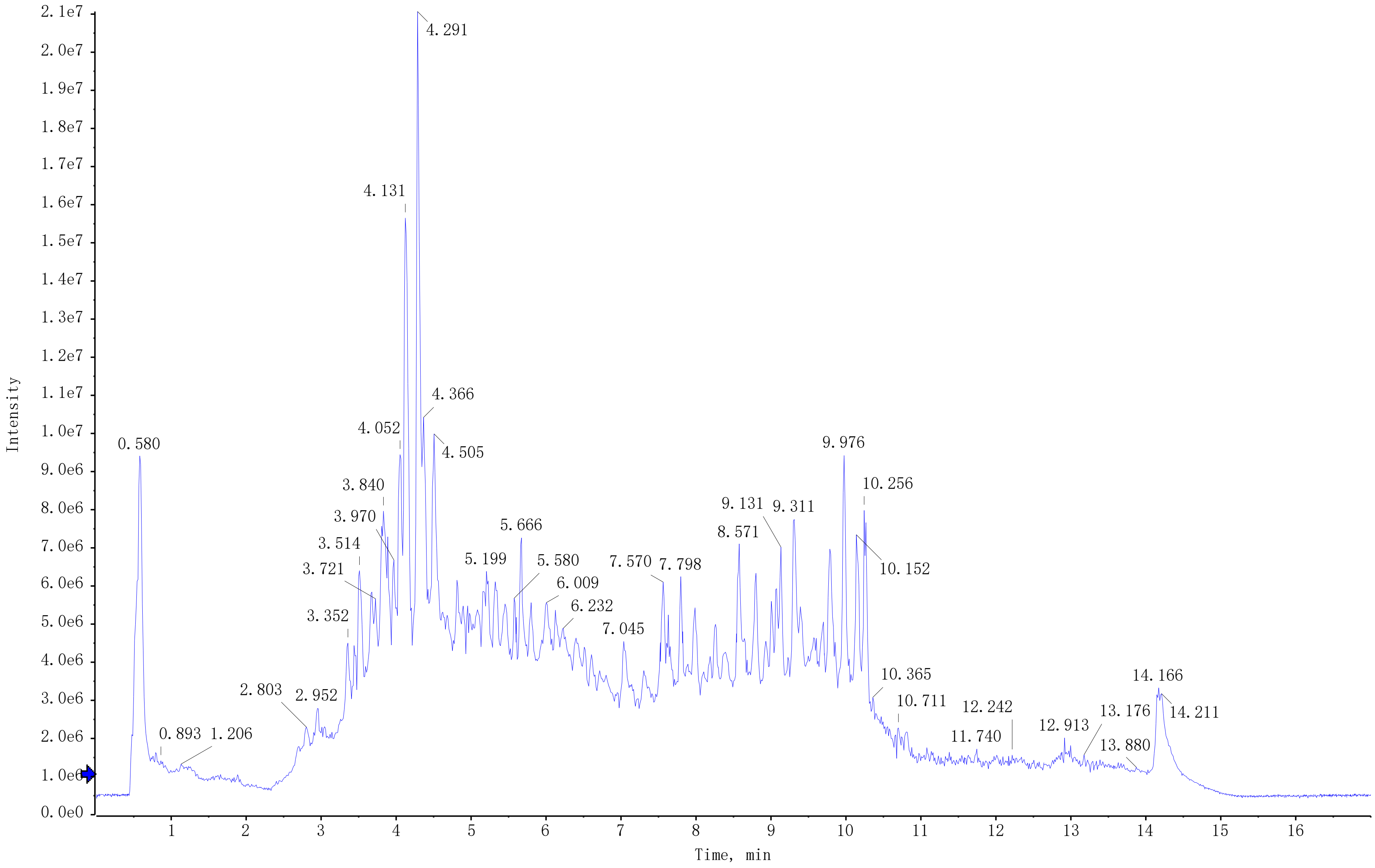

TIC from QC1-W-POS.wiff (sample 1) - QC1-POS, +TOF MS (50 - 1000)

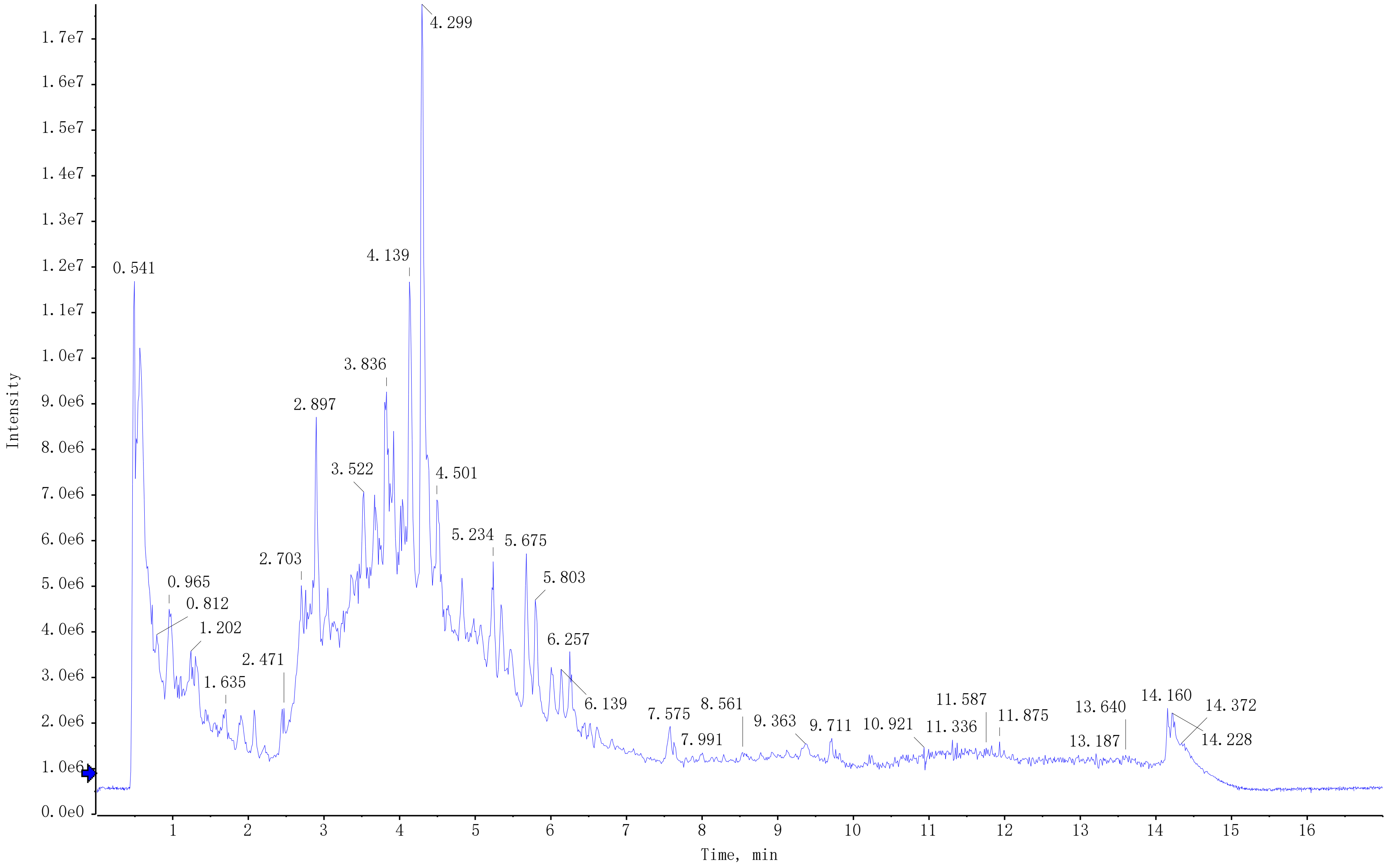

TIC from QC2-W-POS.wiff (sample 1) - QC2-POS, +TOF MS (50 - 1000)

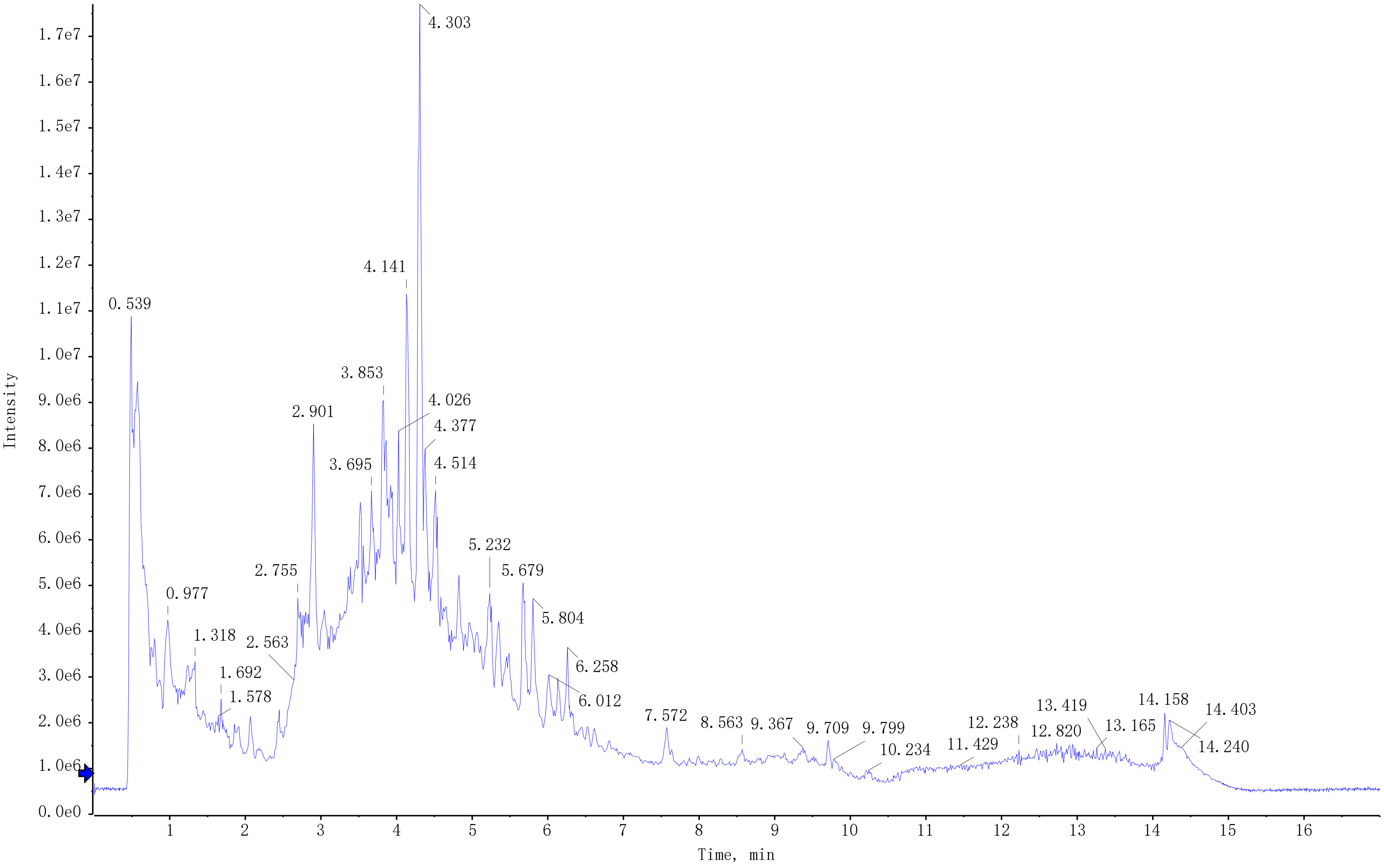

TIC from QC3-W-POS.wiff (sample 1) - QC3-POS, +TOF MS (50 - 1000)

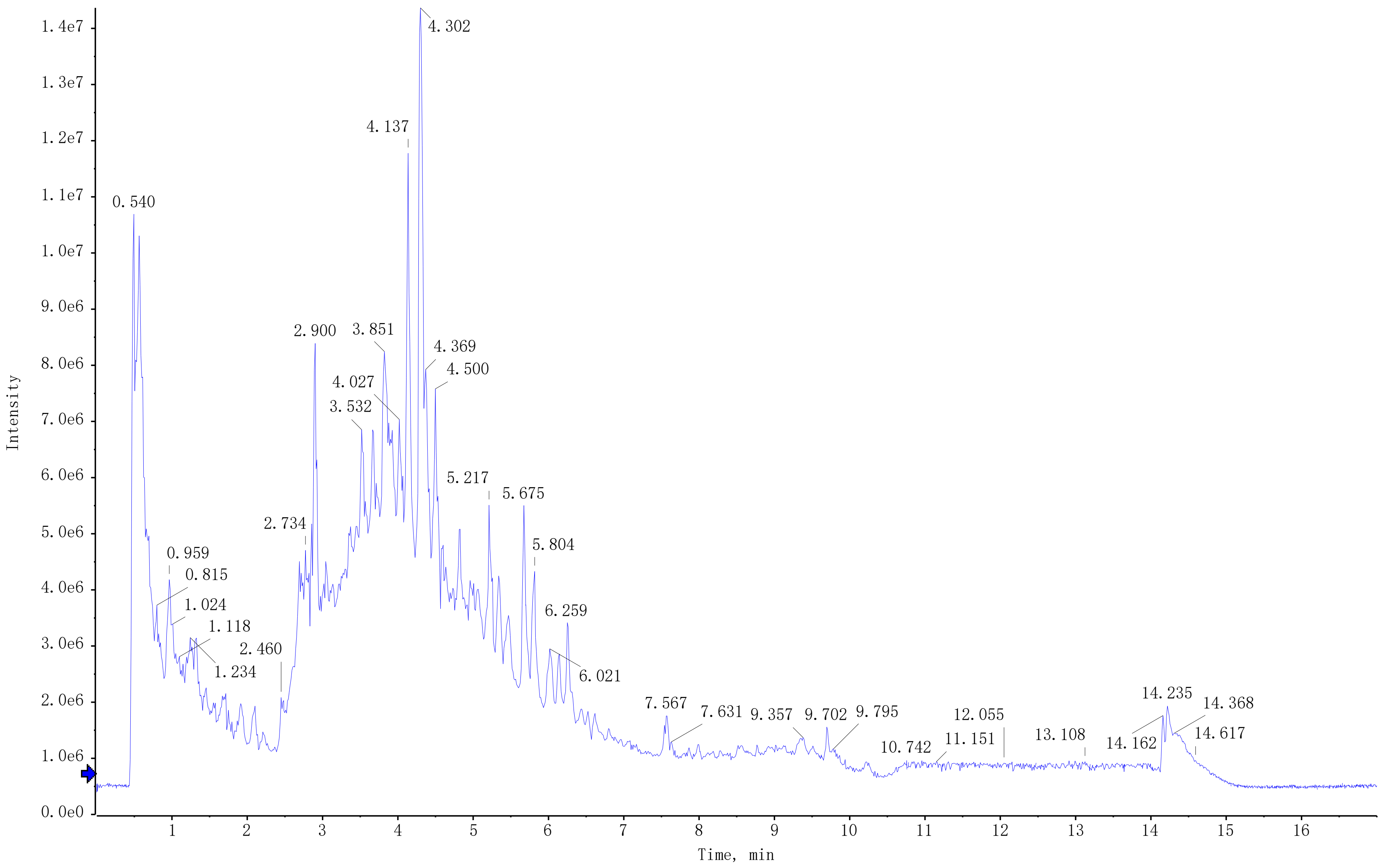

TIC from QC4-W-POS.wiff (sample 1) - QC4-POS, +TOF MS (50 - 1000)

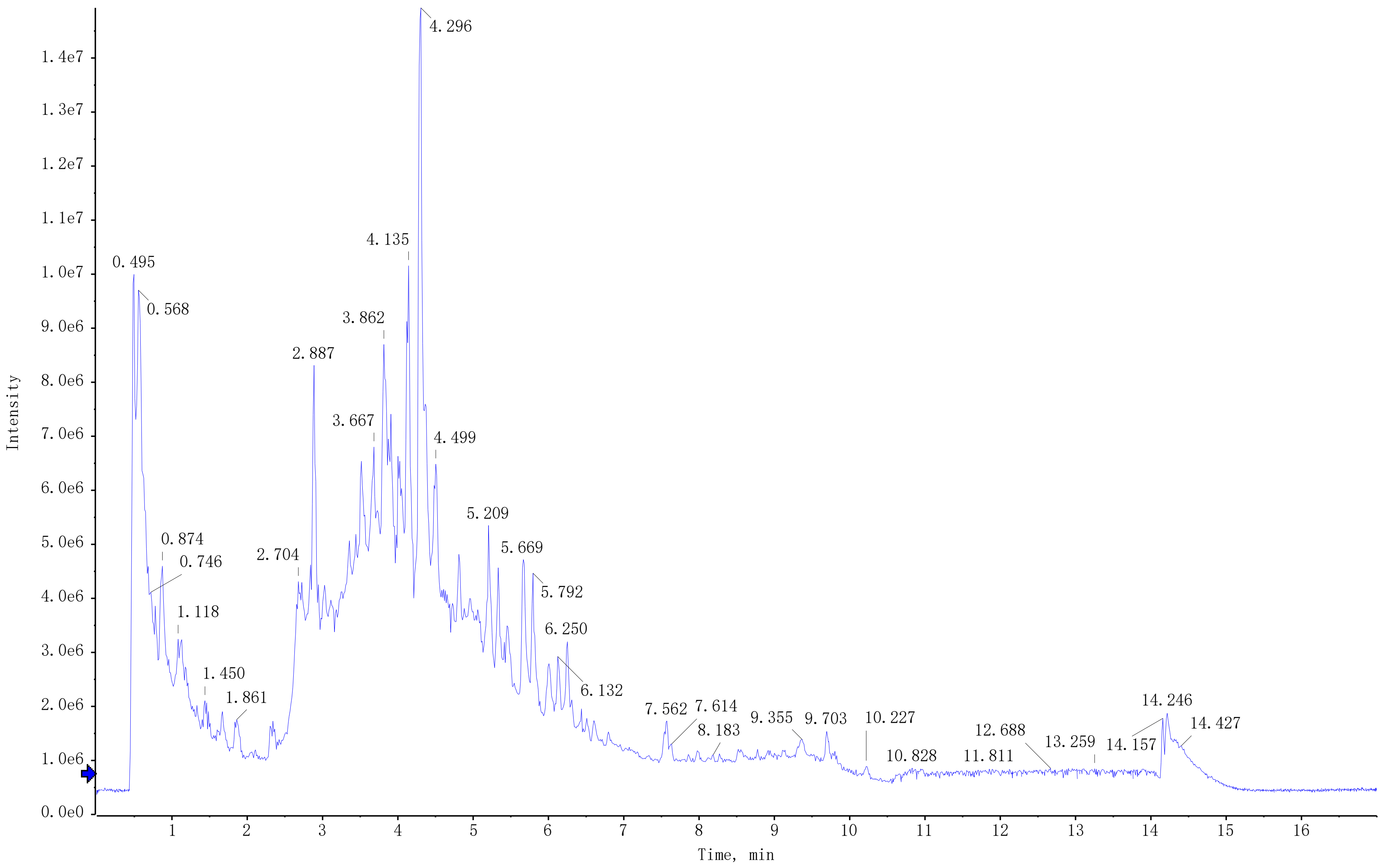

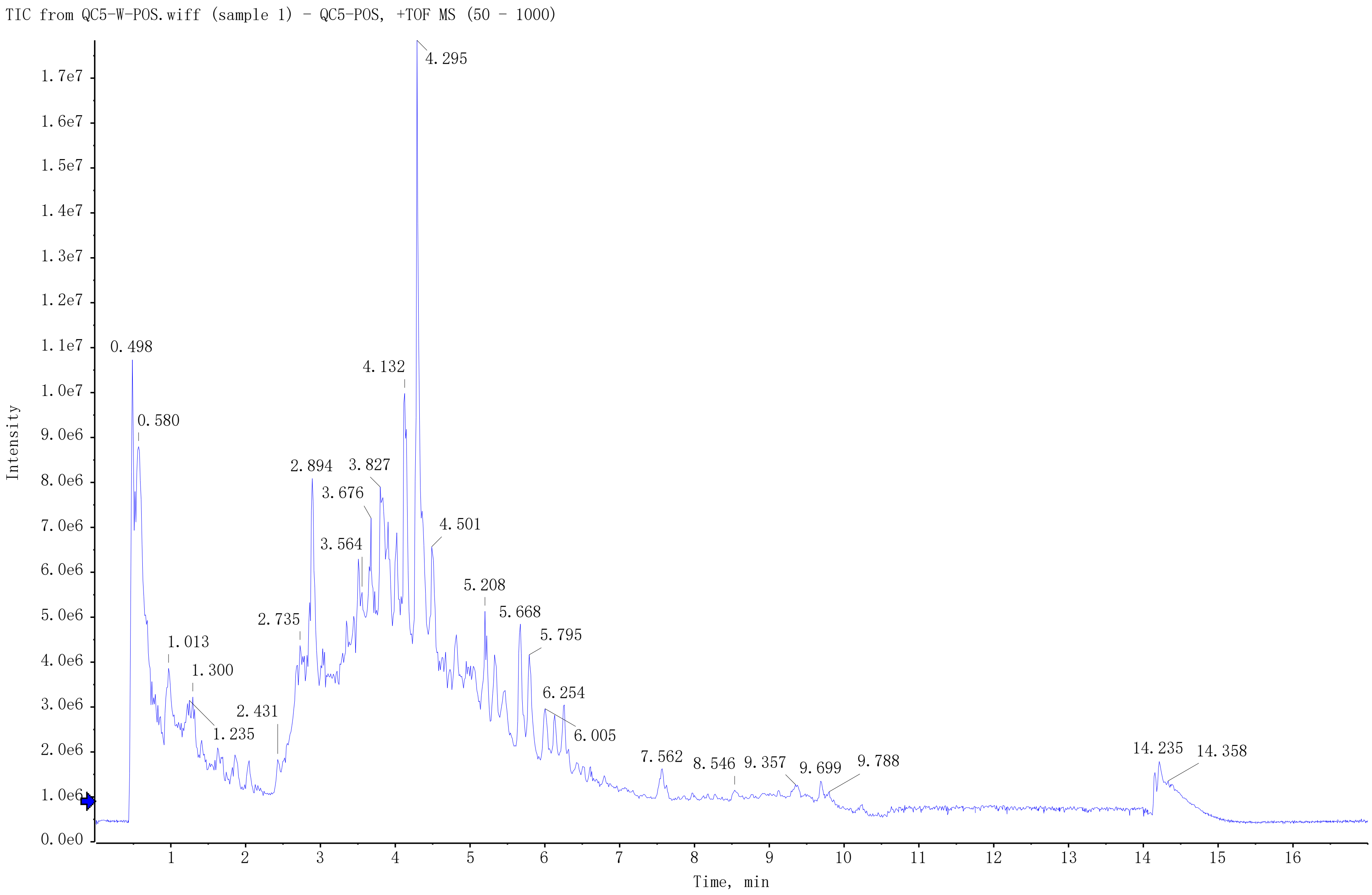

TIC from QC5-W-POS.wiff (sample 1) - QC5-POS, +TOF MS (50 - 1000)
Intensity
Time, min
4.295
0.498
4.132
0.580
2.894
3.827
3.676
4.501
3.564
5.208
5.668
5.795
2.735
1.013
1.300
6.254
2.431
1.235
6.005
7.562
8.546
9.357
9.699
9.788
14.235
14.358

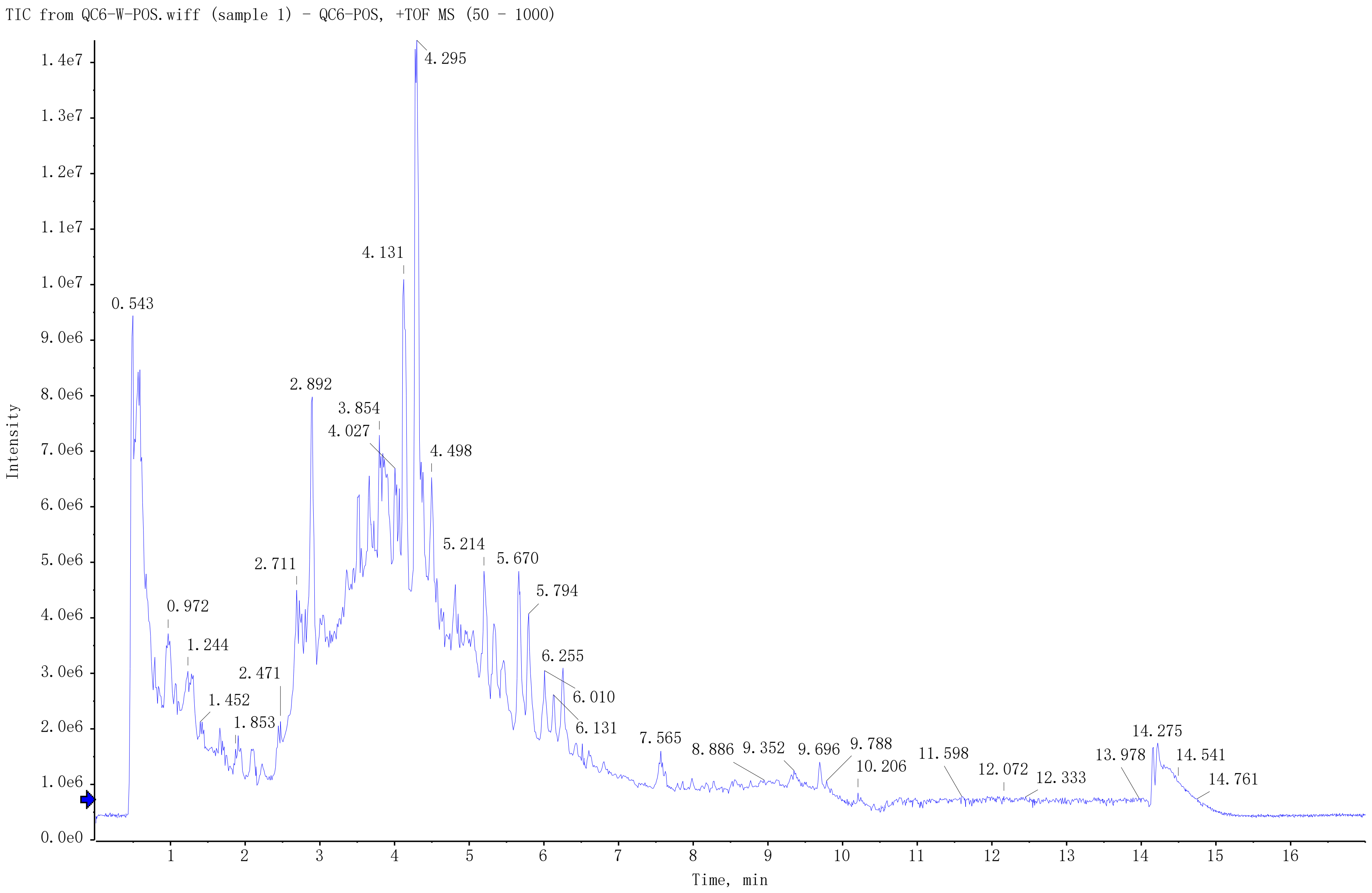
TIC from QC6-W-POS.wiff (sample 1) - QC6-POS, +TOF MS (50 - 1000)
Intensity
1.4e7
1.3e7
1.2e7
1.1e7
1.0e7
9.0e6
8.0e6
7.0e6
6.0e6
5.0e6
4.0e6
3.0e6
2.0e6
1.0e6
0.0e0
0.543
0.972
1.244
1.452
1.853
2.471
2.711
2.892
3.854
4.027
4.131
4.295
4.498
5.214
5.670
5.794
6.255
6.010
6.131
7.565
8.886
9.352
9.696
9.788
10.206
11.598
12.072
12.333
13.978
14.275
14.541
14.761
1
2
3
4
5
6
7
8
9
10
11
12
13
14
15
16
Time, min
