# Supplementary material for: Geographical variation in metabolite profiles and bioactivity of Thesium chinense Turcz. revealed by UPLC-Q-TOF-MS-based metabolomics
Source: Front Plant Sci. 2025 Jan 10;15:1471729. doi: 10.3389/fpls.2024.1471729 (PMC11760594; doi:10.3389/fpls.2024.1471729)
Supplement: Supplementary file 3 [file SupplementaryFile4.doc]

Supplementary File 4: The structures of the differential metabolites and their secondary mass spectrometry information.

| No. | Compound Name | Formula | Adducts | m/z | ion modes |
| --- | --- | --- | --- | --- | --- |
| 1 | Oxysophocarpine | C_15_H_22_N_2_O_2_ | [M+H]+ | 263.1751 | 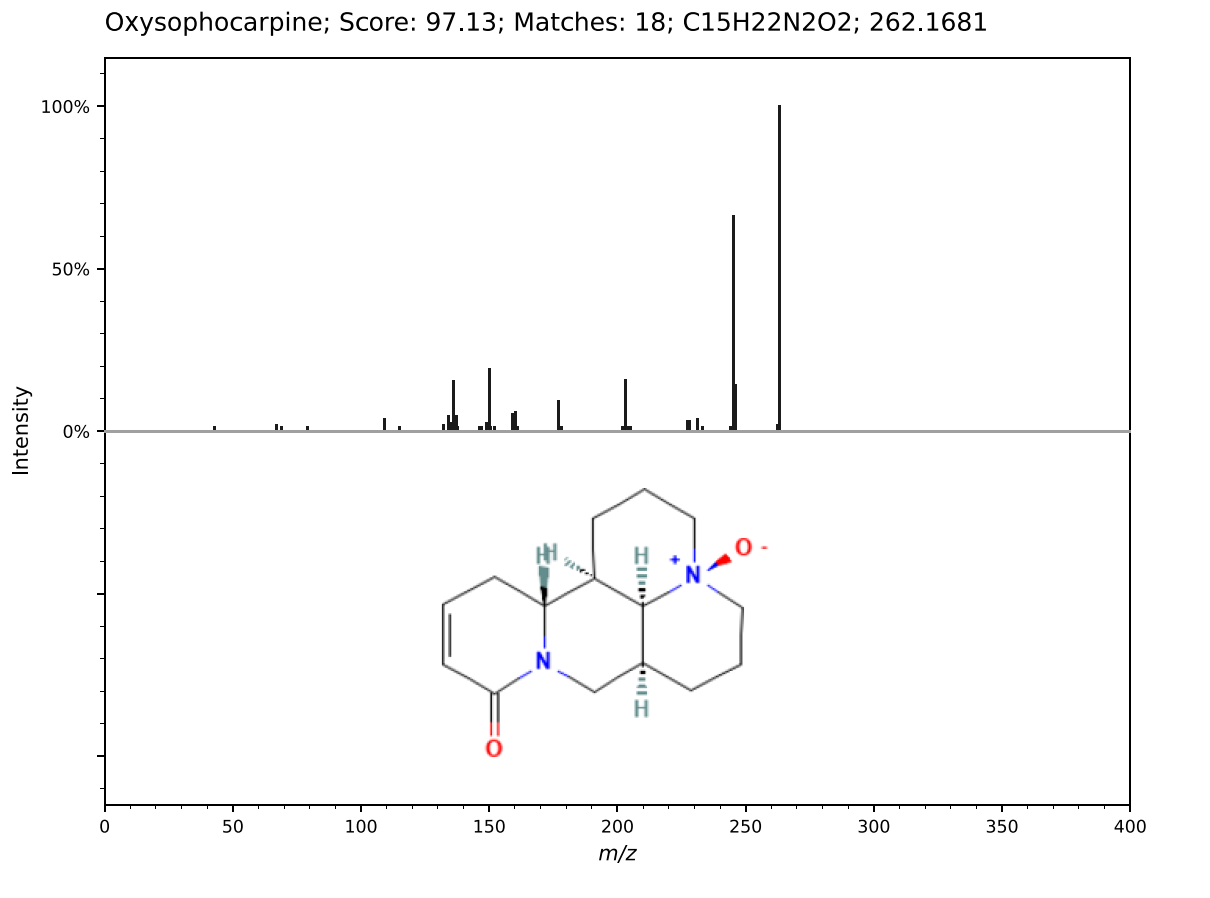 |
| 2 | 2,2-Difluoro-1,3-benzodioxole-4-carboxylic acid | C8H4F2O4 | [M+H]+ | 203.0152 | 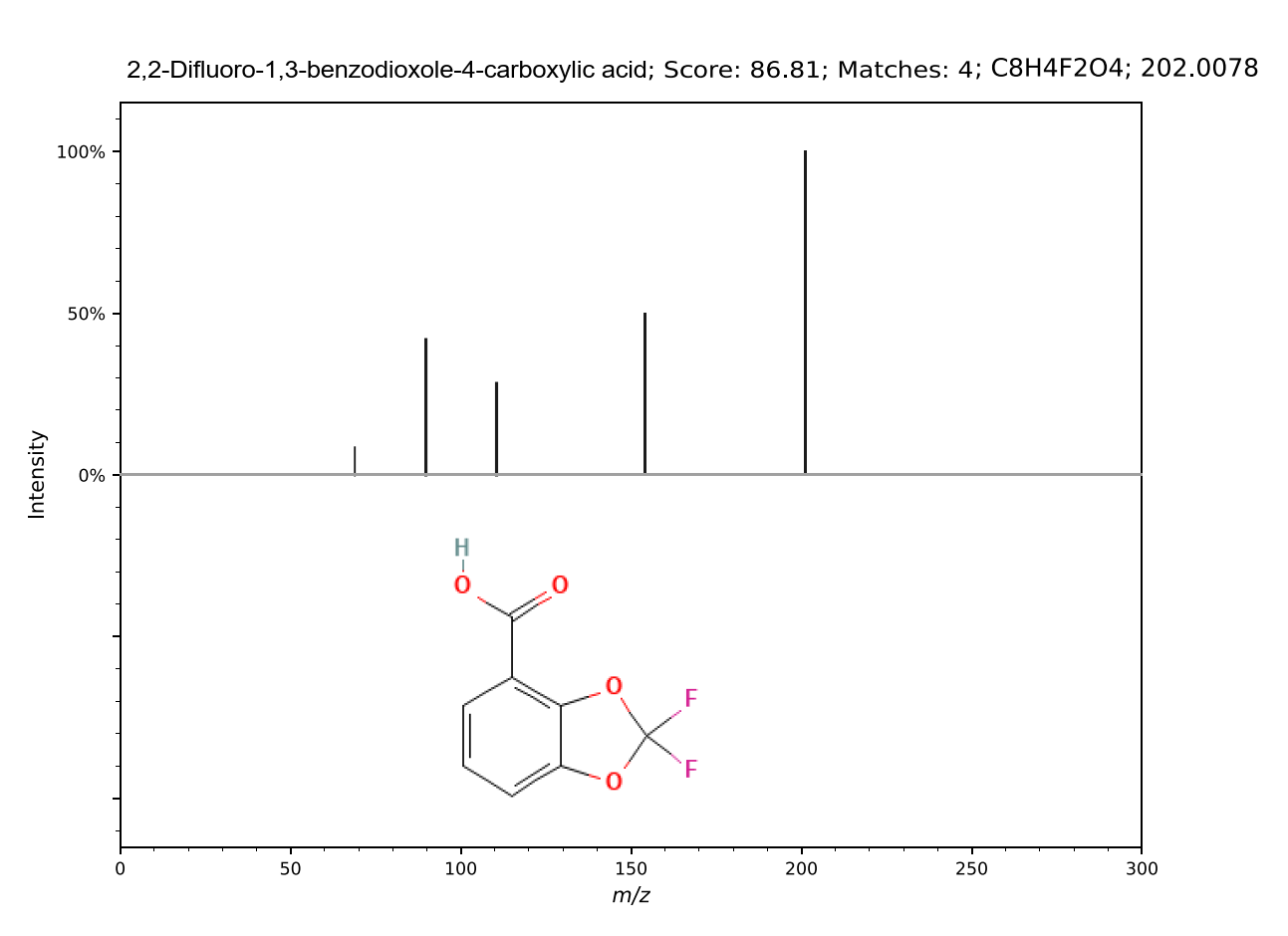 |
| 3 | Neodiosmin | C_28_H_32_O_15_ | [M+H]+ | 609.1814 | 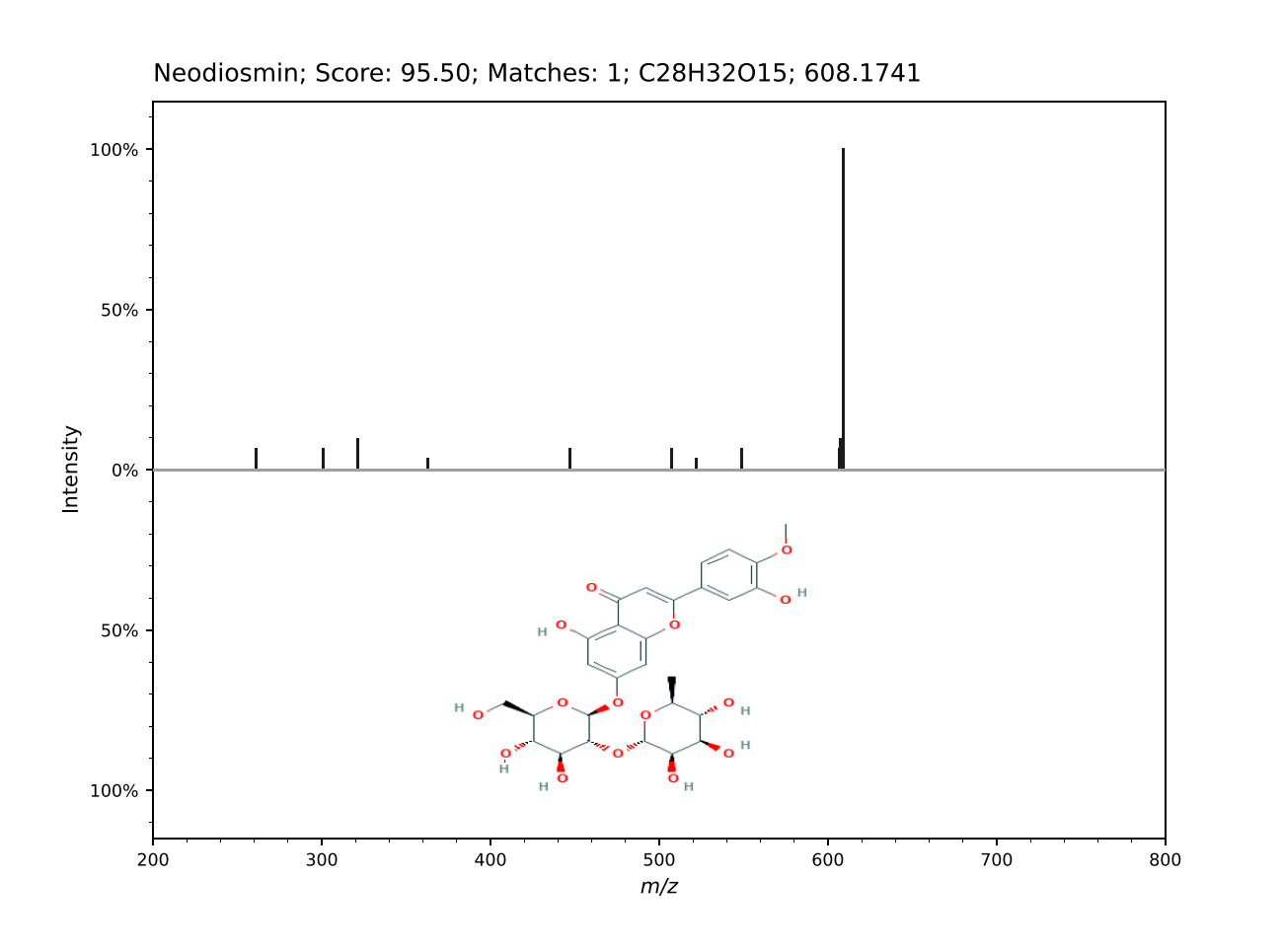 |
| 4 | Baohuoside I | C_27_H_30_O_10_ | [M+Na]+ | 537.1727 | 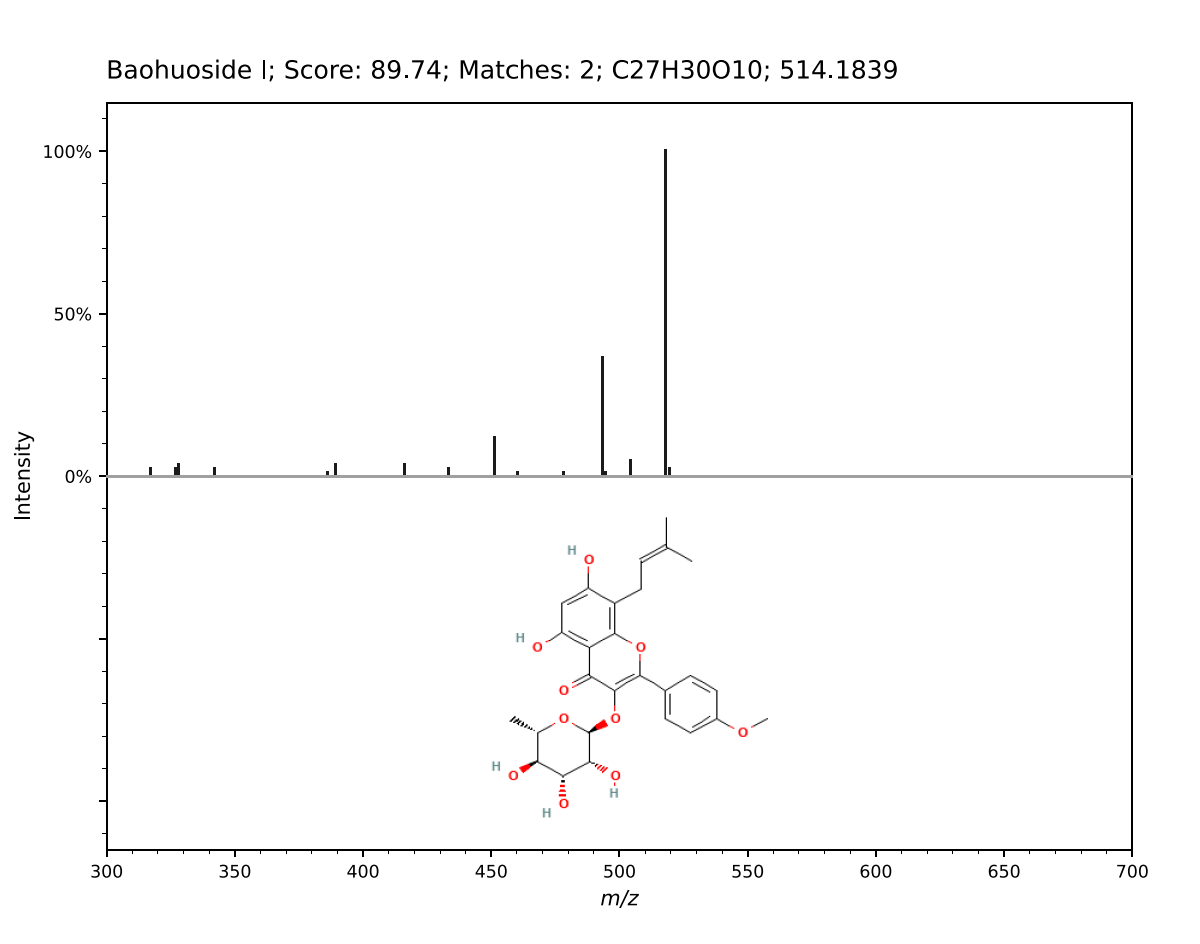 |
| 5 | kaempferol | C_15_H_10_O_6_ | [M+H]+ | 287.0549 | 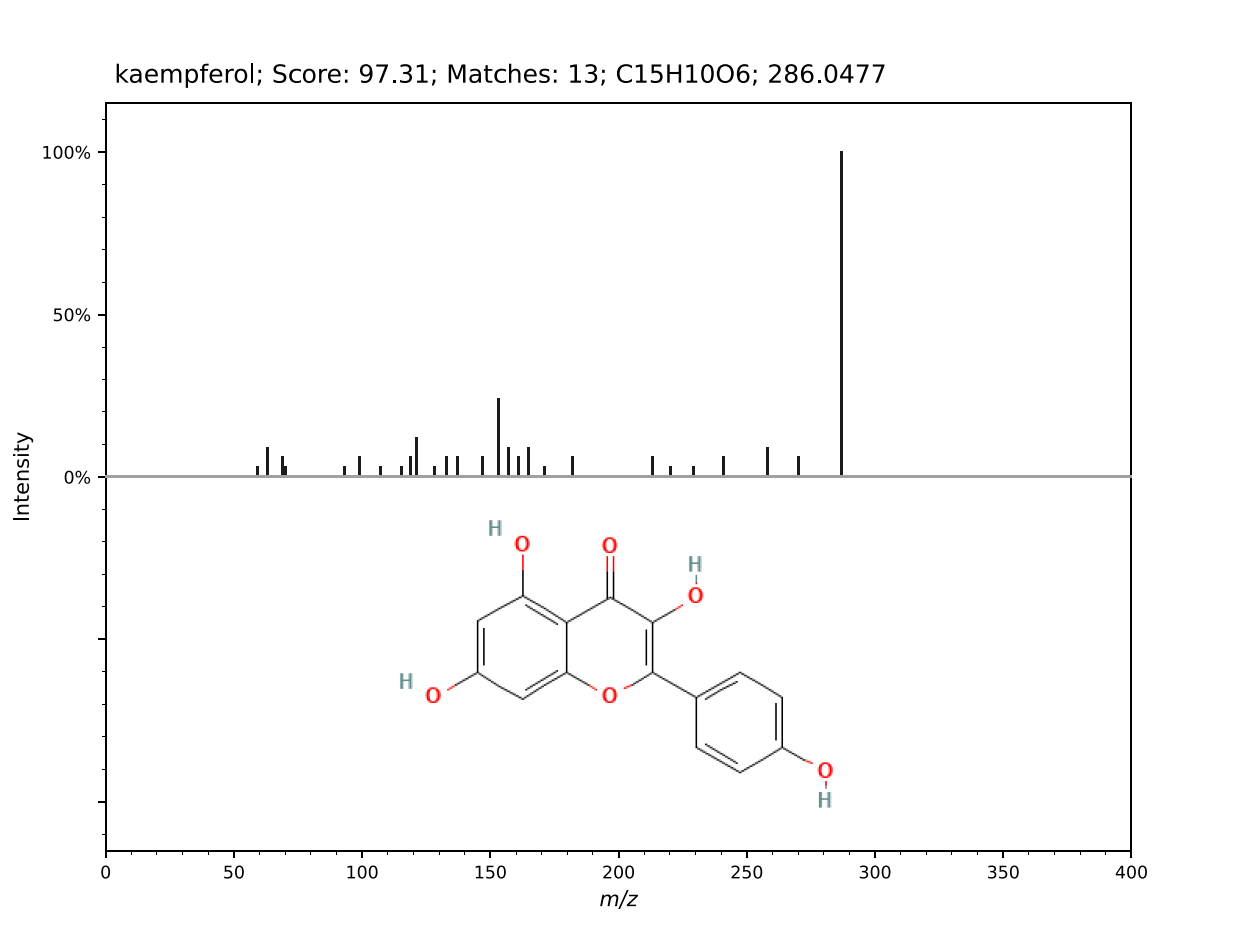 |
| 6 | Sanggenone H | C_20_H_18_O_6_ | [M+CH3OH+H]+ | 387.1444 | 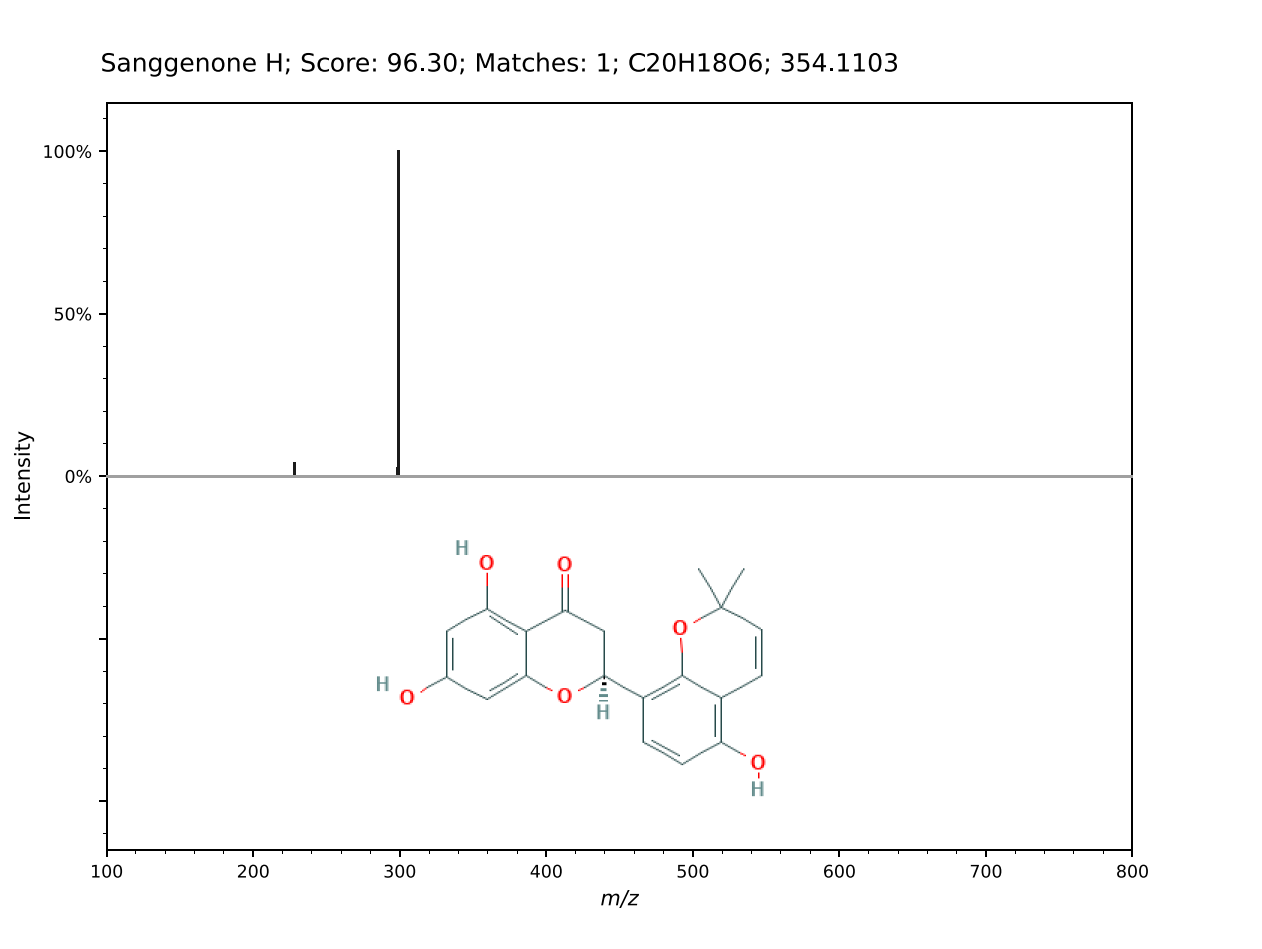 |
| 7 | 3,4,5-Trihydroxy-1-cyclohexene-1-carboxylic acid | C_7_H_10_O_5_ | [M+H]+ | 175.0610 | 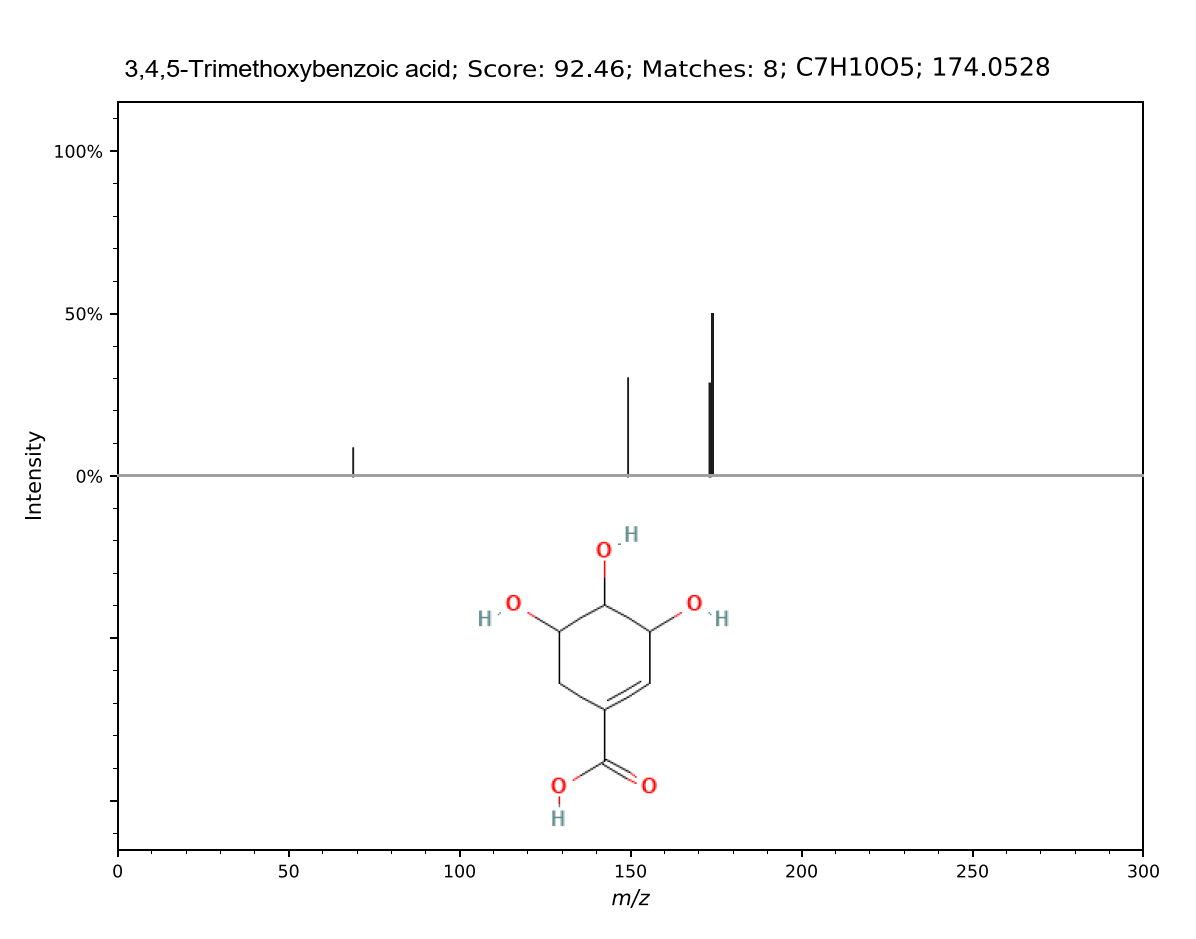 |
| 8 | Myristicin | C_11_H_12_O_3_ | [M+H]+ | 193.0852 | 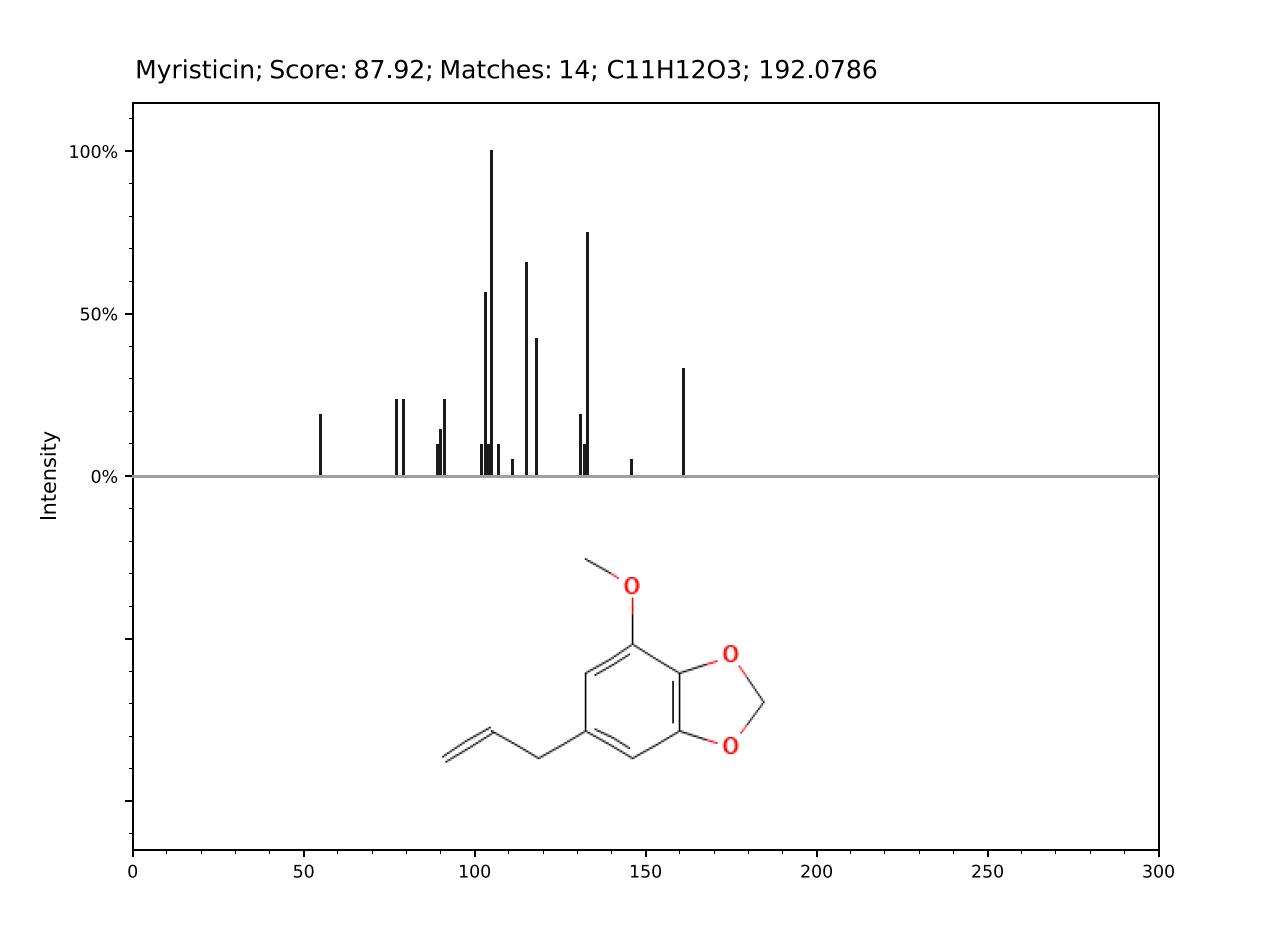 |
| 9 | 13-alpha-(21)-Epoxyeurycomanone | C_20_H_24_O_10_ | [M+H]+ | 425.1452 | 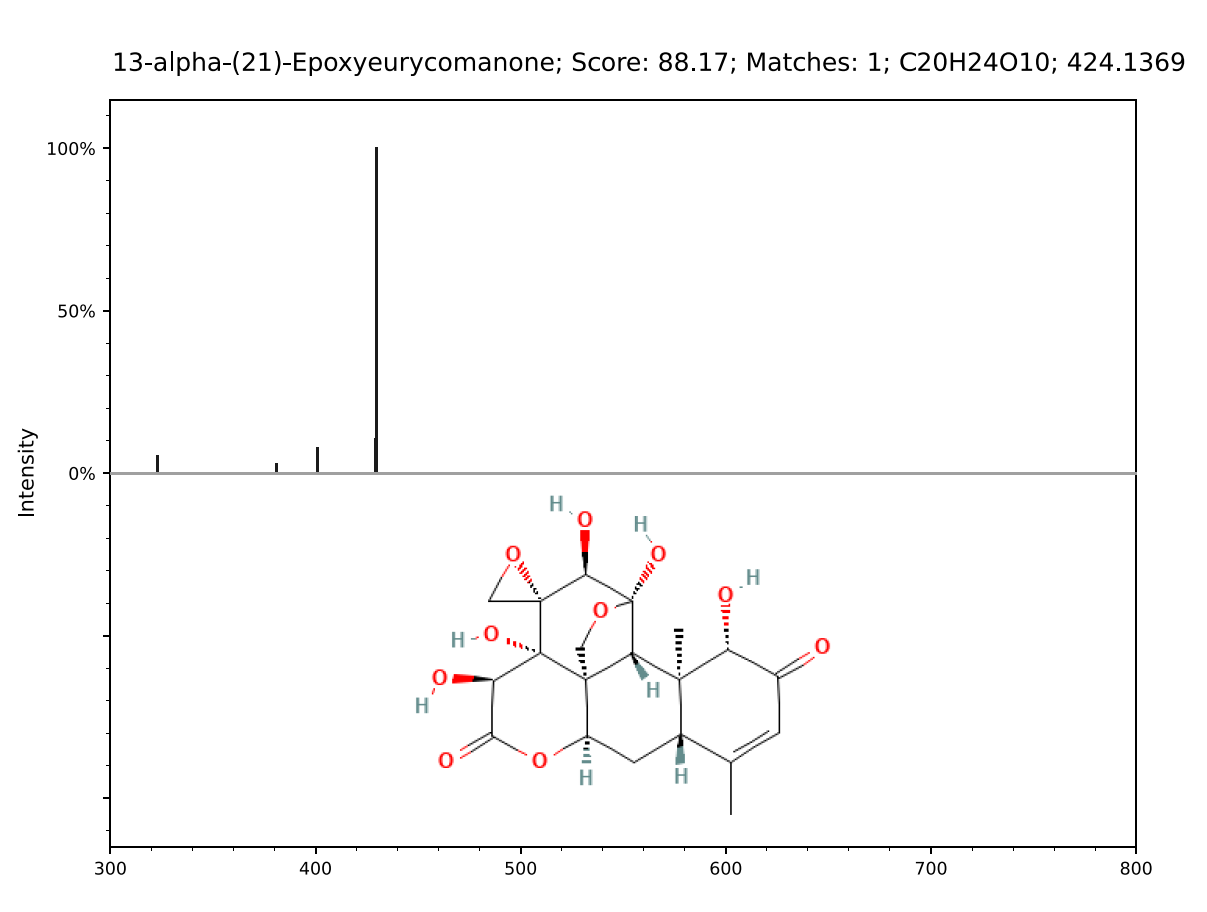 |
| 10 | Sophoraflavonoloside | C_27_H_30_O_16_ | [M+H]+ | 611.1622 | 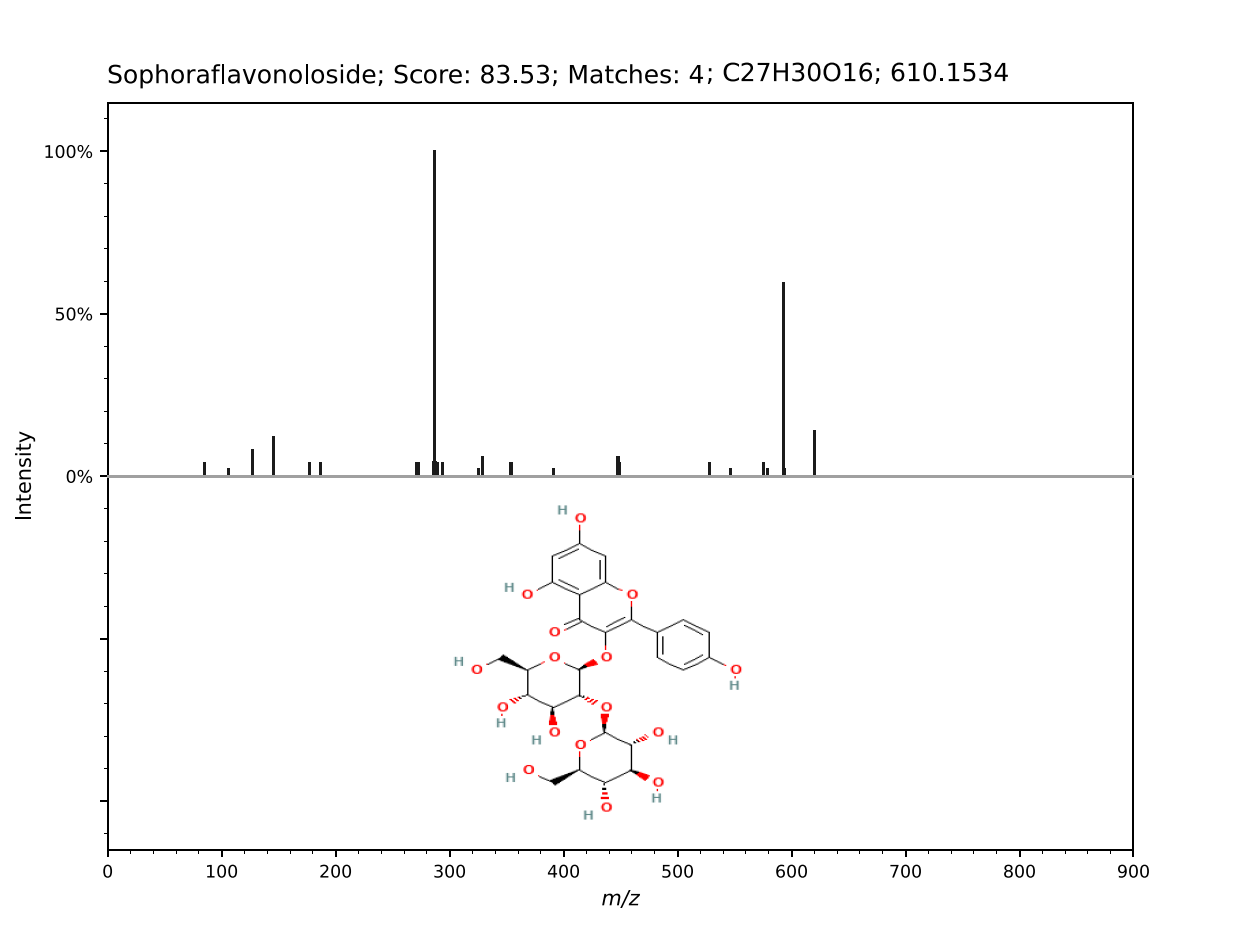 |
| 11 | Loliolide | C_11_H_16_O_3_ | [M+H]+ | 197.1174 | 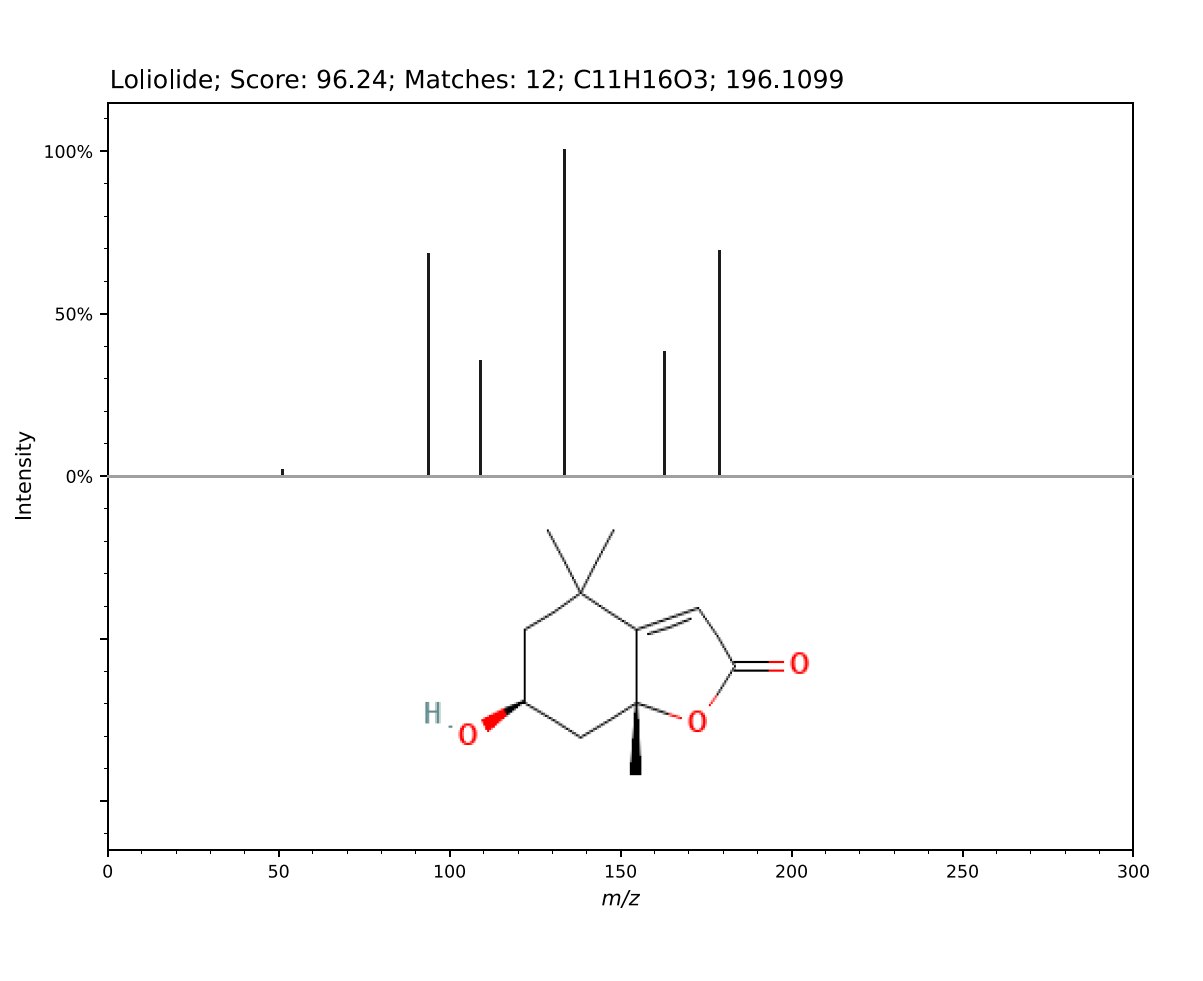 |
| 12 | Ferulic acid | C_10_H_10_O_4_ | [M+H]+ | 195.0657 | 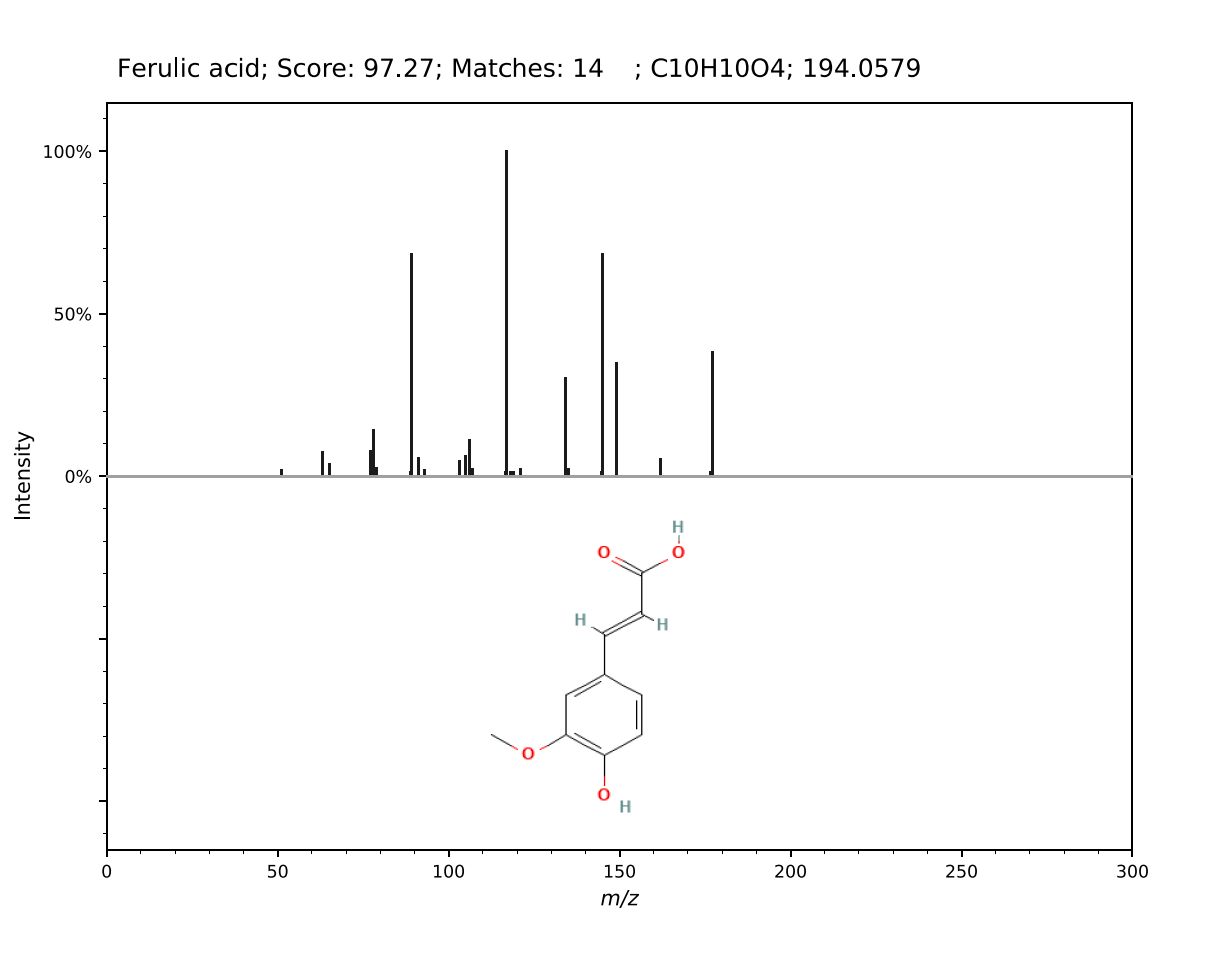 |
| 13 | Kaempferol 3-O-vicianoside | C_26_H_28_O_15_ | [M+H]+ | 581.1504 | 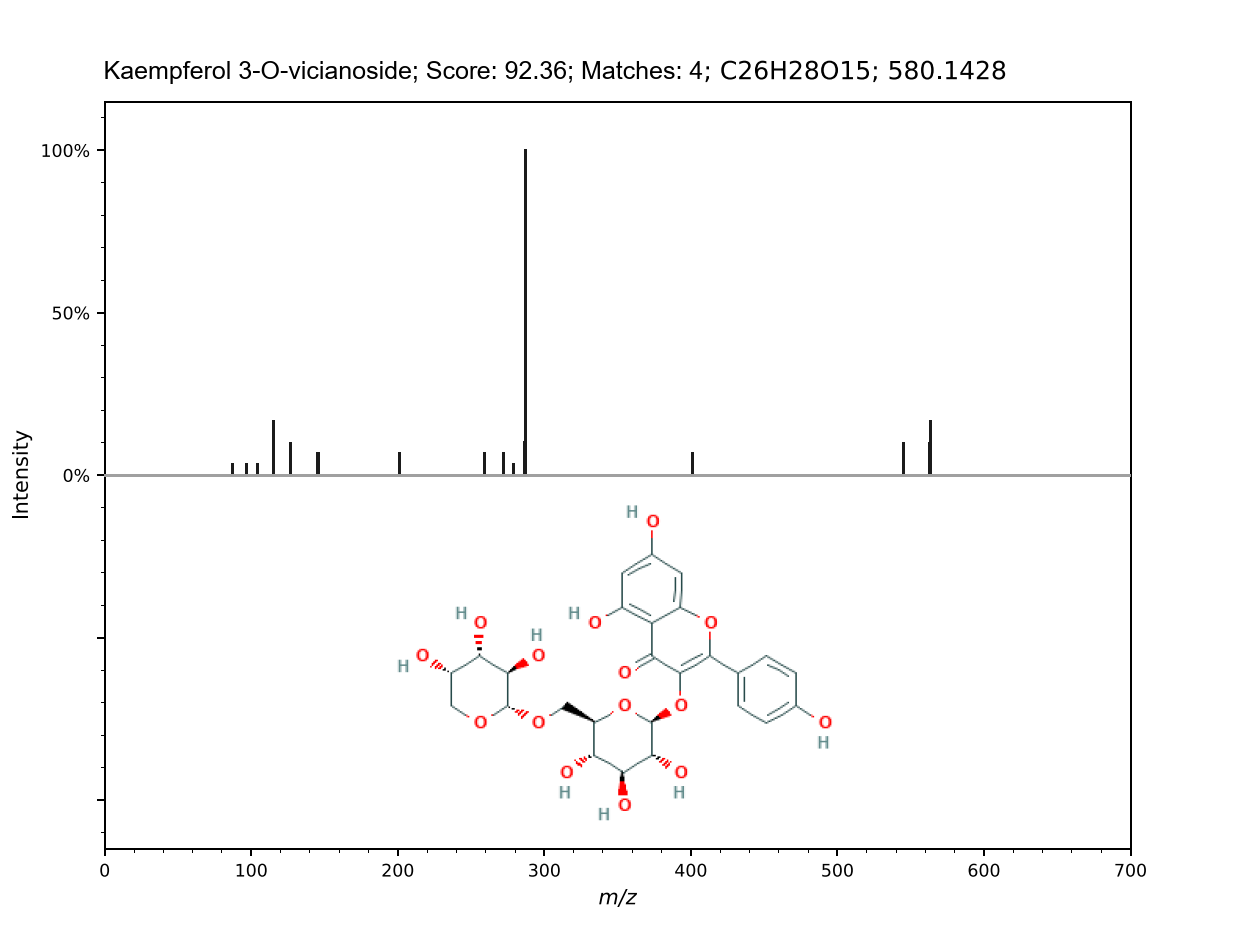 |
| 14 | Piceid | C_20_H_22_O_8_ | [M+H]+ | 391.1394 | 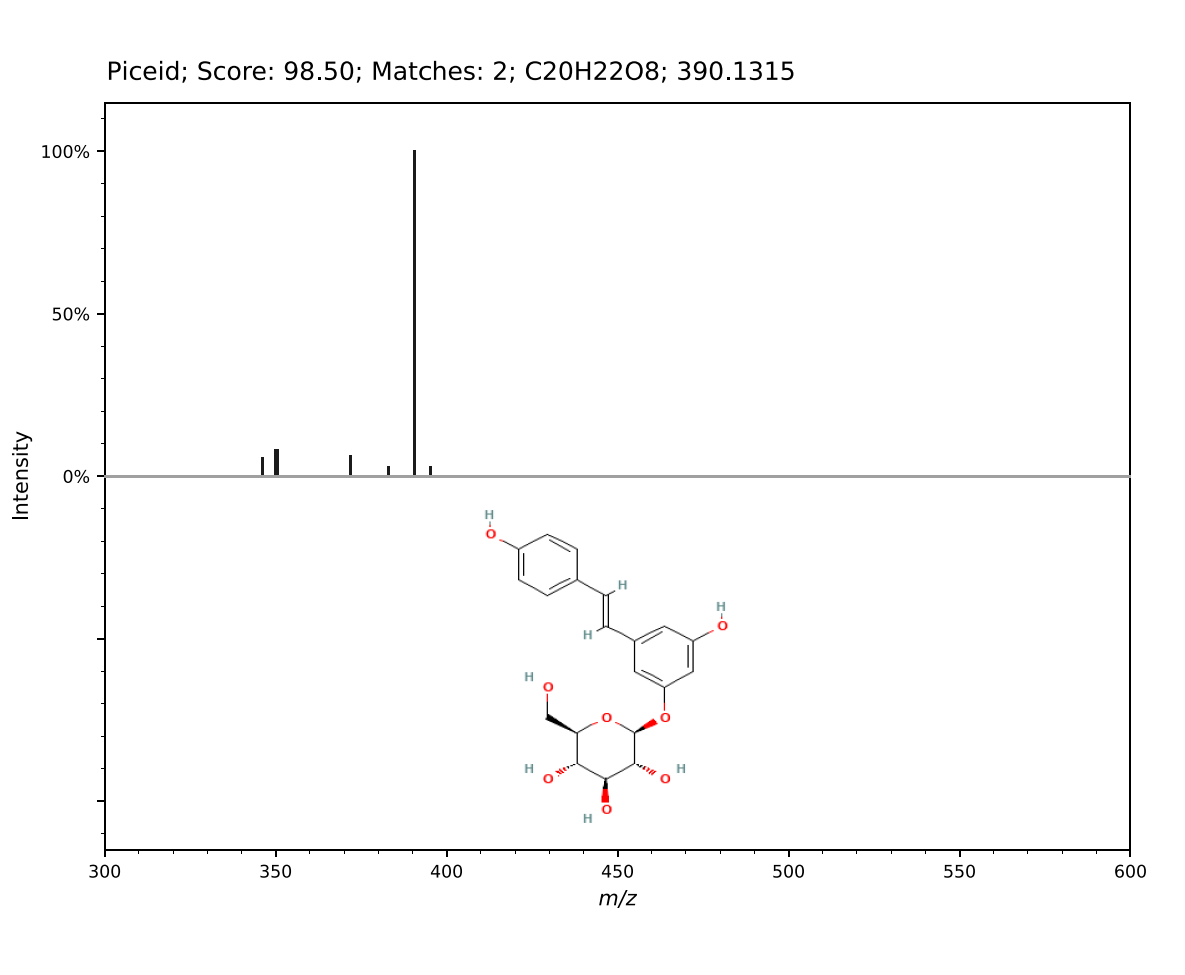 |
| 15 | Lespedin | C_27_H_30_O_14_ | [M+H]+ | 579.1722 | 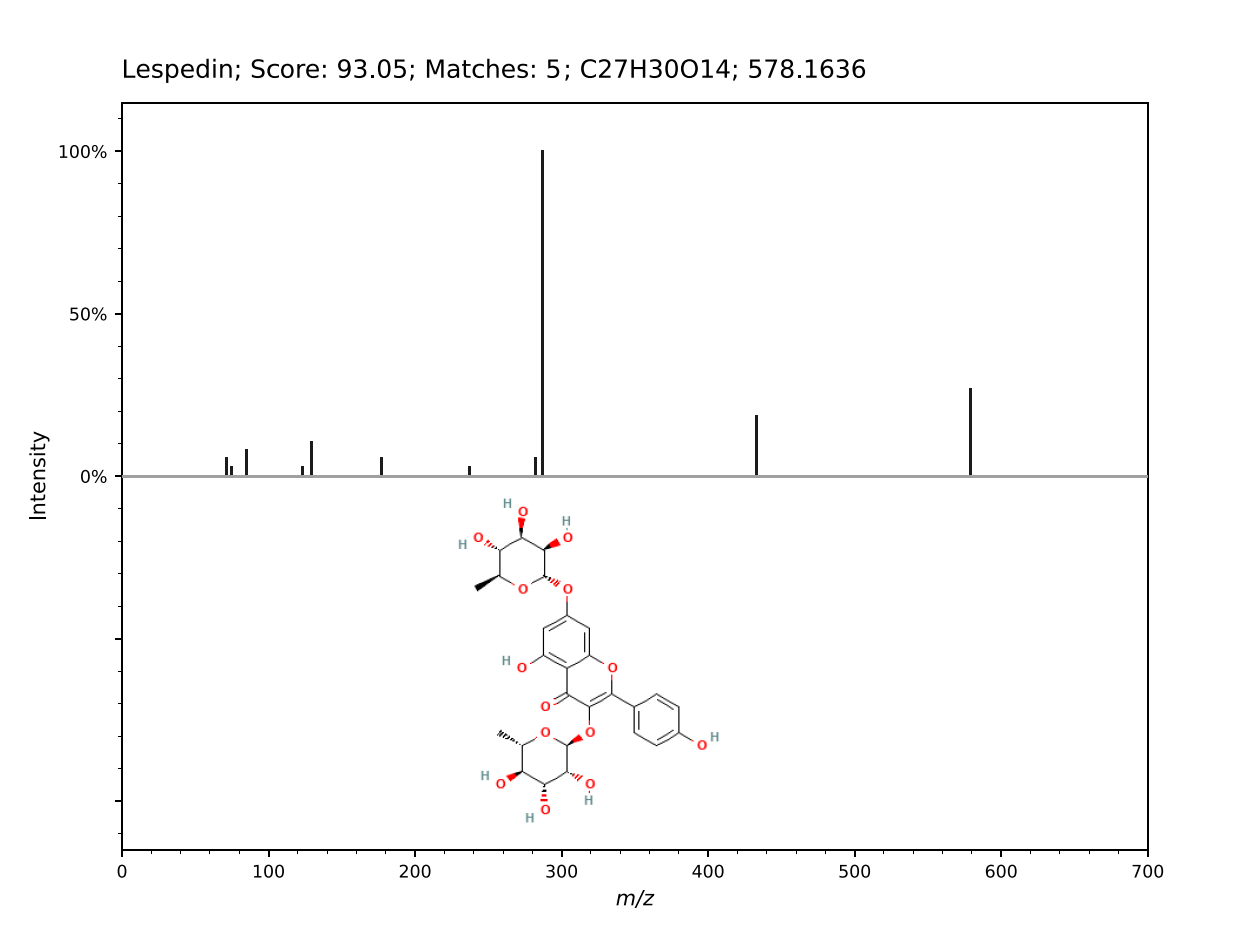 |
| 16 | Vincanidine | C_19_H_20_N_2_O_2_ | [M+H]+ | 309.1589 | 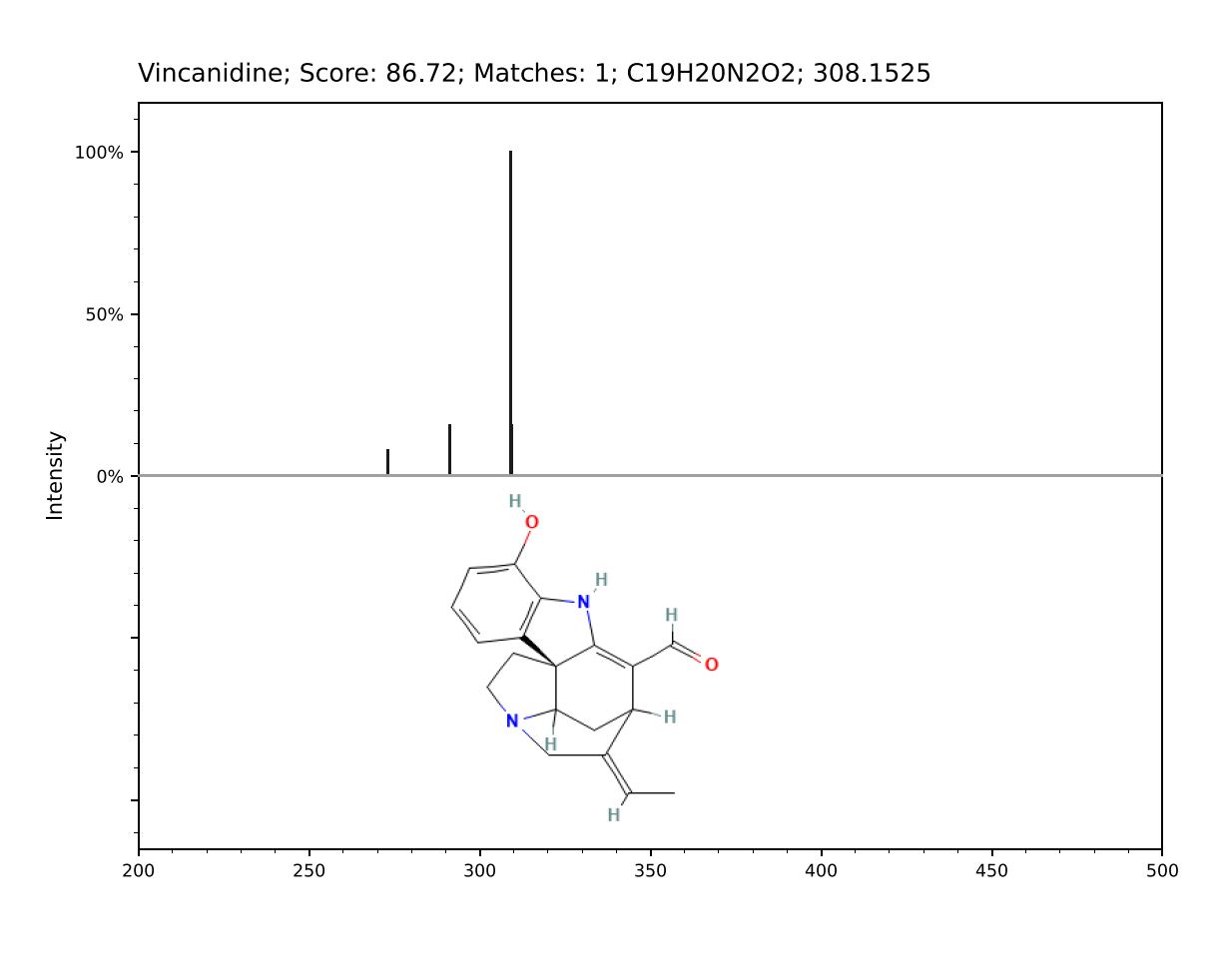 |
| 17 | 3,4,5-Trimethoxybenzoic acid | C_10_H_12_O_5_ | [M+H]+ | 213.0759 | 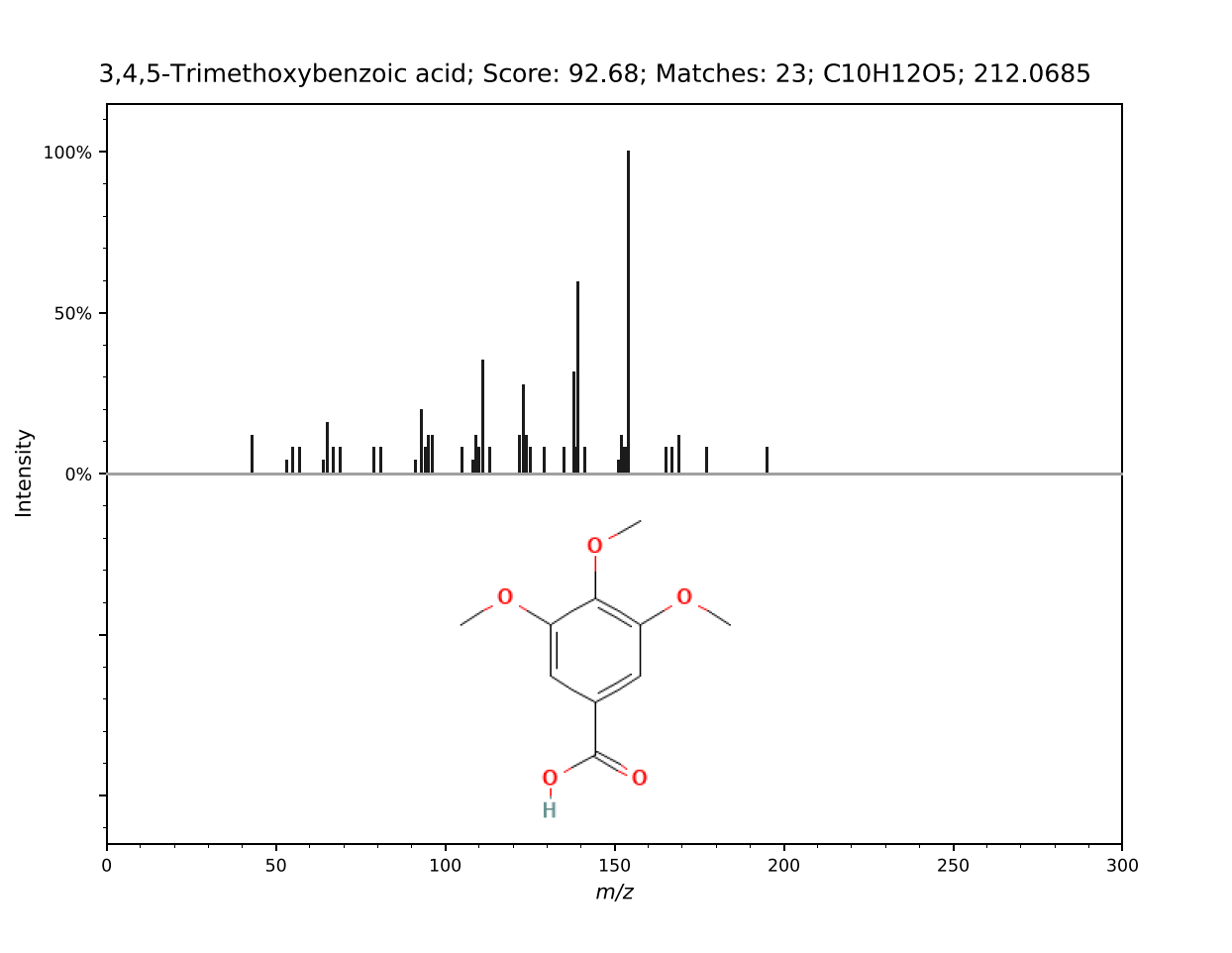 |
| 18 | 16-Hydroxyhexadecanoic acid | C_16_H_32_O_3_ | [M+H]+ | 273.2429 | 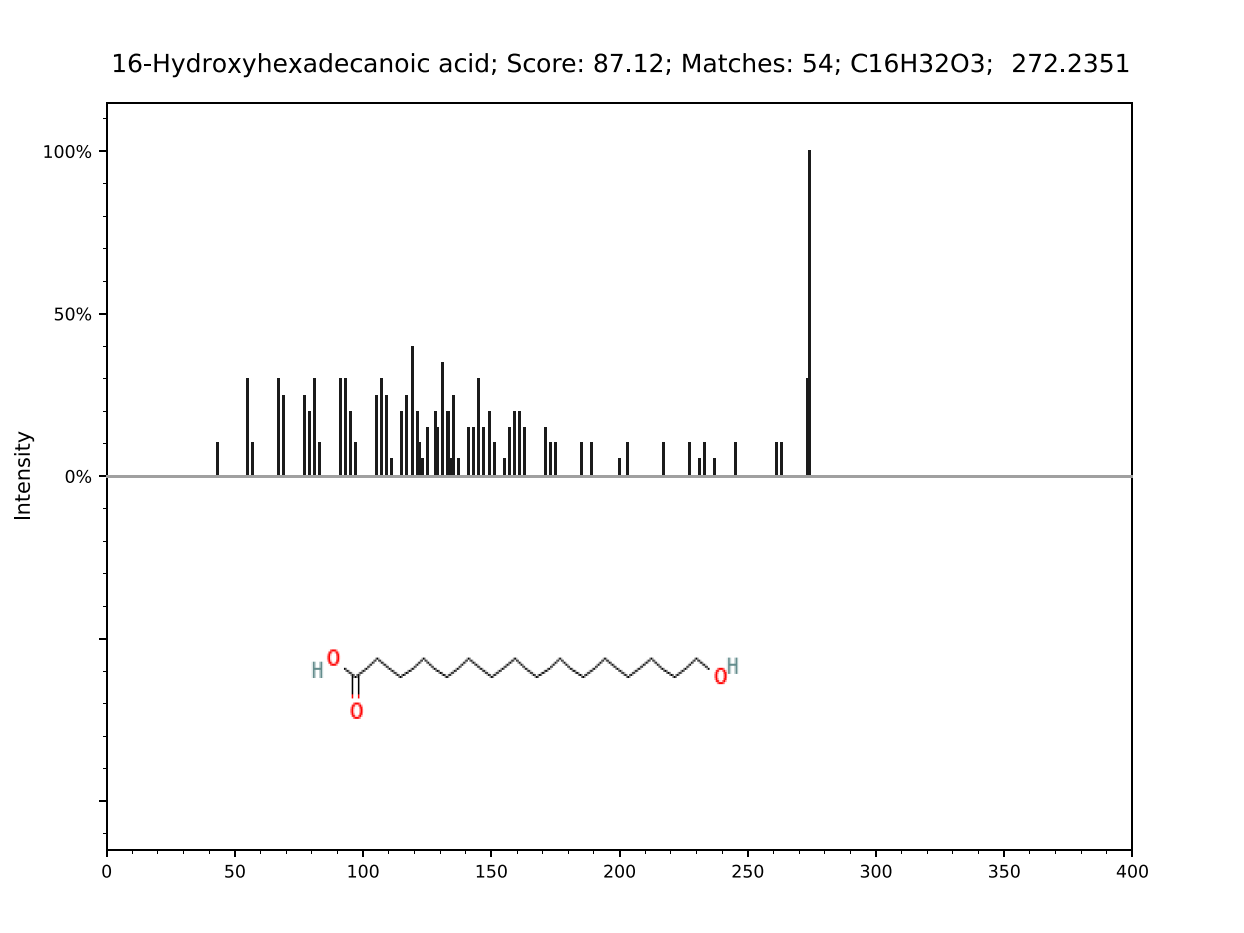 |
| 19 | Hexadecanedioic acid | C_16_H_30_O_4_ | [M+H]+ | 287.2224 | 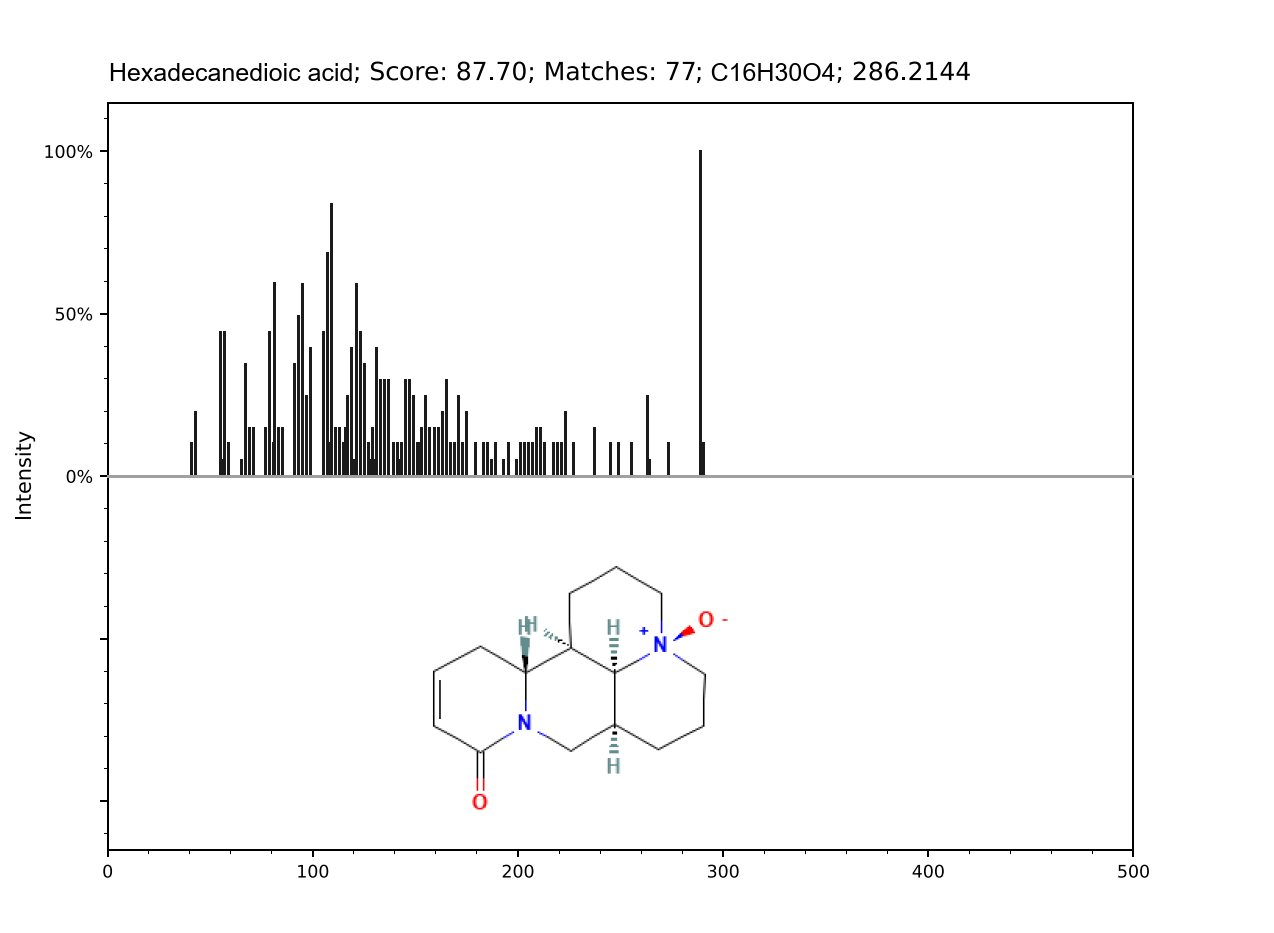 |
| 20 | 4-(Tridecanoylamino)benzoic acid | C_20_H_31_NO_3_ | [M+H]+ | 334.2383 | 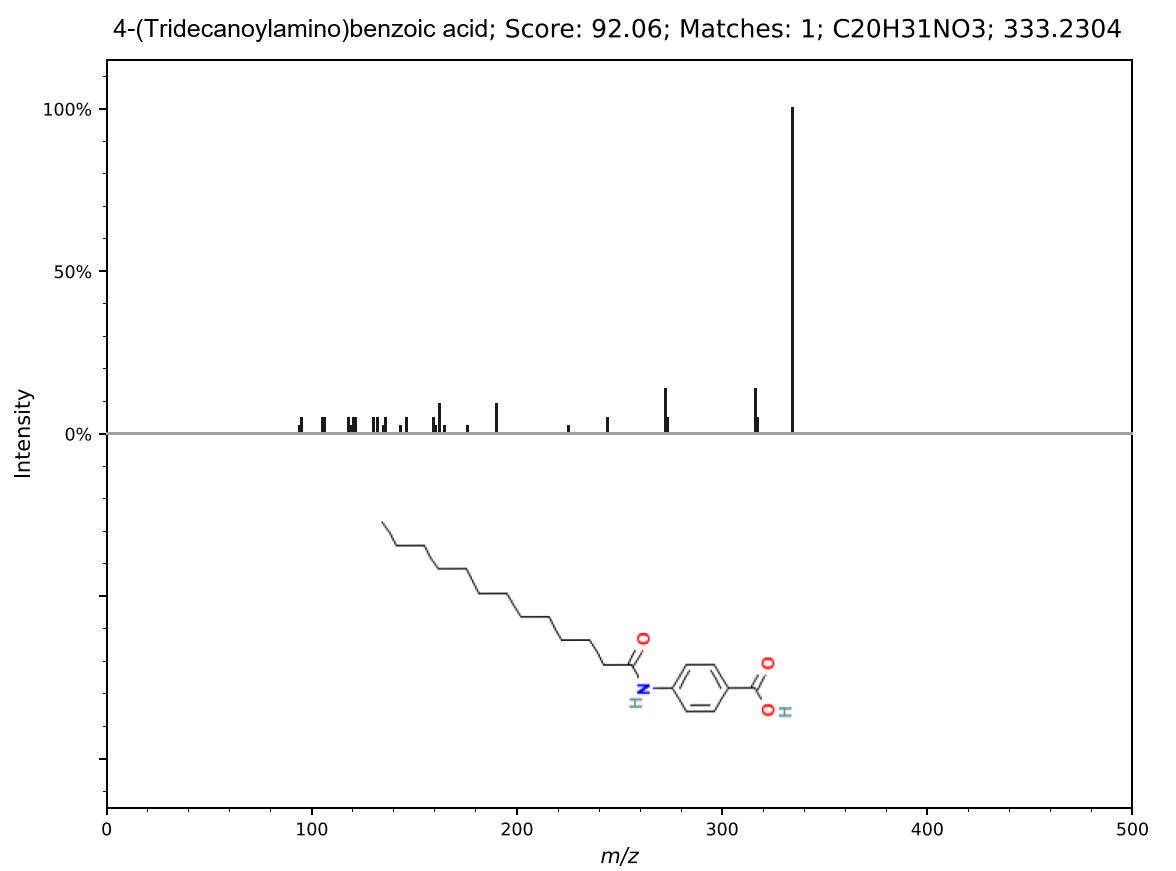 |
| 21 | Henicosanoic acid | C_21_H_42_O_2_ | [M+H]+ | 327.3243 | 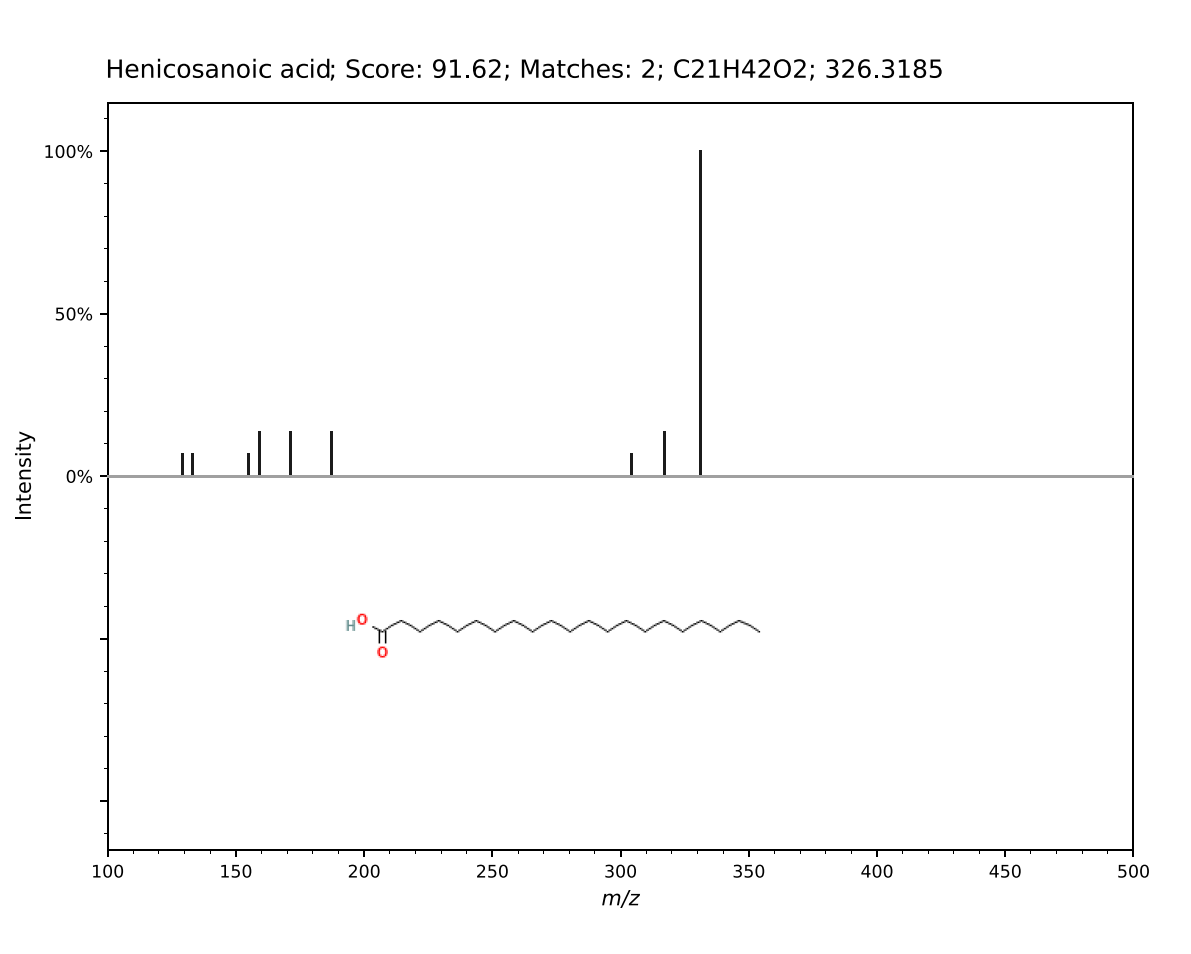 |
| 22 | 2-Amino-1-phenylethanol | C_8_H_11_NO | [M+NH4]+ | 155.1173 | 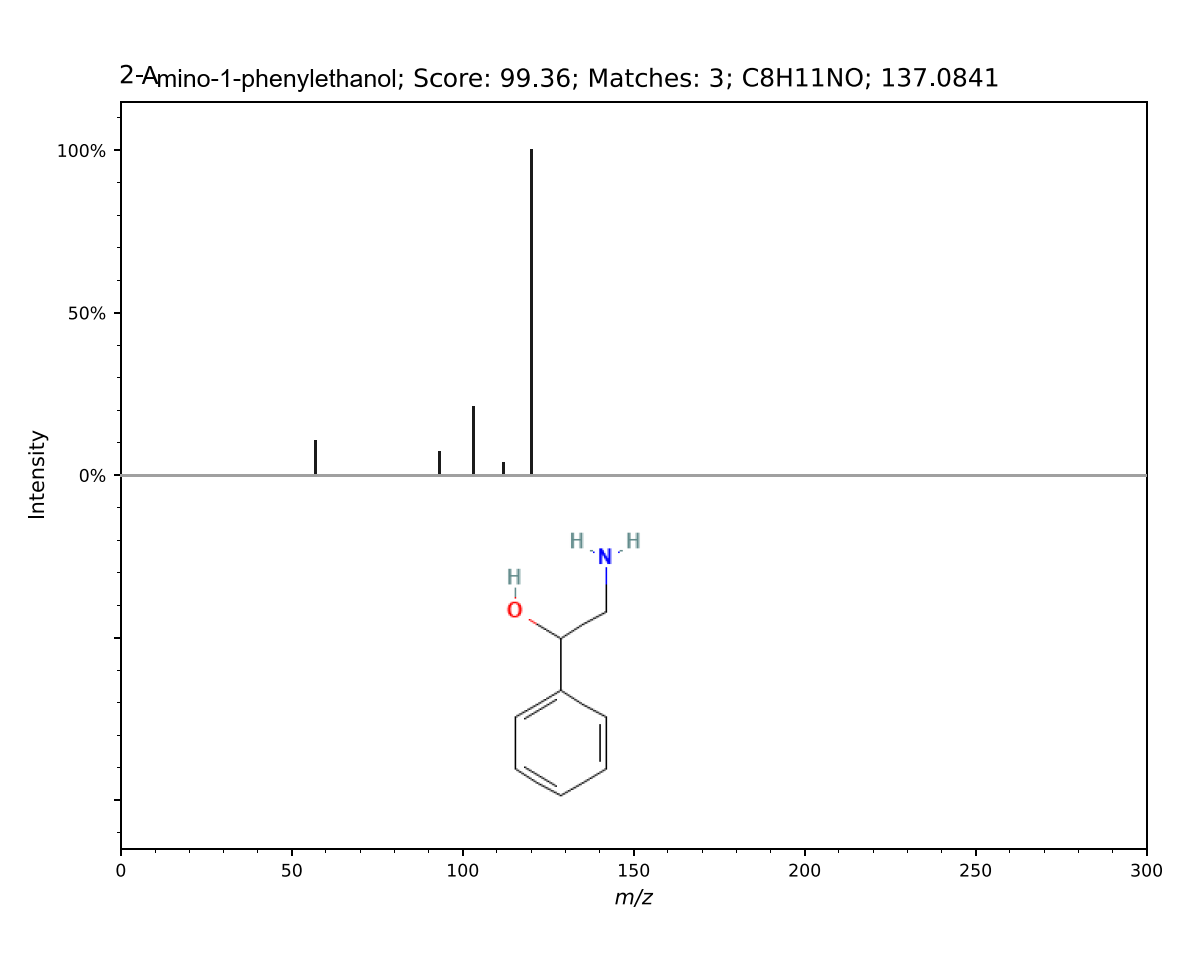 |
| 23 | Valtrate | C_22_H_30_O_8_ | [M+2H]2+ | 212.1050 | 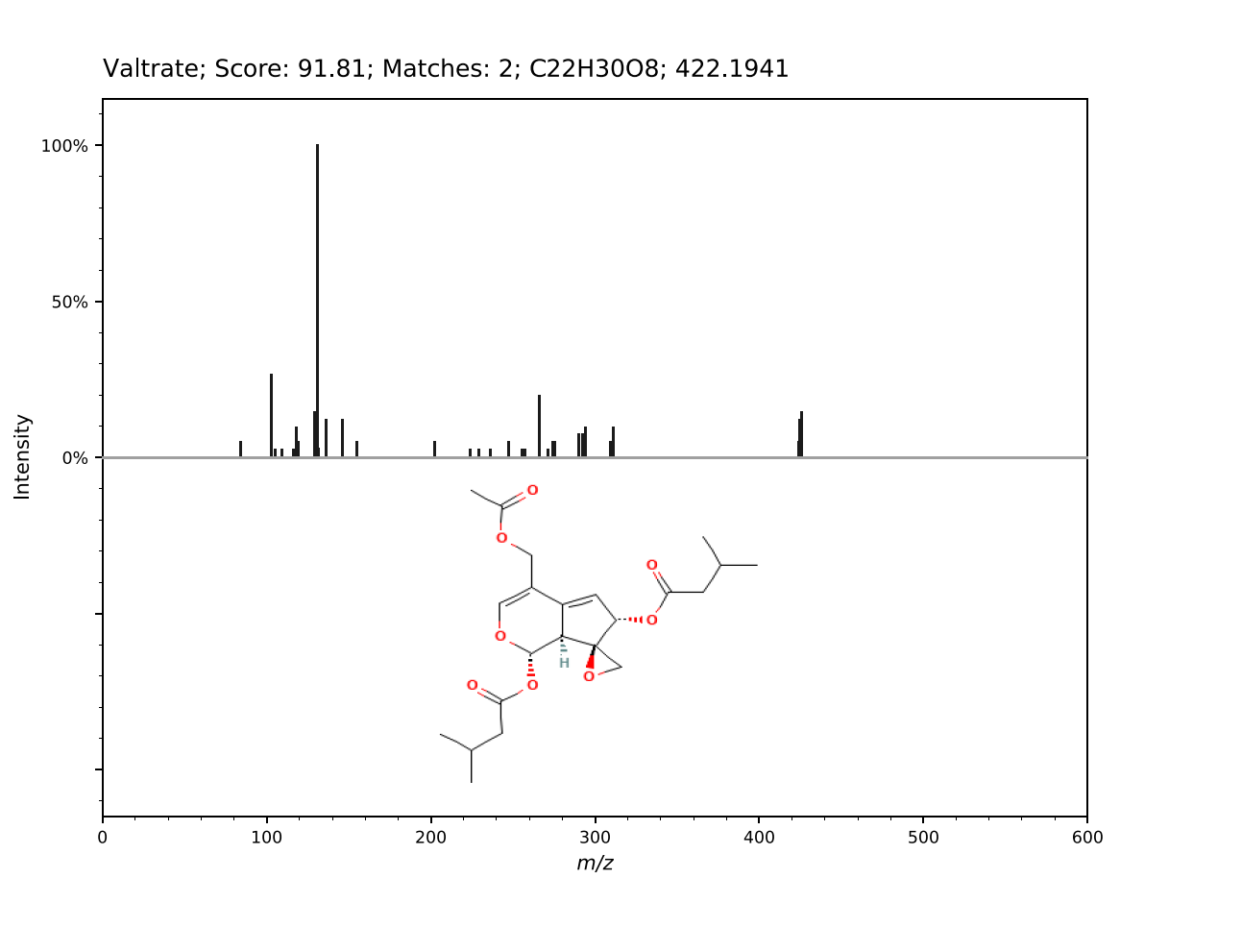 |
| 24 | 7-Hydroxy-3-(2-hydroxy-propyl)-5-methyl-isochromen-1-one | C_13_H_14_O_4_ | [M+H]+ | 235.0970 | 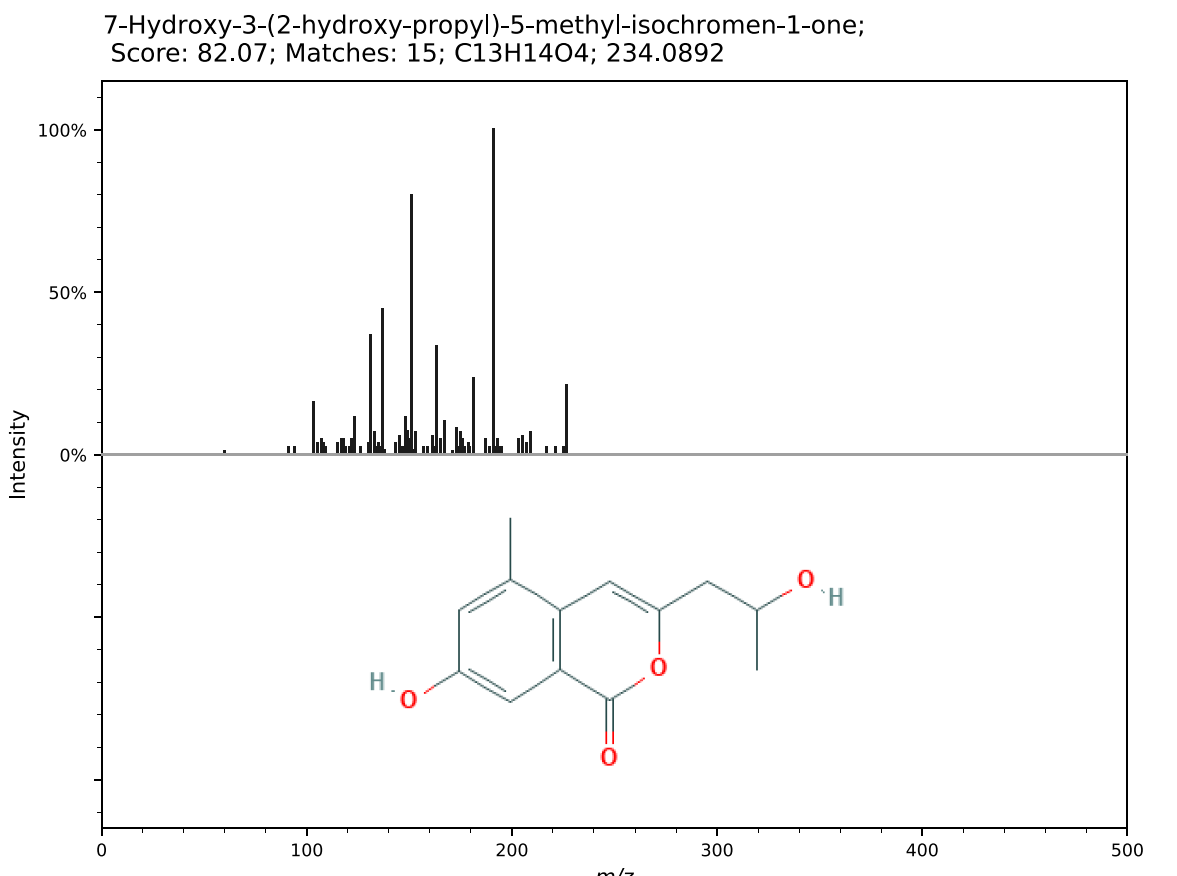 |
| 25 | Phytol | C_20_H_40_O | [M+H-H2O]+ | 279.3048 | 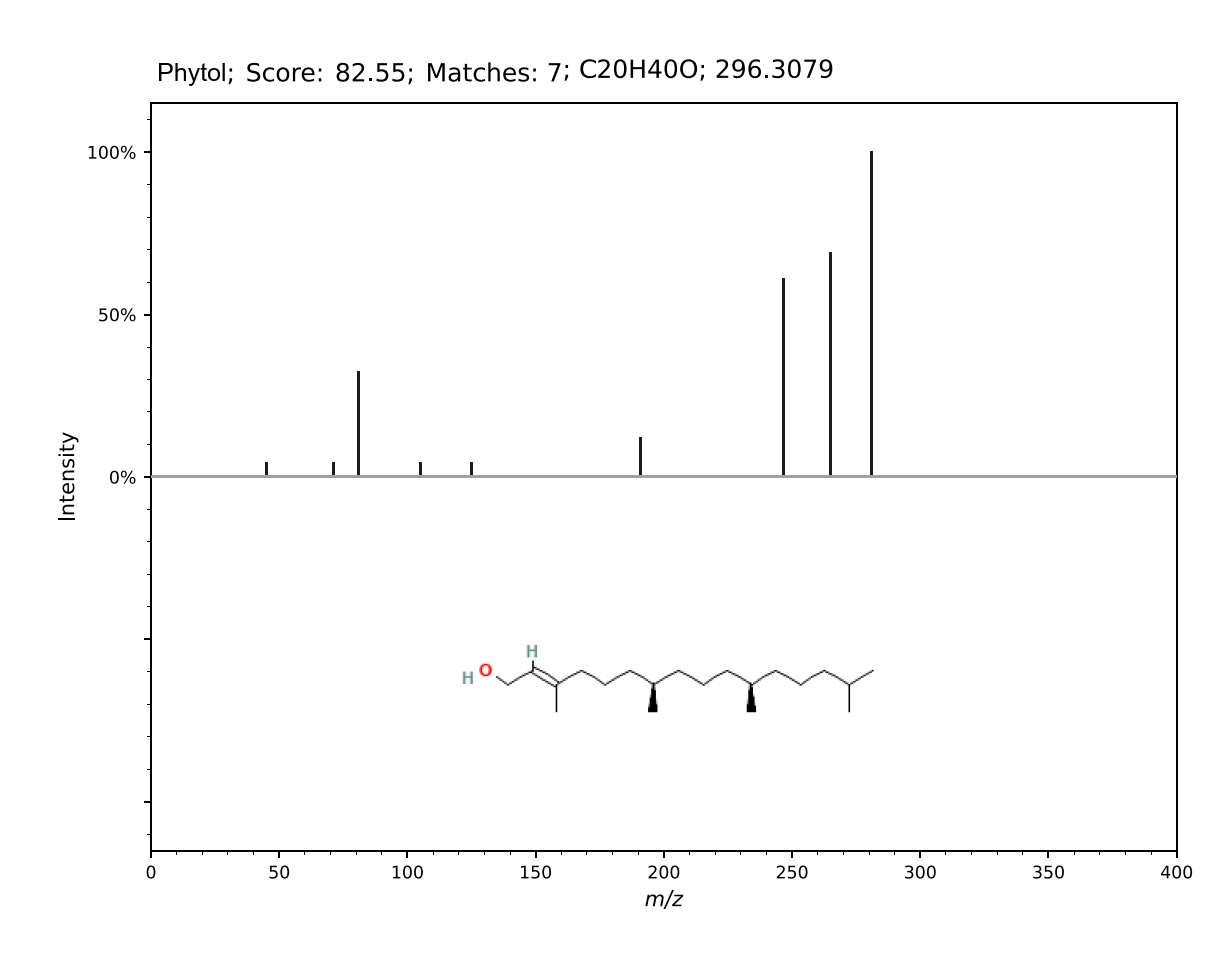 |
| 26 | 15-Kete | C_20_H_30_O_3_ | [M+H]+ | 319.2273 | 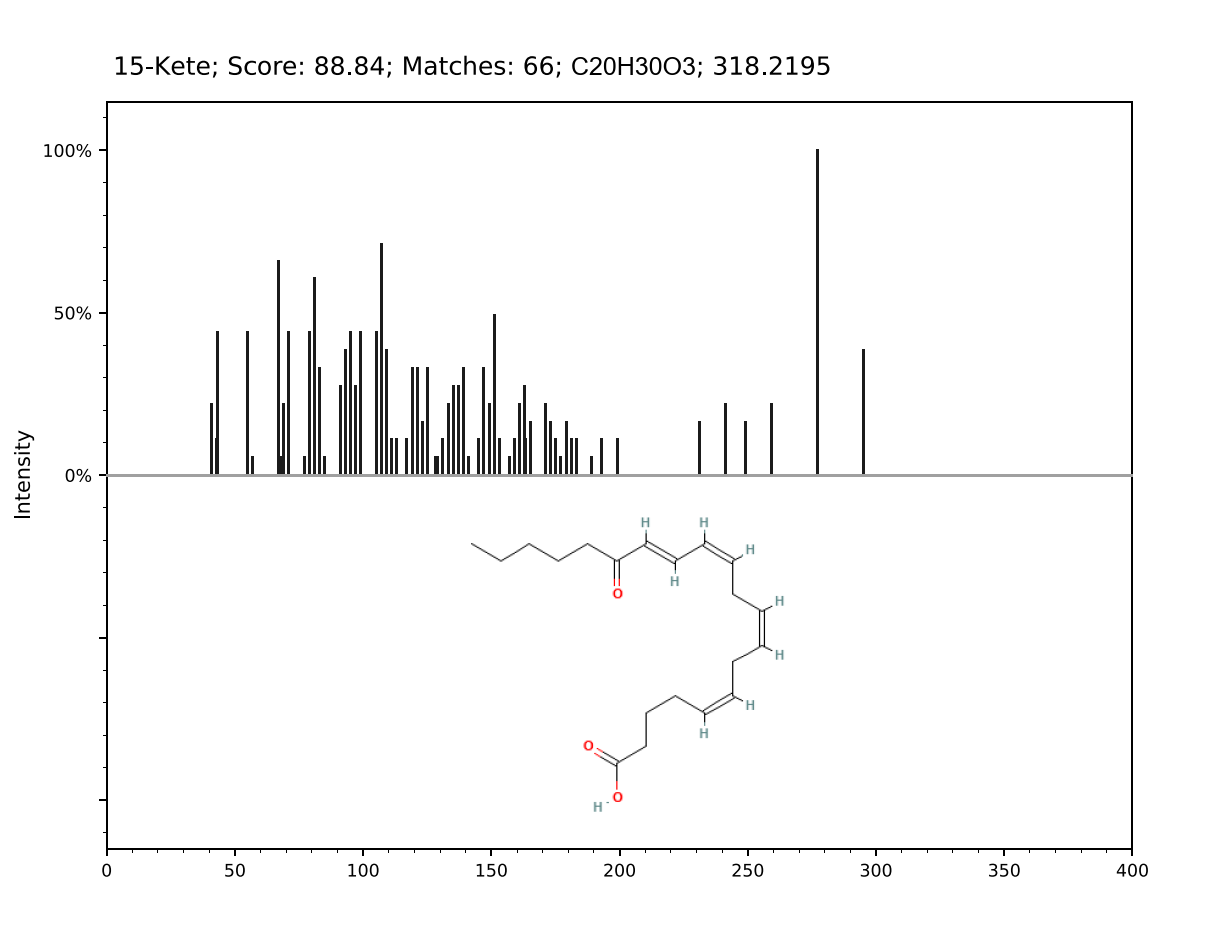 |
| 27 | Okaramine D | C_33_H_34_N_4_O_6_ | [M+H]+ | 583.2554 | 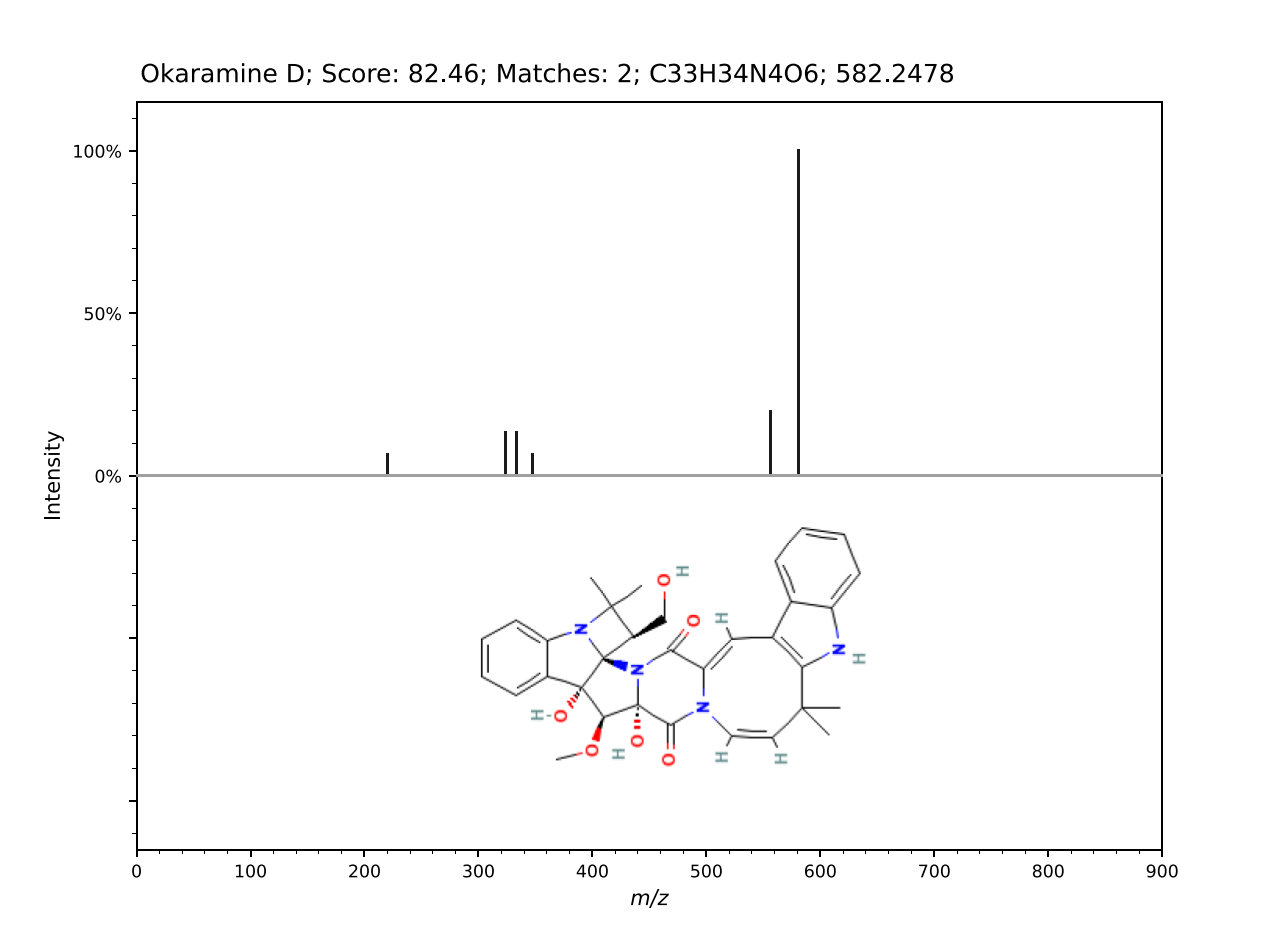 |
| 28 | Afzelin | C_21_H_20_O_10_ | [M+H]+ | 433.1131 | 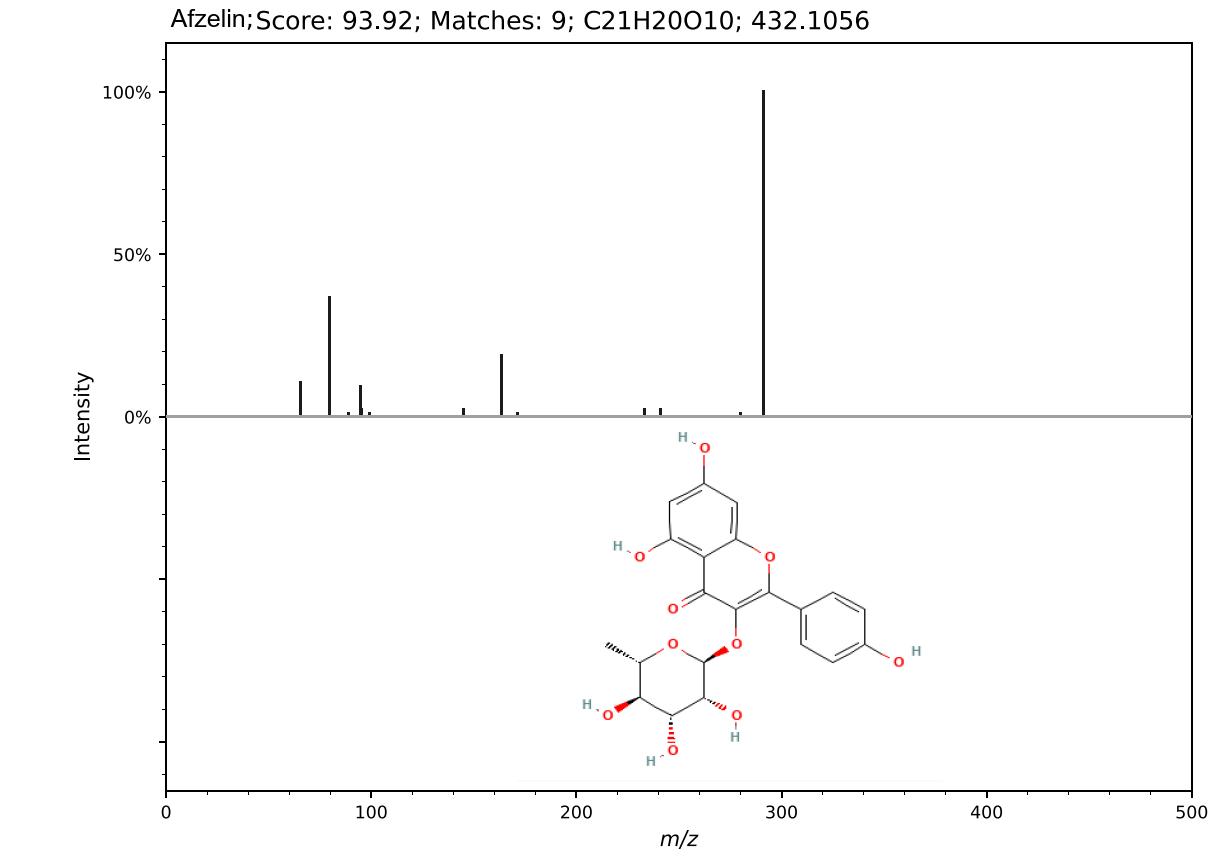 |
| 29 | Anileridine | C_22_H_28_N_2_O_2_ | [M+Na]+ | 375.2033 | 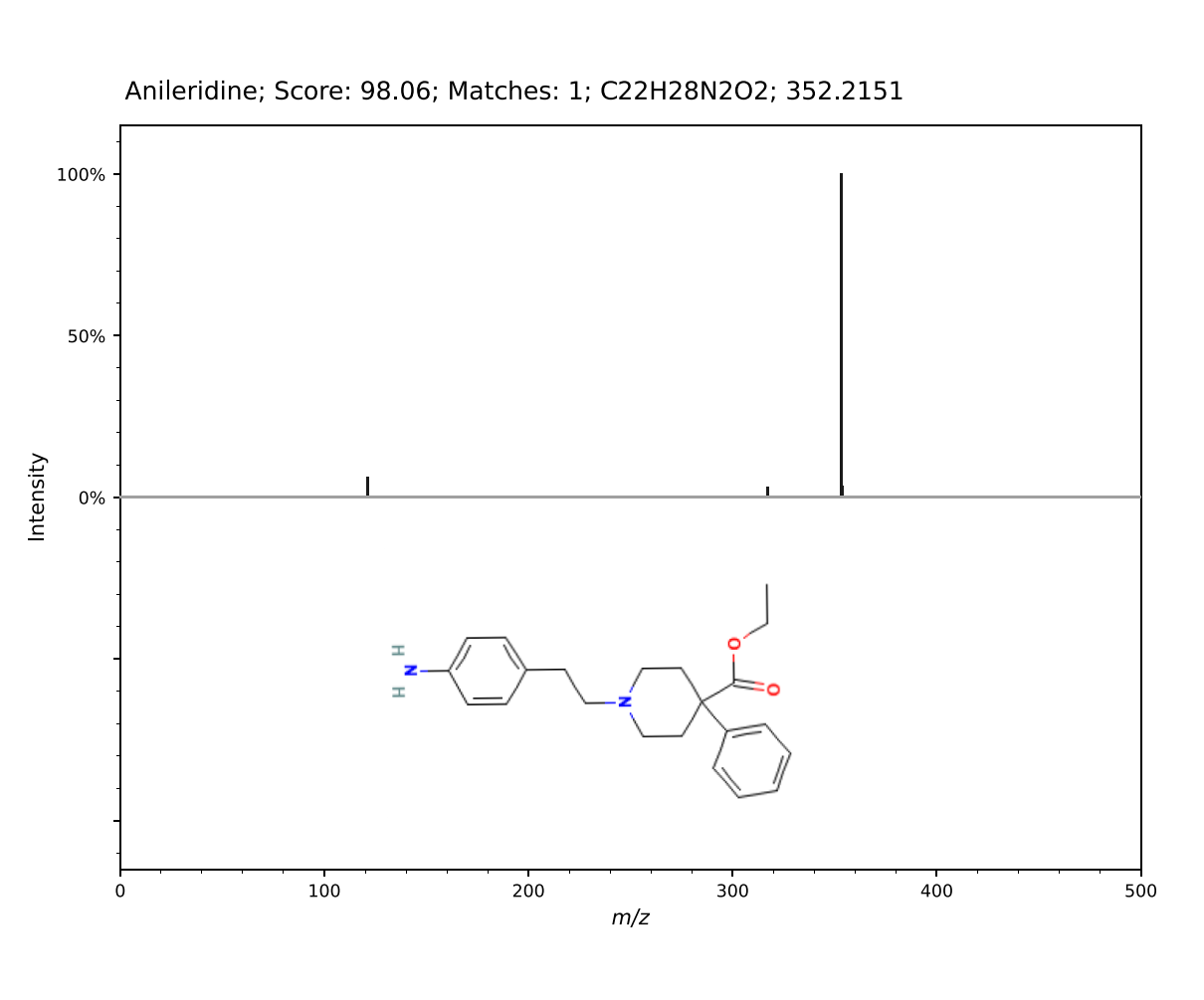 |
| 30 | Tricin 5-glucoside | C_23_H_24_O_12_ | [M+H]+ | 493.1346 | 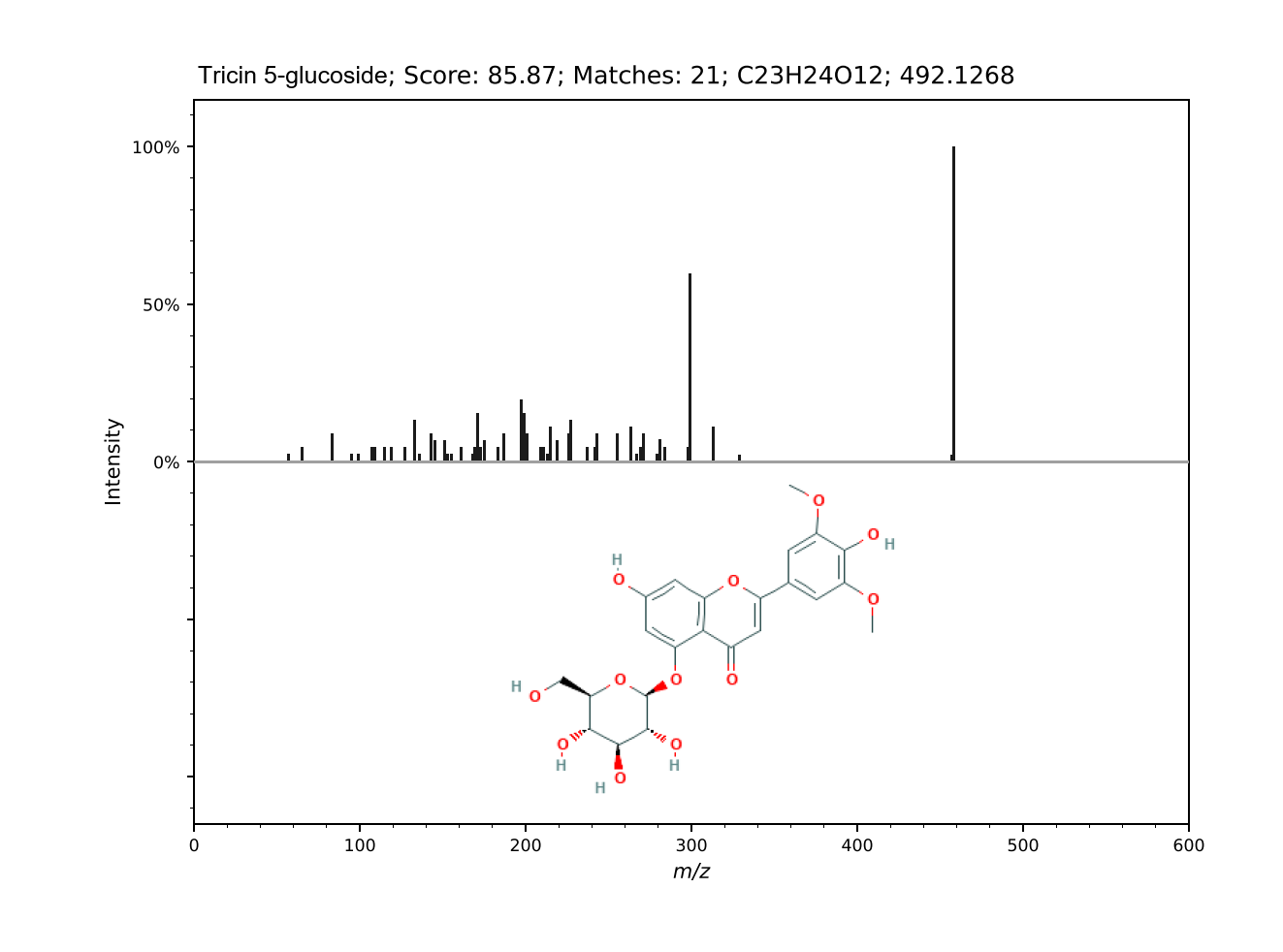 |
| 31 | 1,2-Dihydroxyheptadec-16-yn-4-yl acetate | C_19_H_34_O_4_ | [M+H]+ | 327.2535 | 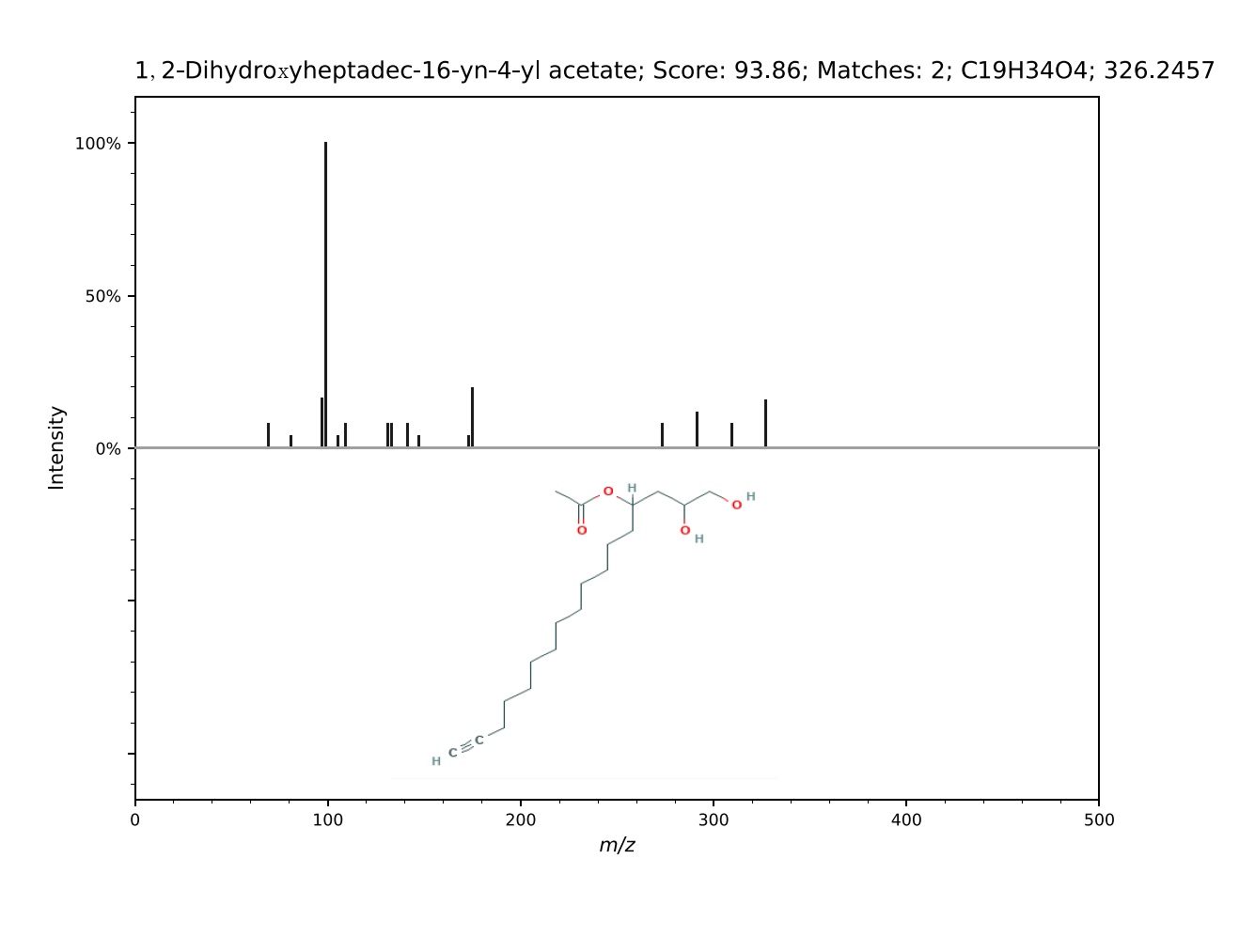 |
| 32 | Glechomafuran | C_15_H_20_O_3_ | [M+H]+ | 249.1490 | 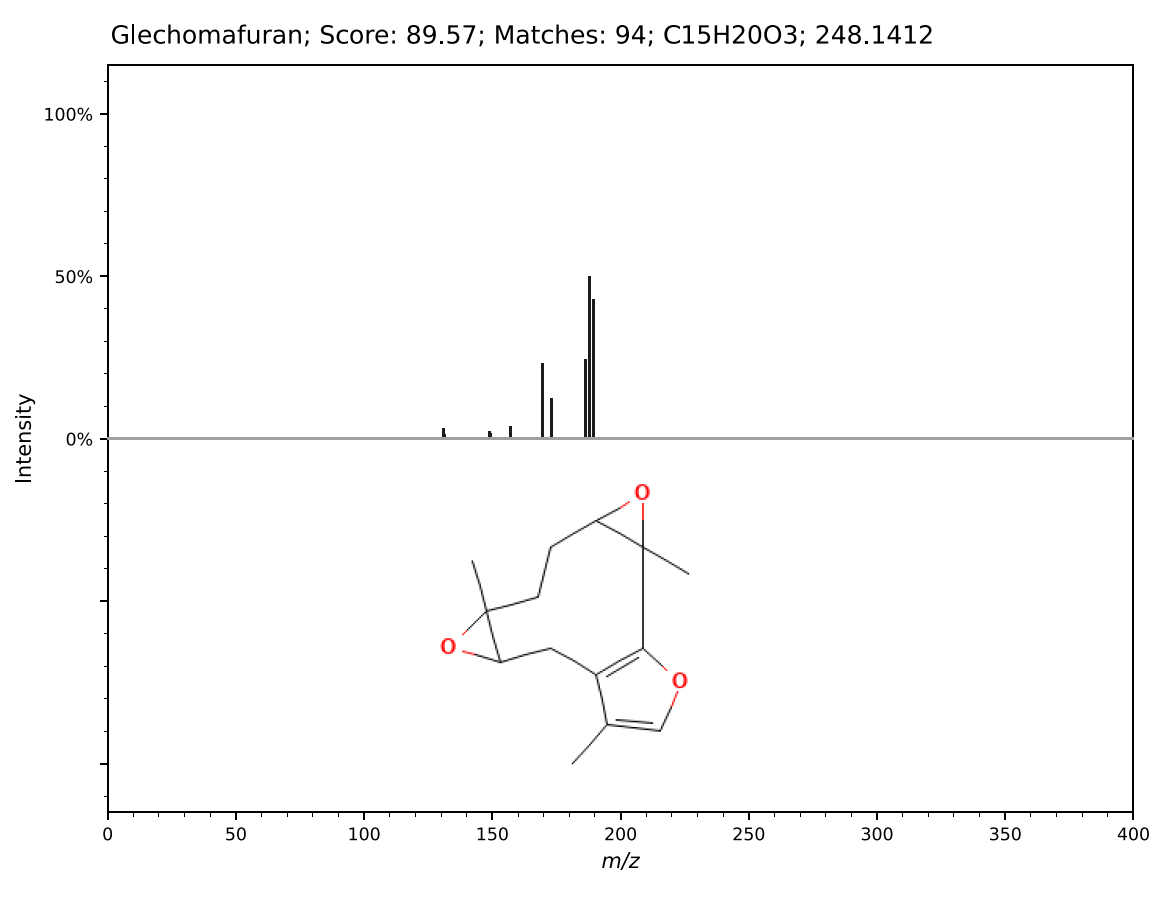 |
| 33 | 4-Methoxy-6-pentyl-2-prop-1-en-2-yl-2,3-dihydro-1-benzofuran-7-carboxylic acid | C_18_H_24_O_4_ | [M+H]+ | 305.1754 | 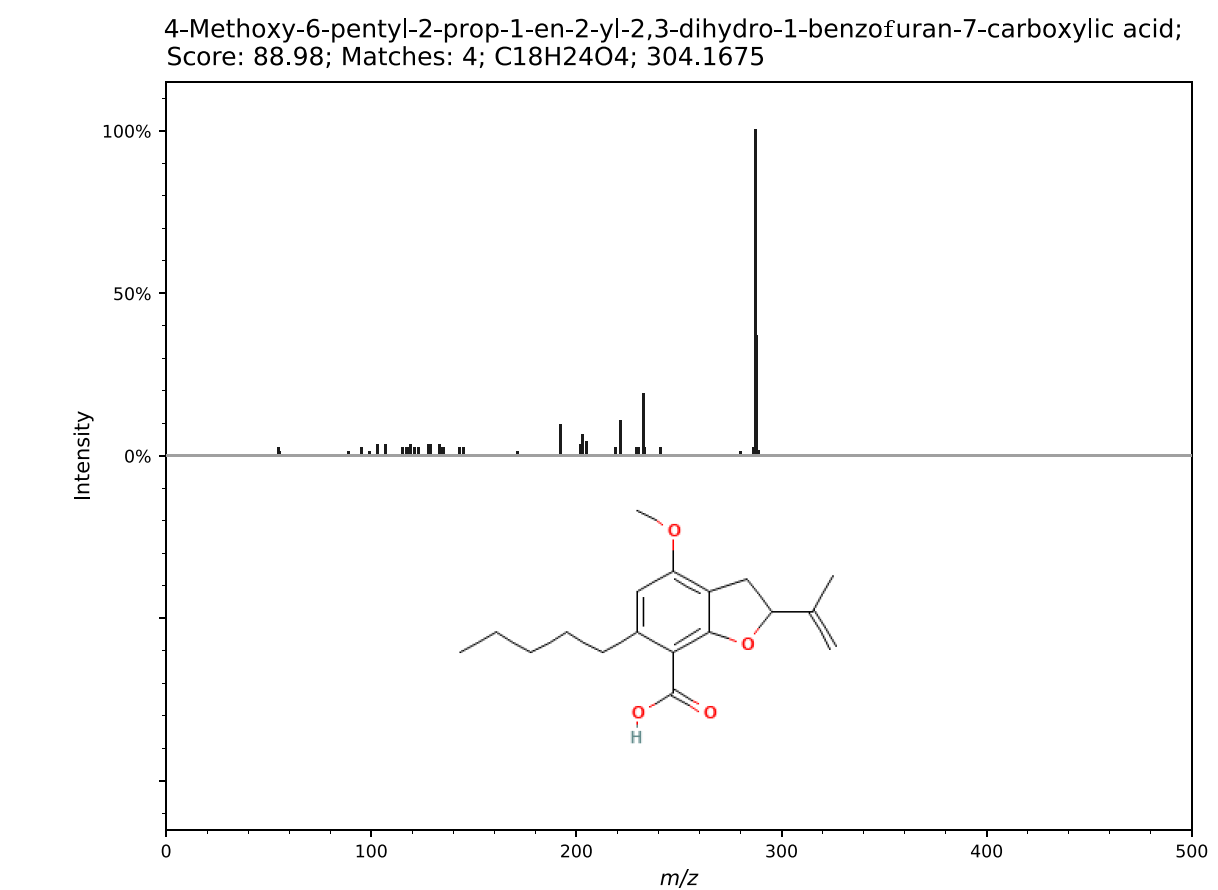 |
| 34 | Vinpocetine | C_22_H_26_N_2_O_2_ | [M+H]+ | 351.2084 | 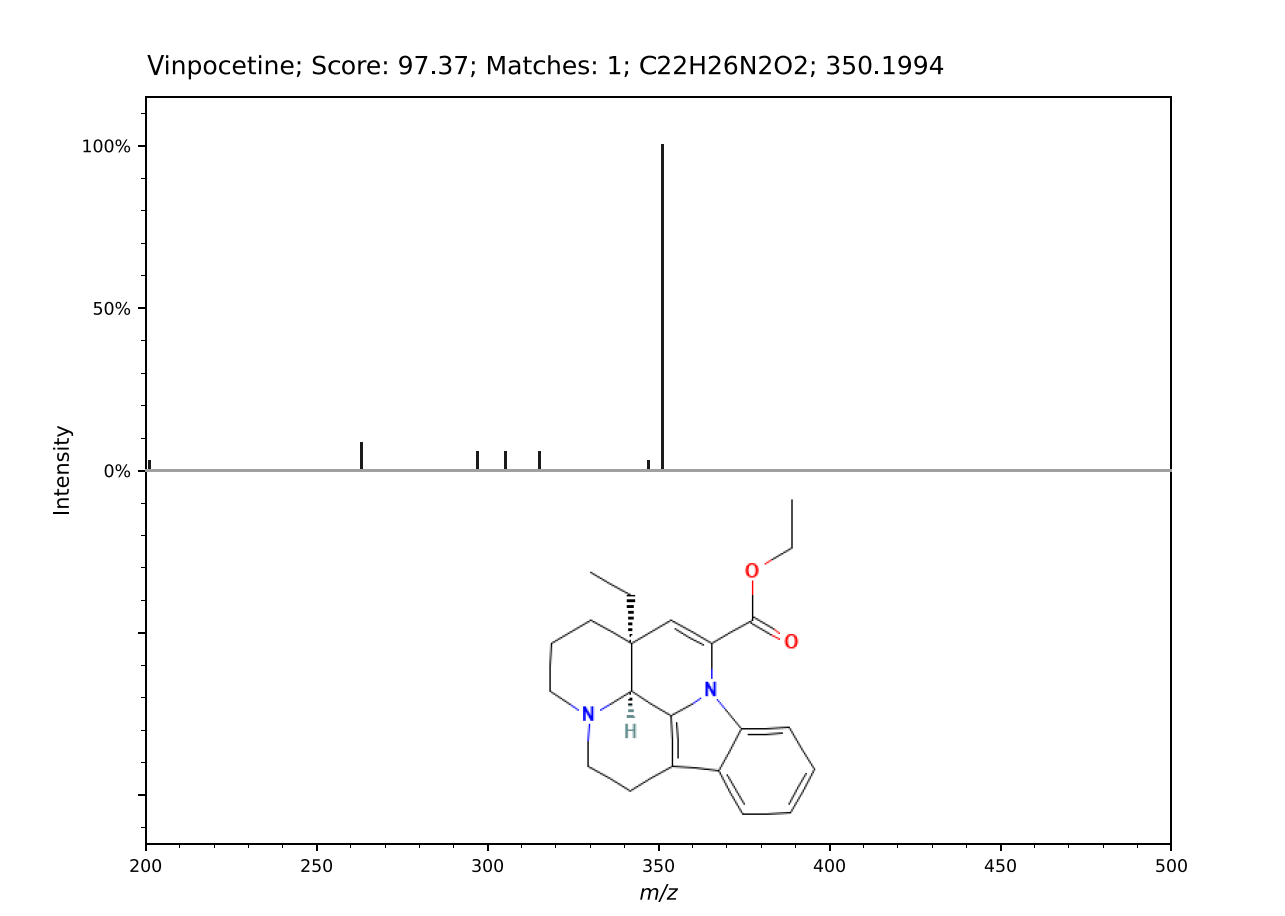 |
| 35 | Myristoylcarnitine | C_21_H_41_NO_4_ | [M+H]+ | 372.3110 | 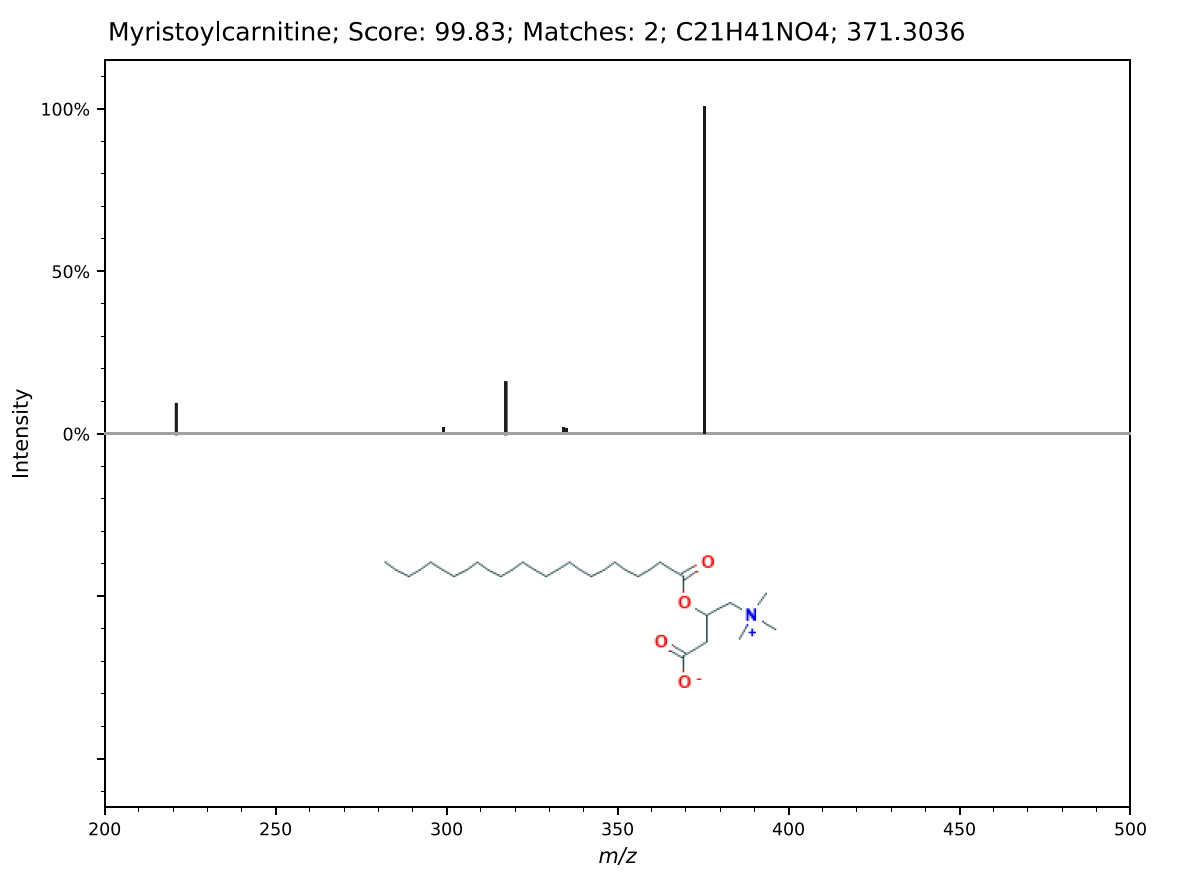 |
| 36 | Sodium Houttuyfonate | C12H23NaO5S | [M+NH4]+ | 320.1497 | 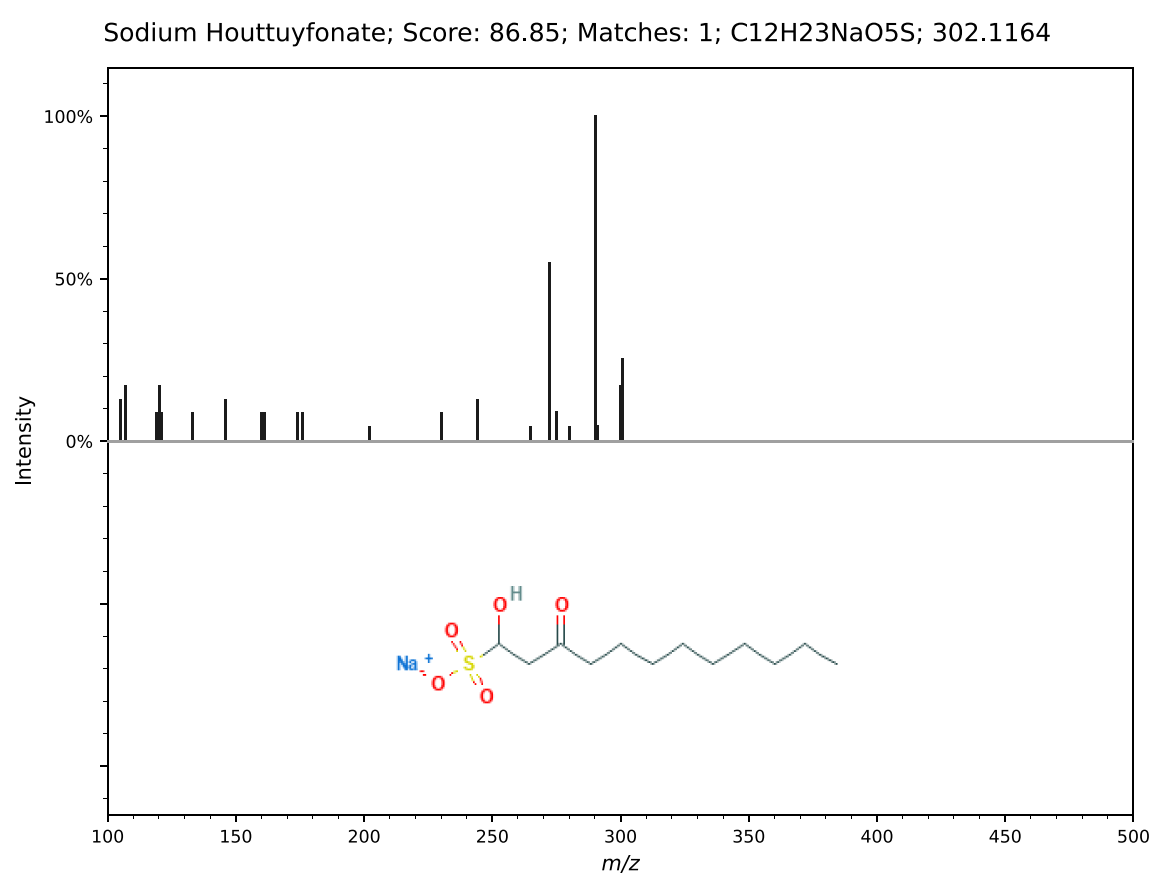 |
| 37 | Tryptamine | C_10_H_12_N_2_ | [M-H]- | 159.0917 | 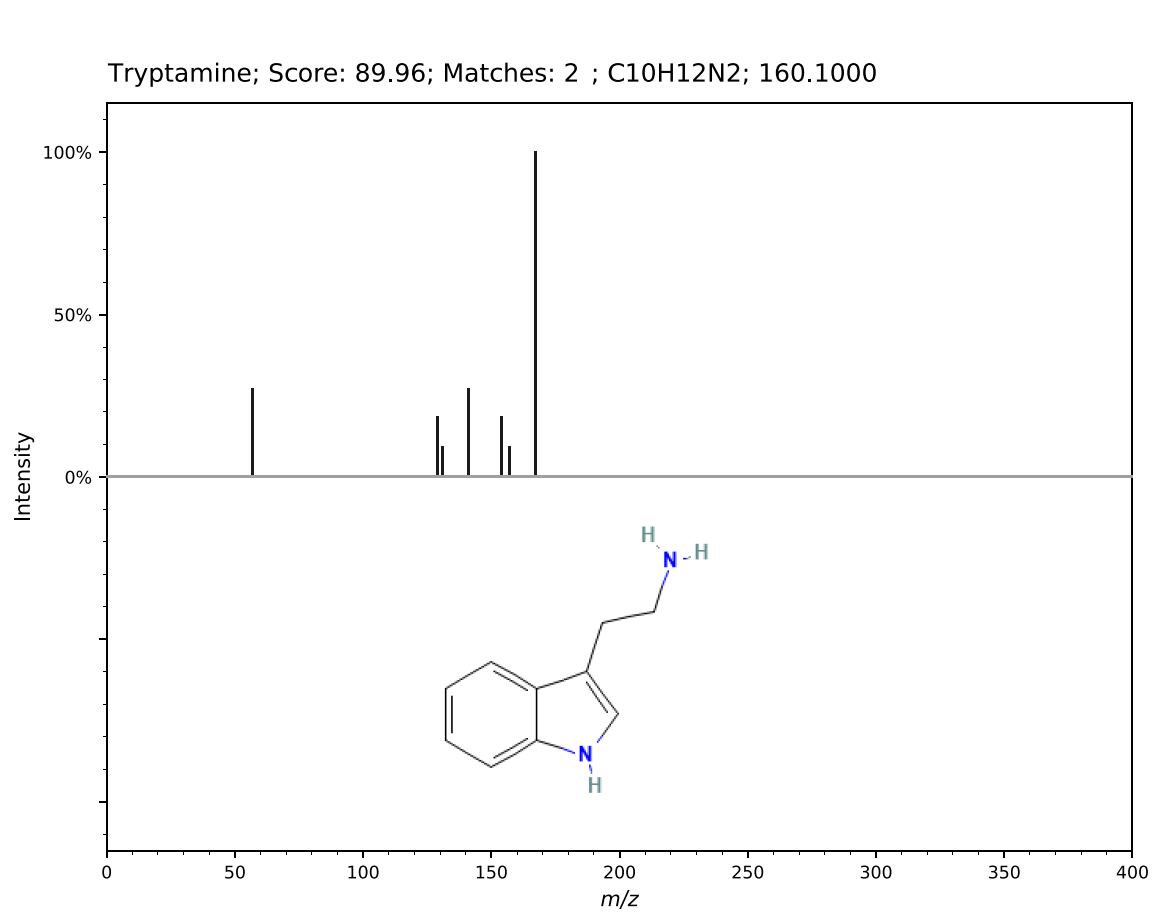 |
| 38 | 6-Pentyl-2H-pyran-2-one | C_10_H_14_O_2_ | [M-H2O-H]- | 147.0805 | 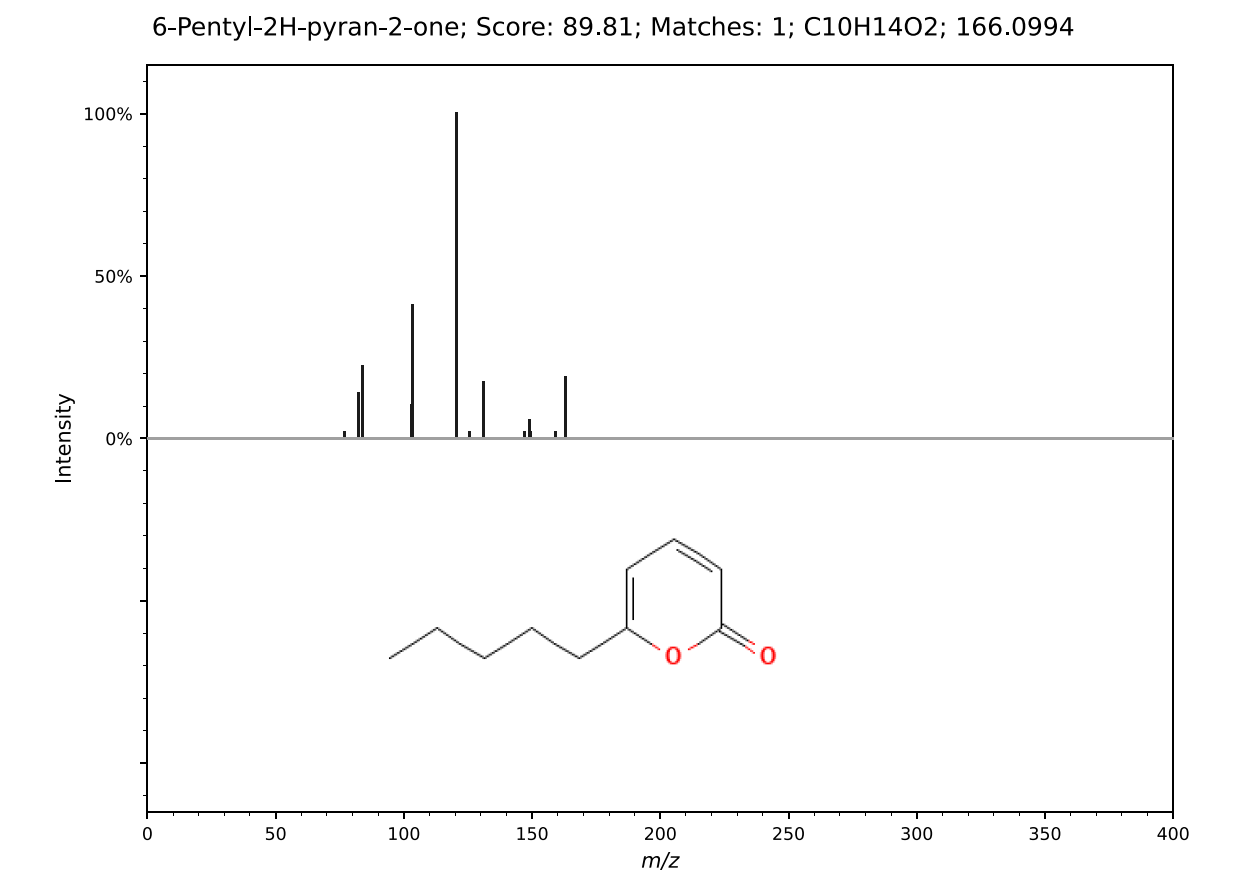 |
| 39 | Cyclomethyltryptophan | C_12_H_12_N_2_O_2_ | [M-H]- | 215.0818 | 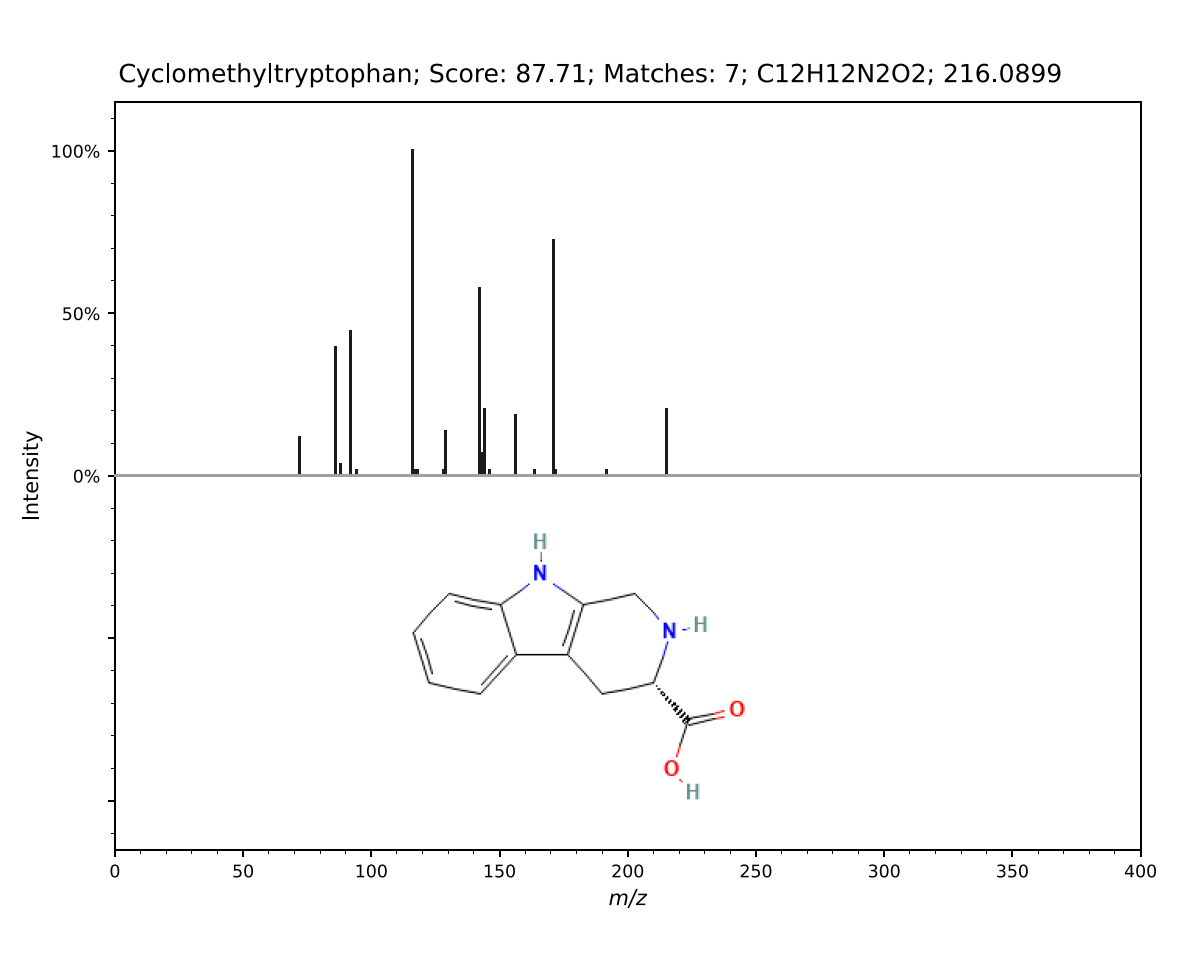 |
| 40 | Apigenin | C_15_H_10_O_5_ | [M-H]- | 269.0452 | 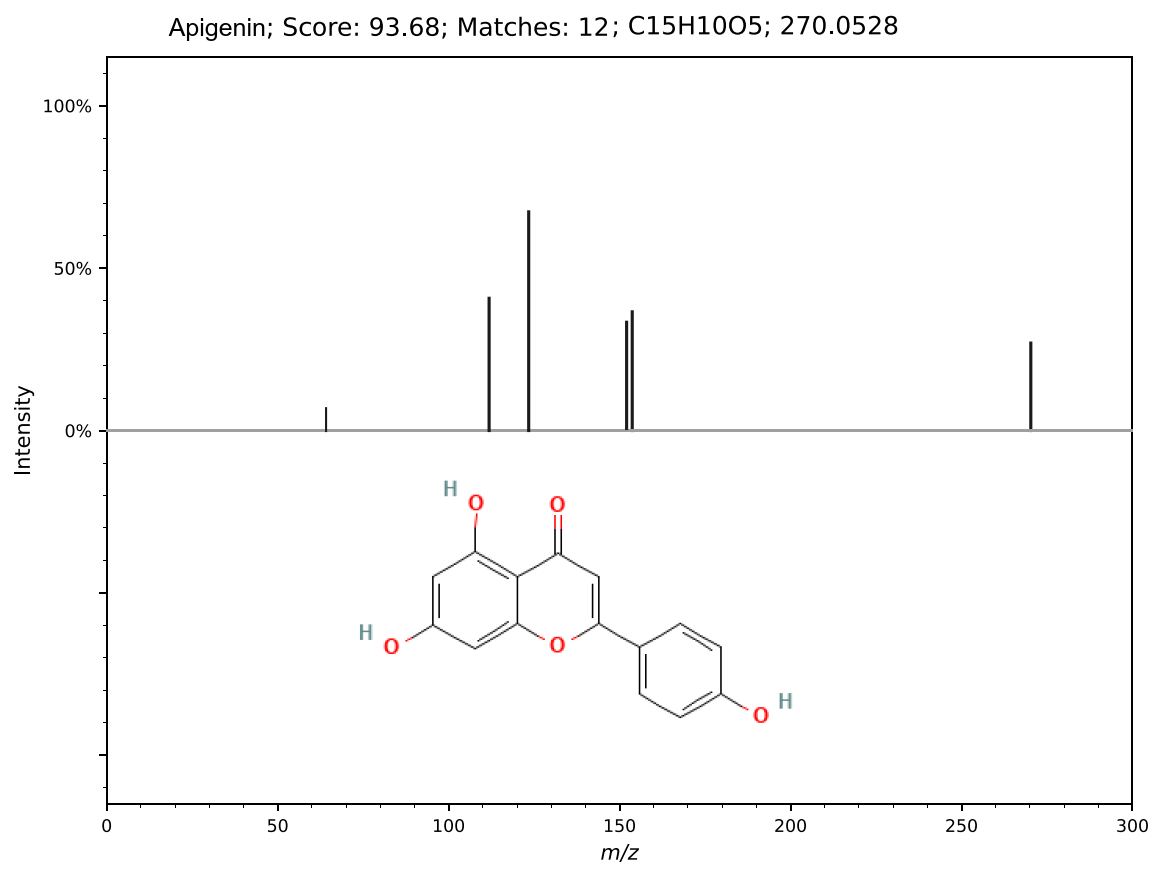 |
| 41 | 4,6-Dihydroxy-4-(hydroxymethyl)-3,4a,8,8-tetramethyl-5,6,7,8a-tetrahydronaphthalen-1-one | C_15_H_24_O_4_ | [M-H]- | 267.1592 | 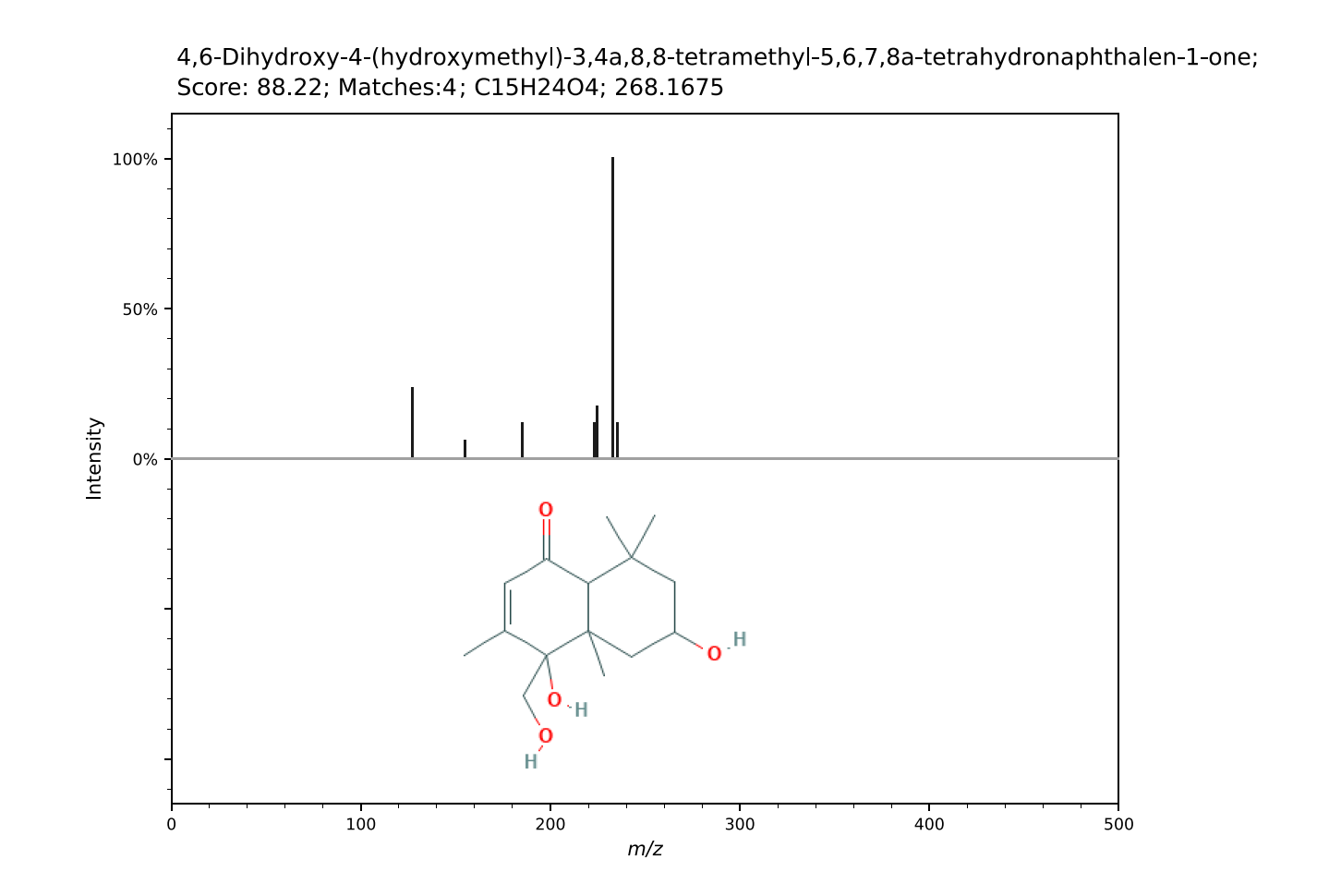 |
| 42 | (10E,15E)-12,13-dihydroxy-9-oxooctadeca-10,15-dienoic acid | C_18_H_30_O_5_ | [M-H]- | 325.2011 | 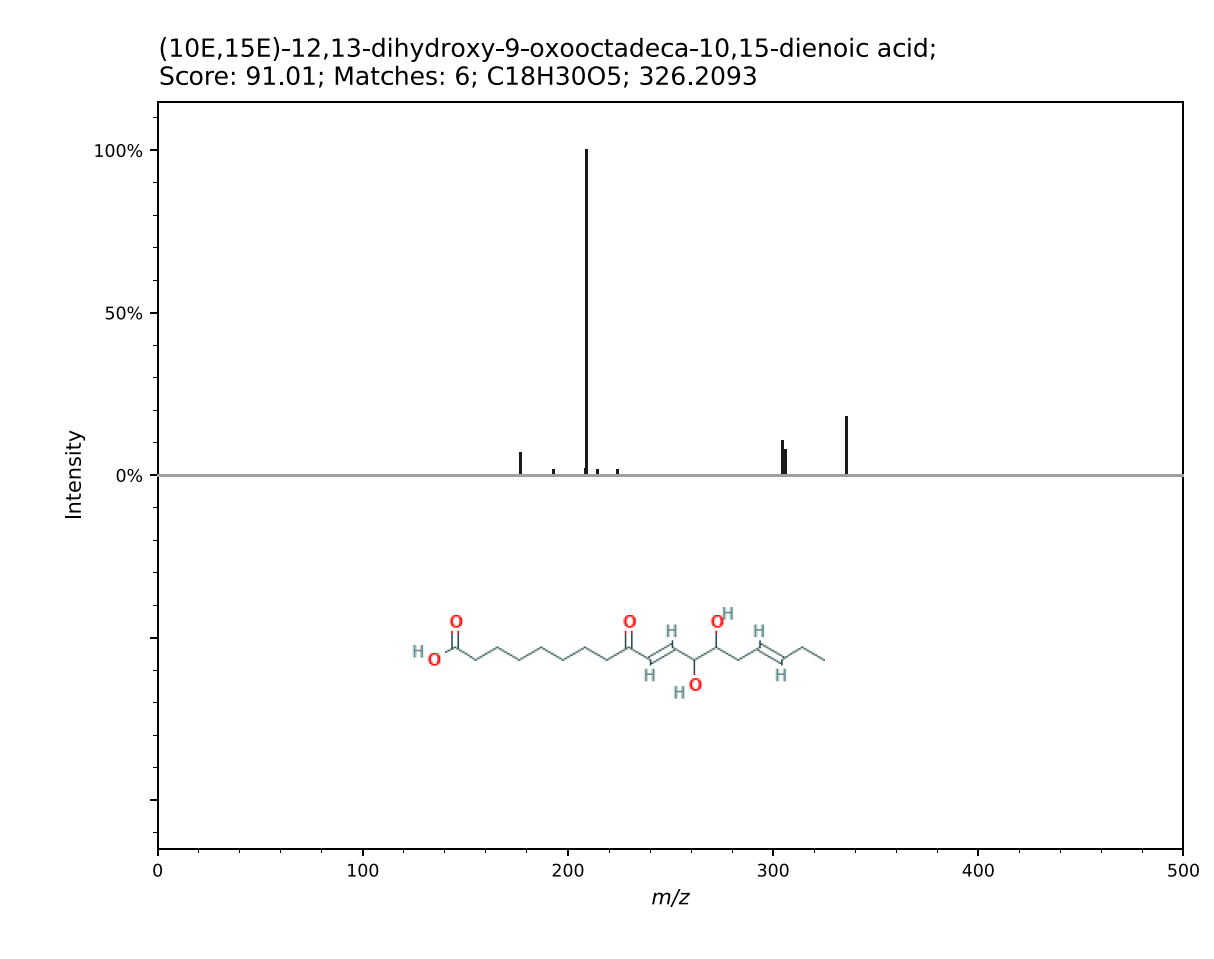 |
| 43 | Astragalin | C_21_H_20_O_11_ | [M-H]- | 447.0931 | 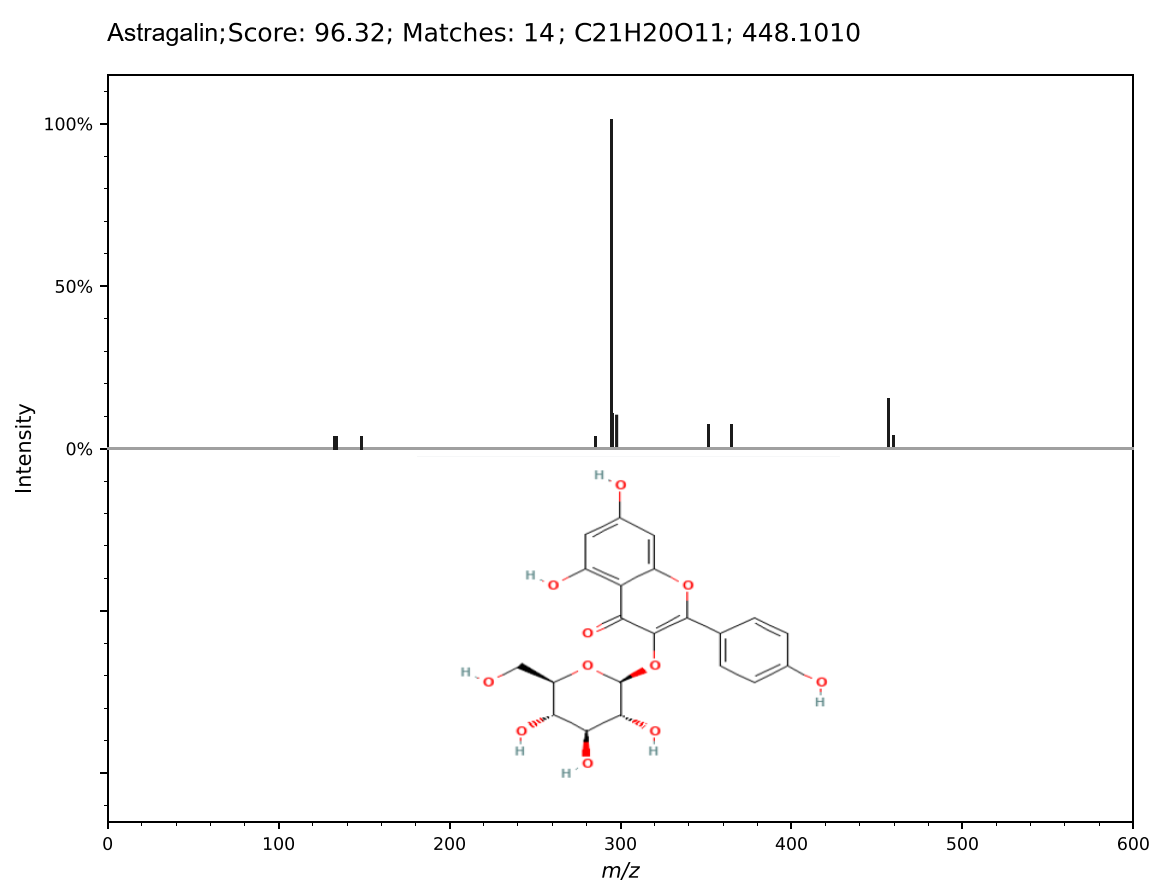 |
| 44 | 2-(3,4-dihydroxyphenyl)-3,5-dihydroxy-8-methoxy-7-[(2S,3R,4S,5S,6R)-3,4,5-trihydroxy-6-(hydroxymethyl)oxan-2-yl]oxychromen-4-one | C_22_H_22_O_13_ | [M-H]- | 493.0977 | 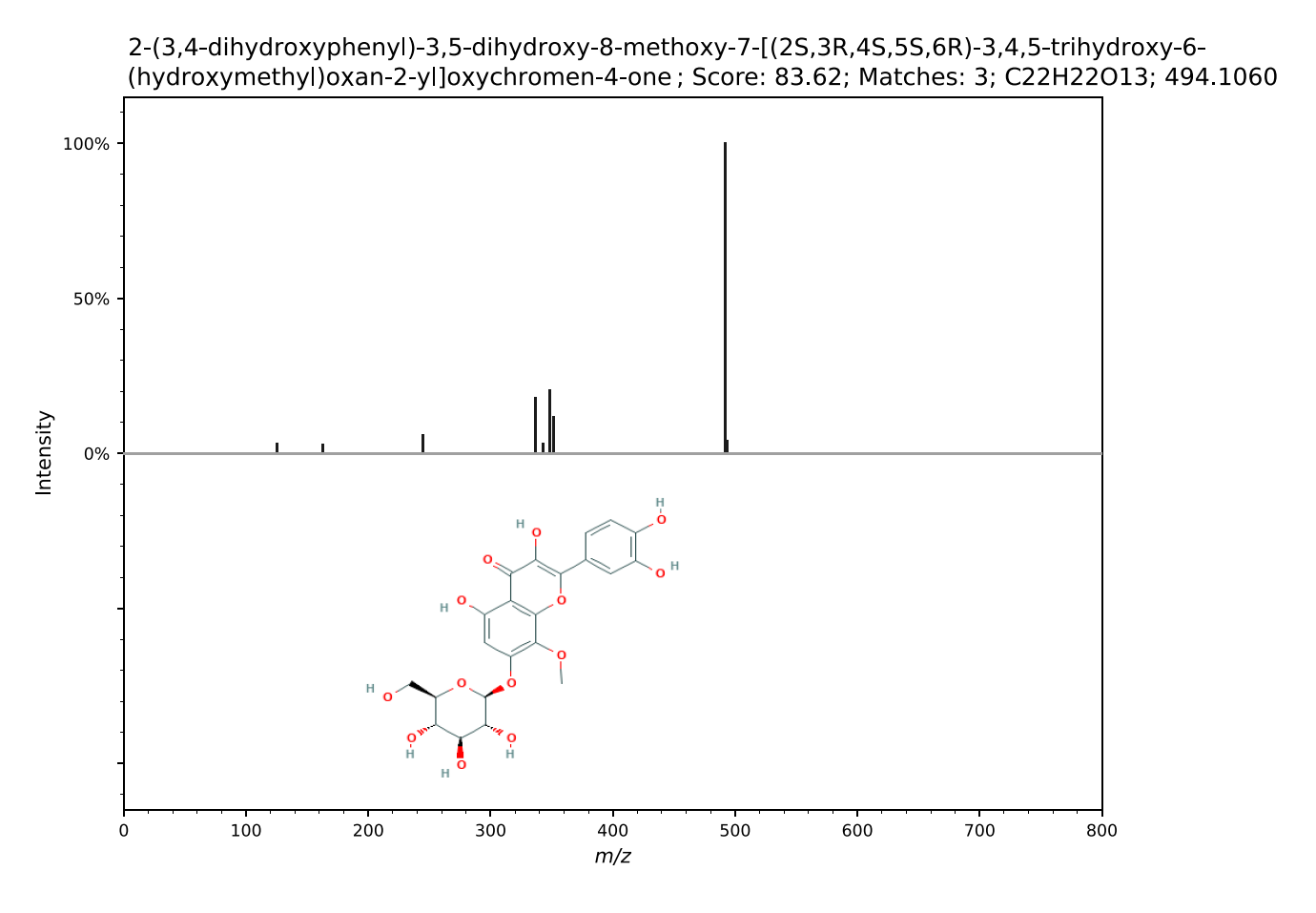 |
| 45 | Methyl 11-methoxy-19-methyl-16,17-didehydro-18-oxayohimban-16-carboxylate | C_22_H_26_N_2_O_4_ | [M-H]- | 381.1816 | 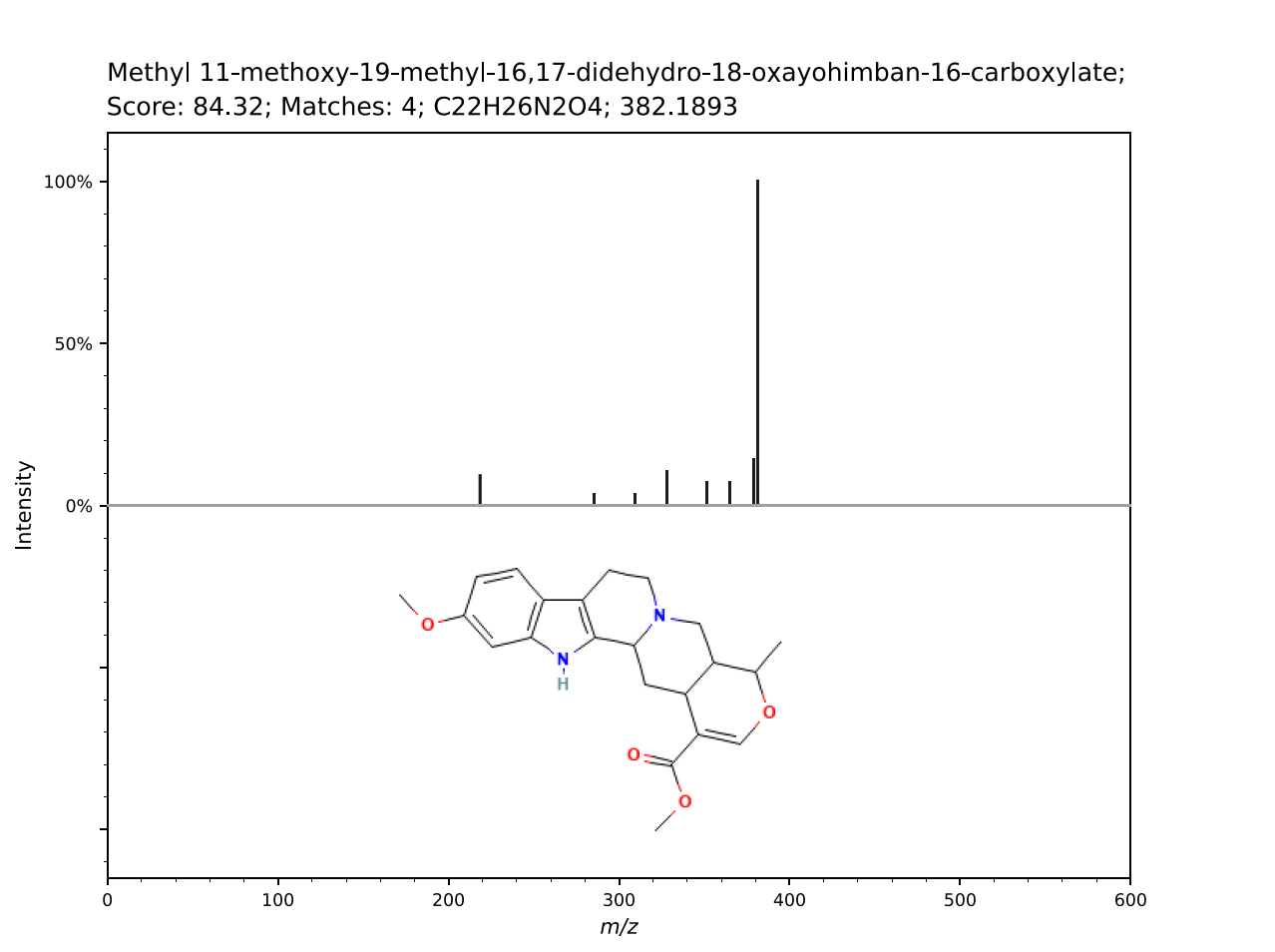 |
| 46 | Luteolin 7-(6''-malonylglucoside) | C_24_H_22_O_14_ | [M-H]- | 533.0928 | 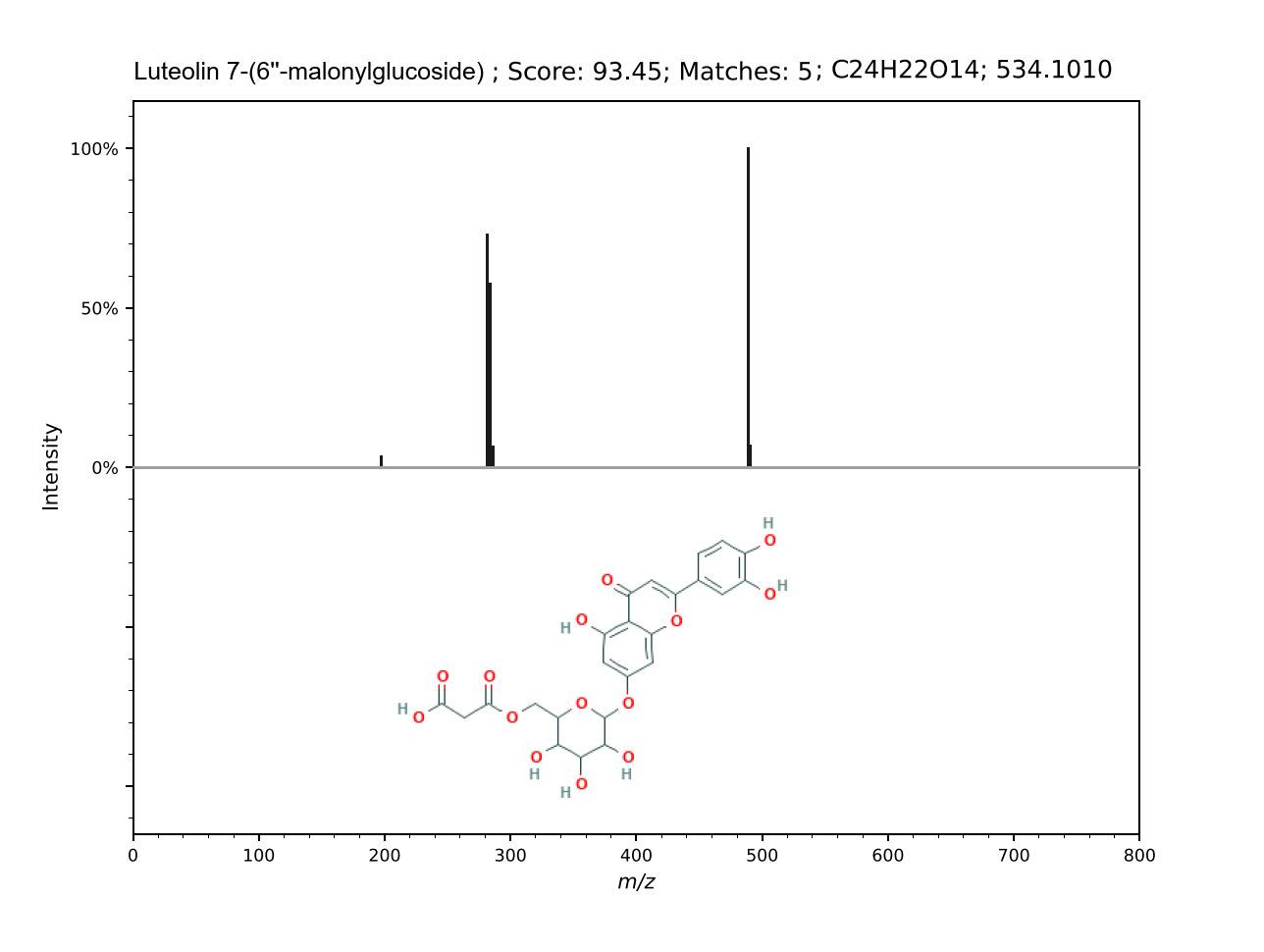 |
| 47 | Isoschaftoside | C_26_H_28_O_14_ | [M-H]- | 563.1413 | 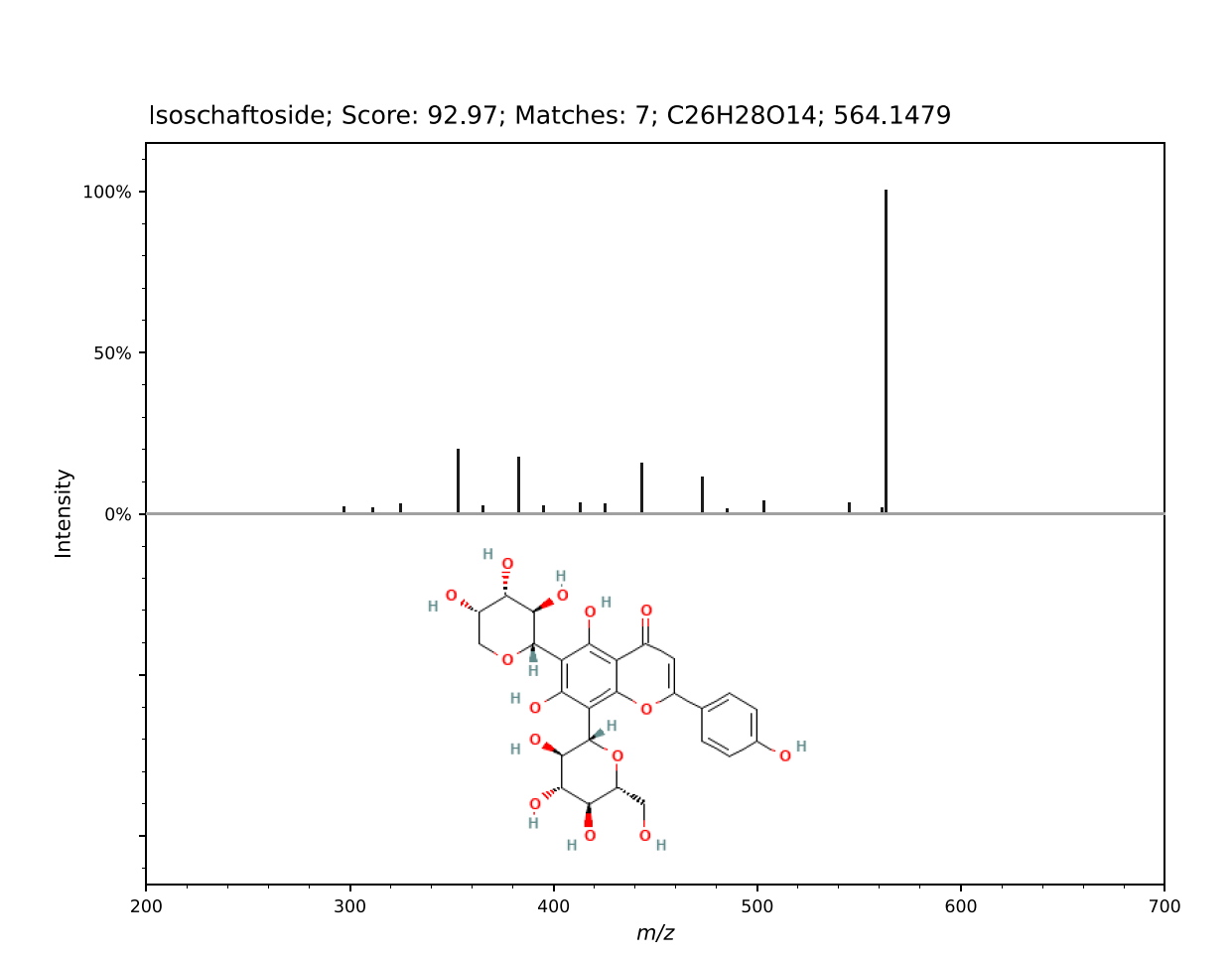 |
| 48 | Kaempferol-3-O-glucorhamnoside | C_27_H_30_O_15_ | [M-H]- | 593.1503 | 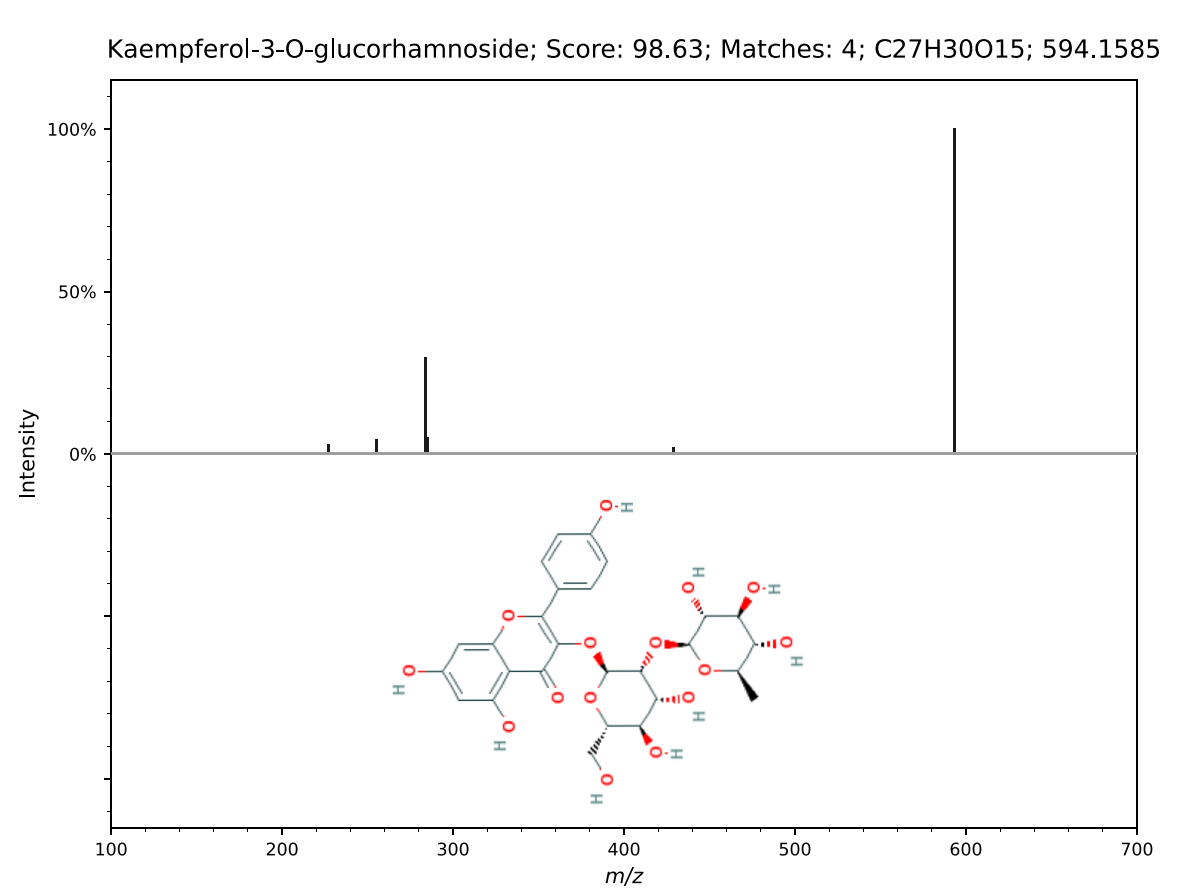 |
| 49 | Kaempferol 7-neohesperidoside | C_27_H_30_O_15_ | [M+Cl]- | 629.1290 | 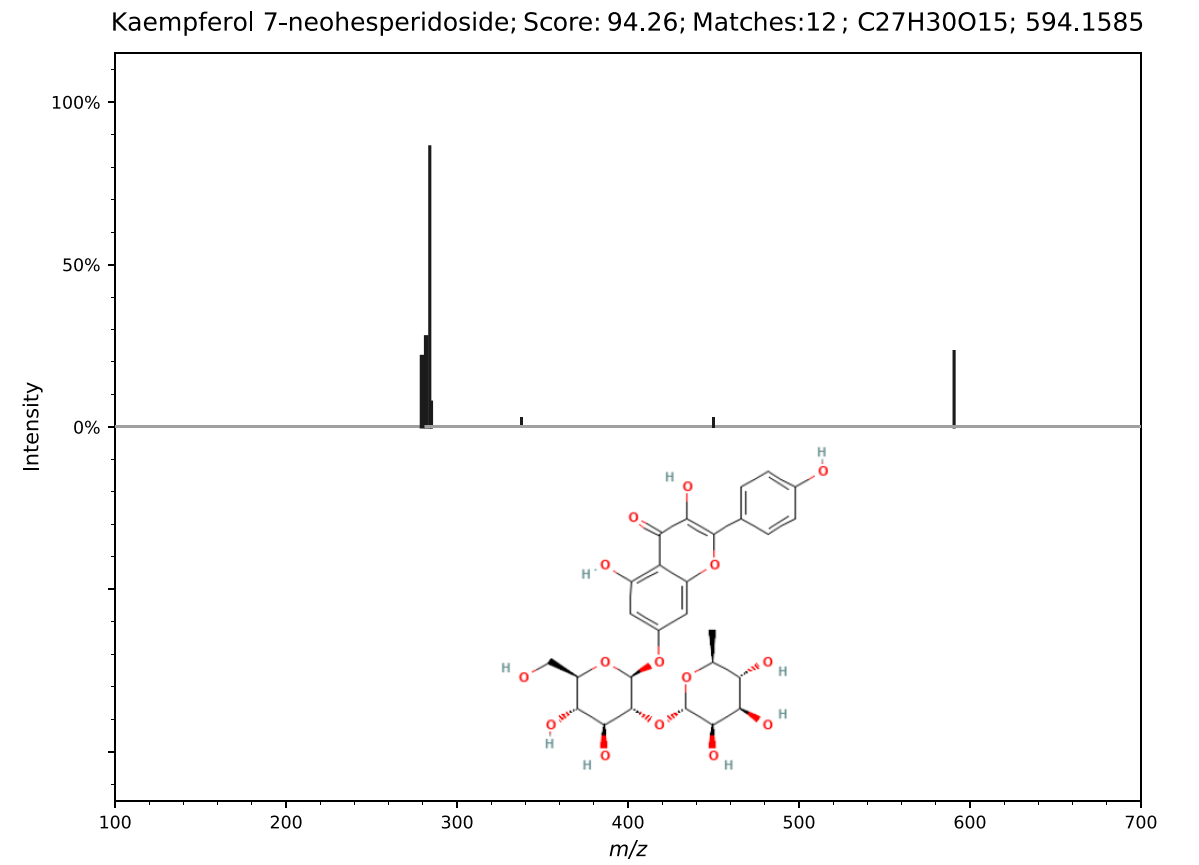 |
| 50 | Rutin | C_27_H_30_O_16_ | [M-H]- | 609.1467 | 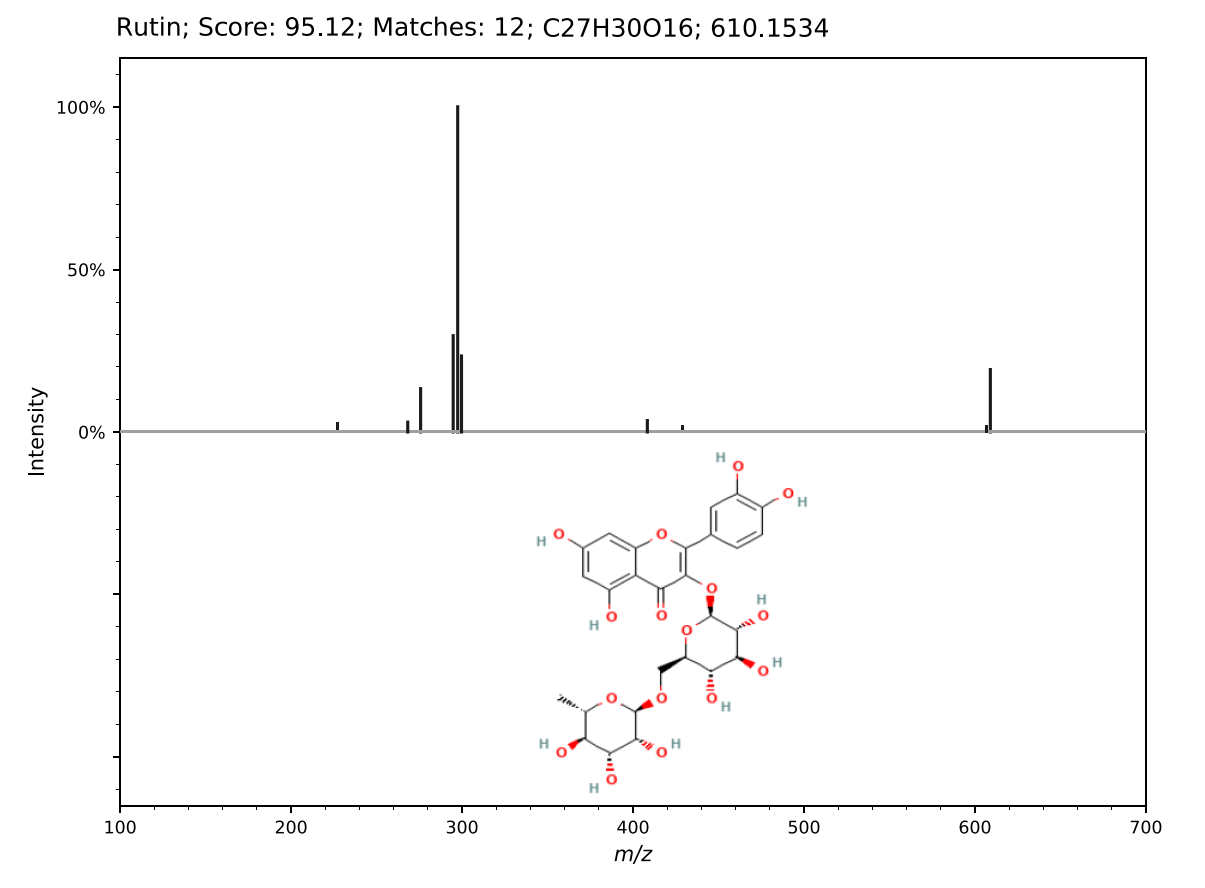 |
| 51 | Rhamnetin 3-sophoroside | C_28_H_32_O_17_ | [M-H]- | 639.1557 | 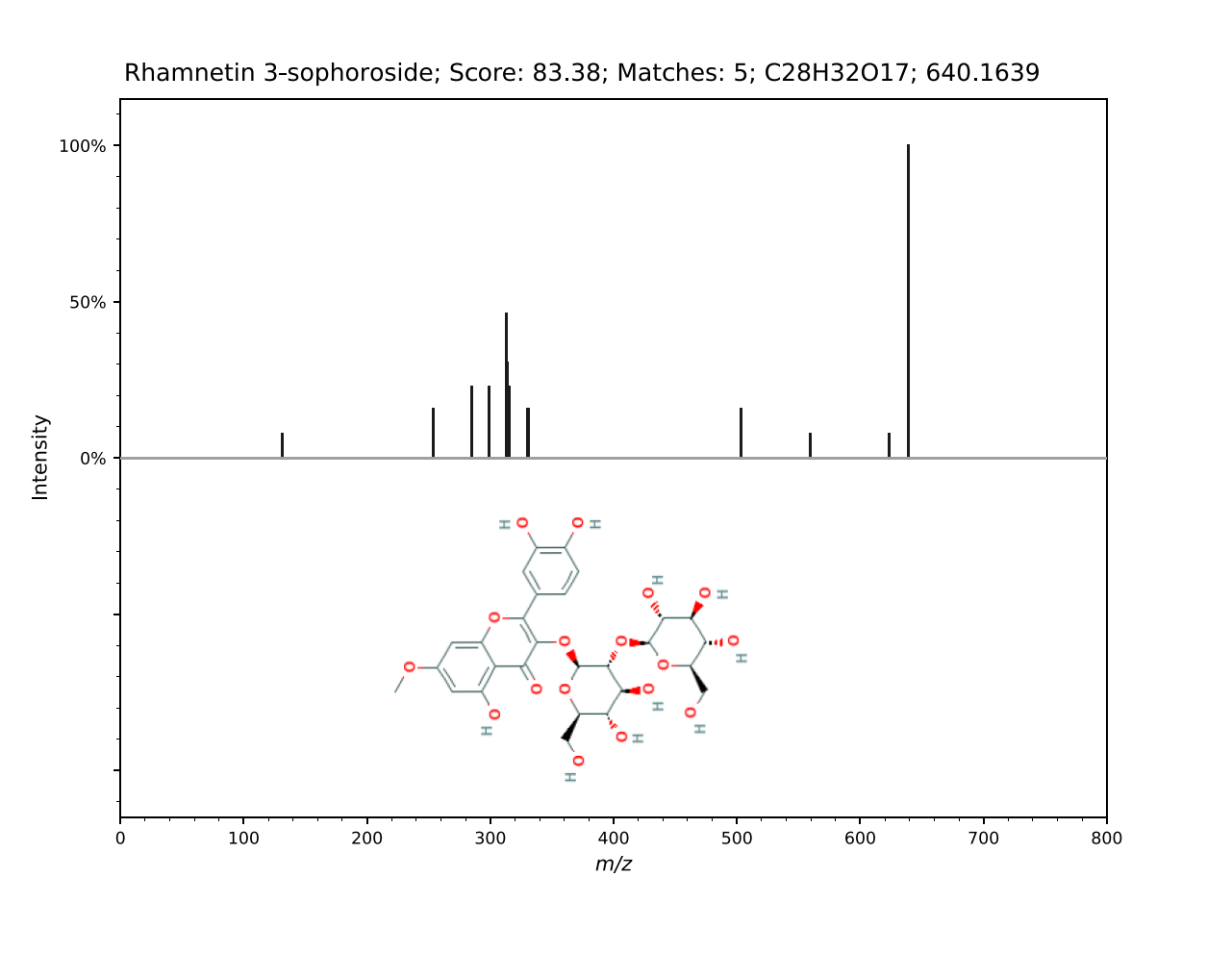 |
| 52 | cis-Aconitic acid | C_6_H_6_O_6_ | [M-H]- | 173.0075 | 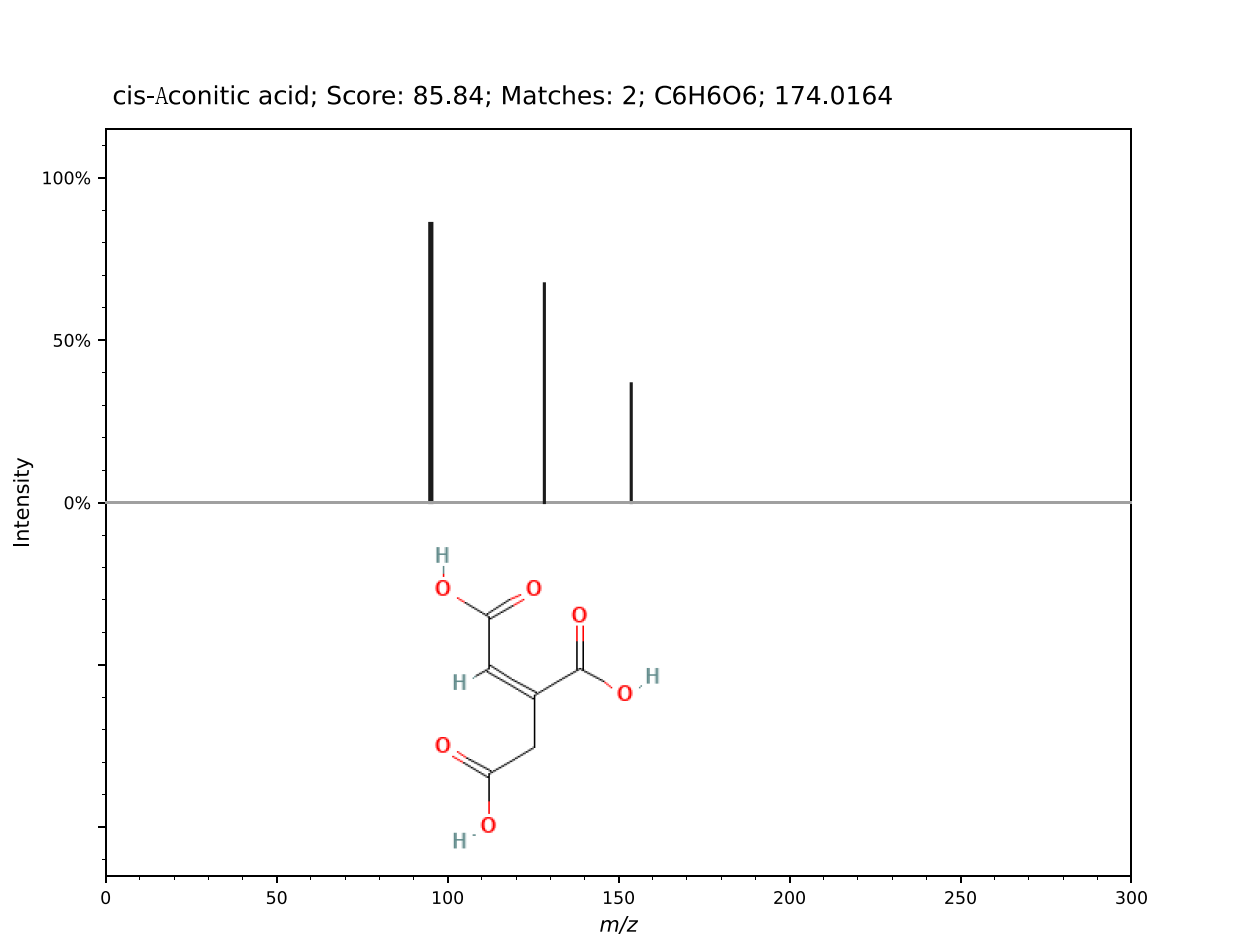 |
| 53 | Pyridoxal | C_8_H_9_NO_3_ | [M-H]- | 166.0502 | 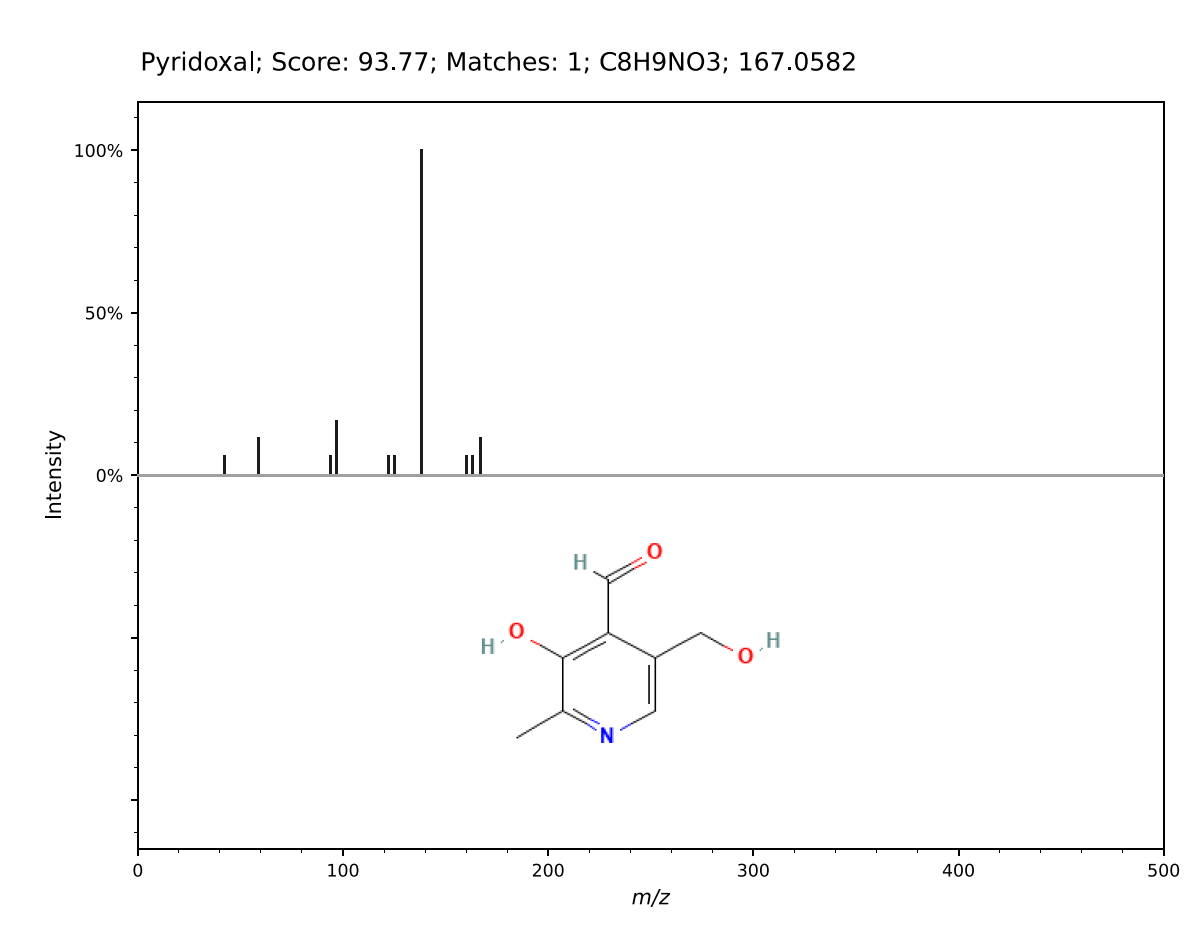 |
| 54 | 2-(4-Hydroxyphenyl)propanoic acid | C_9_H_10_O_3_ | [M-H]- | 165.0551 | 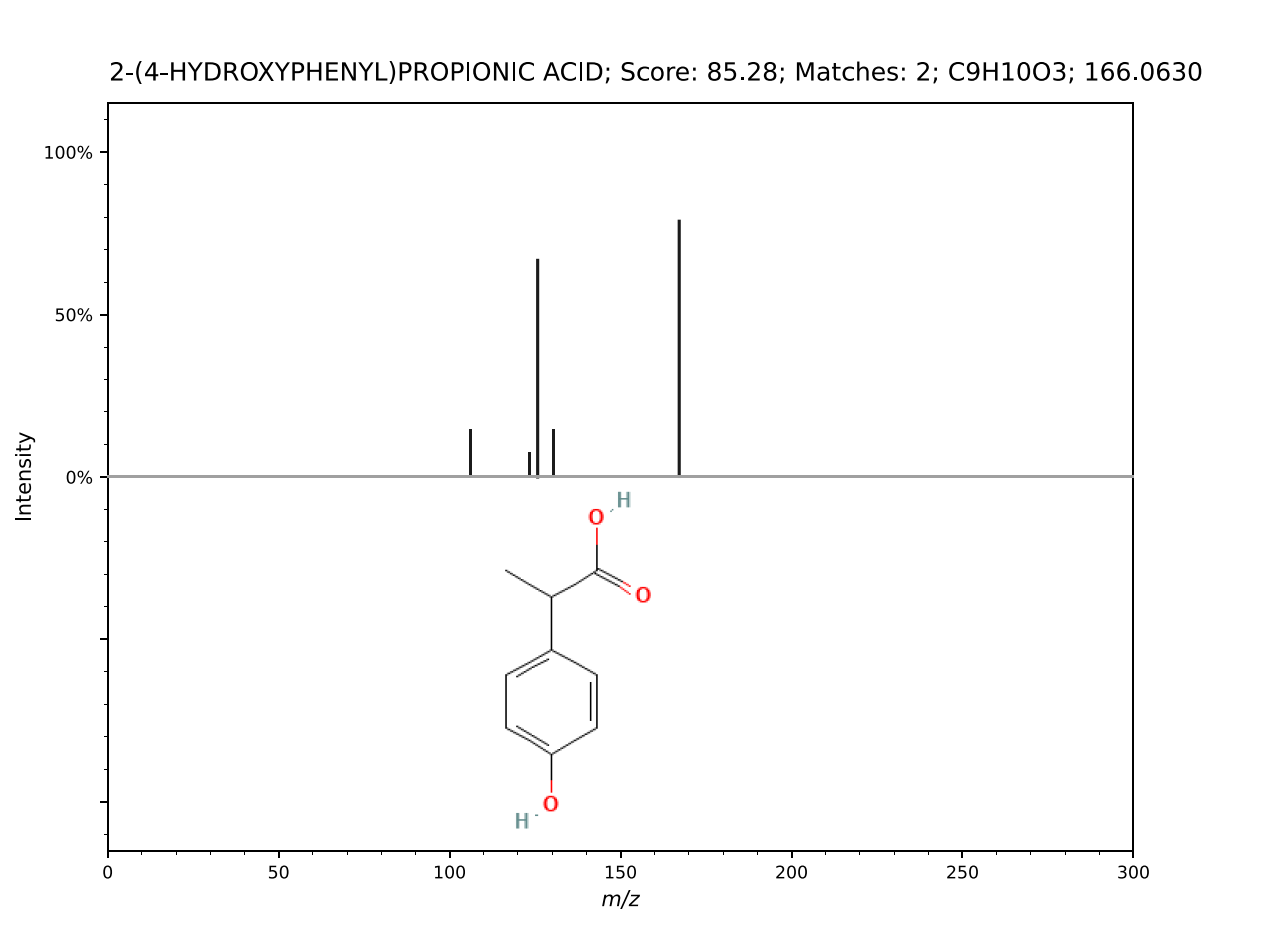 |
