# Supplementary figures and images for: Geographical variation in metabolite profiles and bioactivity of Thesium chinense Turcz. revealed by UPLC-Q-TOF-MS-based metabolomics
Source: Front Plant Sci. 2025 Jan 10;15:1471729. doi: 10.3389/fpls.2024.1471729 (PMC11760594; doi:10.3389/fpls.2024.1471729)

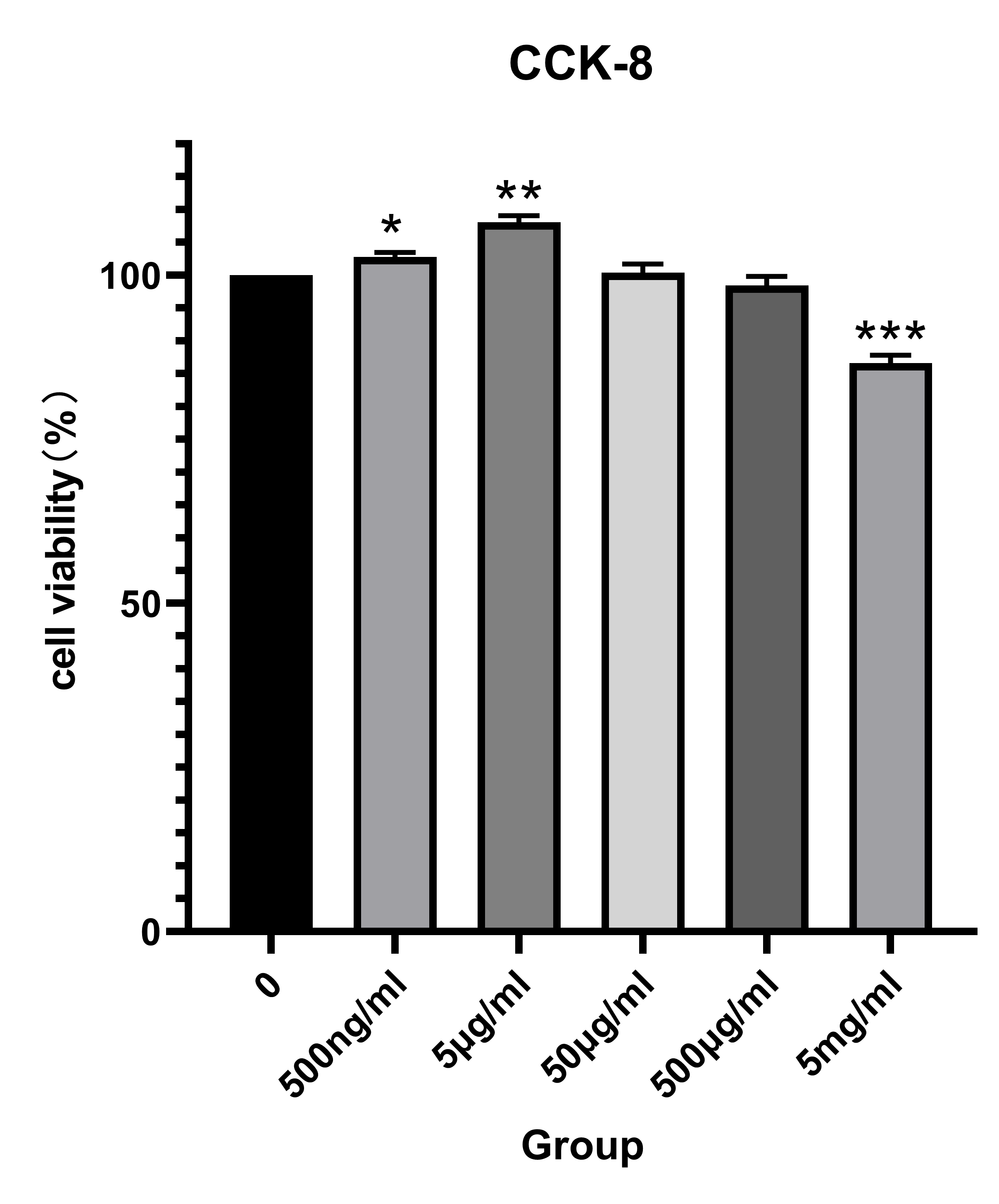

Supplement: Supplementary file 6 [file Image1.tif]
